# Titanium‐Mediated Rearrangement of Bis(alkynyl)boranes: B─C Activation versus C─H Activation

**DOI:** 10.1002/anie.202504229

**Published:** 2025-05-19

**Authors:** Chenchang Ma, Alexander Matler, Shuai Zhu, Thayalan Rajeshkumar, Laurent Maron, Qing Ye

**Affiliations:** ^1^ Institute for Inorganic Chemistry and Institute for Sustainable Chemistry & Catalysis with Boron Julius‐Maximilians‐Universität Würzburg 97074 Würzburg Germany; ^2^ Department of Chemistry Southern University of Science and Technology Shenzhen 518055 P.R. China; ^3^ Laboratoire de Physique et Chimie des Nanoobjets, INSA, CNRS, UPS Université de Toulouse Toulouse 31077 France

**Keywords:** Alkynyl, Bond Activation, Borane, Structure Elucidation, Titanium

## Abstract

Reactions between Rosenthal's titanocene (Cp_2_Ti) and decamethyltitanocene (Cp*_2_Ti) synthons with various bis(alkynyl)boranes were investigated. A series of titanium‐fused boracyclobutenes were obtained through the reaction of the Cp*_2_Ti synthon with (Me_3_Si)_2_NB(CCR)_2_ or PhB(CCPh)_2_. This represents the first realization of approaching this structural motif via the rearrangement of bis(alkynyl)boranes within the coordination sphere of a *d*‐block metal. These findings break the previous limitation that the boron substituent must be an amino group. Furthermore, the reaction of the Cp*_2_Ti synthon with (Me_3_Si)PhNB(CCPh)_2_ or (Mes_2_B)PhNB(CCPh)_2_ (Mes = 2,4,6‐trimethylphenyl) led to the formation of novel tethered metallocene complexes, in which the titanacyclopentadiene structure is linked to one of the cyclopentadienyl groups via a ─CH_2_─BR─ bridge. Both experimental and theoretical studies provided insights into an unprecedented reaction mechanism. The process involves the initial formation of an *η* ^2^‐coordinated bis(alkynyl)borane intermediate, which was detected and analyzed by NMR spectroscopy and X‐ray diffraction analysis. This intermediate subsequently undergoes either B─C_alkynyl_ bond activation or C_Me_─H activation of the Cp* ligand, leading to the formation of two distinct types of products.

## Graphical abstract

Bis(alkynyl)boranes bearing different *B*‐substituents reacted with Cp*_2_Ti(Me_3_SiCCSiMe_3_), leading to the formation of B─C activation products (top right) and C─H activation products (bottom right), respectively.

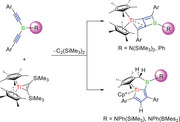

## Introduction

**Figure 1:**
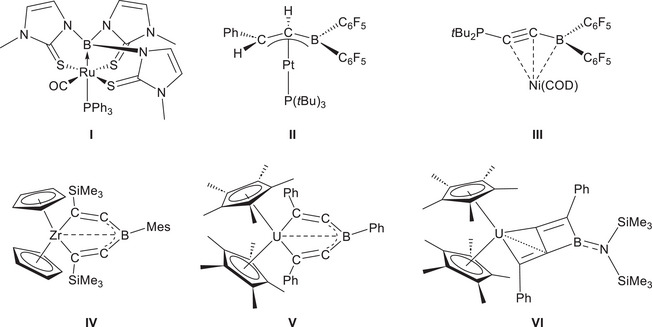
Selected examples of borane‐transition metal complexes. **I**: First boratrane complex. **II**: *η* ^*3*^‐vinylborane complex. **III**: *η* ^3^‐BCC‐coordinated alkynylborane complex. **IV**: First *η* ^5^‐coordinated bis(alkynyl)borane zirconocene complex. **V**: First *η* ^5^‐coordinated bis(alkynyl)borane uranocene complex. **VI**: First uranaborabicyclic compound.

Tricoordinate organoboranes constitute an important class of boron compounds. Because of the presence of an empty p orbital on boron, they can act as Lewis acids and electron acceptors in chemical reactions or photophysical processes. In addition, the facile cleavage of B─C bonds enables further derivatization at the carbon atom. With these remarkable features, tricoordinate organoboranes find widespread use in inert bond activation, catalysis, sensing, optoelectronic materials, and organic synthesis.^[^ ^1^, ^2^, ^3^, ^4^, ^5^, ^6^ ^]^ In addition, organoboranes can also serve as ligands, which opens new avenues for modulating the electronic environment at the coordinated metal center.^[^ ^7^, ^8^ ^]^ For instance, as Lewis acids, organoboranes can form TM→B dative bonds with Lewis basic transition metals.^[^ ^9^, ^10^, ^11^, ^12^ ^]^ First structural confirmation of such a compound was done by the group of Hill in 1999, where ruthenium complexes were treated with a bis(azolyl)borate chelating ligand to form a boratrane complex **I**.^[^ ^13^ ^]^ In addition, alkenylboranes and alkynylboranes can serve as acyclic boron‐containing *π*‐ligands, as exemplified by compounds **II** and **III** (Figure 1).^[^ ^14^, ^15^, ^16^ ^]^ **II** represents an *η* ^3^‐vinylborane complex with a borataallyl‐like coordination mode,^[^ ^14^, ^15^ ^]^ while **III** represents an *η* ^3^‐alkynylborane complex with a strong nickel boron dative interaction.^[^ ^16^ ^]^

In 2019, our group reported a ZrC_4_B‐cycle **IV** (Figure 1), in which a bis(alkynyl)borane ligand coordinates to Zr in a unique *η^5^*‐manner.^[^ ^17^ ^]^ Complex **IV** was obtained through the reaction of bis(alkynyl)boranes with Rosenthal's reagent [Cp_2_Zr(Py)(Me_3_SiCCSiMe_3_)],^[^ ^18^, ^19^, ^20^ ^]^ a powerful Zr(II)‐synthon.^[^ ^17^ ^]^ Unlike Rosenthal's reagent‐mediated alkyne reductive coupling, in which two electrons from Zr(II) are typically used to form a C─C *σ*‐bond between two alkynes,^[^ ^18^ ^]^ in this case, the two electrons from Zr(II) were instead delocalized across the C–B–C moiety due to the presence of a bridging electron deficient borane moiety. In 2020, the *η^5^*‐coordination mode was further extended to *f*‐block metals, leading to the successful synthesis and characterization of the first *η^5^*‐C₂BC₂‐coordinated uranocene complex **V** (Figure 1).^[^ ^21^ ^]^ Moreover, it was revealed that the boron substituent affects their stability: the strong reducing power of U(II) and the π‐electron donating amino substituent on boron induced further rearrangement of the bis(alkynyl)borane ligand within the uranium coordination sphere, forming uranium‐fused boracyclobutenes **VI** (Figure 1). Previously, this type of zigzag‐diene‐supported hetero‐dinuclear structural motif was only known for M/Si (M = Ti or Zr).^[^ ^22^, ^23^, ^24^ ^]^ Building on this discovery, the first examples of BN‐butafulvenes were synthesized through the further hydrolysis of **VI**, highlighting the potential of bis(alkynyl)boranes in the construction of novel boracycles.^[^ ^25^ ^]^

Although Ti, Zr, and U share some similarities, such as the relative stability of their +4 oxidation state, the ionic radius of Ti(IV) is significantly smaller than those of Zr(IV) and U(IV) (Ti^IV^ 74.5 pm, Zr^IV^ 86 pm, and U^IV^ 103 pm).^[^ ^26^ ^]^ The allowed coordination number of U is higher than that of Ti due to its larger ionic radius. Uranium was the only known metal that can rearrange bis(alkynyl)borane into a boracyclobutene structure, with the substituent on boron limited to an amino group. In contrast, titanium offers advantages in terms of low toxicity, earth abundance, and cost‐effectiveness, which uranium cannot match. Therefore, beyond the fundamental research significance of understanding the behavior of the unique bis(alkynyl)borane ligands in the coordination spheres of different metals, achieving the rearrangement of bis(alkynyl)boranes through titanium as a replacement for uranium would represent an important step. Herein, we present our progress in the coordination chemistry of a series of bis(alkynyl)boranes with Ti, as well as the Ti‐mediated rearrangement of bis(alkynyl)boranes.

## Results and Discussions

**Figure 2:**
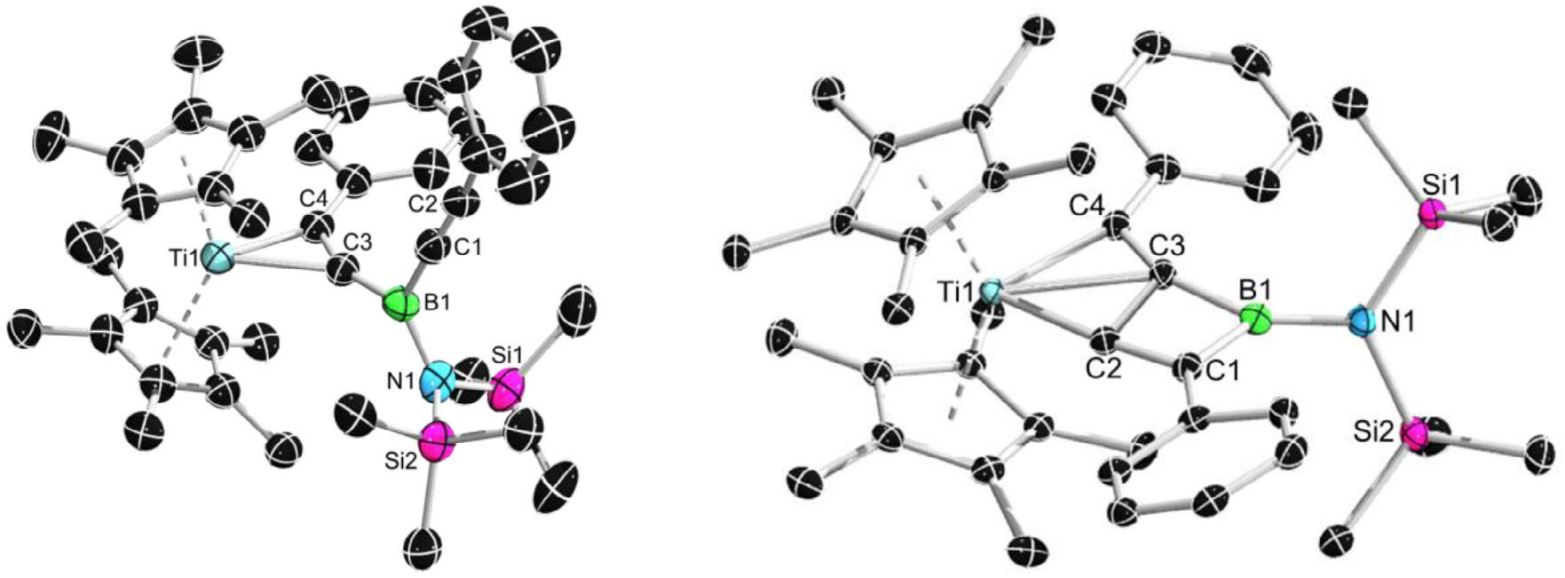
Single crystal structure of complex **3a‐int** (left) and **3a** (right) in the solid state (ellipsoids set at 50% probability). Hydrogen atoms are omitted for clarity. Selected bond lengths [Å], angles and torsions [°] for **3a‐int**: Ti─C3 2.246(3), Ti─C4 2.085(3), B1─N1 1.476(4), B1─C1 1.59(3), B1─C3 1.517(5), C1─C2 1.20(4), C3─C4 1.305(4), B1─C3─C4 156.8(3), C3─C4─Ti1 79.4(2), C4─C2─Ti1 65.8(2), and C1─B1─C3─C4 76(2); for **3a**: Ti─C2 2.011(1), Ti─C3 2.308(1), Ti─C4 2.118(1), B1─N1 1.457(2), B1─C1 1.602(2), B1─C3 1.536(2), C1─C2 1.375(2), C3─C4 1.326(2), C2─C3 1.622(2), B1─C3─C4 156.0(1), C1─C2─Ti1 174.5(1), and C1─C2─C3─C4 177.6(1).

**Scheme 1:**
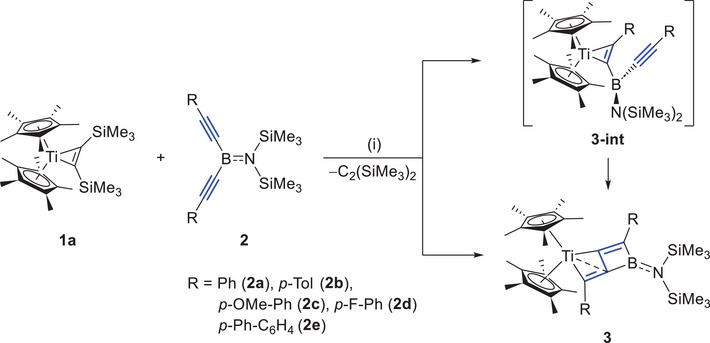
Synthesis of **3a–e**. (i) 1.0 equiv **2**, C_6_D_6_, 80 °C, and 24 h. Isolated yield: **3a**, 85%; **3b**, 62%; **3c**, 73%; **3d**, 77%; and **3e**, 75%.

The reactions of bis(alkynyl)boranes **2a‐e** with decamethyltitanocene **1** were performed in C_6_D_6_ and monitored by ^1^H‐ and ^11^B‐NMR spectroscopy. In contrast to the previously reported reactions with [Cp*_2_U(Me_3_SiCCSiMe_3_)]^[^ ^21^ ^]^ (Cp* = *η^5^*‐C_5_Me_5_) or [Cp_2_Zr(Py)(Me_3_SiCCSiMe_3_)]^[^ ^17^ ^]^ (Py = pyridine), elevated temperatures are required as no reaction occurs at room temperature. Taking the reaction of **1** with **2a** as an example, at 50 °C, multinuclear as well as 2D‐NMR monitoring displayed two sets of new signals. One set of signals (i.e., *δ*_H_ 1.93, 0.34) was converted into the other set (i.e., *δ*_H_ 1.71, 0.44) upon prolonged heating or further elevated temperatures (80 °C), indicating that the reaction proceeds via an intermediate to form the final product. In order to identify the intermediate, the reaction mixture was heated at 50 °C for 16 h, resulting in an intermediate/product ratio of 3:1. The mixture was then stored at −30 °C as a saturated hexane solution for crystallization. A mixture of single crystals of the intermediate and final product were obtained. Through X‐ray diffraction analysis, the intermediate was identified as an *η* ^2^‐complex **3a‐int** (Figure 2, left),^[^ ^27^ ^]^ and the product was determined to be a dinuclear zigzag‐butadiene complex **3a** (Figure 2, right). Targeted synthesis of **3a** could be achieved under conditions of 80 °C for 24 h, with **3a** being isolated in 85% yield as orange red solids. The NMR monitoring results of the reactions of all other bis(alkynyl)boranes **2b–e** with **1** showed an identical reaction profile. In all cases, the dinuclear zigzag‐butadiene product was isolated in decent yields (Scheme 1).

Compound **3a** crystallizes in the monoclinic space group *P2_1_/n*. The fused borete unit is nearly planar (C1–C2–C3–C4 torsion angle: 177.6(1)°). The Ti1–C2 distance (2.011(1) Å) is about 10 pm shorter than Ti1–C4 (2.118(1) Å). It should be noted that a similar T‐shaped geometry with shorter distances to metal has also been observed in our previously reported uranium analogues.^[^ ^21^ ^]^ The C1─C2 and C3─C4 bond lengths (1.375(2), 1.326(2) Å) are within the range of C═C bonds while the C2─C3 bond length (1.622(2) Å) is notably longer than the average value of a C─C single bond (≈1.54 Å). The tricoordinate boron center adopts a trigonal planar geometry as indicated by the sum of the angles around boron (359.5°). Similar to the case of Ti1, the distance from B1 to T‐shaped carbon C3 is about 7 pm shorter than that of B1─C1 bond (B1─C3 1.536(2), B1─C1 1.602(2) Å). Suitable single crystals of compounds **3b,d,e** could also be obtained. In all cases, the Ti1─C2 (**3b**: 1.93(1), **3d**: 1.910(7), and **3e**: 1.93(1) Å) are significantly shorter than the Ti1─C4 (**3b**: 2.17(2), **3d**: 2.188(8), and **3e**: 2.19(1) Å) bond lengths. The B1─C1 (1.54(1)─1.55(1) Å) and B1─C3 (1.58(2)─1.62(1) Å) as well as the B1─N1 (1.46(5)─1.506(8) Å) bond lengths of **3b,d,e** are all in good agreement with the structural data of **3a**. The same applies to the planarity of the central borete unit as indicated by the C1–C2–C3–C4 torsion angles of **3b,d,e** (178(1)–180(1)°).

**Scheme 2:**
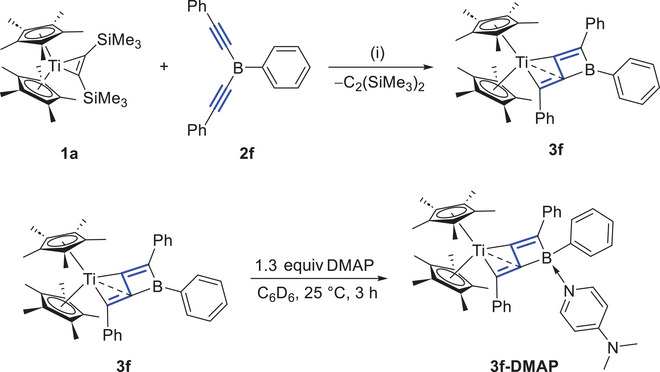
Synthesis of **3f** and **3f‐DMAP**. (i) 1.3 equiv **2f**, C_6_D_6_, 25 °C, and 12 h. Isolated yield: **3f**, 72%; **3f‐DMAP**, 95%.

Our previous studies have demonstrated that the coordination mode of bis(alkynyl)boranes is closely related to the type of substituents on boron. For instance, when coordinating with Cp*_2_U, replacing the R group in **2** from N(SiMe_3_)_2_ to a phenyl group causes the bis(alkynyl)borane ligand to adopt an *η* ^5^‐coordination mode, forming a UC_4_B ring. Therefore, we decided to investigate whether a similar shift in coordination mode would occur with Cp*_2_Ti. To this end, the *B*‐phenyl substituted bis(alkynyl) borane **2f** was added to 1.3 equivs of **1a** in C_6_D_6_ at ambient temperature (Scheme 2, top). The reaction was monitored by ^1^H‐, ^11^B‐, and ^13^C‐NMR spectroscopy. The ^1^H‐NMR spectra indicated the dissociation of bis(trimethylsilyl)acetylene and an almost quantitative conversion of **1a** and **2f** into the product displaying a singlet at *δ*_H_ 1.65 for Cp*. The ^11^B‐NMR signal was slightly upfield‐shifted from *δ*_B_ 46.3 to *δ*_B_ 44.0. By ^13^C‐ and HMBC (heteronuclear multiple bond correlation) 2D‐NMR spectroscopy, it could be observed that the product displays two characteristic quaternary carbon signals (*δ*_C_ 169.1, 191.1), which are very similar to those of C1 and C4 in **3** (e.g., **3a** *δ*_C_ = 164.0, 194.4). Therefore, compound **3f** should be formed, which represents the first *B*‐aryl substituted zigzag‐butadiene Ti/B‐hetero‐dinuclear structure.

**Figure 3:**
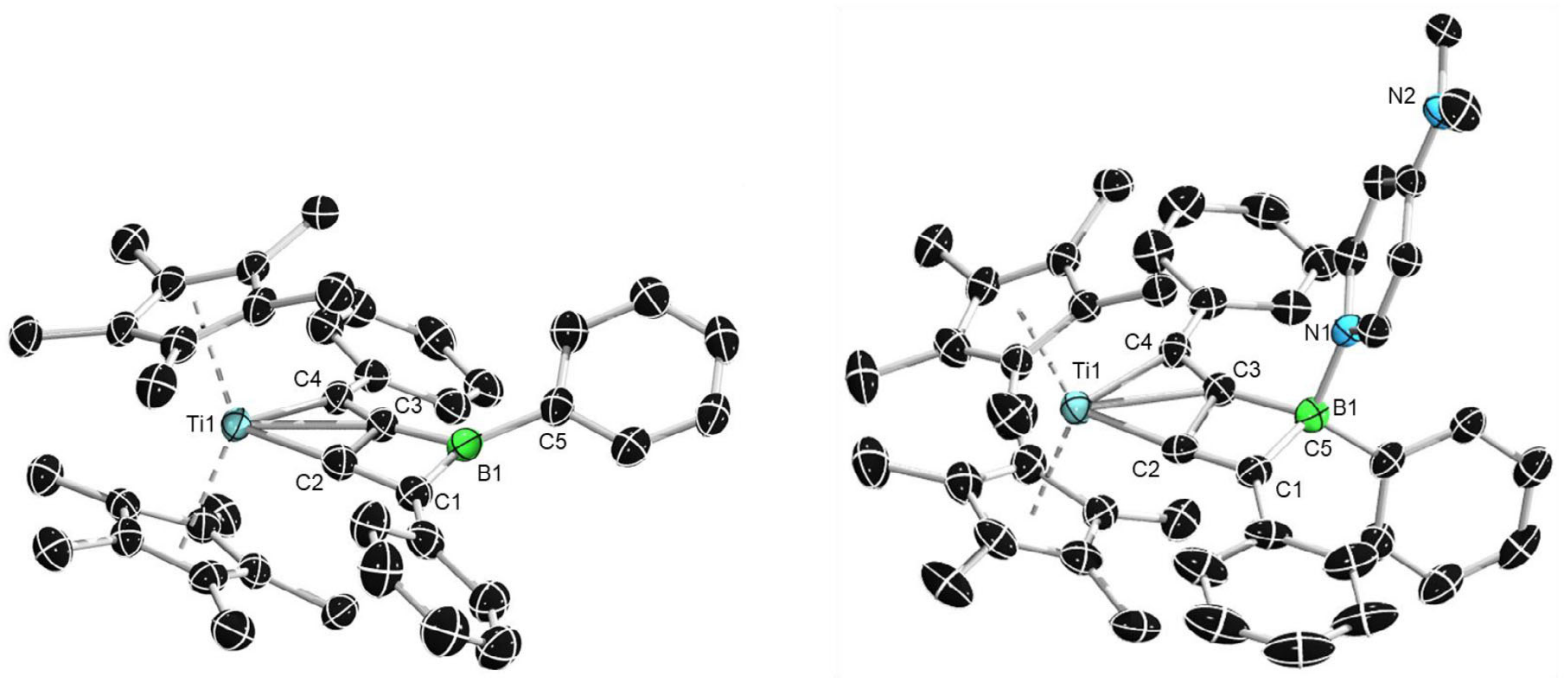
Single crystal structure of complex **3f** (left) and **3f‐DMAP** (right) in the solid state (ellipsoids set at 50% probability). Hydrogen atoms are omitted for clarity. Selected bond lengths [Å], angles and torsions [°] for **3f**: Ti1─C2 2.0197(18), Ti1─C3 2.2834(17), Ti1─C4 2.2834(17), B1─C5 1.564(3), B1─C1 1.528(3), B1─C3 1.567(3), C1─C2 1.370(2), C3─C4 1.325(2), C2─C3 1.620(2), B1─C3─C4 156.02(17), C1─C2─Ti1 170.91(14), and C1─C2─C3─C4 169.01(17). For **3f‐DMAP**: Ti1─C2 2.022(2), Ti1─C3 2.286(2), Ti1─C4 2.099(2), B1─C1 1.607(3), B1─C3 1.683(3), B1─N1 1.606(3), C1─C2 1.351(3), C3─C4 1.321(3), C2─C3 1.593(3), B1─C3─C4 152.21(19), C1─C2─Ti1 174.93(17), and C1─C2─C3─C4 177.7(2).

Red single crystals of **3f**, suitable for X‐ray diffraction analysis, were obtained by fractional crystallization from a saturated *n*‐hexane solution at −30 °C. The molecular structure of **3f** is shown in Figure 3 (left). Compound **3f** crystallized in the triclinic space group *P* $\bar{1}$. The Ti1─C2 bond (2.0197(18) Å) is approximately 26 pm shorter than the Ti1─C4 bond (2.2834(17) Å), a difference significantly larger than that (10 pm) observed in **3a**. The C1─C2 and C3─C4 bond lengths (1.370(1) and 1.325(2) Å, respectively) fell within the range expected for C═C double bonds, while the C2─C3 bond length (1.620 Å) is longer than the average C─C single bond (≈1.54 Å). The three‐coordinate boron center exhibited a trigonal planar geometry, as indicated by the sum of the angles around boron (358.9°). The fused borete unit in **3f** displays a C1–C2–C3–C4 torsion angle of 169.01(17)°), which differs from that observed in **3a–e** (178(1)–180(1)°), indicating a slight distortion of the dinuclear zigzag‐butadiene structure.

When 1.3 equiv of 4‐dimethylaminopyridine (DMAP) were added to a C_6_D_6_ solution of **3f** and reacted at room temperature for 3 h (Scheme 2, below), the formation of a new boron‐containing species was confirmed by ^11^B‐NMR spectroscopy, showing an upfield shifted resonance compared to the C_6_D_6_ solution of **3f** (**3f**: *δ*_B_ 43.3, **3f‐DMAP**: *δ*_B_ −7.9). After workup, an orange solid was obtained. Single crystals of **3f‐DMAP** suitable for X‐ray diffraction analysis were obtained by storing a saturated toluene/*n*‐hexane 1:3 solution at −30 °C for 3 days (Figure 3, right). As indicated by the C1–C2–C3–C4 torsion angle of 177.7(2)°, the dinuclear zigzag‐butadiene skeleton turns planar upon quaternization on the fused borete unit. It should be mentioned that while DMAP merely forms an adduct with **3f** at room temperature, it readily inserts into the amino‐substituted borete unit in **VI** under the same reaction conditions.^[^ ^25^ ^]^ This difference is likely due to the stronger Lewis acidity of the boron center in **3f** compared to that in **VI**, making it more prone to stopping at the adduct formation step.

To further investigate the electronic and steric effect of *B*‐substituents on coordination mode, two novel *B*‐amino bis(alkynyl)boranes **2** **g** and **2h** were synthesized. The detailed synthetic procedure is available in the Supporting Information. In **2** **g**, originating from **2a**, one of the SiMe_3_ groups was replaced with a phenyl group, while in **2** **h**, the two SiMe_3_ groups were replaced with a phenyl and a ─BMes_2_ (Mes = mesityl) group, respectively. It should be noted that, despite the change in the substituents on the nitrogen, the ^11^B‐NMR signals of **2** are nearly identical to that of **2a** (**2a**: *δ*_B_ 31.3, **2g**: *δ*_B_ 28.5, and **2h**: *δ*_B_ 32.5), suggesting that in solution the electronic environment of the bis(alkynyl) boron center is not significantly affected by changes in substituents on the amino group.

**Figure 4:**
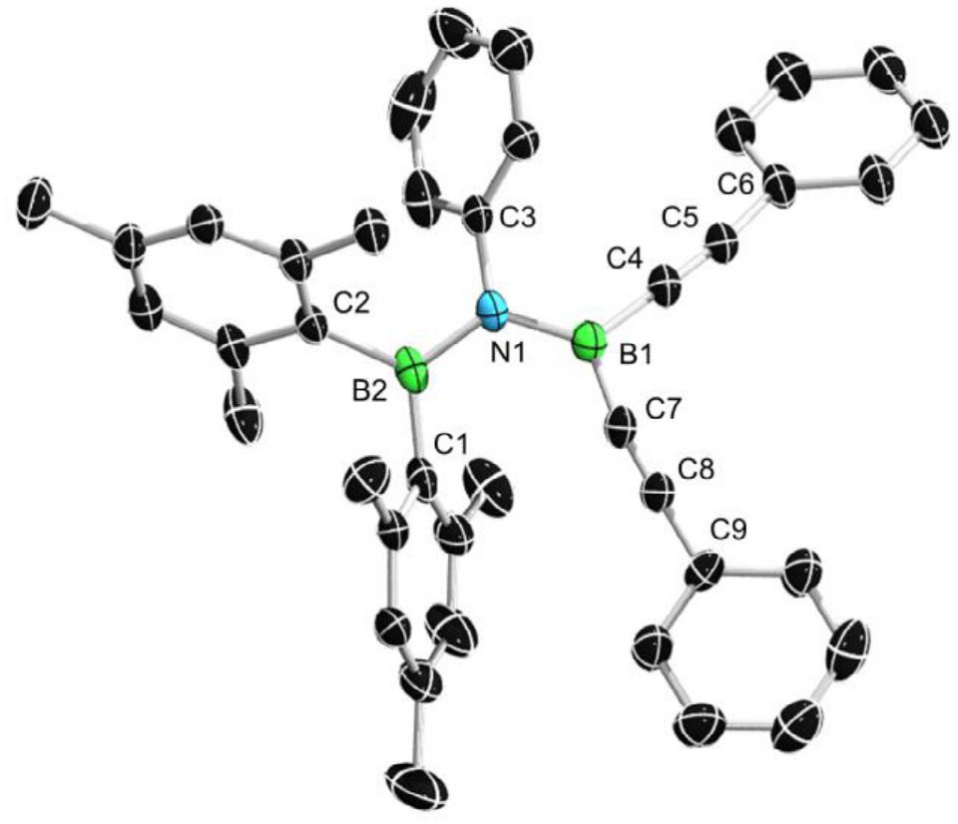
Single crystal structure of **2** **h**. Thermal ellipsoids are drawn at the 50% probability level. Some ellipsoids and hydrogen atoms have been removed for clarity. Selected bond lengths [Å] and angles [°]: N1─B1 1.438(6), N1─B2 1.452(6), C4─C5 1.202(6), C7─C8 1.209(7), C4─B1─C7 117.5(4), C4─B1─N1 119.3(4), C7─B1─N1 123.2(4), C3─N1─B1─C7 18.2(5), and C1─N1─B2─C3 18.8(4).

Single crystals of **2** **h** suitable for X‐ray diffraction analysis were obtained upon storing a saturated hexane solution at −30 °C for 3 days. The molecular structure of **2h** is shown in Figure 4. Compound **2** **h** crystallizes in the triclinic space group *I2/a*. The N1−B1 (1.438(6) Å) bond length is similar and slightly longer than the BN bond in the previously reported amino bis(alkynyl)borane **2e** (1.428(4) Å).^[^ ^25^ ^]^ The N1−B2 (1.452(6) Å) bond length is longer than that in phenyl(trimethylsilyl)amine (1.407(2) Å).^[^ ^28^ ^]^ These data demonstrate the delocalization of the electron lone pair on N1 among the B1−N1−B2 skeleton in the solid state.

**Scheme 3:**
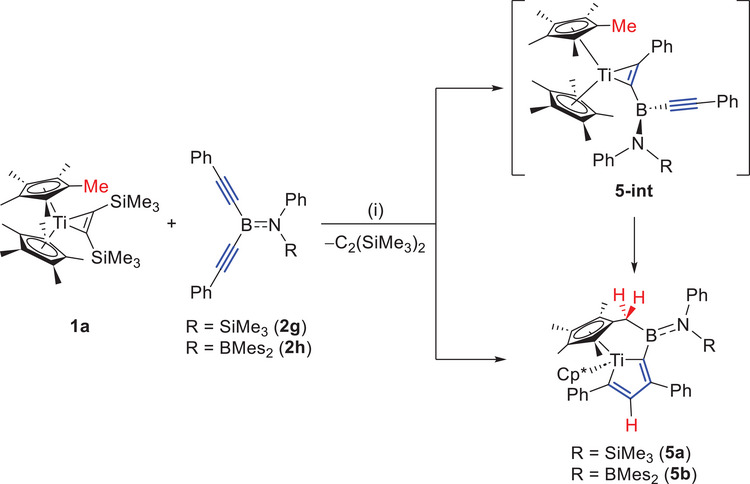
Synthesis of **5a** and **5b**. (i) 1.2 equiv **2**, C_6_D_6_, 70 °C, and 2 d. Isolated yield: **5a**, 61%; **5b**, 65%.

As shown in Scheme 3, the newly obtained bis(alkynyl)boranes **2g,h** were further reacted with **1a**. Similar to the reactions of **2a–e** with **1** mentioned above, the reactions of **2g,h** with **1** did not proceed at room temperature. The reaction of **1a** with **2** **g** was performed in C_6_D_6_ and monitored by ^1^H‐ and ^11^B‐NMR spectroscopy. At 55 °C, the dissociation of bis(trimethylsilyl)acetylene was observed, accompanied by the appearance of new signals corresponding to Cp* (*δ*_H_ 1.65) and ─SiMe_3_ (*δ*_H_ 0.36) in the ^1^H‐NMR spectrum. After two days of heating, the ^11^B‐NMR spectrum indicated the formation of a new boron‐containing species exhibiting a slightly downfield shifted resonance at *δ*_B_ 32.0, which is similar to that observed in the reactions of **2a‐e** with **1** (*δ*_B_ 32–37), suggesting that an *η* ^2^‐complex **5a‐int** was formed. Besides, a tiny amount of an additional new species **5a** was detected in the ^1^H‐NMR spectrum, with the relative ratio of **2** **g**/**5a‐int**/**5a** being approximately 85%/11%/4%. When the temperature was further increased to 70 °C, the ^11^B‐ and ^1^H‐NMR signals of **5a‐int** disappeared, while **2** **g** was gradually converted into **5a**. As the concentration of **5a** increased, a new ^11^B‐NMR signal at 44 ppm was observed. A complete conversion was reached after two days at 70 °C. The final product **5a** displayed a new Cp* (*δ*_H_ 1.67), four methyl groups in the range of 1–2.2 ppm, a methylene group (*δ*_H_ 2.19, 2.90), and an olefinic hydrogen (*δ*_H_ 5.41) signals were observed with an integration ratio of 15:3:3:3:3:2:1, strongly suggesting that one methyl group of one of the two Cp* ligands has undergone C─H activation. The new ─SiMe_3_ signal appeared at 0.19 ppm. The reaction profile of **1a** with **2** **h** was similar. The final boron‐containing product **5b** displayed a ^11^B‐resonance at 54 ppm. A new Cp* (*δ*_H_ 1.81), four methyl groups in the range of 1–3 ppm, a methylene group (*δ*_H_ 1.95, 2.40), and an olefinic hydrogen (*δ*_H_ 6.84) signals were observed, also with an integration ratio of 15:3:3:3:3:2:1.

**Figure 5:**
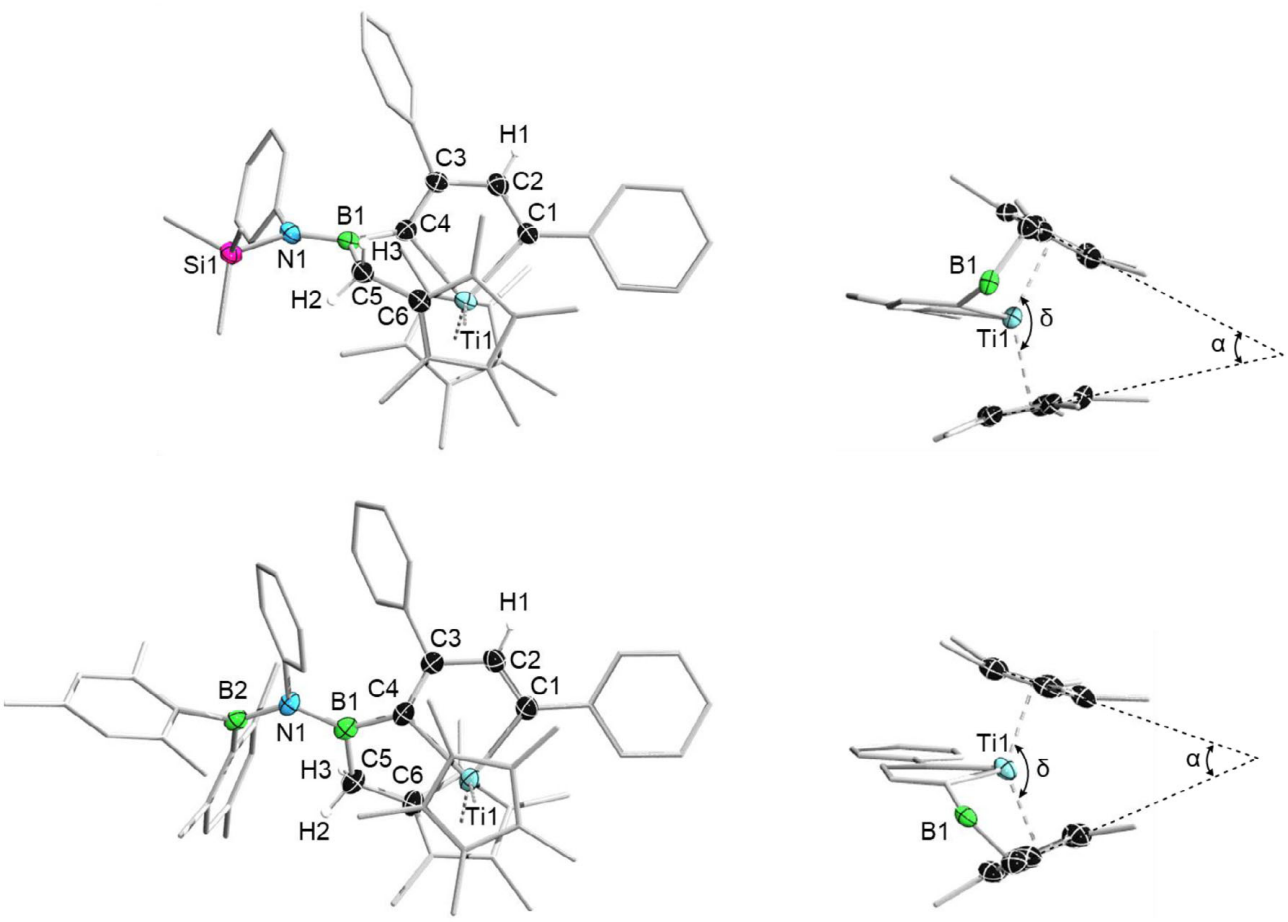
Single crystal structures of **5a** (top left: top view, top right: side view) and **5b** (bottom left: top view, bottom right: side view). Thermal ellipsoids are drawn at the 50% probability level. For top view, some ellipsoids and all the hydrogen atoms have been removed for clarity, except for H1, H2, and H3. For side view, some ellipsoids, all the hydrogen atoms and the substituents on C3 and B1 have been removed for clarity. Selected bond lengths [Å] and angles [°] for **5a**: Ti1─C1 2.173(5), C1─C2 1.357(7), C2─C3 1.465(7), C3─C4 1.371(7), C4─Ti1 2.181(5), B1─C5 1.626(7), B1─N1 1.433(7), B1─C5─C6 108.2(4), Ti1─C1─C2─C3 10.0(6), C1─C2─C3─C4 8.9(7), and Ti1─C4─C3─C2 1.8(5). Selected bond lengths [Å] and angles [°] for **5b**: Ti1─C1 2.167(2), C1─C2 1.350(3), C2─C3 1.467(3), C3─C4 1.371(3), C4─Ti1 2.2131(19), B1─C5 1.602(3), B1─N1 1.475(3), B2─N1 1.448(3), B1─C5─C6 109.70(17), Ti1─C1─C2─C3 12.6(2), C1─C2─C3─C4 10.1(3), and Ti1─C4─C3─C2 1.1(2). *α* represents the tilt angle [°] of the Cp* rings (**5a**: 39.4, **5b**: 40.5). *δ* represents the deformation angle [°] at Ti between the midpoints of the Cp* rings (**5a**: 141.2, **5b**: 140.0).

After workup, brown crystals of **5a** and **5b** (Figure 5) suitable for X‐ray diffraction analysis were obtained. Compound **5a** crystallizes in the triclinic space group *P* $\bar{1}$, while **5b** crystallizes in the monoclinic space group *P2_1_/n*. In sharp contrast to **3**, in which the two alkynyl groups were rearranged to form the zigzag‐butadiene structure, the two alkynyl groups of **2** **g** and **2** **h** have been rearranged to form a titanacyclopentadiene structure, with one methyl group of the Cp* ligand being borylated via C─H activation, affording **5a** and **5b**, respectively (Figure 5). The tilt angles α of the Cp* groups are 39.4° for **5a** and 40.5° for **5b**, while the deformation angles *δ* at Ti between the midpoints of the Cp* rings are 141.2° for **5a** and 140.0° for **5b**.^[^ ^29^ ^]^ Comparing with the nonbridged titania‐cyclopentadiene complexes, such as 1,1‐bis(*η* ^5^‐cyclopentadienyl)‐2,3,4,5‐tetraphenyl‐titana‐cyclopentadiene (*α* = 46.1°, *δ* = 137.8°)^[^ ^30^ ^]^ and 1,1‐bis(*η* ^5^‐cyclopentadienyl)‐3,4‐dimethyl‐2,5‐bis(trimethylsilyl)‐titana‐cyclopentadiene (*α* = 44.5°, *δ* = 137.0°),^[^ ^31^ ^]^ it was observed that the tilt angle in **5a** and **5b** decreases by about 5°, while the deformation angle increases by about 3°.

**Scheme 4:**
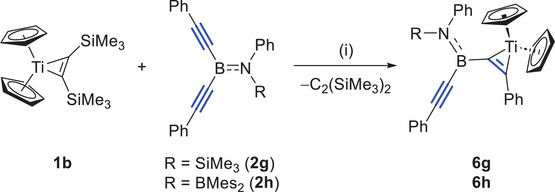
Synthesis of **6** **g** and **6** **h**. (i) 1.0 equiv **2**, C_6_D_6_, 25 °C, and 2 h.

**Figure 6:**
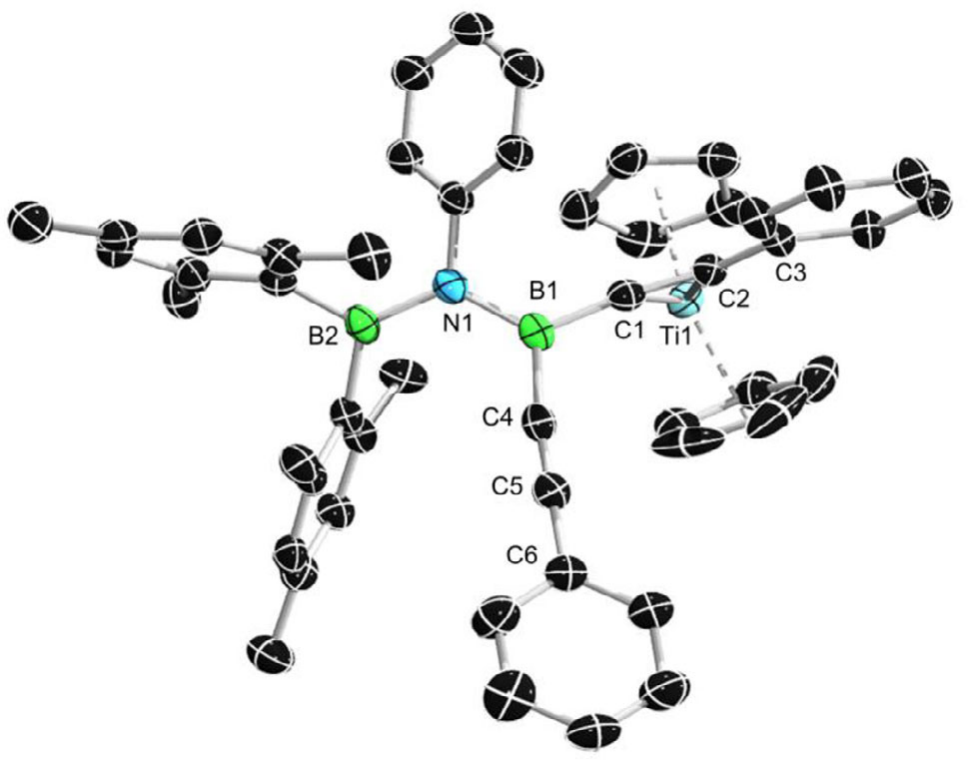
Single crystal structures of **6** **h**. Thermal ellipsoids are drawn at the 50% probability level. Some ellipsoids and hydrogen atoms have been removed for clarity. Selected bond lengths [Å] and angles [°] for **6h**: Ti1─C1 2.2044(16), Ti1─C2 2.0815(16), C1─C2 1.288(2), C4─C5 1.211(2), N1─B1 1.463(2), N1─B2 1.442(2), C1─Ti1─C2 34.82(6), B1─C1─C2 165.38(16), C1─C2─C3 138.62(16), C4─B1─C1─Ti1 87.36(17), and B1─C1─C2─C3 33.1(8).

To further confirm the formation of an *η* ^2^‐intermediate in the first step, we decided to use (C_5_H_5_)_2_Ti[*η* ^2^‐1,2‐C_2_(SiMe_3_)_2_] **1b** as a source of titanacyclopropene complexes to react with **2**. This approach should avoid C─H activation, and thus allowing the reaction to remain at the *η* ^2^‐intermediate stage (Scheme 4). To this end, the bis(alkynyl)aminoborane **2g,h** was added to an equimolar amount of **1b** in C_6_D_6_ at ambient temperature. The reaction was monitored by ^1^H‐ and ^11^B‐NMR spectroscopy. The ^1^H‐NMR spectra indicated the release of bis(trimethylsilyl)acetylene and the almost quantitative conversion of **2** **g,h** and **1b** into products displaying a new signal for the cyclopentadiene (**6g**: *δ*_H_ = 6.22, **6h**: *δ*_H_ = 6.30). The ^11^B‐NMR signals of **6** **g** (32.9 ppm) and **6** **h** (*δ*_B_ = 32.4) are located in the similar region. By ^13^C‐ and HMBC (heteronuclear multiple bond correlation) 2D‐NMR spectroscopy, it could be observed that the product displays one characteristic quaternary carbon signal (**6g**: *δ*_C_ 130.3, **6h**: *δ*_C_ 130.5). Orange crystals of **6** **h** (Figure 6) suitable for X‐ray diffraction analysis were obtained by toluene/pentane vapor diffusion at −30 °C for 2 days. Compound **6** **h** crystallizes in the monoclinic space group *P2_1_/n*. The C1─C2 bond length (1.288(2) Å) represents a significant increase in comparison to the C≡C bond length at the corresponding position in **2** **h** (1.202(6) Å) and is consistent with the C─C bond length in compounds with titana‐cyclopropene structures (1.283(6) Å).^[^ ^32^ ^]^ The Ti1−C1 and Ti1−C2 bond lengths (2.2044(16), 2.0815(16) Å) are consistent with the range for Ti─C bonds. The above data demonstrate the formation of an *η* ^2^‐coordination complex between [Cp₂Ti] and **2** **h**. And based on the NMR similarities, it is believed that the reaction of **1b** with **2** **g** yielded an analogous *η* ^2^‐complex, **6** **g**.

**Figure 7:**
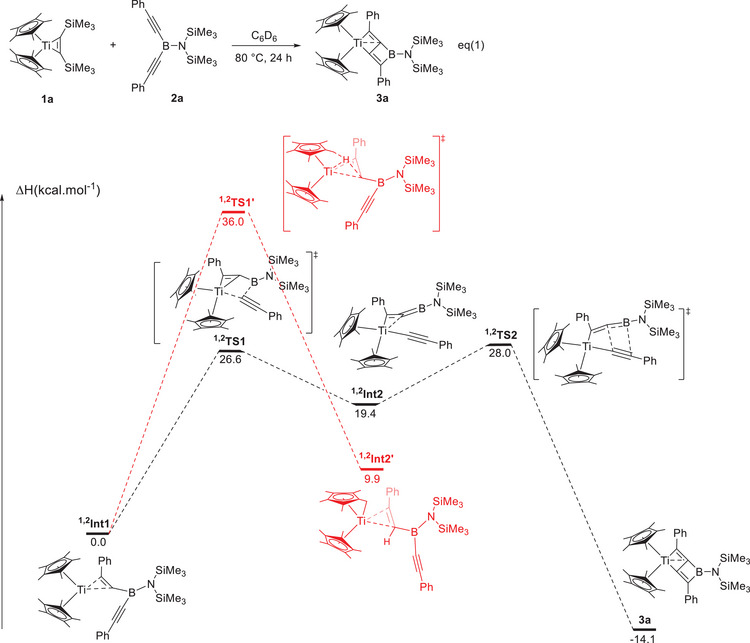
Computed enthalpy profile (energy in kcal.mol^−1^) at room temperature for the formation of **3a** from the reaction of **1a** with **2a**.

Based on the above results, the first step in the formation of **3** as well as **5** is always the generation of an *η* ^2^‐intermediate. This is entirely different from the *η* ^5^‐intermediate mechanism that was previously observed with uranocene.^[^ ^21^ ^]^ To gain insights into the formation of complexes **3a** and **5a** respectively, DFT calculations (B3PW91 functional) were carried out. The computational method is the same as the one used for uranocene so that it is possible to make direct comparisons. The formation of complex **3a** (Figure 7) from the reaction of **1a** with **2a** begins by the coordination of molecule **2a** to the titanium center and release of the bis(trimethylsilyl)acetylene. This step is exothermic and for sake of simplicity our profiles start from the adduct of **2a** to **1a** (labelled **^1,2^Int1** in Figure 7). The facile replacement is easily explained by the fact that in **^1,2^Int1** there is an additional interaction between the second alkyne group and the titanium (IV) center. This secondary bonding interaction is crucial since it slightly activates the B─C bond. Therefore, a B─C bond breaking reaction can occur via **^1,2^TS1** with an accessible energy barrier (26.6 kcal.mol^−1^). Following the intrinsic reaction coordinate it yields the rather unstable alkyl‐alkynyl titanium(IV) intermediate (**^1,2^Int2**, +19.4 kcal.mol^−1^). This unstable intermediate is prompt to react and especially because of the presence of C═B double bond in the alkyl ligand. Indeed, this double bond can undergo a [2+2] cycloaddition with the C─C triple bond of the alkynyl. This cycloaddition occurs with a low barrier (8.6 kcal.mol^−1^) from **^1,2^Int2**, but moderate (28.0 kcal.mol^−1^) from **^1,2^Int1**, explaining the temperature needed to perform the reaction. Following the intrinsic reaction coordinate, it yields complex **3a** whose formation is thermodynamically favorable (−14.1 kcal.mol^−1^ with respect to the entrance channel). The bonding in **3a** was investigated using natural bonding orbitals (NBO) methods (see Supporting Information). It is interesting to note the presence of two Ti─C bonds (Ti─C2 and Ti─C4) polarized toward C (75%) with a Wiberg Bond index (WBI) of 0.77 and the delocalization of the C3─C4 double bond toward the metal as can be seen in the frontier orbitals (see Table S26 in Supporting Information) and explaining the Ti─C4 WBI of 0.19.

**Figure 8:**
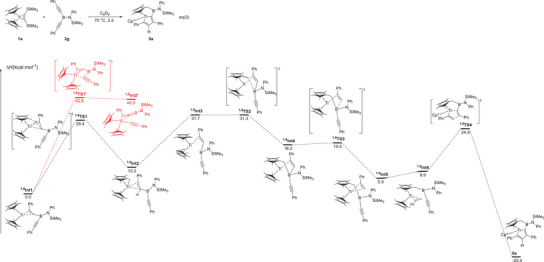
Computed enthalpy profile (energy in kcal.mol^−1^) at room temperature for the formation of **5a** from the reaction of **1a** with **2** **g**.

The formation of complex **5a** from the reaction of **1a** with **2** **g** was thus investigated computationally using the same computational approach (Figure 8). As in the case of the formation of **3a**, the reaction pathway starts from the adduct of **2** **g** to **1a** with the release of one molecule of bis(trimethylsilyl)acetylene (labelled **^1,6^Int1** in Figure 8), which is thermodynamically favorable for the same reason as **^1,2^Int1** in Figure 7. Because of the coordination of the second alkyne group and the titanium center, the transition state associated with the C─B bond breaking (as found in the formation of **3a**) was computed. This TS was located (**^1,6^TS1’**), but the associated barrier is kinetically inaccessible (42.6 kcal.mol^−1^), ruling out this reaction. This can be explained by the presence of the trimethylsilyl substituent that stabilizes a cation at the *β* position, that is at the boron position, while the B─C bond breaking implies primarily relocalizing a negative charge at the alkynyl carbon and a positive charge at the boron center. Therefore, instead, **^1,6^Int1** can transfer a proton from the methyl substituent of the Cp* ring, via **^1,6^TS1**, to the C2 carbon of the metallacyclopropene. The associated barrier is 28.4 kcal.mol^−1^, indicating a kinetically possible reaction. It is interesting to note that a similar TS (**^1,2^TS1’**) was located for the reaction of **1a** with **2a** (Figure 7), but the associated barrier, although accessible (36.0 kcal.mol^−1^) is higher than the B─C bond breaking. After the proton transfer, it yields an unstable intermediate (**^1,6^Int2**), which is a “tuck‐in” complex of titanium(IV)‐alkenyl. The presence of the trigonal planar boron atom in the alkenyl chain with its low‐lying 2*p* empty orbital allows **^1,6^Int2** to evolve by undergoing an electrophilic attack to the CH_2_ group of the tuck‐in ligand. The system reaches **^1,6^TS2** with an associated barrier of 21.1 kcal.mol^−1^ from **^1,6^Int2** (31.2 kcal.mol^−1^ from the entrance). Following the reaction coordinate, it yields the unstable intermediate **^1,6^Int4**, which is bearing a tetra‐coordinated boron center, which is bonded to one Cp* ring via the CH_2_ group and to the titanium center by the alkenyl group (C(Ph)−CH). The rain strain in this 4‐coordinated boron induces an easy B─C(H) bond breaking (**^1,6^TS3**, with no barrier). This allows to form a 3‐coordinated boron and a phenylmetalacyclopropene (**^1,6^Int5)**, which is merely stable (5.9 kcal.mol^−1^ from the entrance channel). Finally, as was already observed in the last step of the formation of **3a**, the presence of a *π* system on the metalcyclopropene and one on the pendant C─C triple bond at the boron center favors a [2+2] cycloaddition reaction. The associated TS was located (**^1,6^TS4**) and the barrier is kinetically accessible (19.0 kcal.mol^−1^ from **^1,6^Int5**, 24.9 kcal.mol^−1^ from the entrance). Following the intrinsic reaction coordinate, it yields complex **5a**, which is thermodynamically very favorable (−20.4 kcal.mol^−1^).

## Conclusion

In summary, we have reported the synthesis and characterization of the first titanium/boron dinuclear zigzag‐diene complexes as well as the novel tethered metallocene complexes by reacting the Rosenthal's Cp*_2_Ti reagent with various bis(alkynyl)boranes. In contrast to the previously reported bis(alkynyl)borane‐to‐boracyclobutene rearrangement mediated by U(II), here the Ti(II) tolerates phenyl substitution at the boron center. The smaller ionic radius of Ti and the relatively smaller coordination number it allows result in a distinct rearrangement mechanism compared to U. NMR monitoring data suggested that the bis(alkynyl)borane initially interacts with the Ti(II) synthon to form an *η^2^*‐coordinated intermediate, which was further confirmed by single‐crystal characterization and theoretical calculations. The more compact intermediate structure resulting from the smaller ionic radius of Ti allows the coordinated alkynyl group to spatially interact with the Cp* methyl group, making the C─H activation possible. The calculated reaction pathway further explains the formation of the two distinct types of products, and elucidates the influence of boron substituents on the selectivity between B─C and C─H activation. The B─C activation step will relocalize a negative charge at the alkynyl carbon and a positive charge at the boron center. The phenyl group in **2f**, with its +M effect, and the two trimethylsilyl groups in **2a‐e**, with their *β*‐effect, both effectively facilitate the increase of positive charge on boron. As a result, B─C activation is more favored in these cases. In contrast, **2** **g** has one fewer trimethylsilyl group, and **2** **h** has no trimethylsilyl groups at all. Without a sufficient silicon *β*‐effect, the reaction barrier for B─C activation becomes inaccessible. Instead, the C─H activation pathway is favored. Further studies on the coordination and rearrangement chemistry of bis(alkynyl)boranes with metals are currently underway in our laboratory.

## Author Contributions

S.Z., C.M., and A.M. carried out all the experiments. T.R. and L.M. performed the DFT calculations. A.M. performed single crystal X‐ray diffraction analysis. Q.Y. conceived and supervised the project. All authors discussed the results and contributed to the final manuscript.

## Conflict of Interests

The authors declare no conflict of interest.

## Supporting information



Supporting information

Supporting information

## Data Availability

The data that support the findings of this study are available in the Supporting Information of this article.
