# Supplementary material for: Titanium‐Mediated Rearrangement of Bis(alkynyl)boranes: B─C Activation versus C─H Activation
Source: Angew Chem Int Ed Engl. 2025 May 19;64(26):e202504229. doi: 10.1002/anie.202504229 (PMC12184290; doi:10.1002/anie.202504229)
Supplement: Supplementary file 1 — Supporting information [file ANIE-64-e202504229-s002.pdf]

Supporting Information

Titanium-Mediated Rearrangement of Bis(alkynyl)boranes: B–C Activation vs C–H Activation

Chenchang Ma,[a+] Alexander Matler,[a+] Shuai Zhu,[b] Thayalan Rajeshkumar,[c] Laurent Maron,[*c] Qing Ye[*a]

[a] Institute for Inorganic Chemistry and Institute for Sustainable Chemistry & Catalysis with Boron, Julius-Maximilians-Universität Würzburg, 97074 Würzburg Germany

[b] Department of Chemistry, Southern University of Science and Technology, 518055 Shenzhen (P. R. China)

[c] Laboratoire de Physique et Chimie des Nanoobjets, INSA, CNRS, UPS, Université de Toulouse, 31077 Toulouse, France

[+] These authors contributed equally to this work.

*Email: laurent.maron@irsamc.ups-tlse.fr (Laurent Maron), qing.ye@uni-wuerzburg.de (Qing Ye)

Table of Contents

General Information. S2
Experimental procedures S2
NMR Spectroscopy S10
High resolution mass spectroscopy data S34
Crystallographic Details S45
Computational details S59
References S107

General Information.

All manipulations were conducted either under an atmosphere of dry argon or in vacuo using standard Schlenk line or glovebox techniques. Deuterated solvents were dried over molecular sieves and degassed by three freeze-pump-thaw cycles before use. All other solvents were distilled and degassed from appropriate drying agents. Both deuterated and non-deuterated solvents were stored under argon over activated 4 Å molecular sieves. All NMR spectra were obtained from a Bruker Avance 500 NMR spectrometer (^{1}H: 500 MHz, ^{13}C{1H}: 125 MHz, ^{11}B: 160 MHz, ^{29}Si: 100 MHz) or a Bruker Avance **I** 400 NMR spectrometer (^{1}H: 400 MHz, ^{13}C{1H}: 100 MHz, ^{11}B: 128 MHz, ^{29}Si: 80 MHz) at 298 K, unless otherwise stated. Chemical shifts (δ) are reported in ppm and internally referenced to the carbon nuclei (^{13}C{^{1}H}) or residual protons (^{1}H) of the solvent. ^{11}B-NMR spectra were referenced to external BF_3·OEt_2. The broad humps centered at around -10 ppm in ^{11}B-NMR spectra arise from the NMR tubes made of borosilicate glass. **1a**-**b**,[1] bis(alkynyl)boranes **2a,b,e**,[2] Phenyl(trimethylsilyl)amine[3] and, phenyl(dimesitylboryl)amine[4] were prepared according to published procedures. High resolution mass spectrometry (HRMS) was performed with a Thermo Fisher Scientific Q-Exactive MS System.

Experimental procedures

Synthesis of **2c**:

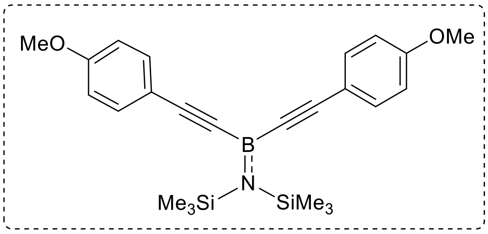

$(Me_3Si)_2NBCl_2$ (220 mg, 909 µmol, 1.0 eq.) was dissolved in hexane (5 ml) and added to a hexane suspension (15 ml) of LiC≡CR (276 mg, 2 mmol, 2.2 eq.) at −35°C. The mixture was stirred at room temperature for 12 h prior to the removal of all volatiles under vacuum. The residue was extracted with hexane, filtered, and the solvent was removed under high vacuum to yield a pale yellow oil (265 mg, 611 µmol, 67%).

^{1}H-NMR (400 MHz, C_6D_6) δ (ppm) = 0.54 (s, 18H, Si*Me*3), 3.17 (s, 6H, OC*H*3), 6.57 – 6.53 (m, 4H, Ar*H*), 7.45 – 7.41 (m, 4H, Ar*H*). **^{11}B{^{1}H} NMR** (128 MHz, C_6D_6) δ (ppm) = 31.2. **^{13}C{^{1}H} NMR** (100 MHz, C_6D_6) δ (ppm) = 4.83, 54.77, 98.65, 109.58, 114.40, 115.97, 133.93, 160.66. **HRMS-ESI** (m/z): Calculated (found) for $C_{24}H_{33}O_2BNSi_2[M+H]^+$ 434.21374 (434.21356).

Synthesis of **2d**:

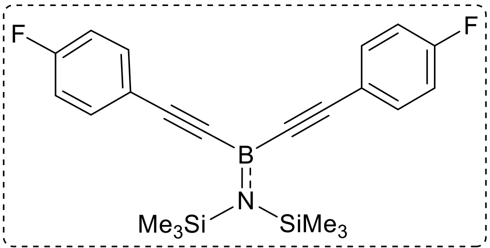

$(Me_3Si)_2NBCl_2$ (201 mg, 831 µmol, 1.0 eq.) was dissolved in pentane (5 ml) and added to a toluene suspension (15 ml) of LiC≡CR (220 mg, 1.75 mmol, 2.1 eq.) at −35°C. The mixture was stirred at room temperature for 12 h prior to the removal of all volatiles under vacuum. The residue

was extracted with hexane, filtered, and the solvent was removed under high vacuum to yield a pale yellow solid (248 mg, 606 µmol, 73%).

^{1}H-NMR (400 MHz, C_6D_6) δ (ppm) = 0.50 (s, 18H, Si*Me*$_3$), 6.55 (m, 4H, Ar*H*), 7.23 (m, 4H, Ar*H*). **^{11}B{^{1}H} NMR** (128 MHz, C_6D_6) δ (ppm) = 31.2. **^{13}C{^{1}H} NMR** (100 MHz, C_6D_6) δ (ppm) = 4.70 (Si*Me*$_3$), 108.3, 115.8, 116.0, 119.77 119.81, 134.1, 134.2, 162,0, 164.5. **HRMS-ESI** (m/z): Calculated (found) for $C_{22}H_{27}BNF_2Si_2$[M+H]$^+$ 410.17078 (410.17377).

Synthesis of **3a**:

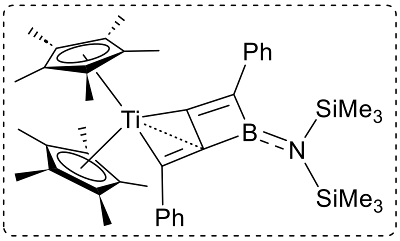

1a (97.8 mg, 200 µmol, 1.0 eq.) and **2a** (74.7 mg, 200 µmol, 1.0 eq.) were dissolved in C_6D_6 and heated at 80 °C for 24 h. After removal of all volatiles, the residue was dissolved in hexane, the precipitating orange solid was washed with hexane (3x1 mL) and extracted with toluene (2x0.5 mL). Slow evaporation of the toluene solution for 3 days at room temperature yielded the analytically pure orange-red product (118 mg, 171 µmol, 85%) as single crystals, which were suitable for X-ray single crystal analysis.

^{1}H-NMR (400 MHz, C_6D_6) δ (ppm) = 0.44 (s, 18H, Si*Me*$_3$), 1.71 (s, 30H, C_5*Me*$_5$), 7.12 (m, 2H, Ph*H*), 7.37 (m, 4H, Ph*H*), 7.78 (m, 2H, Ph*H*), 8.09 (m, 2H, Ph*H*). **^{11}B{^{1}H} NMR** (128 MHz, C_6D_6) δ (ppm) = 45.0. **^{13}C{^{1}H} NMR** (100 MHz, C_6D_6) δ (ppm) = 4.30 (Si*Me*$_3$), 12.6 (C_5*Me*$_5$), 114.9 (C_5*Me*$_5$), 126.2 (Ph), 127.1 (Ph), 127.3 (Ph), 127.9 (Ph), 128.6 (Ph), 132.5 (Ph), 139.76, 139.79, 194.4, 300.2. **HRMS-ESI** (m/z): Calculated (found) for $C_{42}H_{59}NBSi_2Ti$[M+H]$^+$ 692.37665 (692.37531).

Synthesis of **3b**:

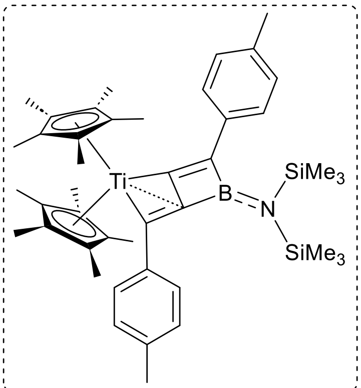

1a (97.8 mg, 200 µmol, 1.0 eq.) and **2b** (80.3 mg, 200 µmol, 1.0 eq.) were dissolved in C_6D_6 and heated at 80 °C for 24 h. After removal of all volatiles, the residue was dissolved in hexane, the precipitating orange solid was washed with hexane (3x1 mL) and extracted with toluene (2x0.5 mL). Slow evaporation of the toluene solution for 3 days at room temperature yielded the analytically pure orange-red product (95.8 mg, 133 µmol, 62%) as single crystals, which were suitable for X-ray single crystal analysis.

^{1}H-NMR (400 MHz, C_6D_6) δ (ppm) = 0.48 (s, 18H, Si*Me*$_3$), 1.75 (s, 30H, C_5*Me*$_5$), 2.21 (s, 3H, C*H*$_3$-Ph), 2.23 (s, 3H, C*H*$_3$-Ph), 7.18 (m, 2H, Ph*H*), 7.21 (m, 2H, Ph*H*), 7.72 (m, 2H, Ph*H*), 8.00 (m, 2H, Ph*H*). **^{11}B{^{1}H} NMR** (128 MHz, C_6D_6) δ (ppm) = 45.9. **^{13}C{^{1}H} NMR** (100 MHz, C_6D_6) δ (ppm) = 4.35 (Si*Me*$_3$), 12.6 (C_5*Me*$_5$), 21.41 (C_6H_4*Me*), 21.44 (C_6H_4*Me*), 114.7, 127.3, 128.6, 129.4, 132.6, 135.5, 136.5, 136.96, 137.01, 193.9, 299,8. **HRMS-ESI** (m/z): Calculated (found) for $C_{44}H_{63}NBSi_2Ti$[M+H]$^+$ 720.40710 (720.40661).

Synthesis of **3c**:

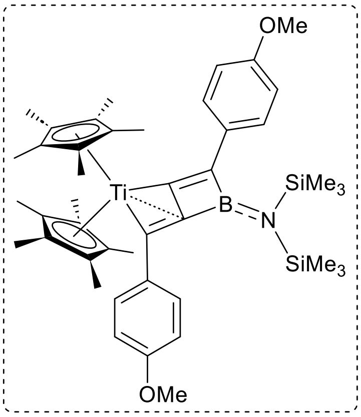

1a (40 mg, 81.8 µmol, 1.0 eq.) and **2c** (35.5 mg, 81.8 µmol, 1.0 eq.) were dissolved in C_6D_6 and heated at 80 °C for 48 h. After removal of all volatiles, the residue was dissolved in hexane, the precipitating orange solid was washed with hexane (3x1 mL) and extracted with toluene (2x0.5 mL). Slow evaporation of the toluene solution for 3 days at room temperature yielded the analytically pure orange-red product (45.1 mg, 60 µmol, 73%) as single crystals, which were suitable for X-ray single crystal analysis.

^{1}H-NMR (400 MHz, C_6D_6) δ (ppm) = 0.47 (s, 18H, Si*Me*$_3$), 1.76 (s, 30H, C_5*Me*$_5$), 3.38 (s, 3H, OC*H*$_3$), 3.41 (s, 3H, OC*H*$_3$), 6.98 – 7.05 (m, 4H, Ar*H*), 7.73 – 7.78 (m, 2H, Ar*H*), 8.02 – 8.07 (m, 2H, Ar*H*). **^{11}B{^{1}H} NMR** (128 MHz, C_6D_6) δ (ppm) = 43.8. **^{13}C{^{1}H} NMR** (100 MHz, C_6D_6) δ (ppm) = 4.35, 12.62, 54.77, 54.85, 113.33, 114 21, 114.61, 128.45, 132.52, 132.67, 133.79, 158.71, 159.31, 163.64, 192.96, 298.50.

Synthesis of **3d**:

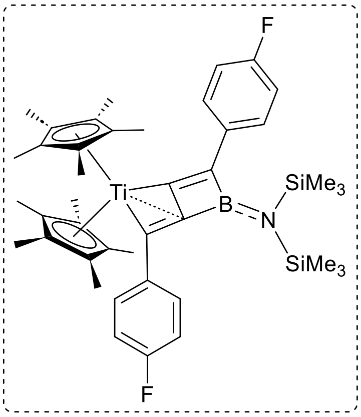

1a (97.8 mg, 200 µmol, 1.0 eq.) and **2d** (81.9 mg, 200 µmol, 1.0 eq.) were dissolved in C_6D_6 and heated at 80 °C for 24 h. After removal of all volatiles, the residue was dissolved in hexane, the precipitating orange solid was washed with hexane (3x1 mL) and extracted with toluene (2x0.5 mL). Slow evaporation of the toluene solution for 3 days at room temperature yielded the analytically pure orange-red product (111.7 mg, 153 µmol, 77%) as single crystals, which were suitable for X-ray single crystal analysis.

^{1}H-NMR (400 MHz, C_6D_6) δ (ppm) = 0.37 (s, 18H, Si*Me*$_3$), 1.66 (s, 30H, C_5*Me*$_5$), 7.01 (m, 4H, Ph*H*), 7.63 (m, 2H, Ph*H*), 7.91 (m, 2H, Ph*H*). **^{11}B{^{1}H} NMR** (128 MHz, C_6D_6) δ (ppm) = 44.6. **^{13}C{^{1}H} NMR** (100 MHz, C_6D_6) δ (ppm) = 4.22 (Si*Me*$_3$), 12.5 (C_5*Me*$_5$), 114.6 (*C*$_5$Me$_5$), 114.8 (*C*$_5$Me$_5$), 114.9 (*C*$_5$Me$_5$), 115.4 (*C*$_5$Me$_5$), 115.6 (*C*$_5$Me$_5$), 133.7, 133.8, 135.8, 135.9, 135.98, 136.02, 160.7, 161.1, 163.2, 163.6, 192.8, 299.0. **HRMS-ESI** (m/z): Calculated (found) for $C_{42}H_{57}NBF_2Si_2Ti[M+H]^+$ 728.35815 (728.35646).

Synthesis of **3e**:

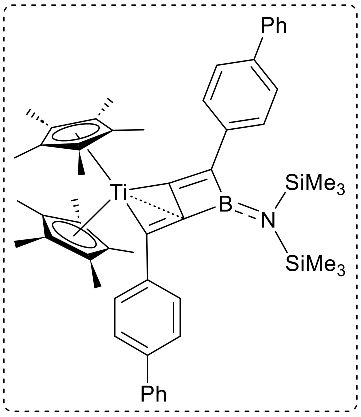

1a (97.8 mg, 200 µmol, 1.0 eq.) and **2e** (105.1 mg, 200 µmol, 1.0 eq.) were dissolved in C_6D_6 and heated at 80 °C for 24 h. After removal of all volatiles, the residue was dissolved in hexane, the precipitating orange solid was washed with hexane (3x1 mL) and extracted with toluene (2x0.5 mL). Slow evaporation of the toluene solution for 3 days at room temperature yielded the analytically pure orange-red product (126.3 mg, 150 µmol, 75%) as single crystals, which were suitable for X-ray single crystal analysis.

^{1}H-NMR (500 MHz, C_6D_6) δ (ppm) = 0.49 (s, 18H, Si*Me*$_3$), 1.77 (s, 30H, C_5*Me*$_5$), 7.15 (m, 2H, Ph*H*), 7.24 (m, 4H, Ph*H*), 7.56 (m, 2H, Ph*H*), 7.64 (m, 2H, Ph*H*), 7.69 (m, 2H, Ph*H*), 7.75 (m, 2H, Ph*H*), 7.89 (m, 2H, Ph*H*), 8.17 (m, 2H, Ph*H*). **^{11}B{^{1}H} NMR** (160 MHz, C_6D_6) δ (ppm) = 47.4. **^{13}C{^{1}H} NMR** (125 MHz, C_6D_6) δ (ppm) = 4.38 (Si*Me*$_3$), 12.6 (C_5*Me*$_5$), 99.7, 115.0, 114.9, 126.6, 127.3, 127.4, 127.7, 129.06, 129.09, 133.1, 138.7, 138.96, 139.00, 140.0, 141.5, 141.6, 164.8, 194.3, 300.6. **HRMS-ESI** (m/z): Calculated (found) for $C_{54}H_{67}NBSi_2Ti[M+H]^+$ 844.43951 (844.43791).

Synthesis of **3f**:

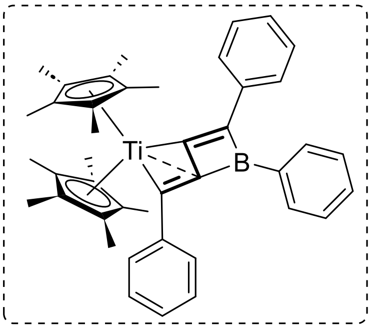

1a (20 mg, 41.0 µmol, 1.0 eq.) and **2f** (15,5 mg, 53.3 µmol, 1.3 eq.) were dissolved in 0.5 mL C_6D_6. After 12 hours at room temperature, all volatiles were removed. The residue was dissolved in hexane, the precipitating orange-red solid was purified by fractional crystallization. The analytically pure red product (15.7 mg, 25.8 µmol, 63%) as single crystals, which were suitable for X-ray single crystal analysis, were obtained from a saturated *n*-hexane solution for 2 days at −30 °C.

^{1}H-NMR (400 MHz, C_6D_6) δ (ppm) = 1.64 (s, 30H, C_5*Me*$_5$), 7.11-7.22 (m, 3H, B-Ph*H*), 7.32 (t, *J* = 7.7 Hz, 3H, Ph*H*), 7.39 (t, 3H, *J* = 7.7 Hz, Ph*H*), 7.70 (dd, 2H, *J* = 8.3 Hz, 1.3 Hz, Ph*H*), 8.07 (dd, 2H, *J* = 8.3 Hz, 1.3 Hz, Ph*H*), 8.31 (dd, 2H, *J* = 7.6 Hz, 1.9 Hz, B-Ph*H*). **^{11}B{^{1}H} NMR** (160 MHz, C_6D_6) δ (ppm) = 43.3. **^{13}C{^{1}H} NMR** (100 MHz, C_6D_6) δ (ppm) = 12.4, 115.0, 124.0, 126.4, 127.6, 128.3, 128.4, 128.9, 131.2, 131.7, 137.2, 139.3, 140.7, 169.1, 191.1, 272.1. **HRMS-LIFDI** (m/z): Calculated (found) for $C_{42}H_{45}BTi$ $[M+H]^+$ 608.3088 (608.3077).

Synthesis of **3f-DMAP**:

DMAP (6.5 mg, 53.2 μmol, 1.3 eq.) was added in the above C_6D_6 solution of **3f**. (24.9 mg, 41.0 μmol, 1.0 eq.). After 3 hours at room temperature the reaction was completed. After removal of all volatiles, the orange residue was washed with hexane (3x1 mL). Pure orange product **3f-DMAP** (28.5 mg, 39.0 μmol, 95%) was obtained by storing a saturated toluene/n-hexane mixed solution (toluene: n-hexane = 1:3) at −30 °C for 3 days, which were suitable for X-ray single crystal analysis.

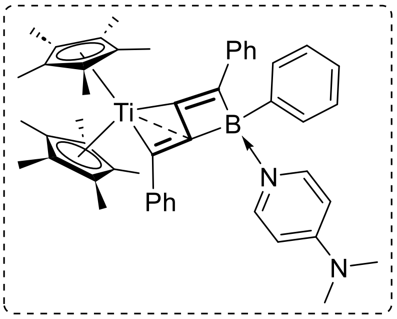

^{1}H-NMR (400 MHz, C_6D_6) δ (ppm) = 1.73 (6H, N*Me*$_2$), 1.85 (15H, C_5*Me*$_5$), 1.92 (15H, C_5*Me*$_5$), 5.31 (d, *J*=7.6, 2H, DMAP-*H*), 7.12 (tt, *J*=7.4, 1.3, 1H, Ph*H*), 7.19 – 77.22 (m, 1H, Ph*H*), 7.25 –.7.30 (m, 1H, Ph*H*), 7.40 – 7.48 (m, 6H, Ph*H*), 7.94 - 7.96 (m, 4H, Ph*H*), 8.35 (dd, *J*=8.3, 1.4, 2H, Ph*H*)), 8.48 (d, *J*=7.5, 2H, DMAP-*H*). **^{11}B{^{1}H} NMR** (160 MHz, C_6D_6) δ (ppm) = –7.9. **^{13}C{^{1}H} NMR** (126 MHz, C_6D_6) δ (ppm) = 12.83, 12.89, 38.20, 105.94, 113.92, 114.77, 124.90, 125.51, 126.17, 127.46, 127.50, 128.56, 128.59, 128.70, 131.74, 132.20, 134.83, 143.30, 143.53, 146.24, 154.50, 193.45, 261.6. **HRMS-LIFDI** (m/z): Calculated (found) for $C_{49}H_{55}B_1Ti_1N_2$ $[M]^+$ 730.3932 (730.3917).

Synthesis of **4a,b** and **2g,h**

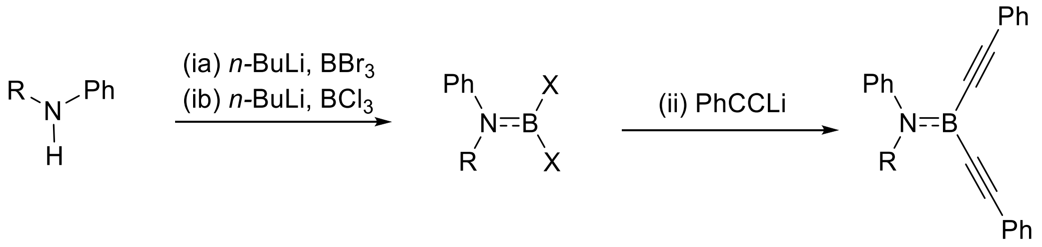

4a: R = $SiMe_3$, X = Br
4b: R = $BMes_2$, X = Cl

2g: R = $SiMe_3$
2h: R = $BMes_2$

4a: Phenyl(trimethylsilyl)amine (6.36 g, 38.5 mmol, 1.0 eq.) was dissolved in 100 mL dry hexane. *n*-BuLi (25.3 mL, 40.4 mmol, 1.05 eq., 1.6 M in hexane) was added dropwise to the above solution at −78 °C. The mixture was stirred at room temperature overnight. The suspension was filtered. The white precipitate was washed with hexane 3 times and dried under vacuum to obtain the lithium salt of phenyl(trimethylsilyl)amine as a white powder (6.32 g, 36.9 mmol, 96%). The white powder was suspended in 100 mL dry toluene. BBr_3 (40.6 mL, 40.6 mmol, 1.10 eq., 1.0 M in heptane) was added to the above suspension dropwise at −78 °C. The mixture was stirred at room temperature overnight. The suspension was filtered. All volatiles were removed under vacuum to afford crude **4a** as a gray oil (9.96 g, 29.8 mmol, 81%). This crude product can be used for further synthesis without purification. Some NMR data of **4a** are as follows: **^{1}H NMR** (400 MHz, C_6D_6) δ (ppm) = 0.11 (s, 9H, Si*Me*$_3$), 6.61-6.64 (m, 2H, Ph*H*), 6.90-

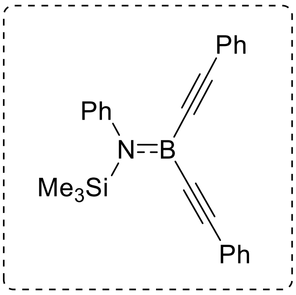

6.94 (m, 1H, Ph*H*), 6.97-7.02 (m, 2H, Ph*H*). **^{11}B{^{1}H} NMR** (128 MHz, C_6D_6) δ (ppm) = 28.9. **^{29}Si NMR** (80 MHz, C_6D_6) δ (ppm) = 17.4.

2g: **4a** (667 mg, 2.0 mmol, 1.0 eq.) was dissolved in 8 mL dry toluene, PhCCLi (430 mg, 4.0 mmol, 2.0 eq.) was suspended in 8 mL dry toluene. The toluene-solution of **4a** was added to the suspension dropwise at −30°C. The mixture was stirred at room temperature for 4 hours. The suspension was filtered. The solvent was removed under vacuum to afford **2g** as an orange oil (519 mg, 1.38 mmol, 69%).

^{1}H NMR(400 MHz, C_6D_6) δ (ppm) = 0.37 (s, 9H, $SiMe_3$), 6.82-6.84 (m, 3H, Ph-*H*), 6.95-6.97 (m, 2H, Ph-*H*), 6.98-7.01 (m, 2H, Ph-*H*), 7.03-7.06 (m, 1H, Ph-*H*), 7.11-7.14 (m, 2H, Ph-*H*), 7.15-7.18 (m, 3H, Ph-*H*), 7.50-7.53 (m, 2H, Ph-*H*). **^{11}B{^{1}H} NMR** (128 MHz, C_6D_6) δ (ppm) = 28.5. **^{13}C{^{1}H} NMR** (100 MHz, C6D6) δ (ppm) = 1.82, 123.75, 125.27, 128.68, 128.76, 128.85, 129.13, 132.19, 132.72, 148.62. **^{29}Si NMR** (80 MHz, C_6D_6) δ (ppm) = 13.64. **HRMS-ASAP** (m/z): Calculated (found) for $C_{25}H_{25}B_1N_1Si_1[M+H]^+$ 378.1844 (378.1842).

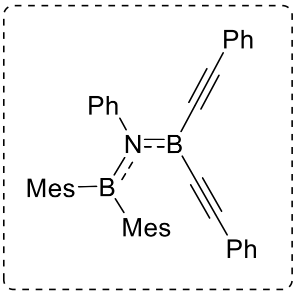

4b: Phenyl(dimesitylboryl)amine (2.73 g, 8 mmol, 1.0 eq.) was dissolved in 25 mL dry toluene. *n*-BuLi (5.5 mL, 8.8 mmol, 1.1 eq., 1.6 M in hexane) was added to the above solution dropwise at −78°C. The mixture was stirred at room temperature overnight. The solvent was removed under vacuum to obtain the lithium salt of phenyl(dimesitylboryl)amine as a pale-yellow solid (2.10 g, 6.05 mmol, 76%). The obtained solid was then suspended in 25 mL dry toluene. BCl_3 (6.2 mL, 6.2 mmol, 1.2 eq., 1.0 M in heptane) was added to the above suspension dropwise at −78°C. The mixture was stirred at room temperature overnight. The suspension was filtered. All volatiles were removed under vacuum to afford pale yellow oil as the crude product, which was crystallized with hexane to get the white solid. The solid was washed with hexane 3 times. All the volatiles were removed again under vacuum to obtain pure **4b** as a white powder (1.45 g, 3.43 mmol, 66%). NMR data of **4b** are as follows: **^{1}H NMR** (400 MHz, C_6D_6) δ (ppm) =1.91 (s, 3H, *p*-CH_3 of Mes), 2.14 (s, 3H, *p*-CH_3 of Mes), 2.24 (s, 6H, *o*-CH_3 of Mes), 2.44 (s, 6H, *o*-CH_3 of Mes), 6.47 (br, s, 2H, Ar-*H*), 6.71-6.76 (m, 3H, Ar-*H*), 6.82-6.86 (m 2H, Ar-*H*), 6.96-6.98 (m, 2H, Ar-*H*). **^{11}B{^{1}H} NMR** (128 MHz, C_6D_6) δ (ppm) =40.8, 57.9. **^{13}C{^{1}H} NMR** (126MHz, C_6D_6) δ (ppm) =21.06, 21.25, 22.83, 23.06, 31.97, 126.35, 128.35, 128.45, 129.29, 146.93. **HRMS-LIFDI** (m/z): Calculated (found) for $C_{24}H_{28}B_1N_1[M]^+$ 341.2309(341.2304).

2h: **4b** (1.18 g, 2.8 mmol, 1.0 eq.) was dissolved in 10 mL dry toluene, PhCCLi (604 mg, 5.6 mmol, 2.0 eq.) was suspended in 10 mL dry toluene. The toluene solution of **4b** was added to the suspension dropwise at −30°C. The mixture was stirred at room temperature for 4 hours. The suspension was filtered. The solvent was removed under vacuum to afford pale yellow oil as the crude product, which was crystallized with hexane to get the white solid. The solid was washed with hexane 3 times. All the volatiles were removed again under vacuum to obtain pure **2h** as a white powder (1.02 g, 1.85 mmol, 66%).

^{1}H NMR (400 MHz, C_6D_6) δ (ppm) =1.95 (s, 3H, *p*-C*H*$_3$ of Mes), 2.10 (s, 3H, *p*-C*H*$_3$ of Mes), 2.37 (s, 6H, *o*-C*H*$_3$ of Mes), 2.59 (s, 6H, *o*-C*H*$_3$ of Mes), 6.53 (s, 2H, Ar-*H* of Mes), 6.74 (s, 2H, Ar-*H* of Mes), 6.86 (s, 3H, Ar-*H*), 6.88 (s, 4H, Ar-*H*), 6.98 (t, *J* = 7.6 Hz, 2H, Ar-*H*), 7.21 (t, *J* = 3.8 Hz, 4H, Ar-*H*), 7.37 (d, *J* = 7.6 Hz, 2H, Ar-*H*). **^{11}B{^{1}H} NMR** (128 MHz, C_6D_6) δ (ppm) = 32.5, 59.5. **^{13}C{^{1}H} NMR** (100 MHz, C_6D_6) δ (ppm) =21.08, 21.22, 23.06, 23.71, 111.90, 123.39, 125.64, 127.74, 128.49, 129.21, 129.26, 133.01, 137.90, 138.93, 139.96, 141.79, 149.19. **HRMS-LIFDI** (m/z): Calculated (found) for $C_{40}H_{37}B_2N_1[M]^+$ 553.3107(553.3109).

Synthesis of **5a**:

2g (75 mg, 0.2 mmol) and **1a** (98 mg, 0.2 mmol) were dissolved in 1 mL C_6D_6. The mixture was heated at 70 °C for 2 days and then all the volatiles were removed to yield a black oil, which crystallized upon storing a concentrated hexane solution at –30 °C overnight. The residue was filtered and washed with cold hexane three times to obtain **5a** as a pale orange solid (83 mg, 0.12 mmol, 61%).

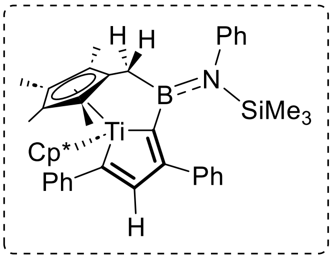

^{1}H NMR (400 MHz, C_6D_6) δ (ppm) = 0.19 (s, 9H, Si*Me*$_3$), 1.20 (s, 3H, C*H*$_3$ of Cp*), 1.67 (s, 15H, C*H*$_3$ of Cp*), 1.71 (s, 3H, C*H*$_3$ of Cp*), 1.84 (s, 3H, C*H*$_3$ of Cp*), 2.04 (s, 3H, C*H*$_3$ of Cp*), 2.19 (d, *J*=15.1, 1H, C*H*$_2$), 2.90 (d, *J*=15.1, 1H, C*H*$_2$), 5.41 (br, 1H, C*H*), 6.51 (d, *J*=7.8, 2H, Ph*H*), 6.84 – 6.87 (m, 3H, Ph*H*), 6.95 – 7.00 (m, 2H, Ph*H*), 7.08 (td, *J*=7.4, 1.0, 3H, Ph*H*), 7.33 (dd, *J*=8.3, 7.1, 3H, Ph*H*), 7.45 – 7.51 (m, 2H, Ph*H*). **^{11}B{^{1}H} NMR** (160 MHz, C_6D_6) δ (ppm) = 44.5. **^{13}C{^{1}H} NMR** (126 MHz, C_6D_6) δ (ppm) = 2.58, 10.73, 11.19, 13.01, 13.96, 14.25, 19.56, 114.55, 115.39, 120.28, 121.27, 121.84, 121.97, 124.26, 125.10, 125.66, 125.86, 127.39, 128.18, 128.39, 130.30, 130.97, 146.66, 148.28, 149.10, 210.70. **^{29}Si NMR** (100 MHz, C_6D_6) δ (ppm) = 9.04. **HRMS-LIFDI** (m/z): Calculated (found) for $C_{50}H_{47}B_2N_1Zr_1$ $[M]^+$ 773.2936 (773.2922)

Synthesis of **5b**:

2h (111 mg, 0.2 mmol) and **1a** (98 mg, 0.2 mmol) were dissolved in 1 mL C_6D_6. The mixture was heated at 70 °C for 2 days and after the removal of volatiles yielded as a black oil, which crystallized upon storing concentrated hexane solution at −30 °C overnight. The residue was filtered and washed with cold hexane three times to obtain **5b** as a brown solid (113 mg, 0.13 mmol, 65%).

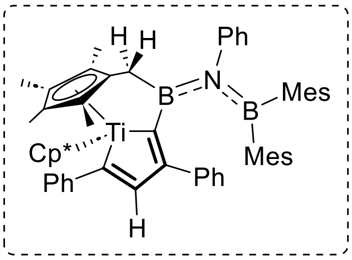

^{1}H NMR (400 MHz, C_6D_6) δ (ppm) = 1.12 (s, 3H, CH_3 of Cp*), 1.64 (s, 3H, CH_3 of Cp*), 1.75 (s, 3H, CH_3 of Cp*), 1.81 (s, 15H, CH_3 of Cp*), 1.88 (s, 3H, CH_3 of Mes), 1.89 (s, 3H, CH_3 of Cp*), 1.95 (d, *J*=16.9 Hz, 1H, C*H*$_2$), 2.14 (s, 3H, CH_3 of Mes), 2.28 (s, 3H, CH_3 of Mes), 2.38 (s, 3H, CH_3 of Mes), 2.40 (d, *J*=16.9 Hz, 1H, C*H*$_2$), 2.55 (s, 3H, CH_3 of Mes), 2.75 (s, 3H, CH_3 of Mes), 6.41 (s, 1H, *m*-H of Mes), 6.48 (s, 1H, *m*-H of Mes), 6.61– 6.66 (m, 1H, Ph*H*), 6.69 (t, *J*=7.5, 2H, Ph*H*), 6.73 (s, 1H, C*H*), 6.84 (br, 2H,

Ph), 6.86 (s, 1H, *m*-H of Mes), 6.91 (s, 1H, *m*-H of Mes), 6.93 – 6.98 (m, 1H, Ph*H*), 7.02 (td, *J*=7.4, 1.2, 2H, Ph*H*), 7.05 – 7.10 (m, 1H, Ph*H*), 7.15 – 7.18 (m, 3H, Ph*H*), 7.23 – 7.29 (m, 2H, Ph*H*). **^{11}B{^{1}H} NMR** (160 MHz, C_6D_6) δ (ppm) = 54.0. **^{13}C{^{1}H} NMR** (126 MHz, C_6D_6) δ (ppm) = 10.58, 11.54, 12.55, 13.33, 14.01, 14.20, 20.36, 20.86, 21.32, 23.85, 24.10, 24.13, 24.76, 115.70, 115.74, 120.40, 121.37, 121.99, 124.48, 125.34, 125.51, 125.87, 126.63, 127.30, 127.74, 128.18, 128.45, 128.55, 128.68, 128.76, 128.80, 132.47, 133.37, 136.76, 137.79, 138.96, 139.46, 139.99, 140.28, 141.07, 142.65, 145.74, 149.01, 149.56, 211.49. **HRMS-LIFDI** (m/z): for $C_{60}H_{67}B_2NTi$ $[M]^+$ Calculated 871.4934, found 871.4915.

Synthesis of **6h**:

2h (166 mg, 0.3 mmol) and **1b** (105 mg, 0.3 mmol) were dissolved in 1 mL C_6D_6. The mixture was stirred at room temperature for 2 hours and then the removal of volatiles yielded a dark brown oil, which crystallized upon storing a concentrated hexane solution at −35°C overnight. The residue was filtered and washed with cold hexane three times to obtain **6h** as an orange solid (138 mg, 0.19 mmol, 63%).

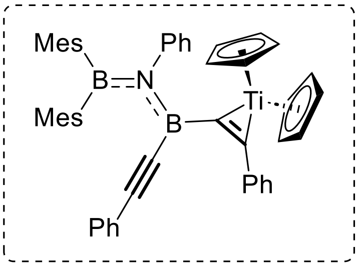

^{1}H NMR (400 MHz, C_6D_6) δ (ppm) = 1.81 (br, 6H, *o*-CH_3 of Mes), 1.98 (s, 3H, *p*-CH_3 of Mes), 2.08 (s, 3H, *p*-CH_3 of Mes), 2.33 (s, 6H, *o*-CH_3 of Mes), 6.30 (s, 10H, Cp), 6.54 (s, 2H, *m*-H of Mes), 6.62 (s, 2H, *m*-H of Mes), 6.85 (br, 3H, Ph-*H*), 6.96 – 6.98 (m, 5H, Ph-*H*), 7.01 – 7.04 (m, 5H, Ph-*H*), 7.25 (d, 2H, Ph-*H*, *J* = 8.0 Hz). **^{11}B{^{1}H} NMR** (160 MHz, C_6D_6) δ (ppm) = 54.8, 32.4. **^{13}C{^{1}H} NMR** (126 MHz, C_6D_6) δ (ppm) = 21.09, 21.20, 22.96, 23.05, 117.51, 125.60, 127.33, 127.57, 128.32, 128.35, 128.40, 128.59, 128.74, 130.47, 137.36, 138.07, 140.14, 140.58, 142.74, 149.58. **HRMS-LIFDI** (m/z): for $C_{50}H_{47}B_2NTi$ $[M]^+$ Calculated 731.3369, found 731.3361.

NMR Spectroscopy

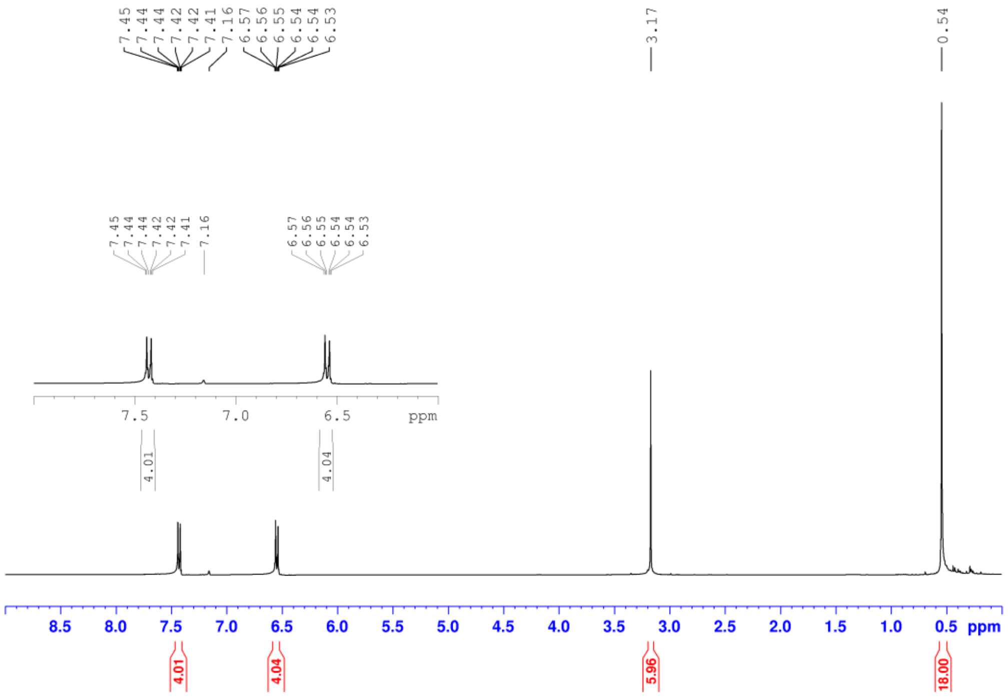

Figure S1: ^{1}H-NMR (400MHz, 298K) spectrum of compound **2c** in C_6D_6.

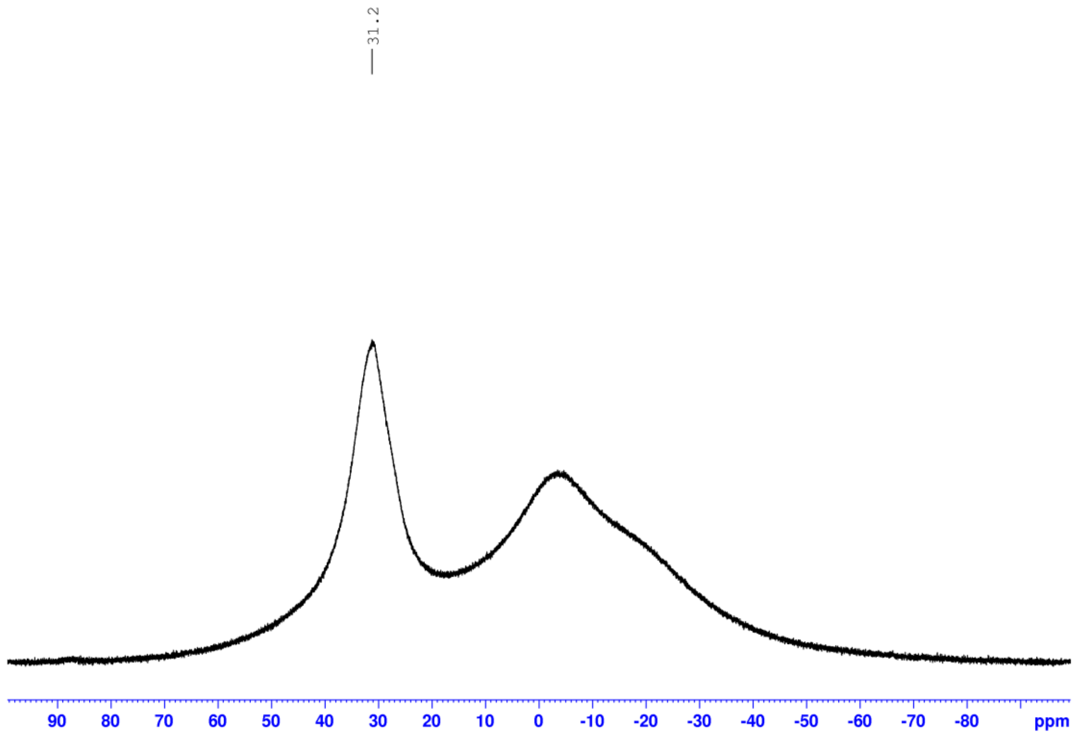

Figure S2: ^{11}B-NMR (128MHz, 298K) spectrum of compound **2c** in C_6D_6.

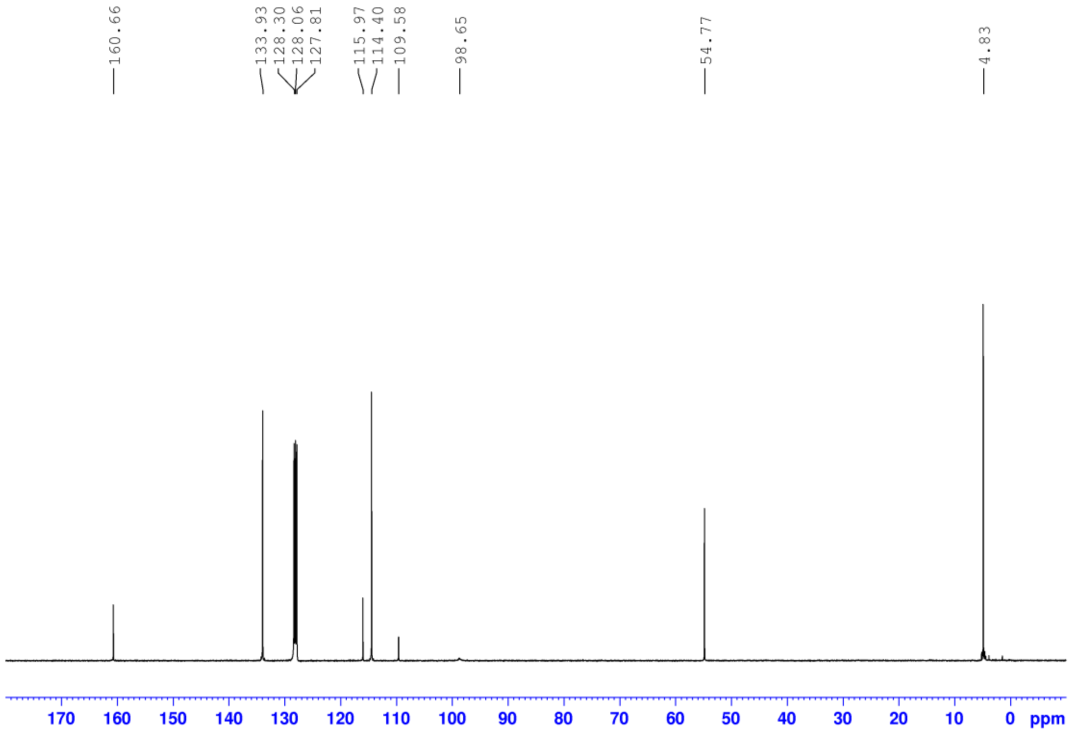

Figure S3: ^{13}C-NMR (100MHz, 298K) spectrum of compound **2c** in C_6D_6.

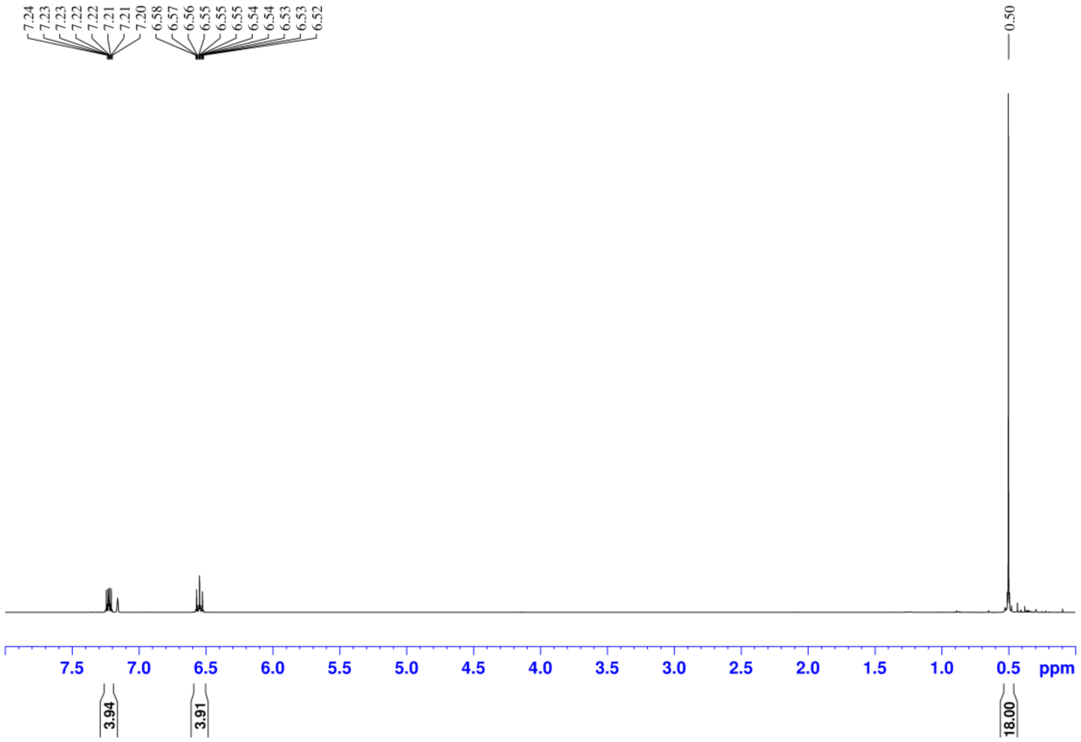

Figure S4: ^{1}H-NMR (400MHz, 298K) spectrum of compound **2d** in C_6D_6.

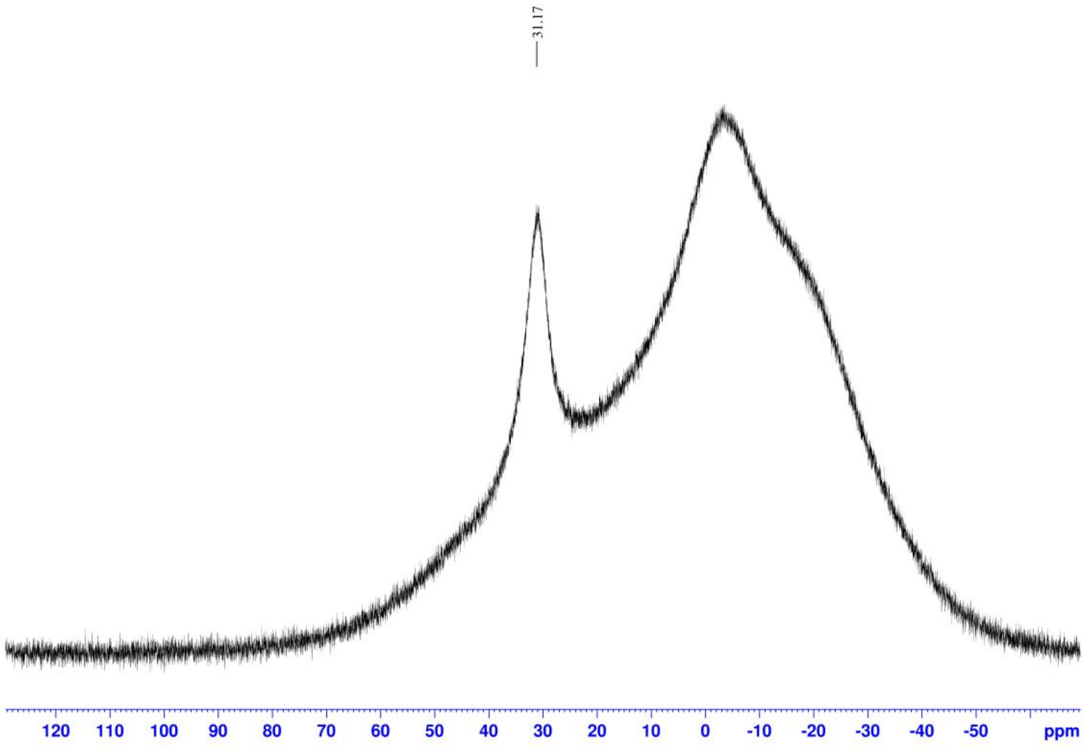

Figure S5: ^{11}B-NMR (160MHz, 298K) spectrum of compound **2d** in C_6D_6.

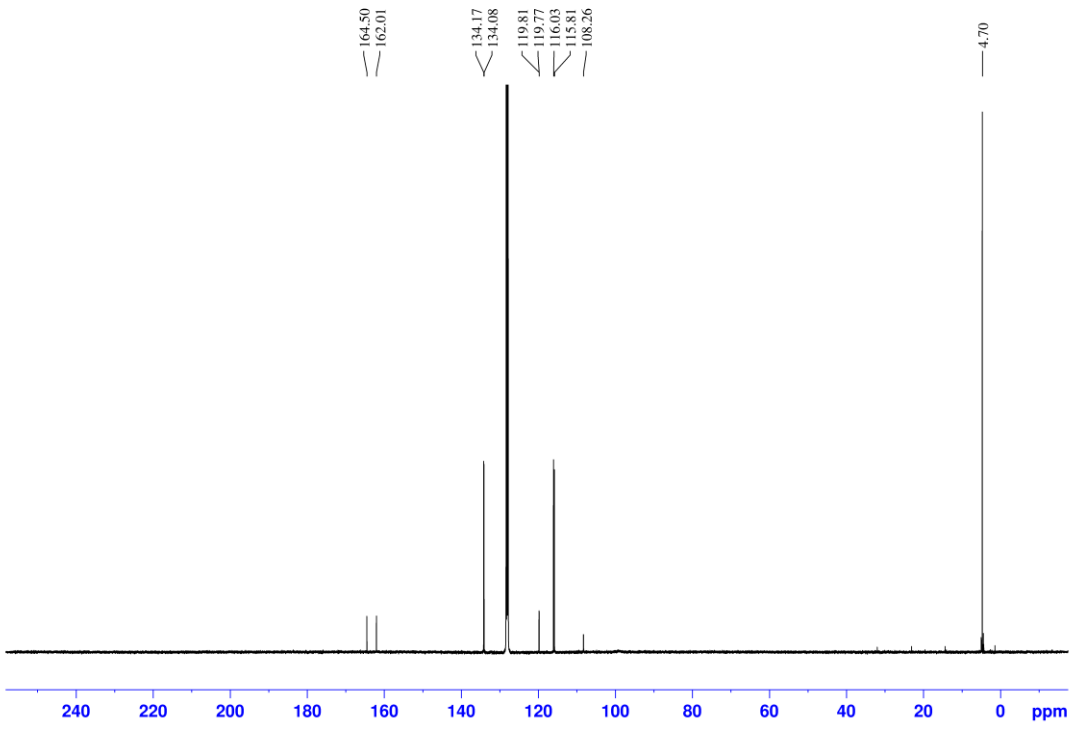

Figure S6: ^{13}C-NMR (100MHz, 298K) spectrum of compound **2d** in C_6D_6.

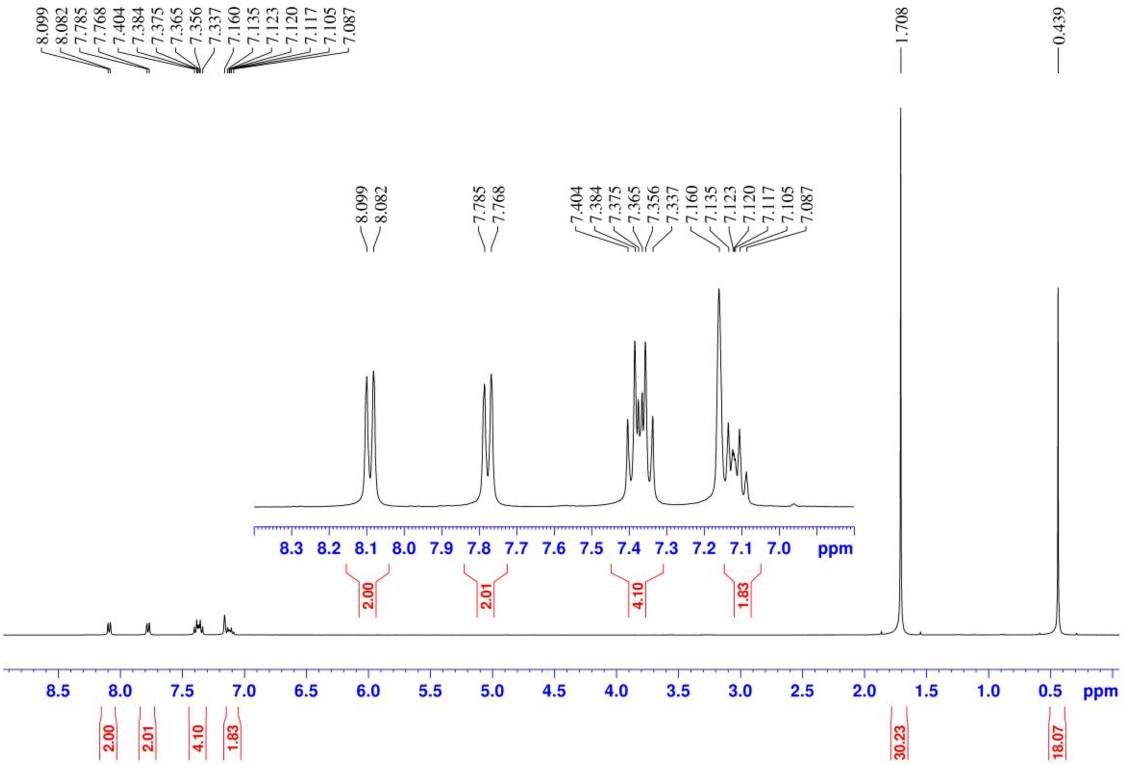

Figure S7: ^{1}H-NMR (400MHz, 298K) spectrum of compound **3a** in C_6D_6.

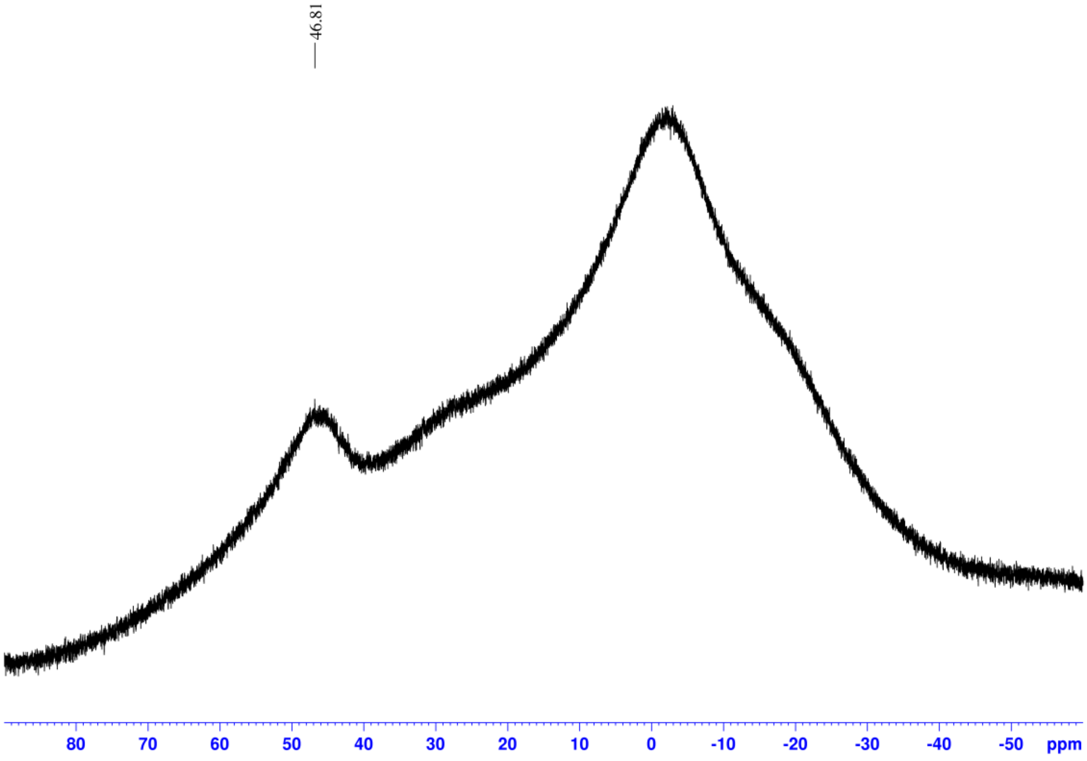

Figure S8: ^{11}B-NMR (160MHz, 298K) spectrum of compound **3a** in C_6D_6.

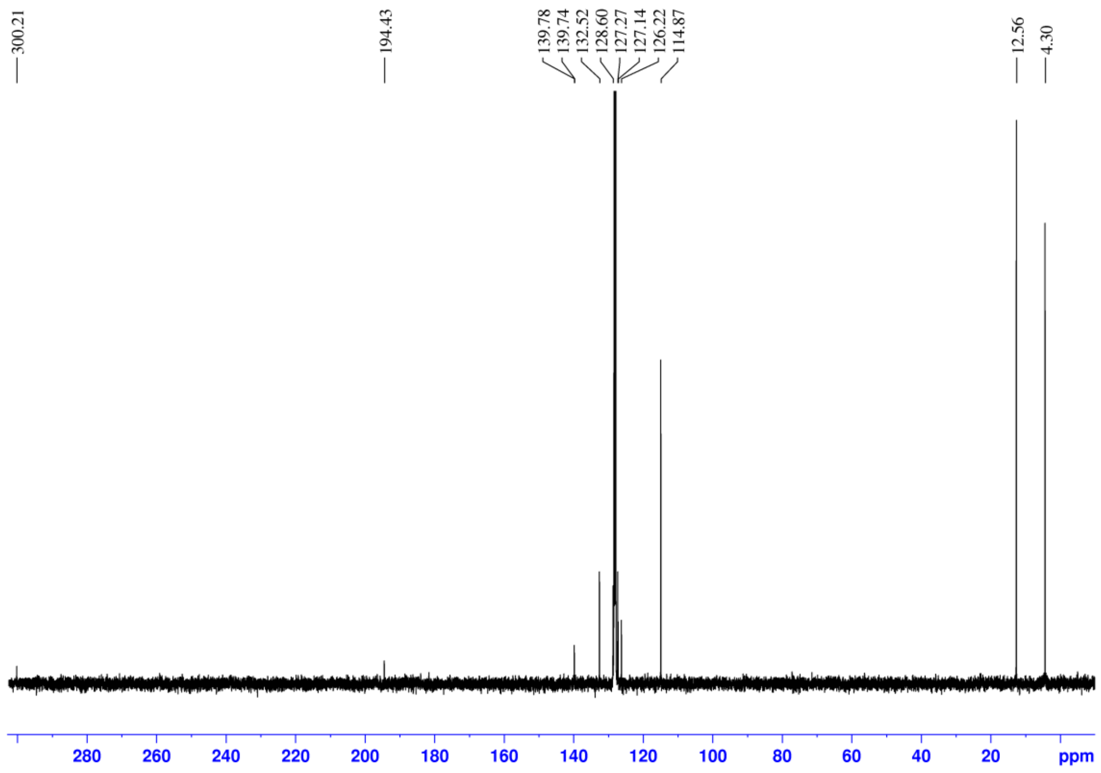

Figure S9: ^{13}C-NMR (100MHz, 298K) spectrum of compound **3a** in C_6D_6.

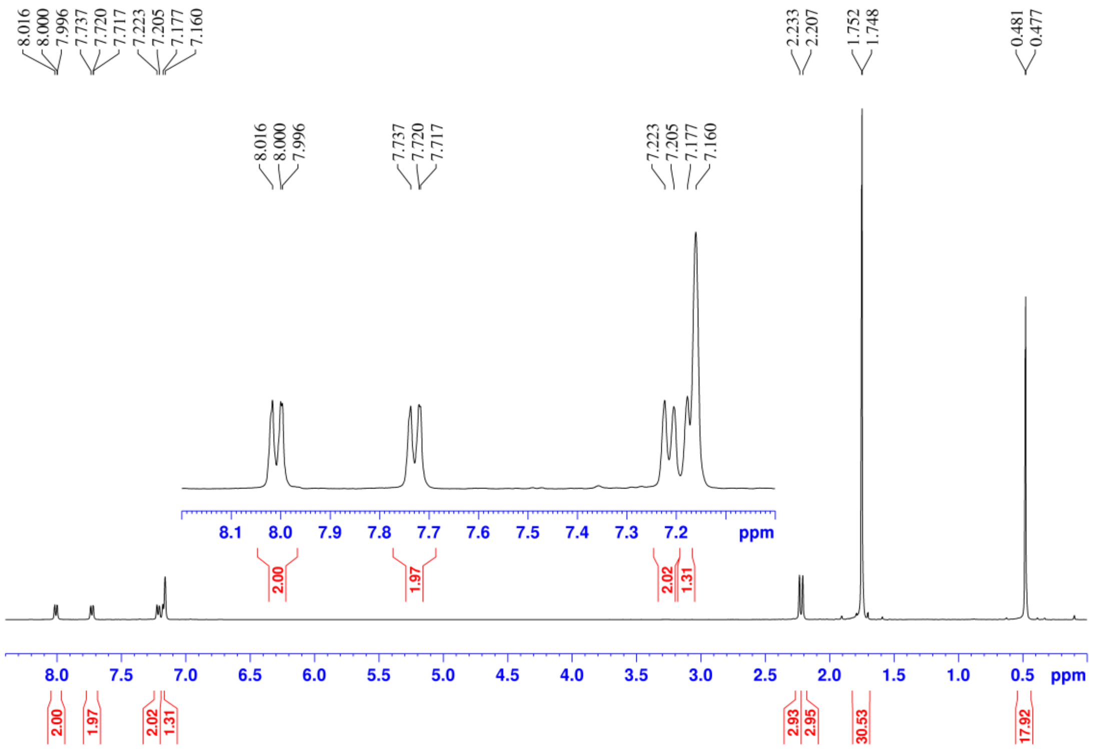

Figure S10: ^{1}H-NMR (400MHz, 298K) spectrum of compound **3b** in C_6D_6.

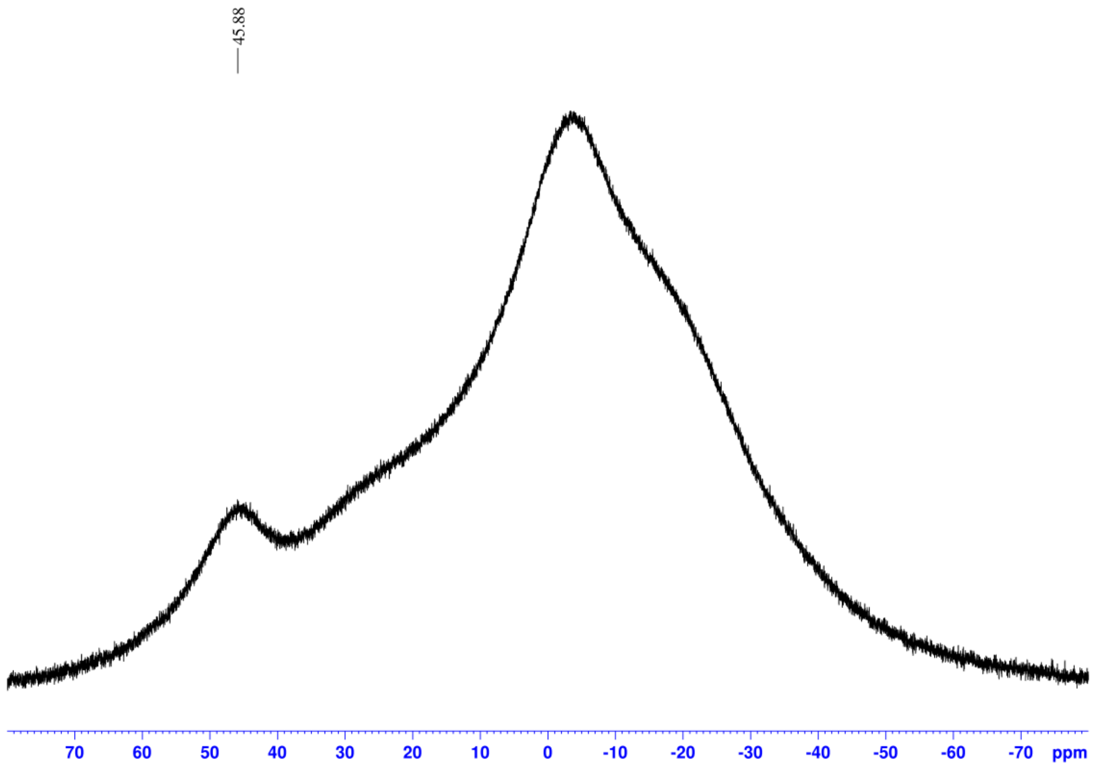

Figure S11: ^{11}B-NMR (128MHz, 298K) spectrum of compound **3b** in C_6D_6.

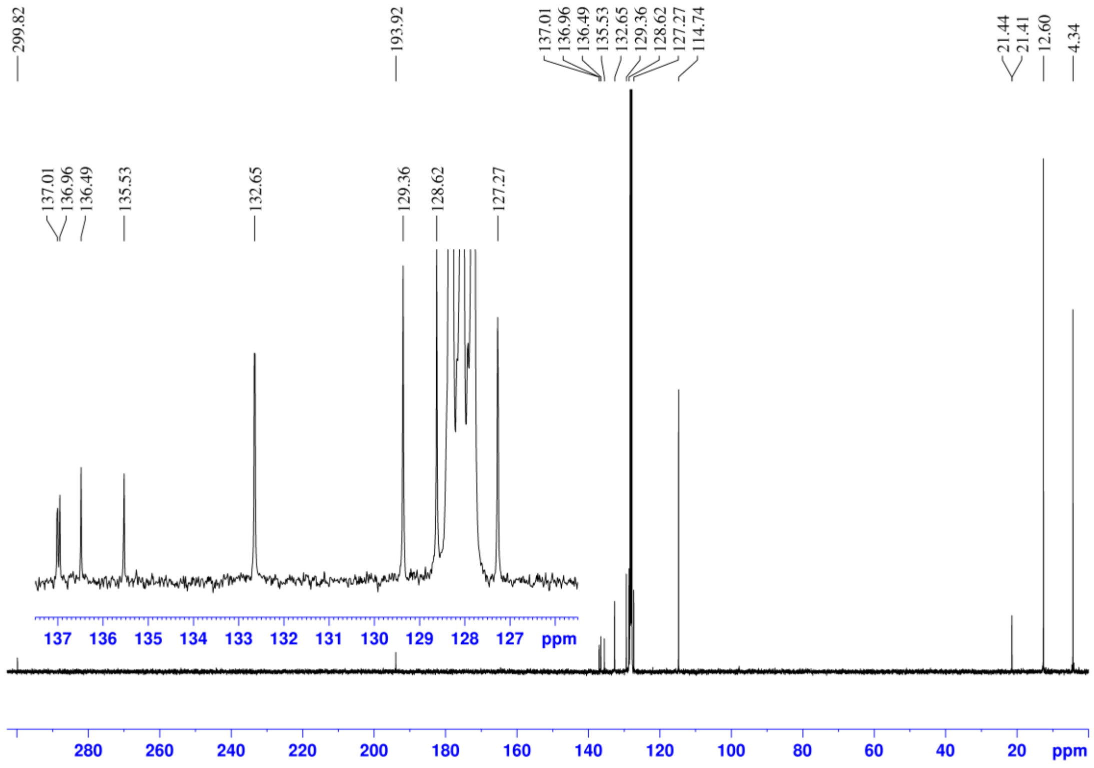

Figure S12: ^{13}C-NMR (400MHz, 298K) spectrum of compound **3b** in C_6D_6.

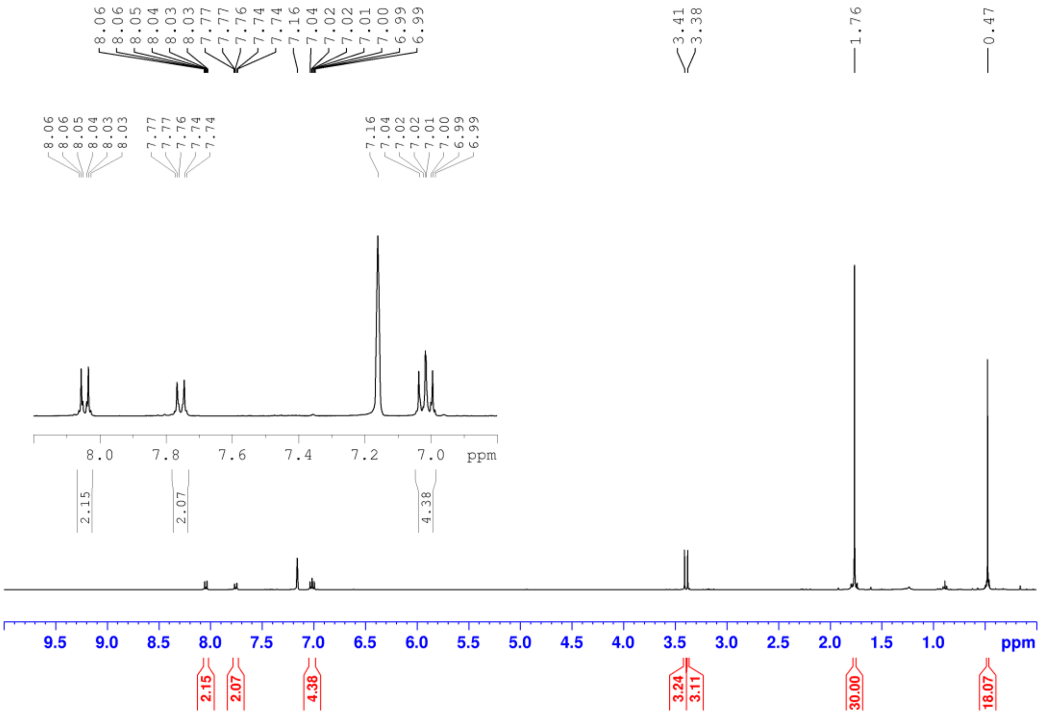

Figure S13*:* ^{1}H-NMR (400MHz, 298K) spectrum of compound **3c** in C_6D_6.

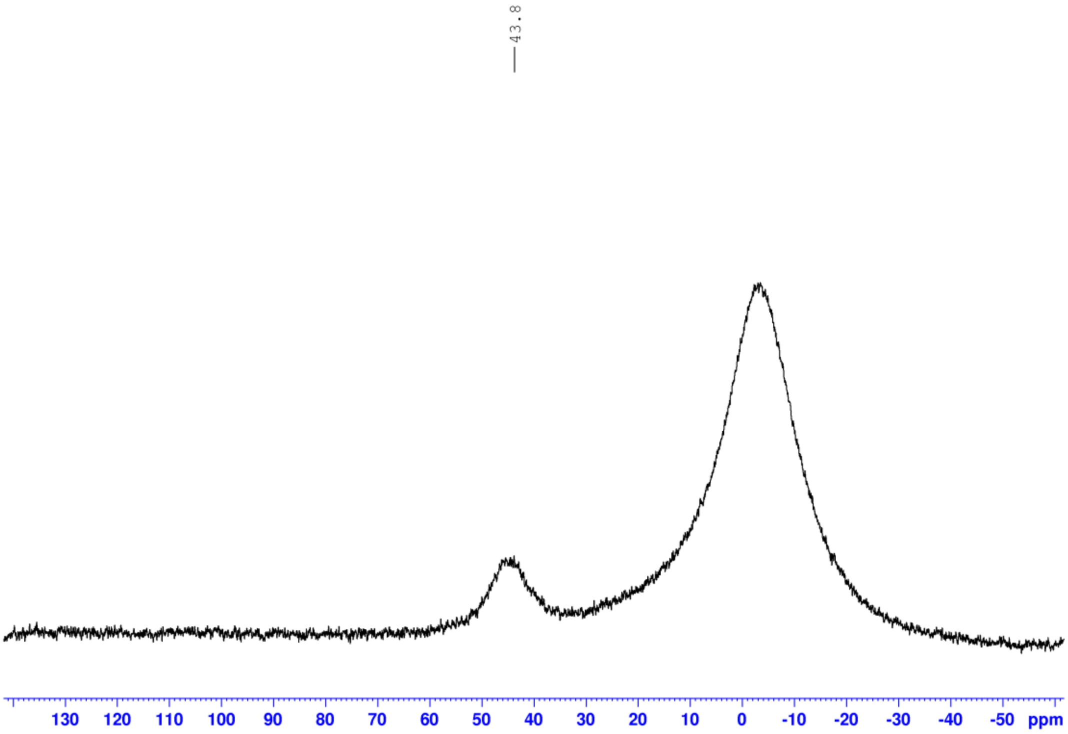

Figure S14*:* ^{11}B-NMR (160MHz, 298K) spectrum of compound **3c** in C_6D_6.

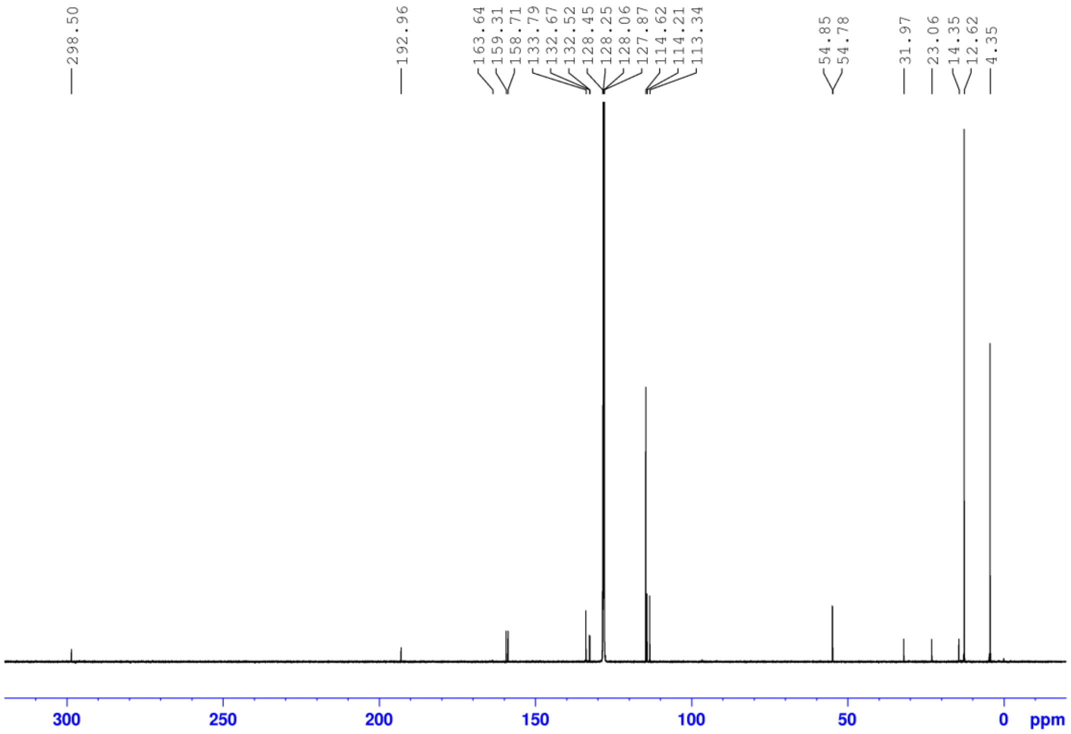

Figure S15: ^{13}C-NMR (126MHz, 298K) spectrum of compound **3c** in C_6D_6.

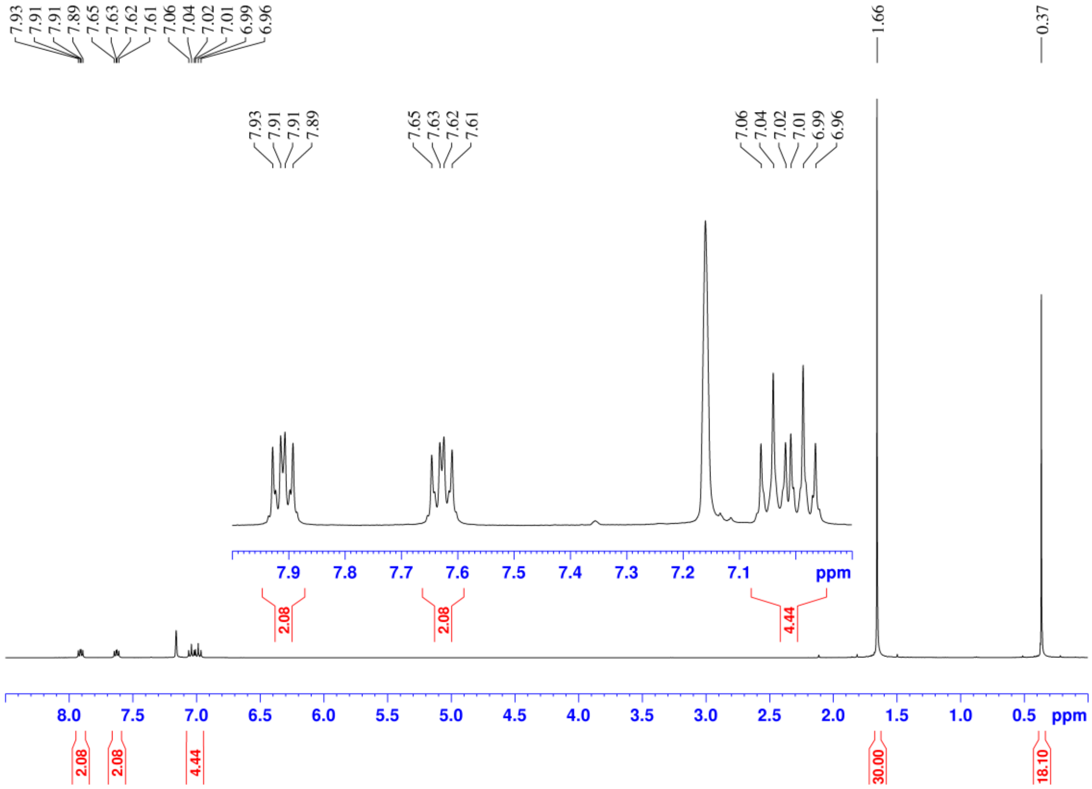

Figure S16: ^{1}H-NMR (400MHz, 298K) spectrum of compound **3d** in C_6D_6.

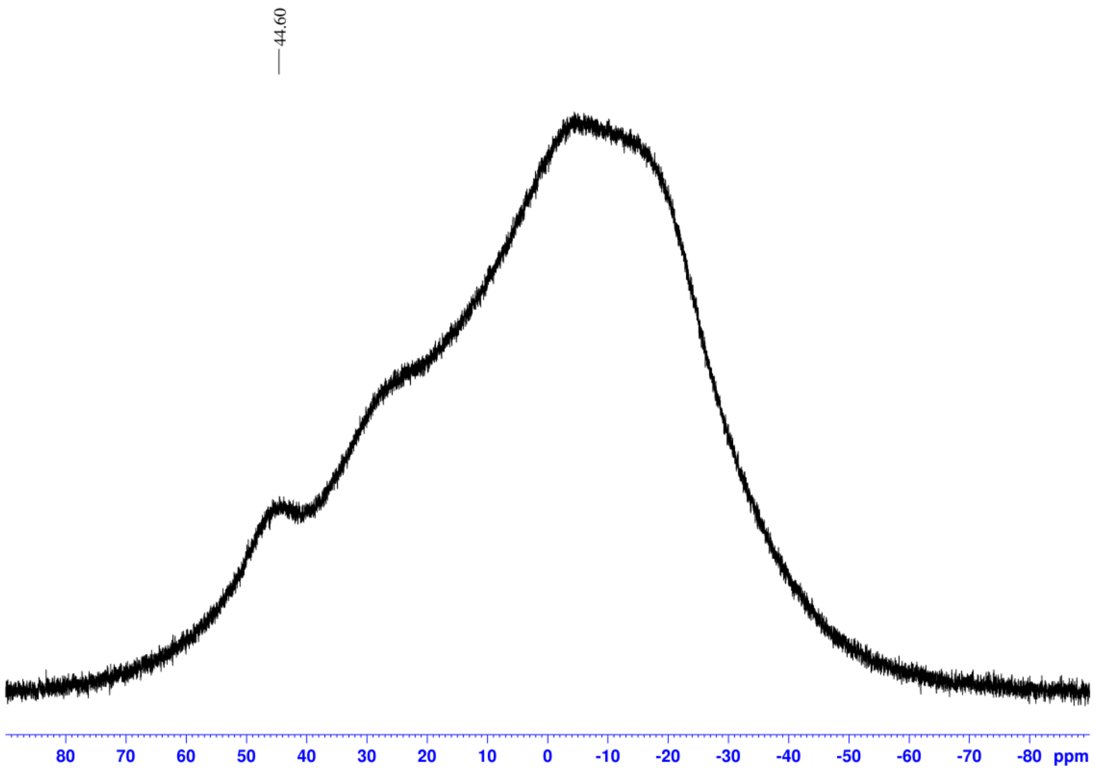

Figure S17: ^{11}B-NMR (400MHz, 298K) spectrum of compound **3d** in C_6D_6.

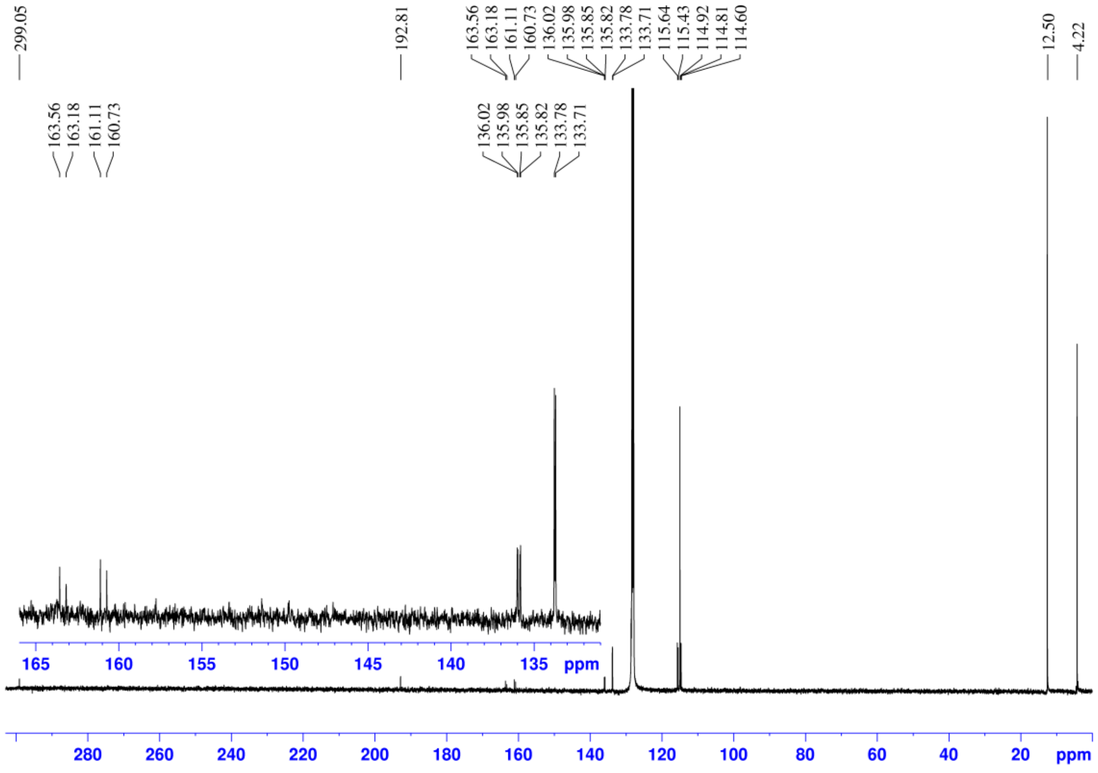

Figure S18: ^{13}C-NMR (400MHz, 298K) spectrum of compound **3d** in C_6D_6.

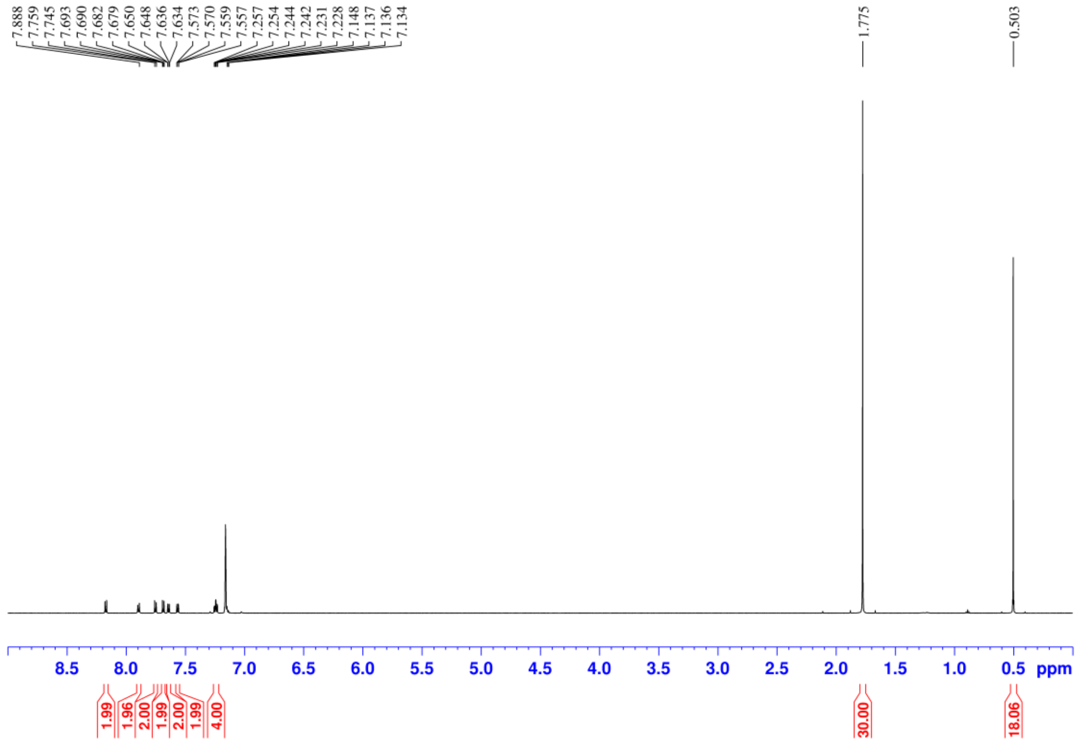

Figure S19: ^{1}H-NMR (600MHz, 298K) spectrum of compound **3e** in C_6D_6.

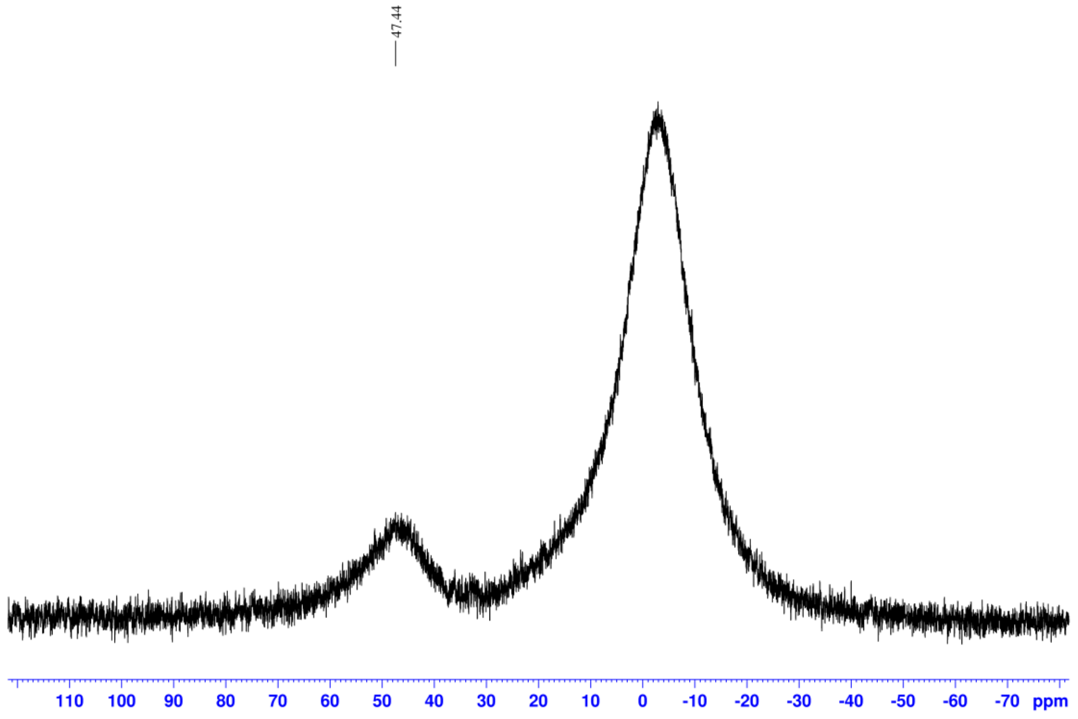

Figure S20: ^{11}B-NMR (160MHz, 298K) spectrum of compound **3e** in C_6D_6.

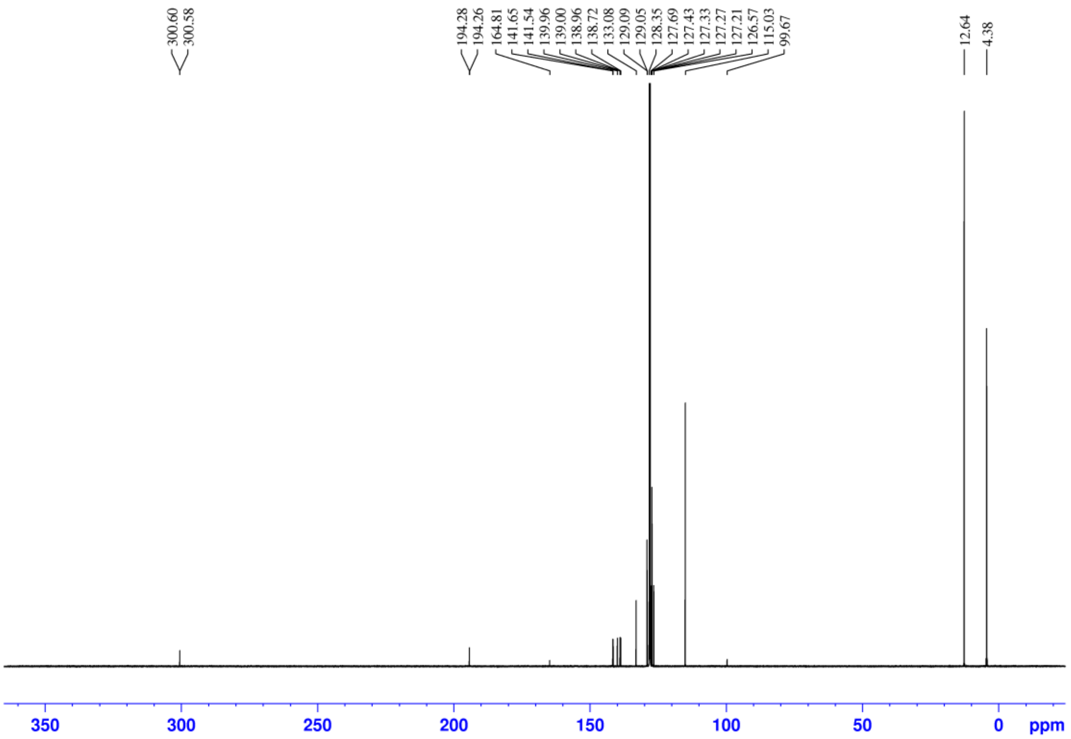

Figure S21: ^{13}C-NMR (150MHz, 298K) spectrum of compound **3e** in C_6D_6.

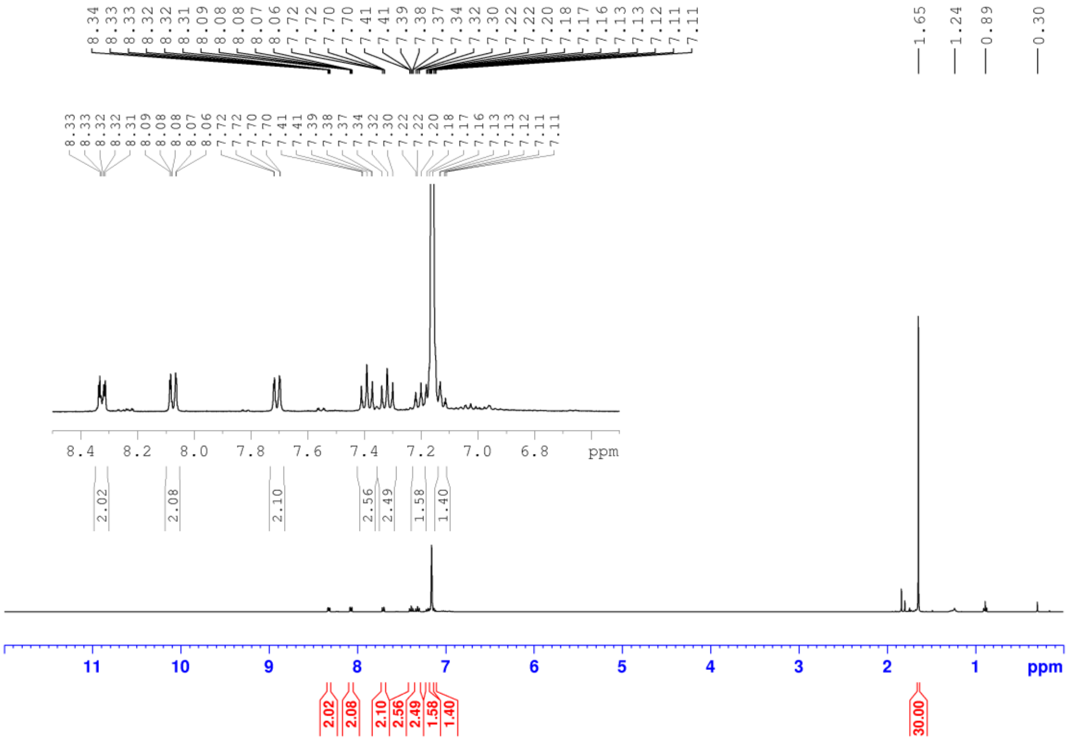

Figure S22: ^{1}H-NMR (400MHz, 298K) spectrum of compound **3f** in C_6D_6.

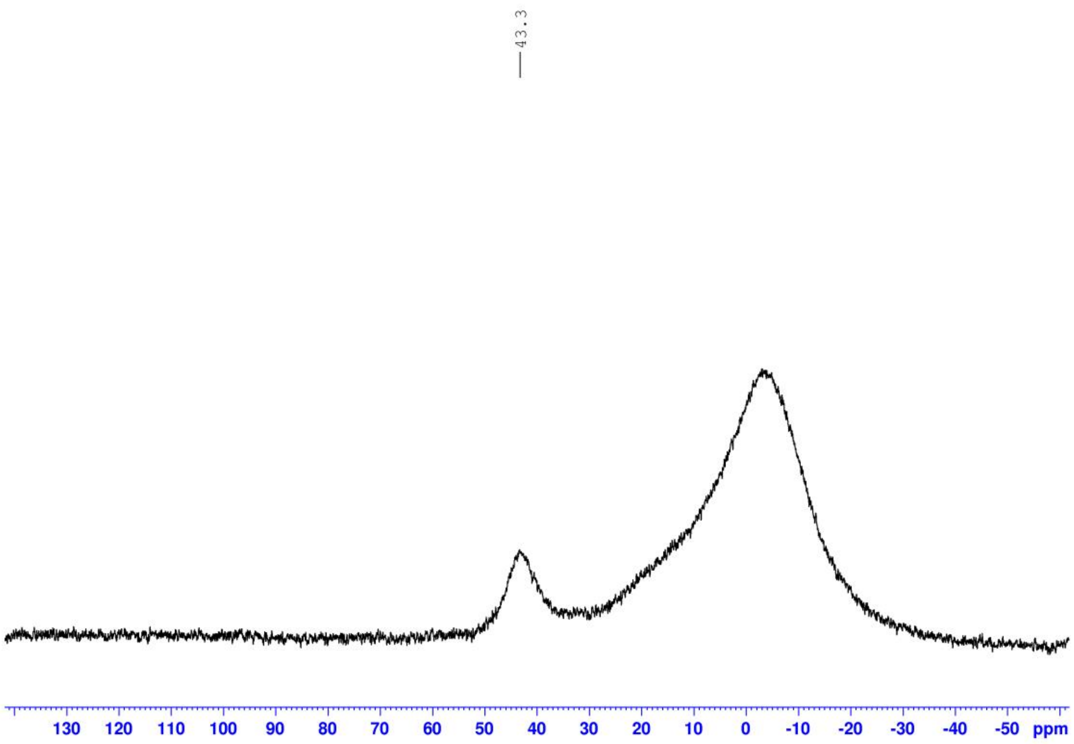

Figure S23: ^{11}B-NMR (160MHz, 298K) spectrum of compound **3f** in C_6D_6.

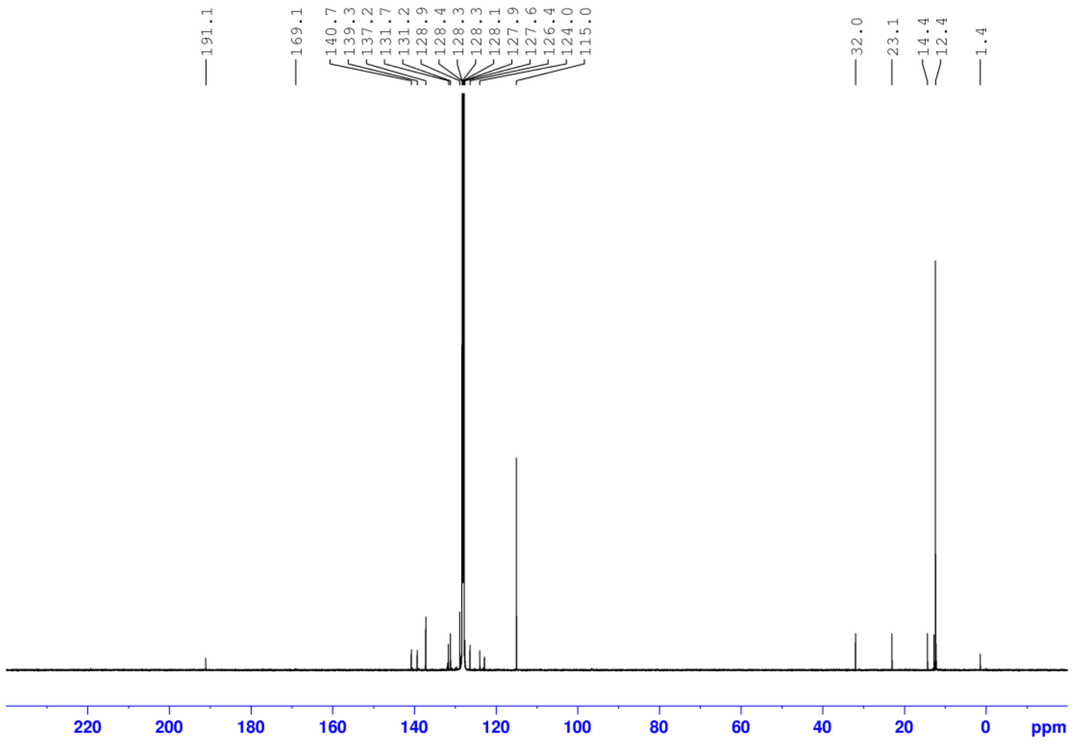

Figure S24: ^{13}C-NMR (126MHz, 298K) spectrum of compound **3f** in C_6D_6.

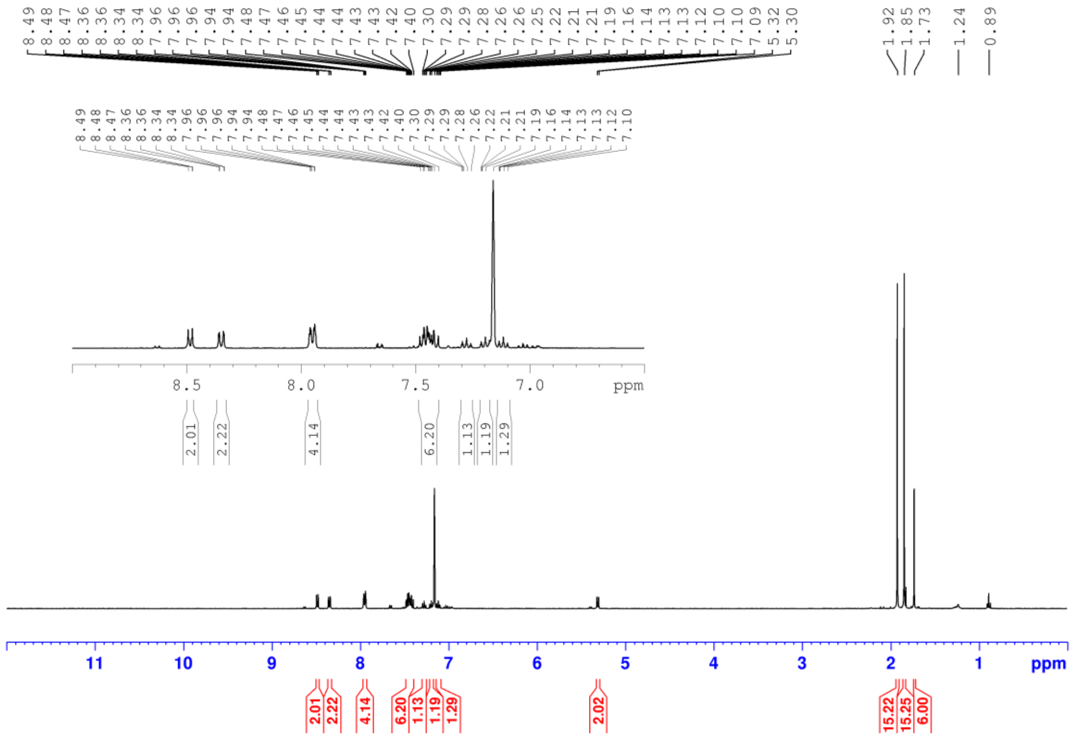

Figure S25: ^{1}H-NMR (400MHz, 298K) spectrum of compound **3f-DMAP** in C_6D_6.

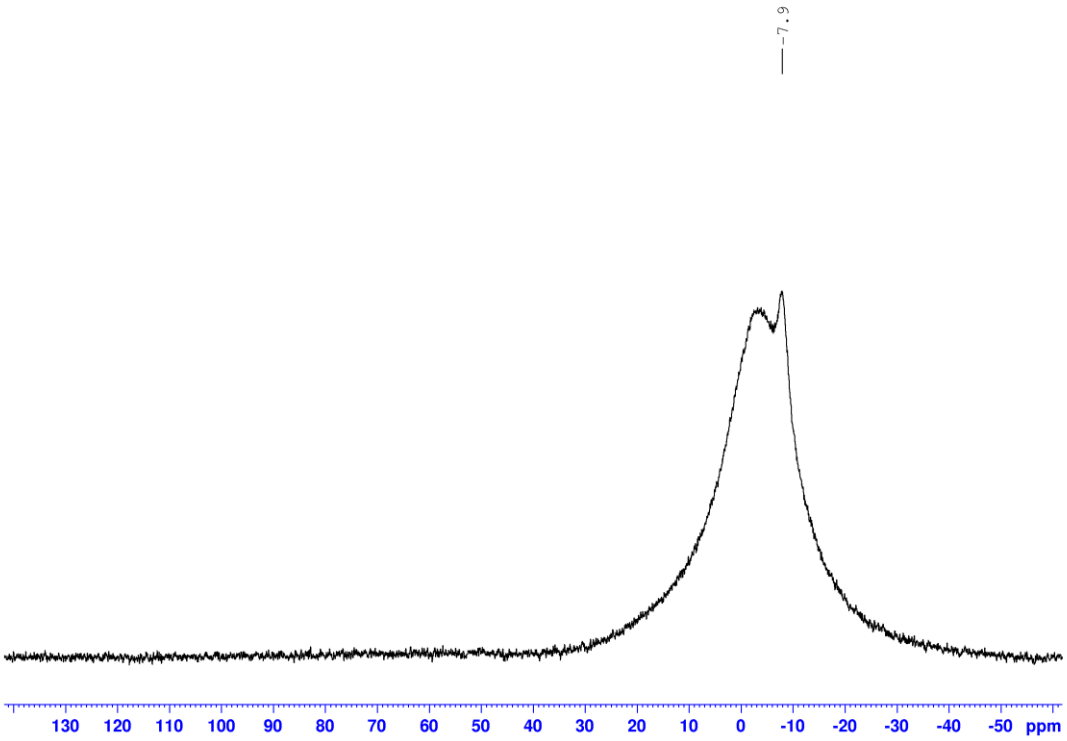

Figure S26: ^{11}B-NMR (160MHz, 298K) spectrum of compound **3f-DMAP** in C_6D_6.

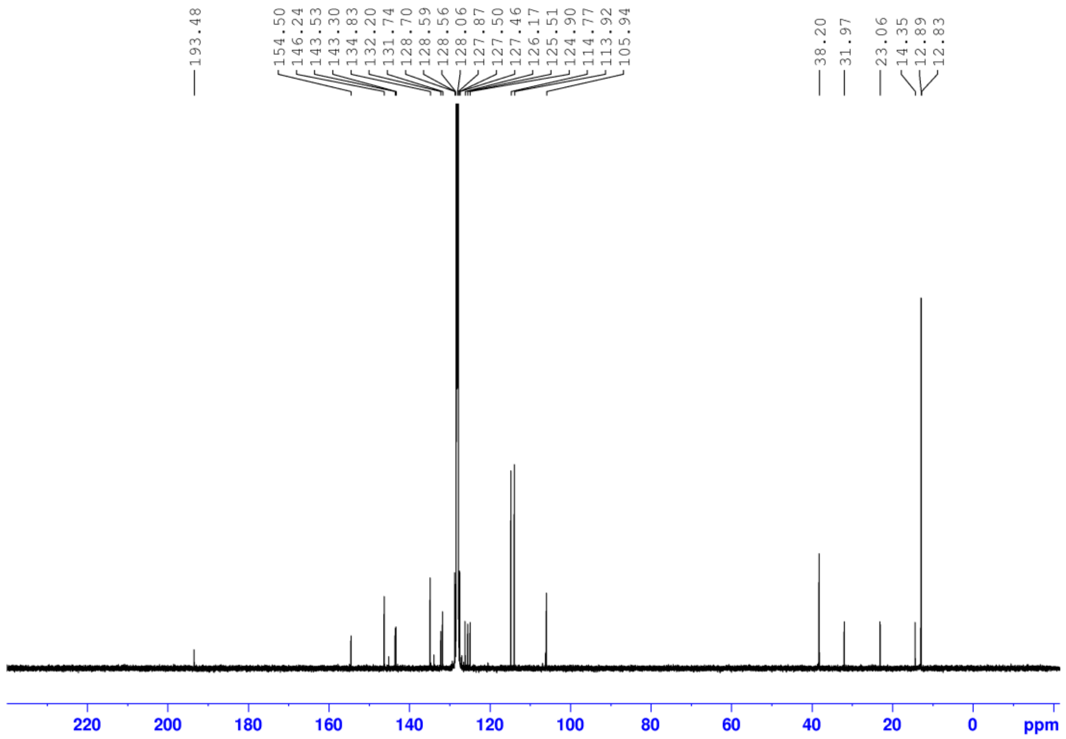

Figure S27: ^{13}C-NMR (126MHz, 298K) spectrum of compound **3f-DMAP** in C_6D_6.

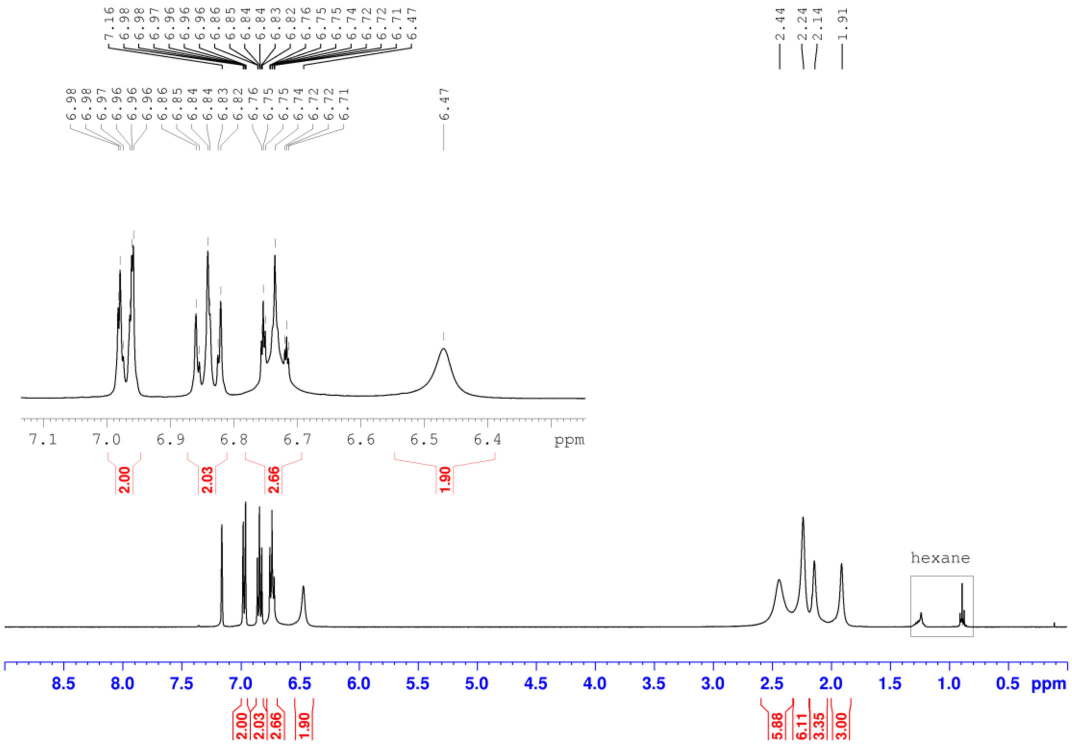

Figure S28: ^{1}H-NMR (400MHz, 298K) spectrum of compound **4b** in C_6D_6.

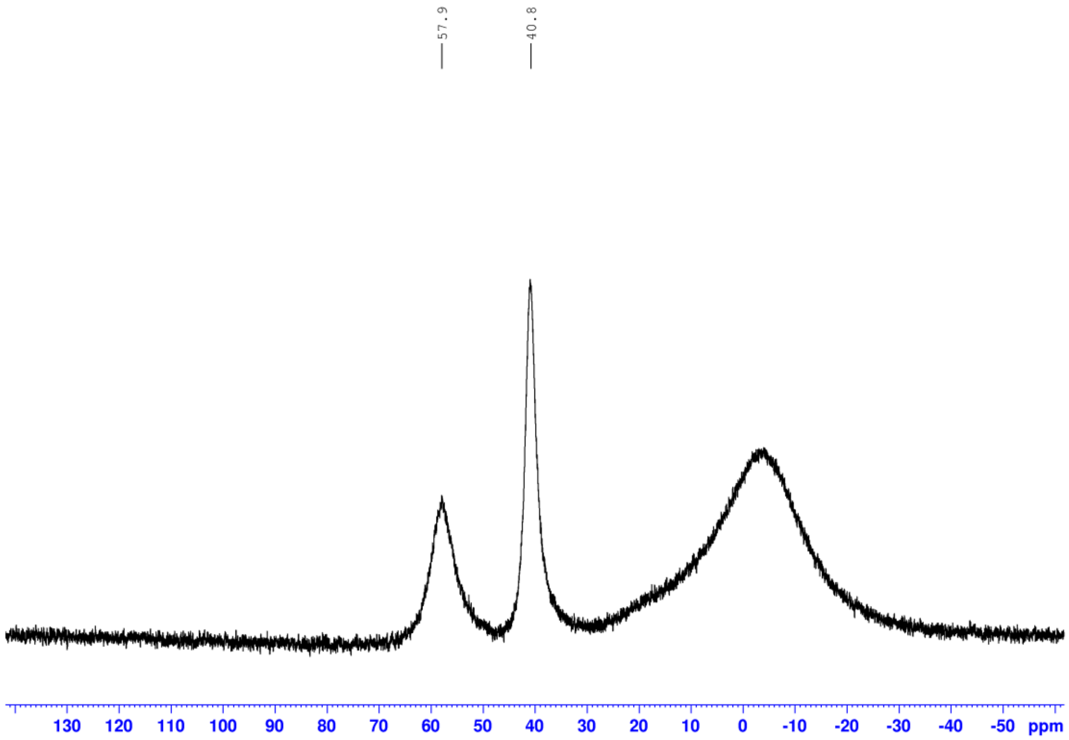

Figure S29: ^{11}B-NMR (160MHz, 298K) spectrum of compound **4b** in C_6D_6.

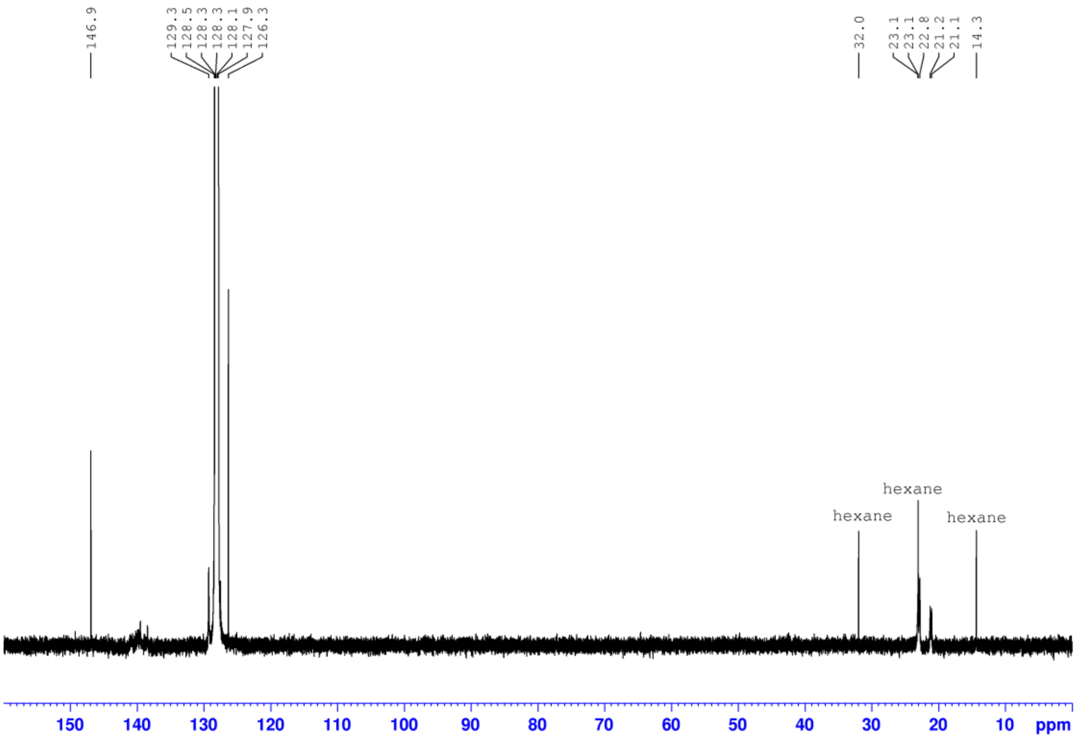

Figure S30: ^{13}C-NMR (126MHz, 298K) spectrum of compound **4b** in C_6D_6.

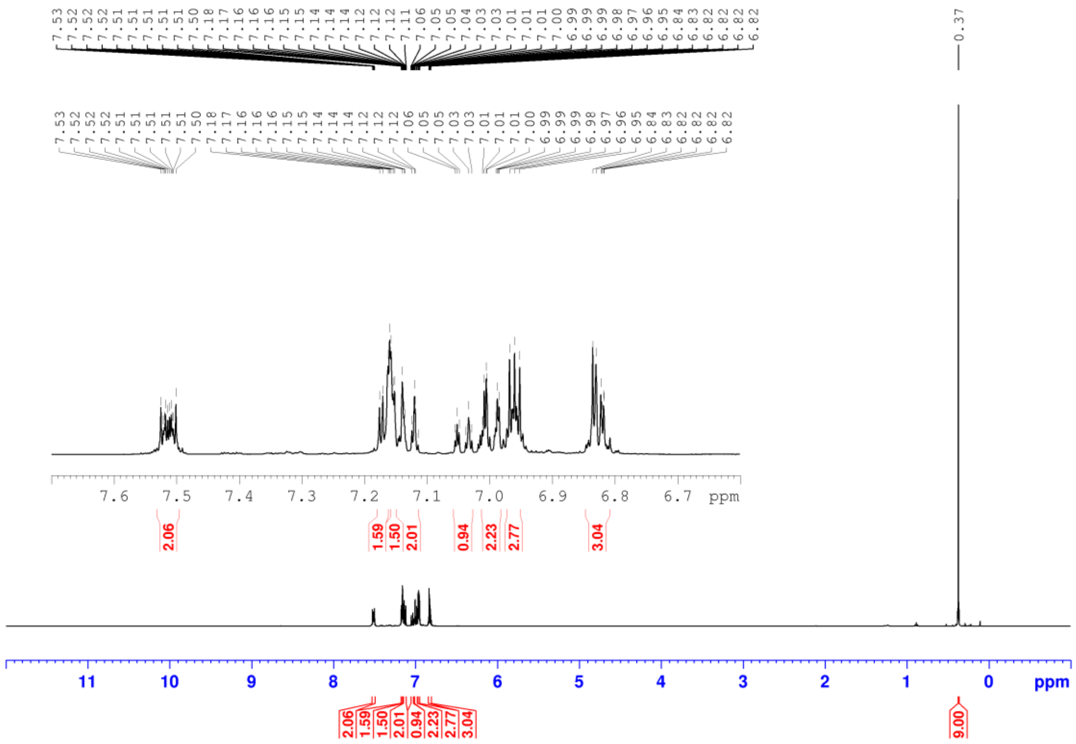

Figure S31: ^{1}H-NMR (400MHz, 298K) spectrum of compound **2g** in C_6D_6.

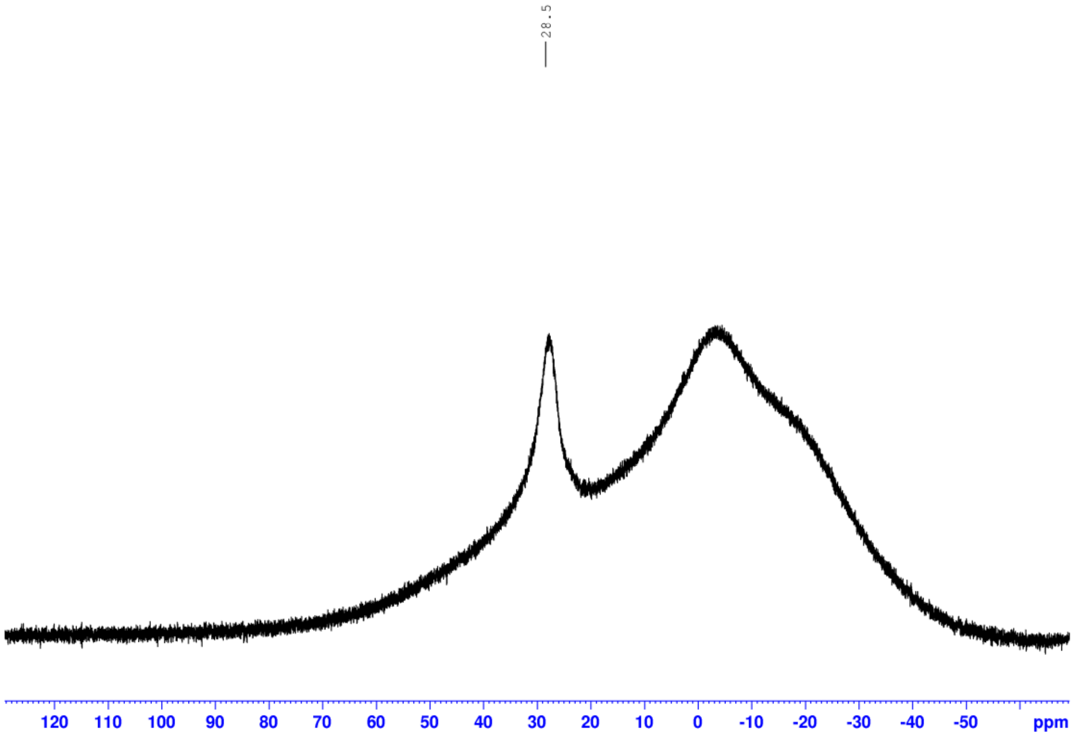

Figure S32: ^{11}B-NMR (128MHz, 298K) spectrum of compound **2g** in C_6D_6.

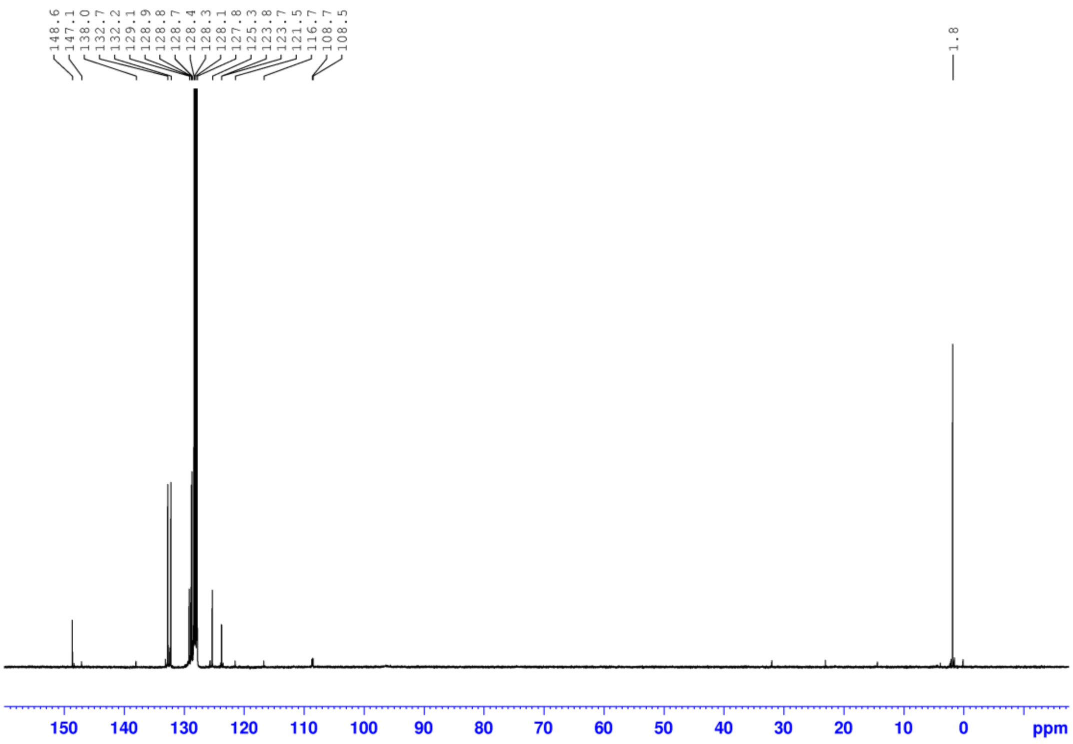

Figure S33: ^{13}C-NMR (100MHz, 298K) spectrum of compound **2g** in C_6D_6.

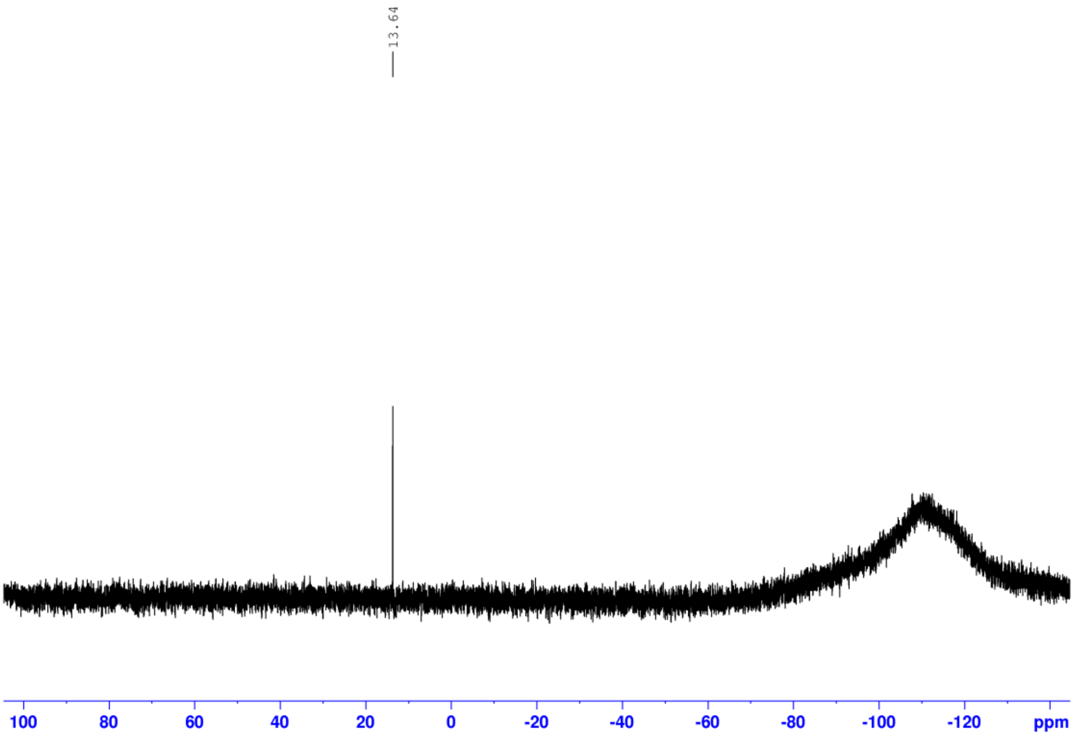

Figure S34: ^{29}Si-NMR (80MHz, 298K) spectrum of compound **2g** in C_6D_6.

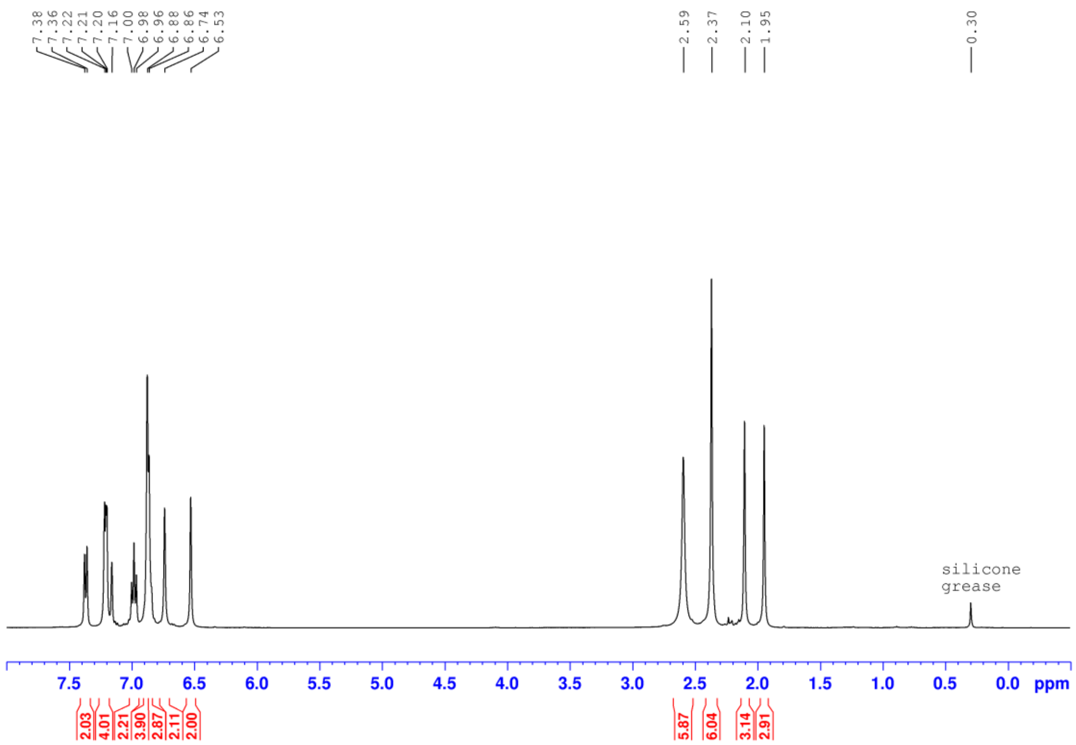

Figure S35: ^{1}H-NMR (400MHz, 298K) spectrum of compound **2h** in C_6D_6.

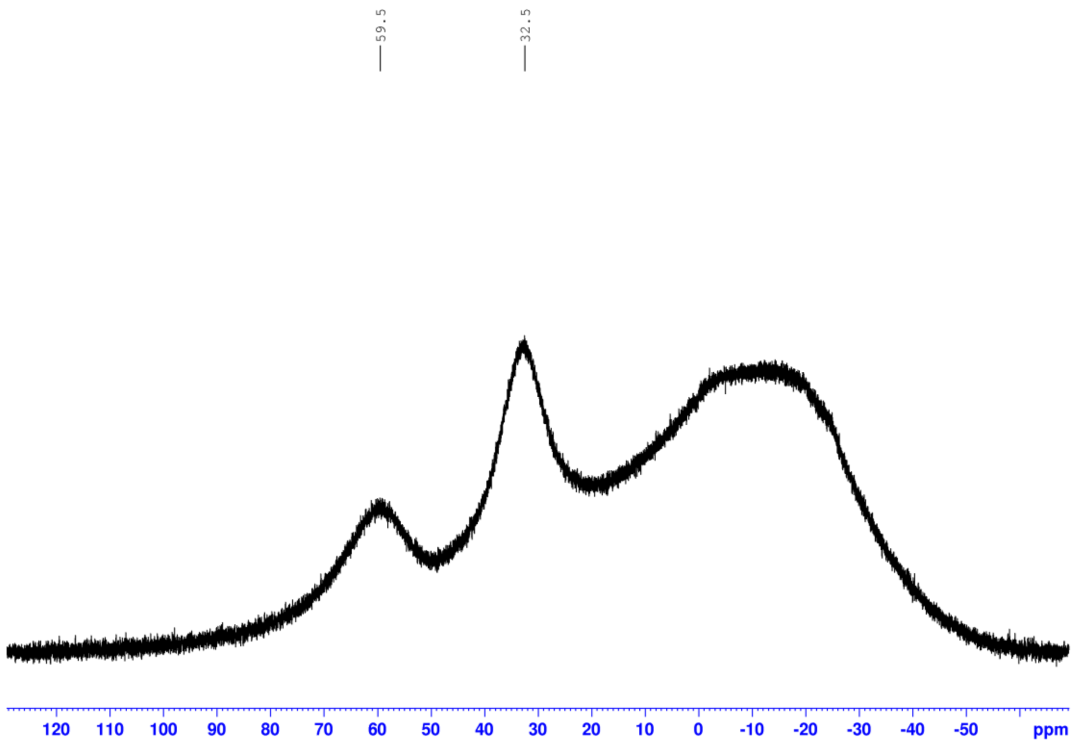

Figure S36: ^{11}B-NMR (128MHz, 298K) spectrum of compound **2h** in C_6D_6.

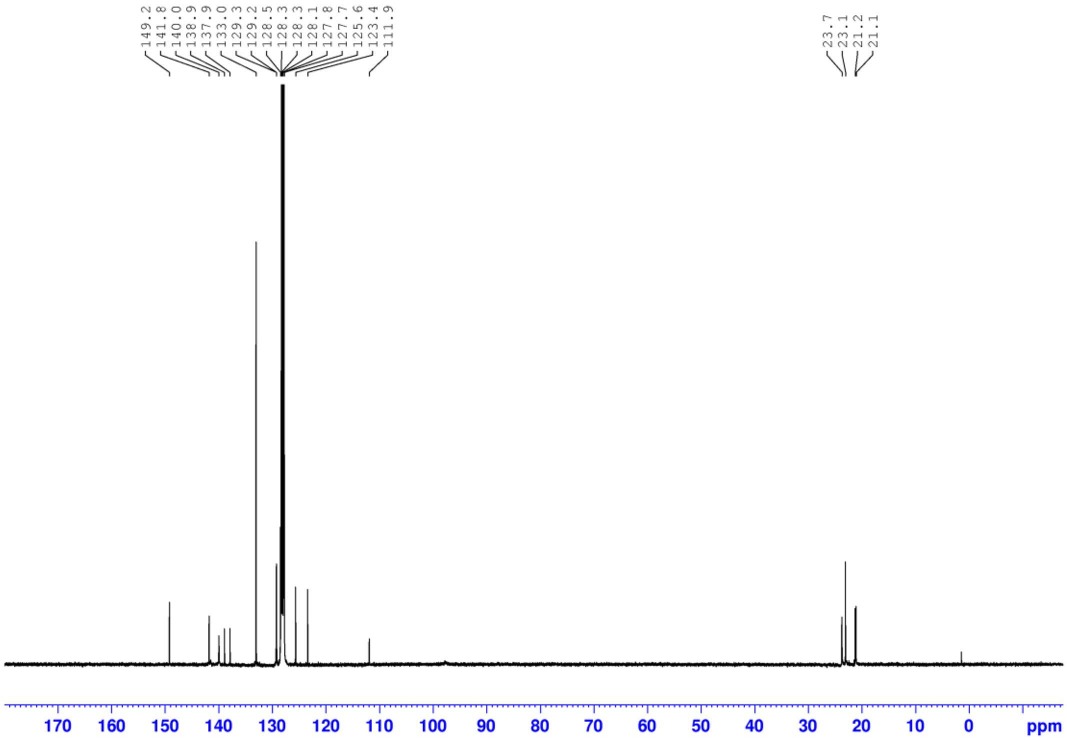

Figure S37: ^{13}C-NMR (100MHz, 298K) spectrum of compound **2h** in C_6D_6.

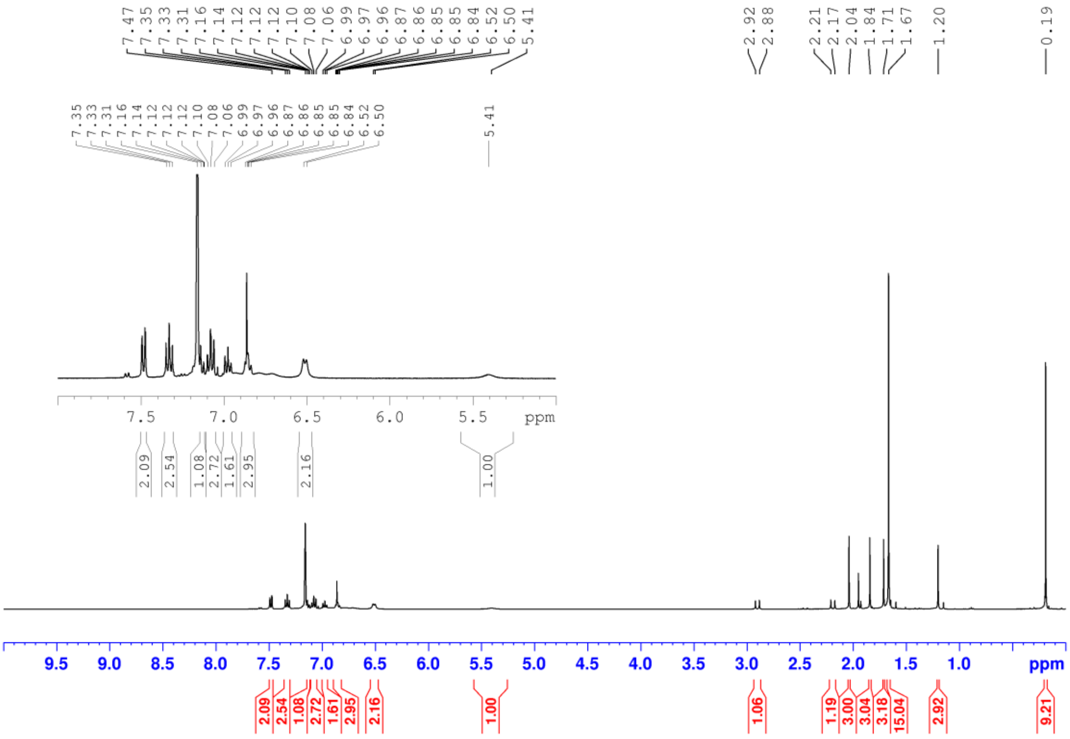

Figure S38: ^{1}H-NMR (400MHz, 298K) spectrum of compound **5a** in C_6D_6.

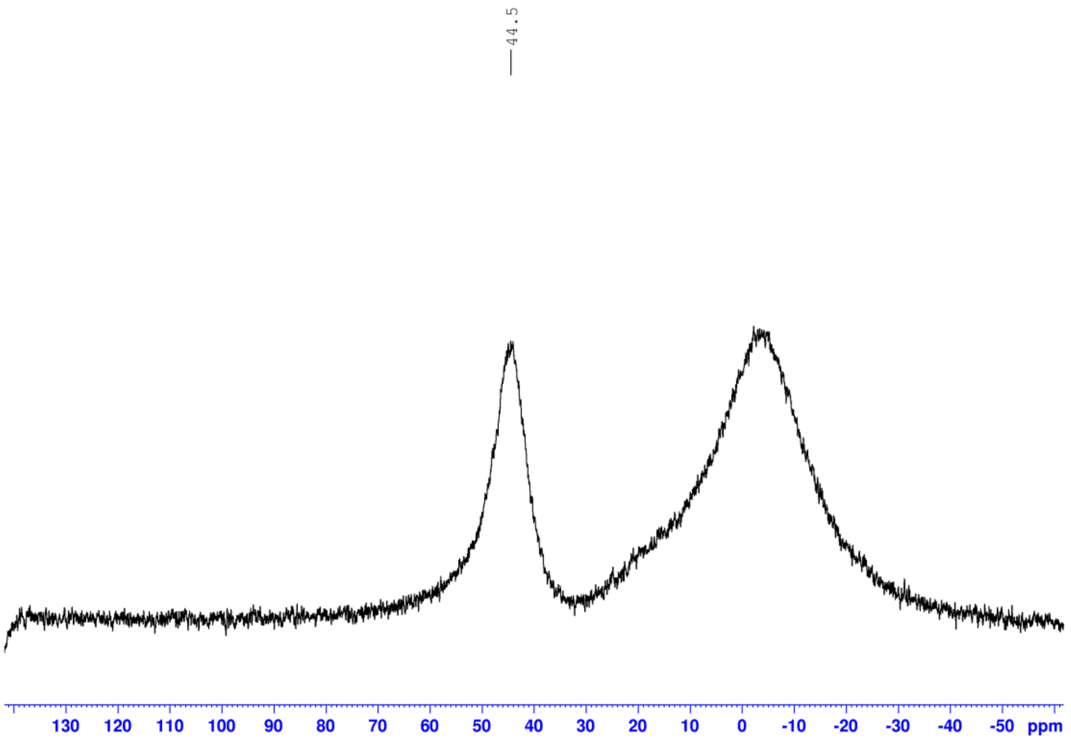

Figure S39: ^{11}B-NMR (160MHz, 298K) spectrum of compound **5a** in C_6D_6.

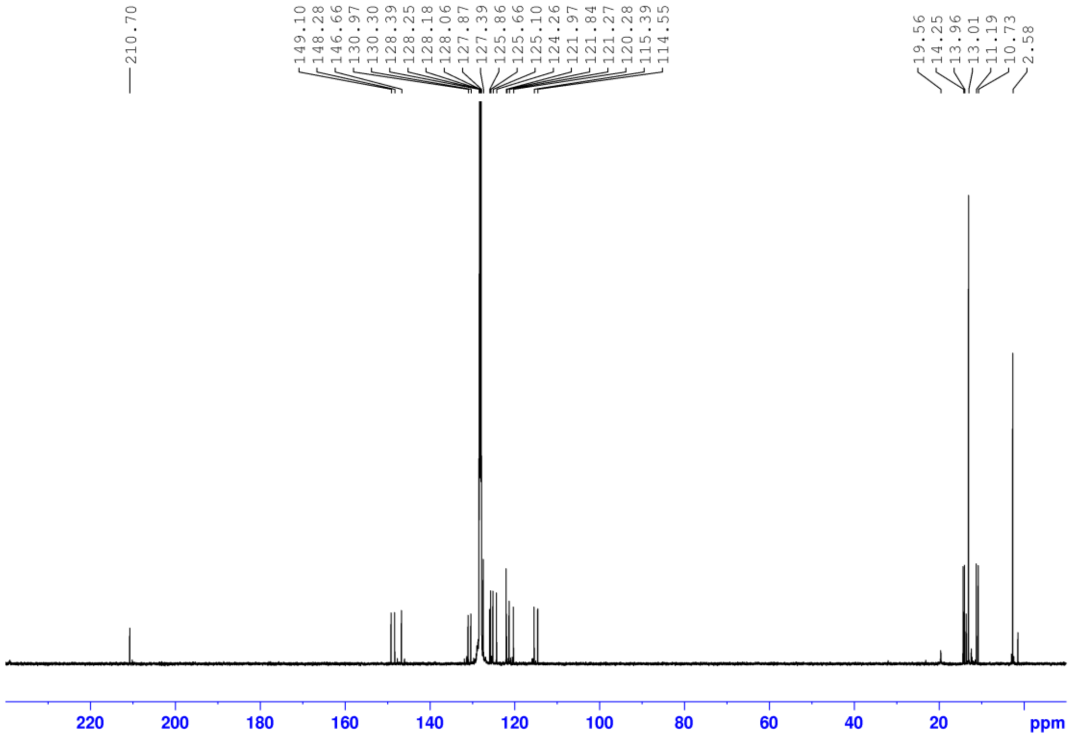

Figure S40: ^{13}C-NMR (126MHz, 298K) spectrum of compound **5a** in C_6D_6.

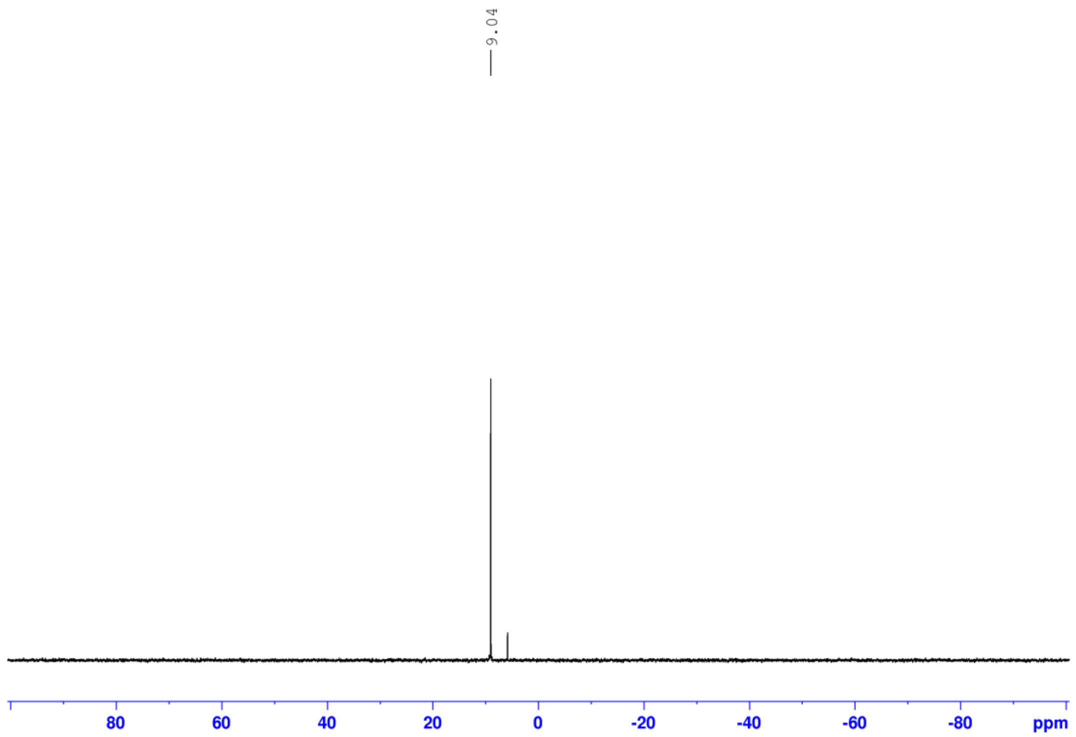

Figure S41: ^{29}Si-NMR (100MHz, 298K) spectrum of compound **5a** in C_6D_6.

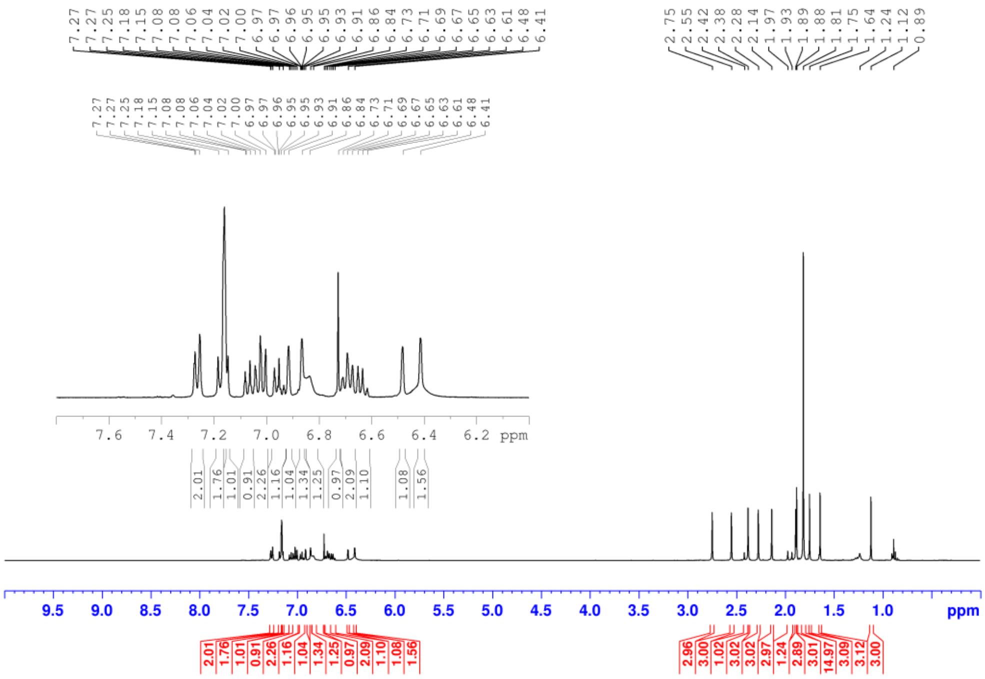

Figure S42: ^{1}H-NMR (400MHz, 298K) spectrum of compound **5b** in C_6D_6.

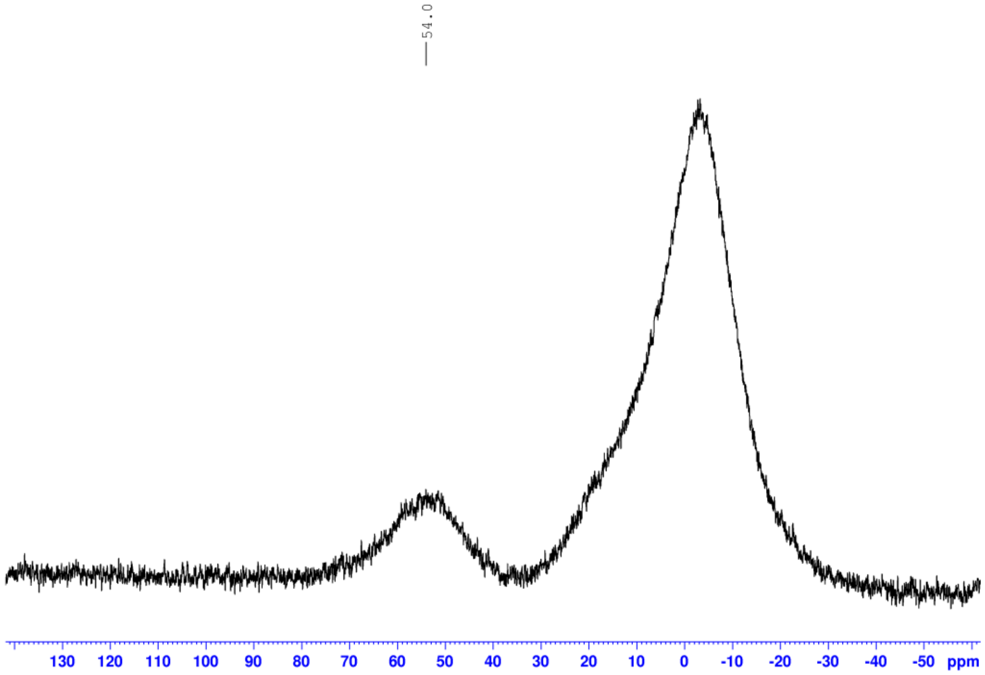

Figure S43: ^{11}B-NMR (160MHz, 298K) spectrum of compound **5b** in C_6D_6.

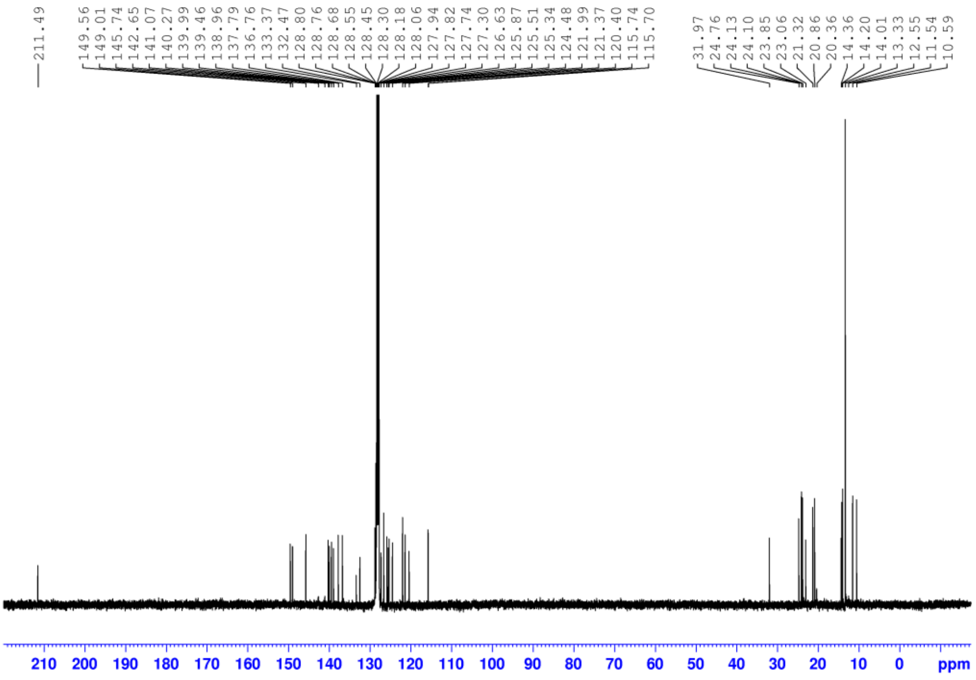

Figure S44: ^{13}C-NMR (126MHz, 298K) spectrum of compound **5b** in C_6D_6.

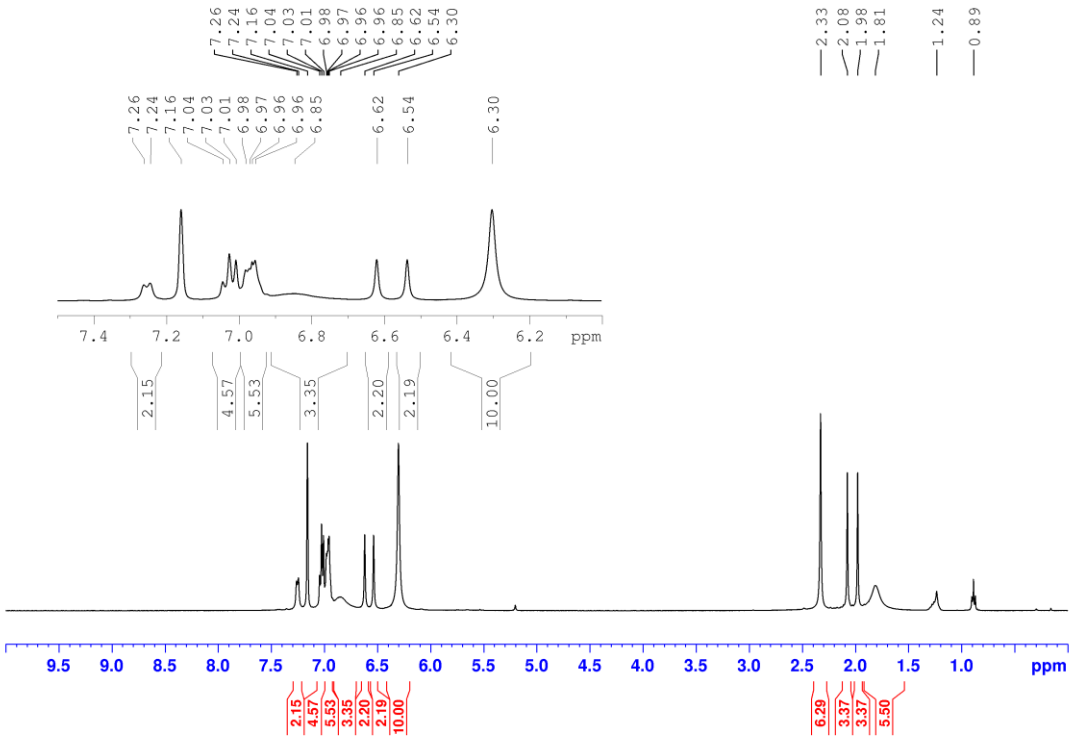

Figure S45: ^{1}H-NMR (400MHz, 298K) spectrum of compound **6h** in C_6D_6.

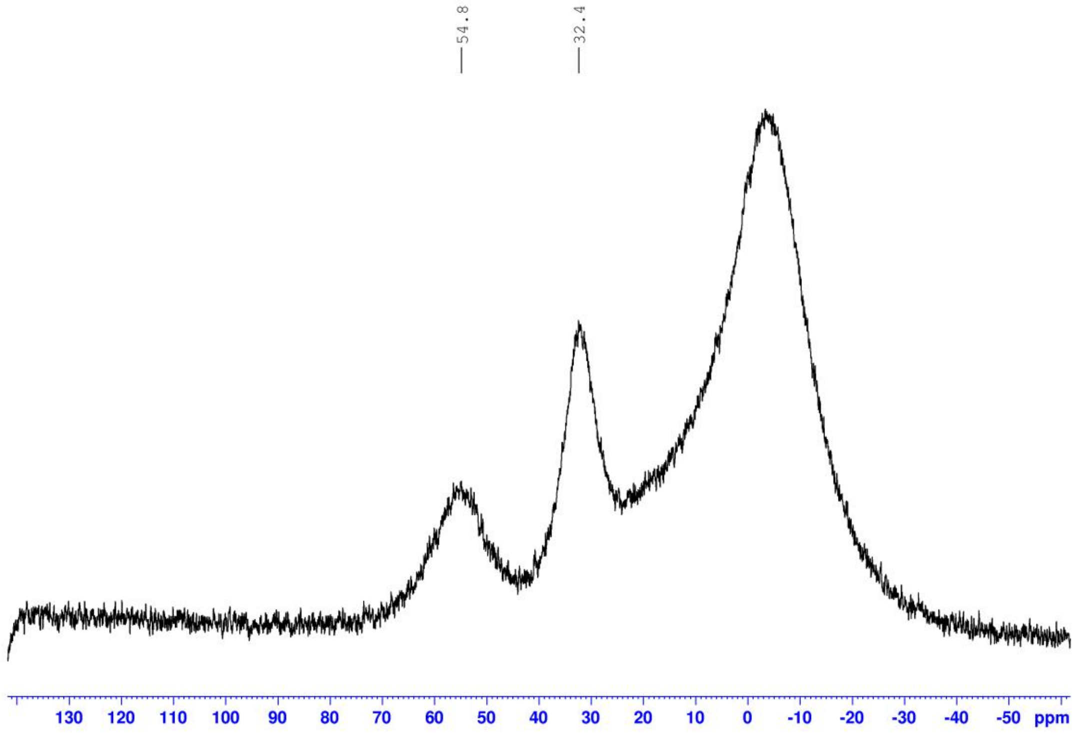

Figure S46: ^{11}B-NMR (160MHz, 298K) spectrum of compound **6h** in C_6D_6.

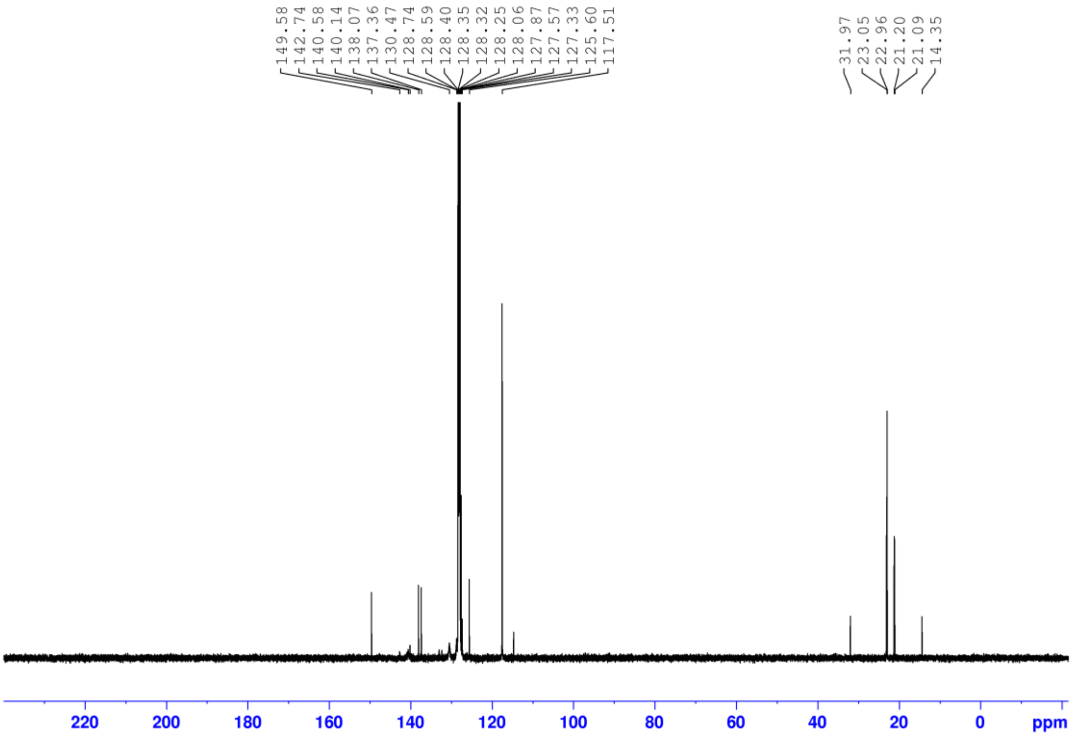

Figure S47: ^{13}C-NMR (126MHz, 298K) spectrum of compound **6h** in C_6D_6.

High resolution mass spectroscopy data

Z93b

Positive mode

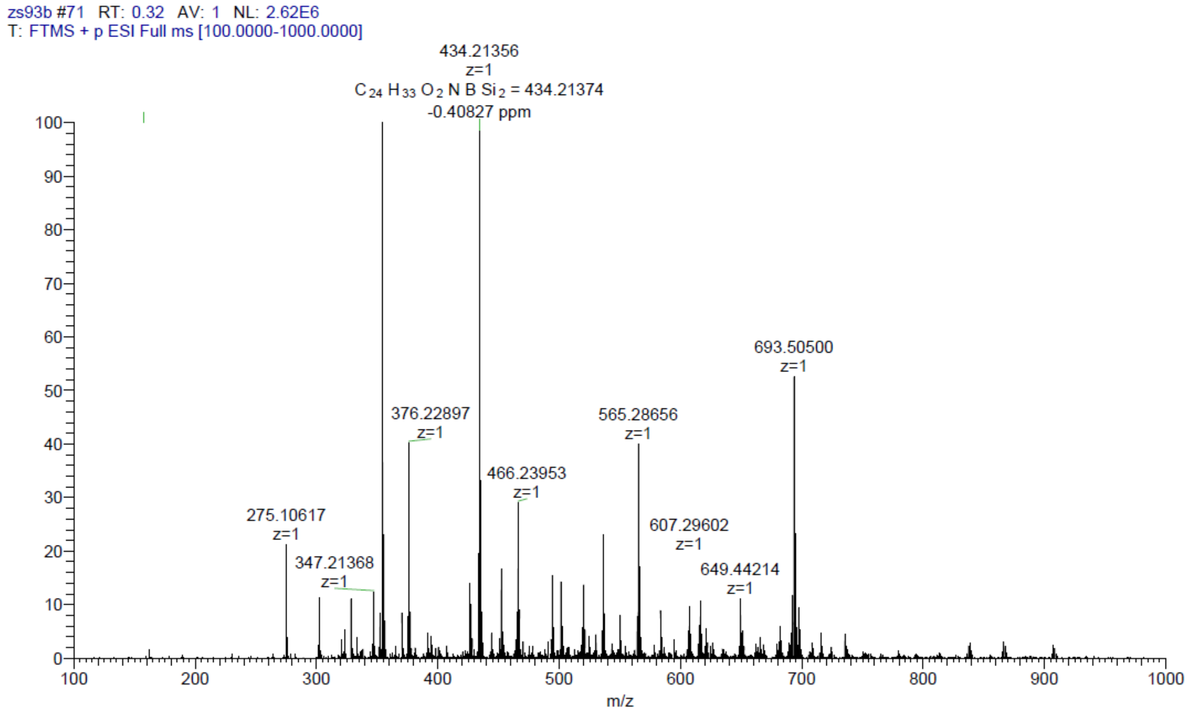

Figure S48: HRMS spectrum of compound **2c**.

[M+H]$^+$

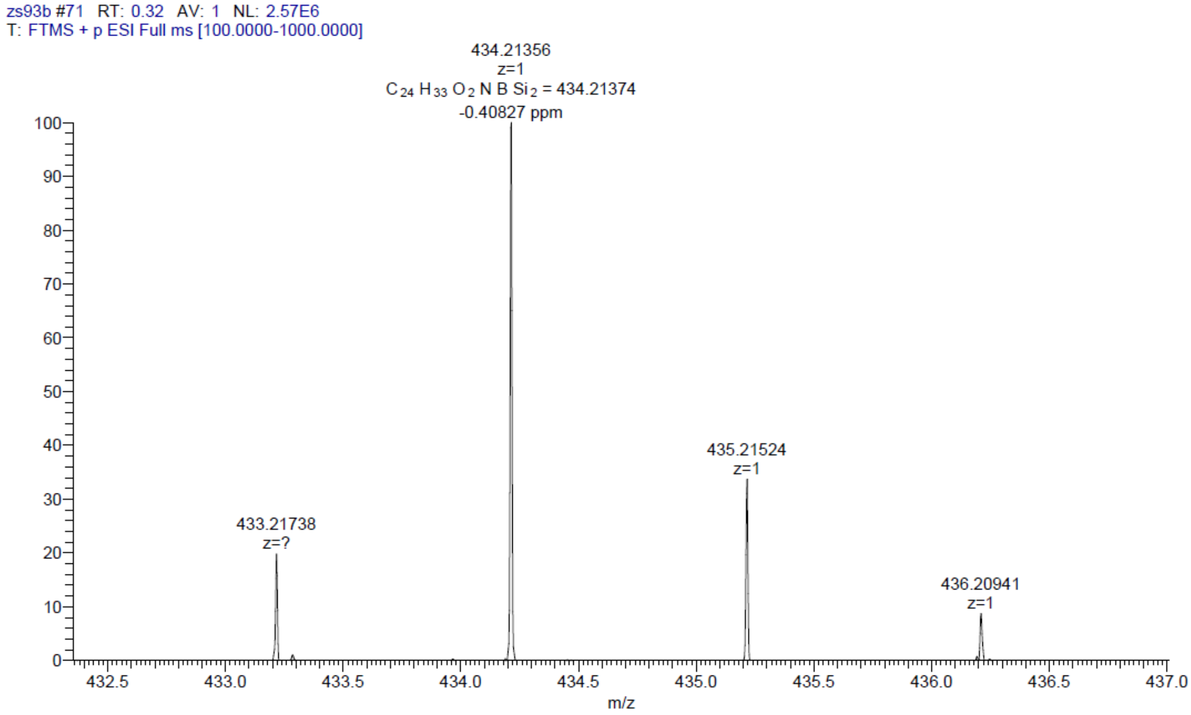

Figure S49: Zoomed in HRMS spectrum of compound **2c**.

Z93c

Positive mode

zs93c #128 RT: 0.58 AV: 1 NL: 3.19E8
T: FTMS + p ESI Full ms [100.0000-1000.0000]

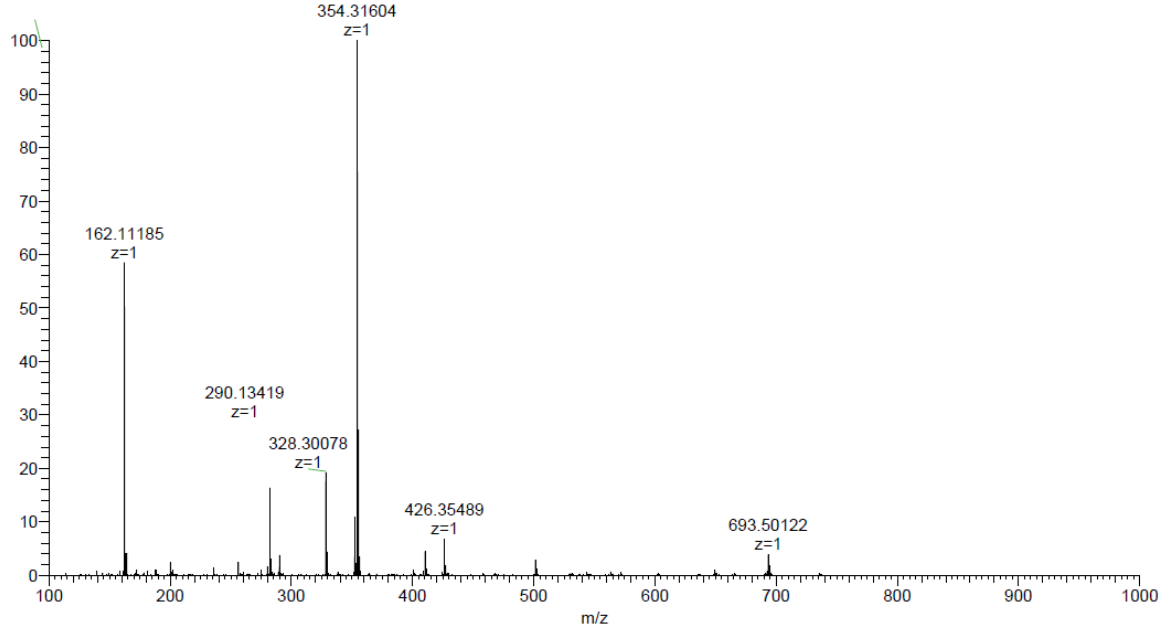

Figure S50: HRMS spectrum of compound **2d**.

[M+H]+

zs93c #128 RT: 0.58 AV: 1 NL: 1.41E7
T: FTMS + p ESI Full ms [100.0000-1000.0000]

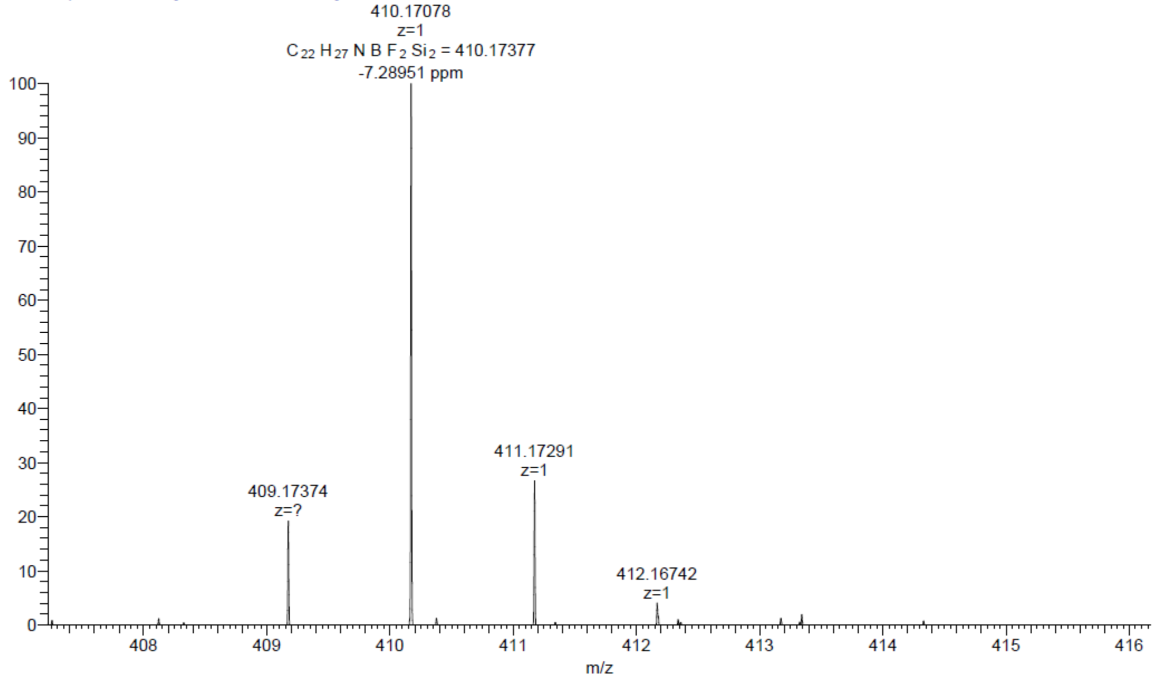

Figure S51: Zoomed in HRMS spectrum of compound **2d**.

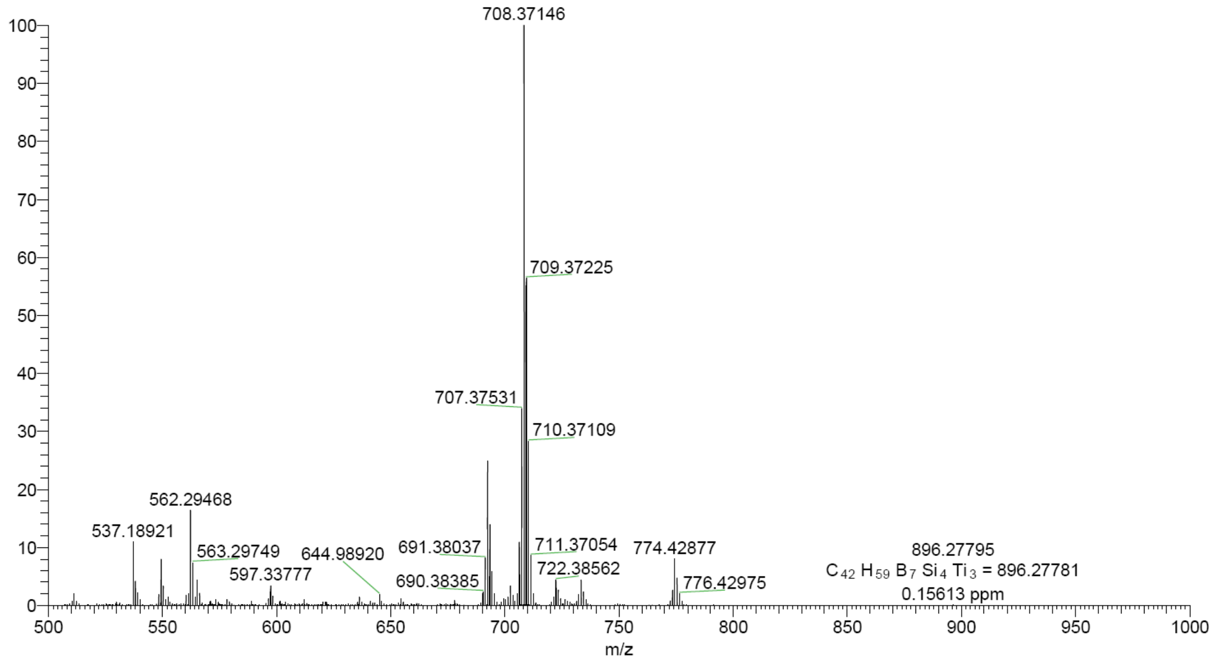

Figure S52: HRMS spectrum of compound **3a**.

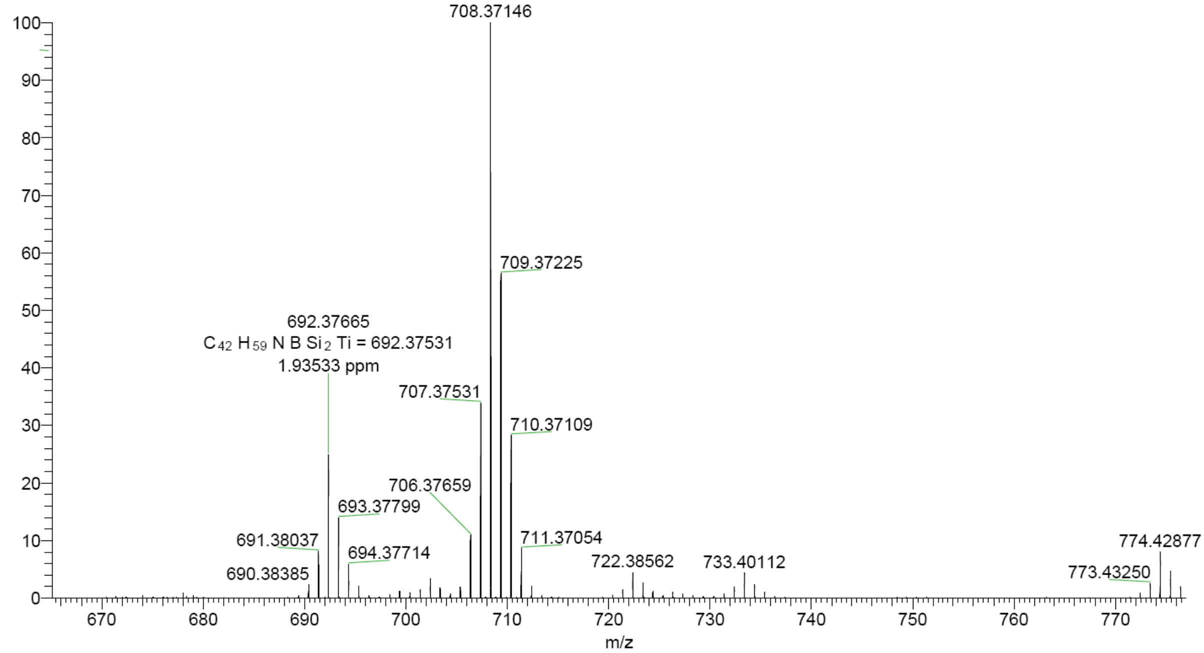

Figure S53: Zoomed in HRMS spectrum of compound **3a**.

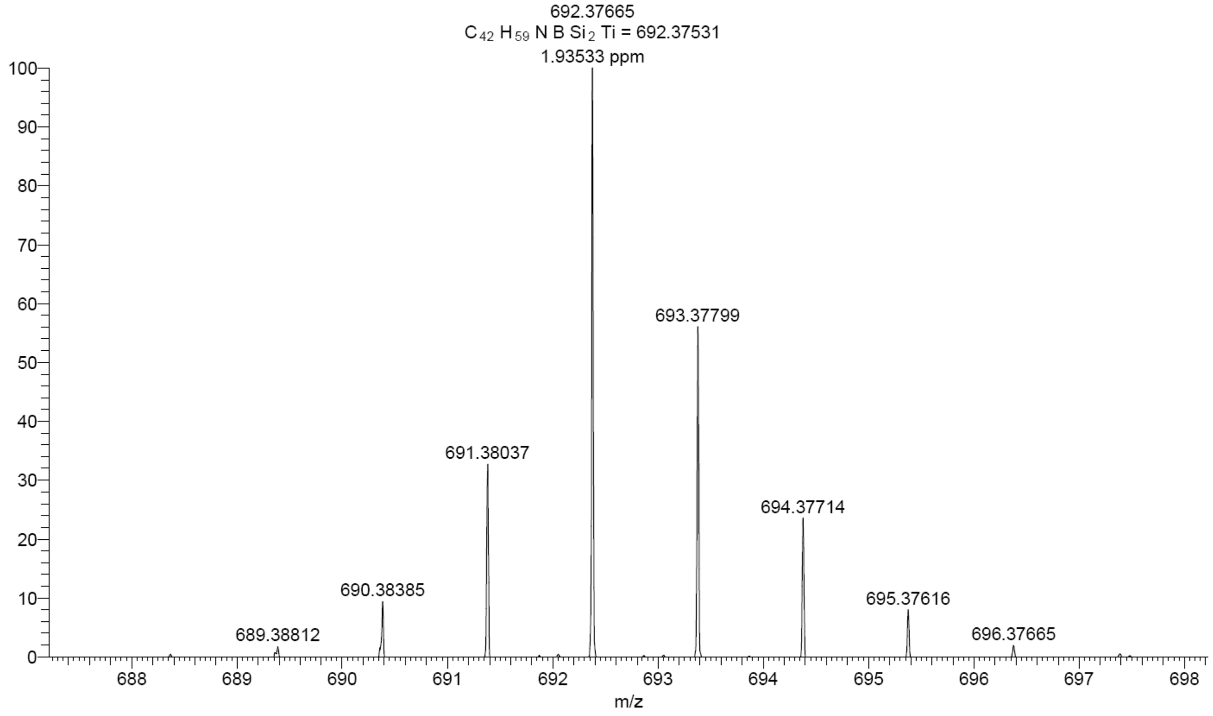

Figure S54: Zoomed in HRMS spectrum of compound **3a**.

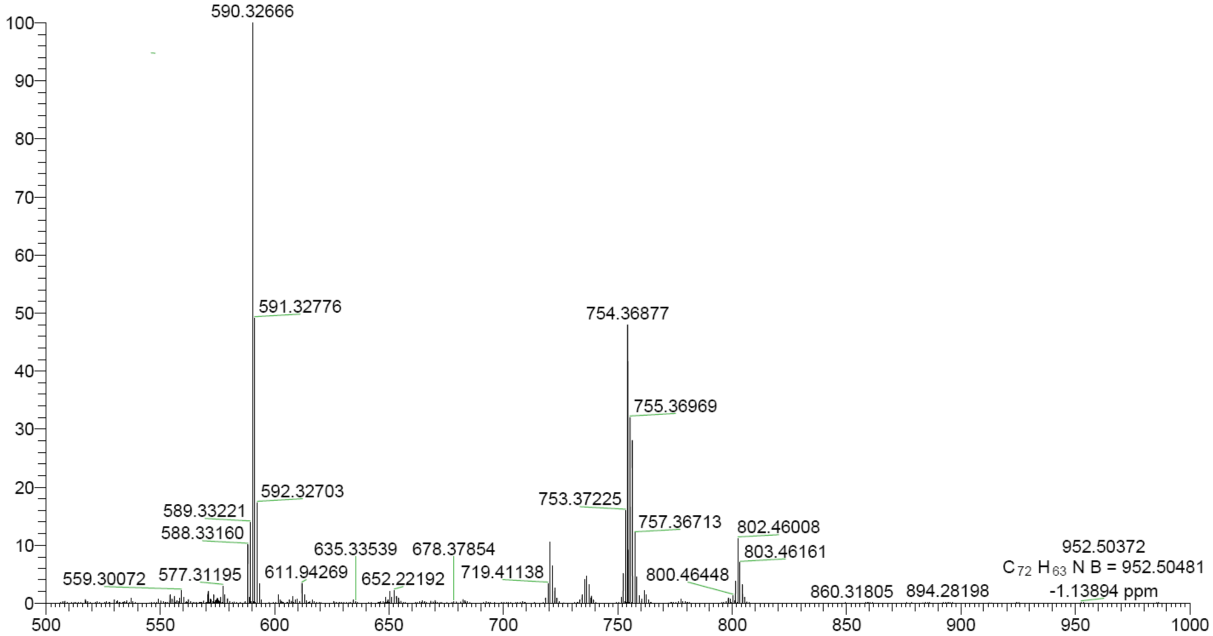

Figure S55: HRMS spectrum of compound **3b**.

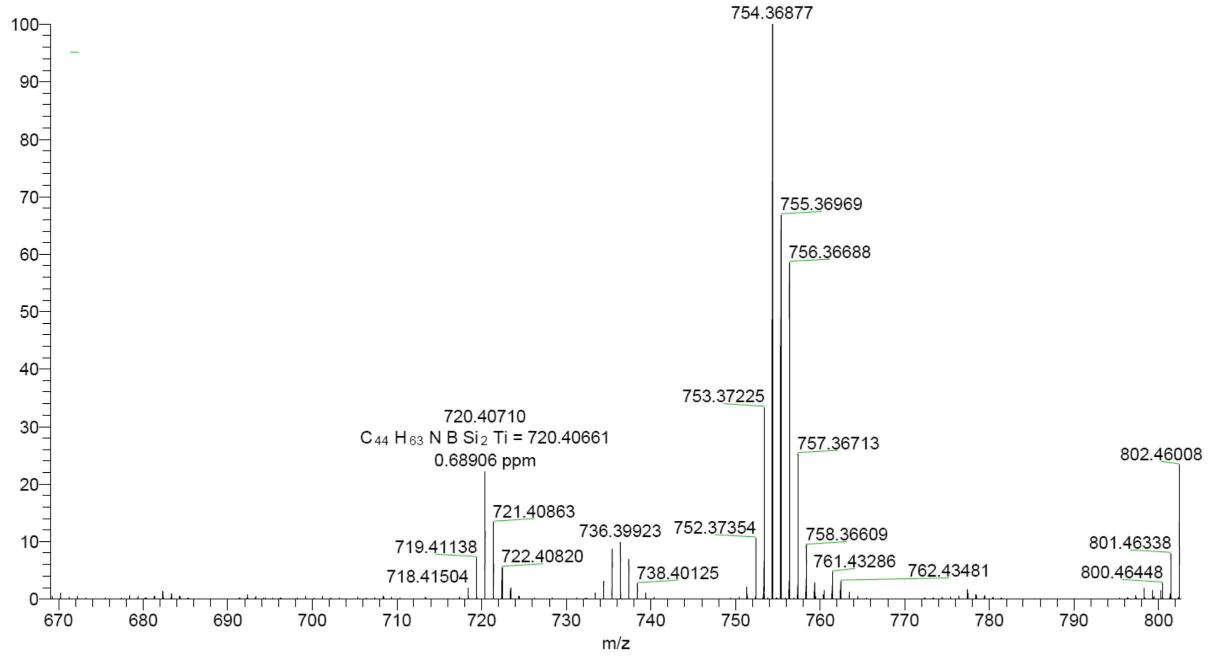

Figure S56: Zoomed in HRMS spectrum of compound **3b**.

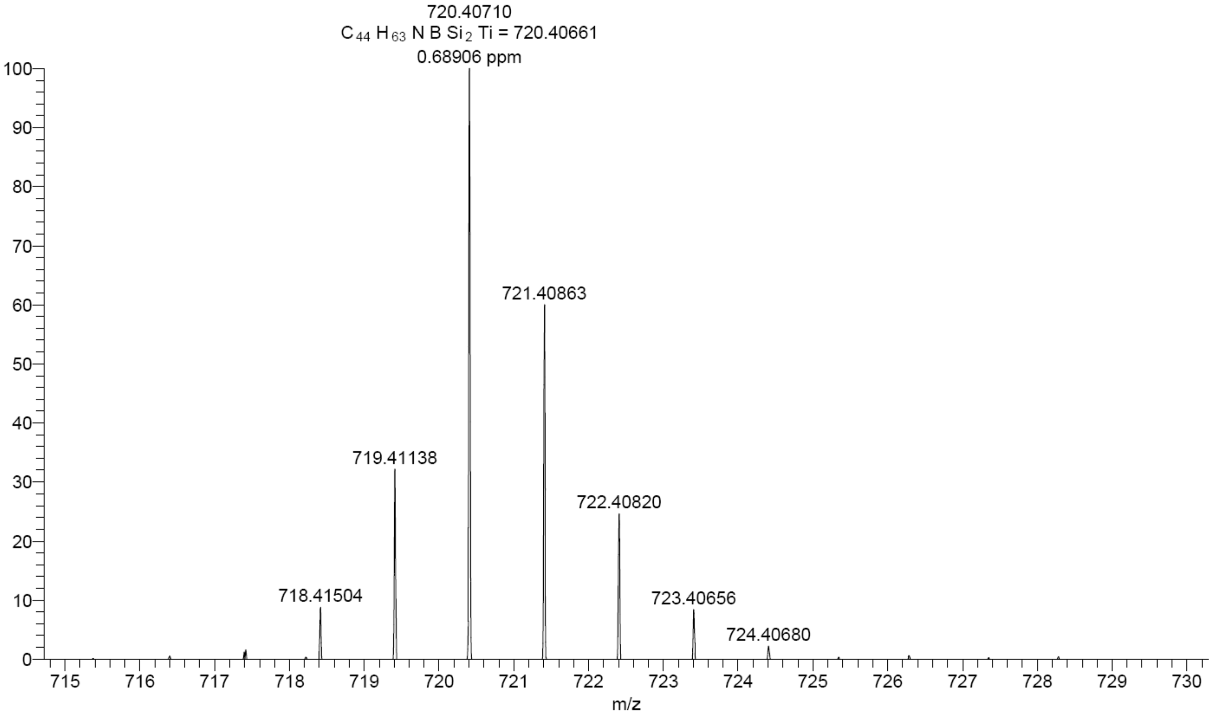

Figure S57: Zoomed in HRMS spectrum of compound **3b**.

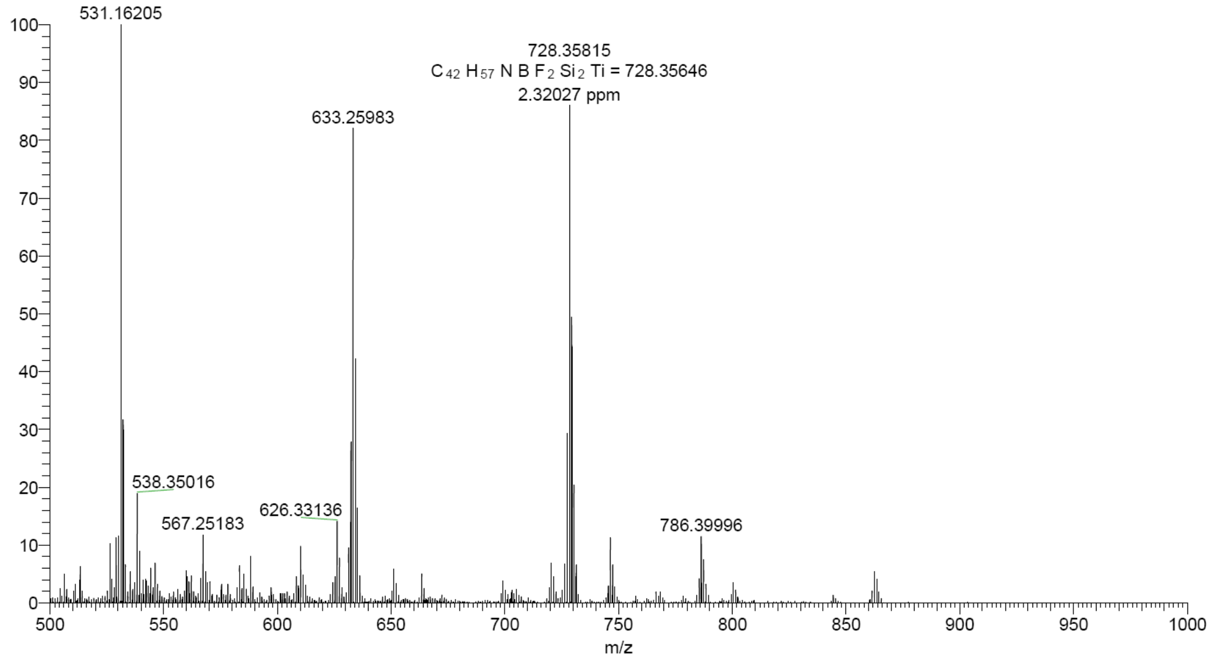

Figure S58: HRMS spectrum of compound **3d**.

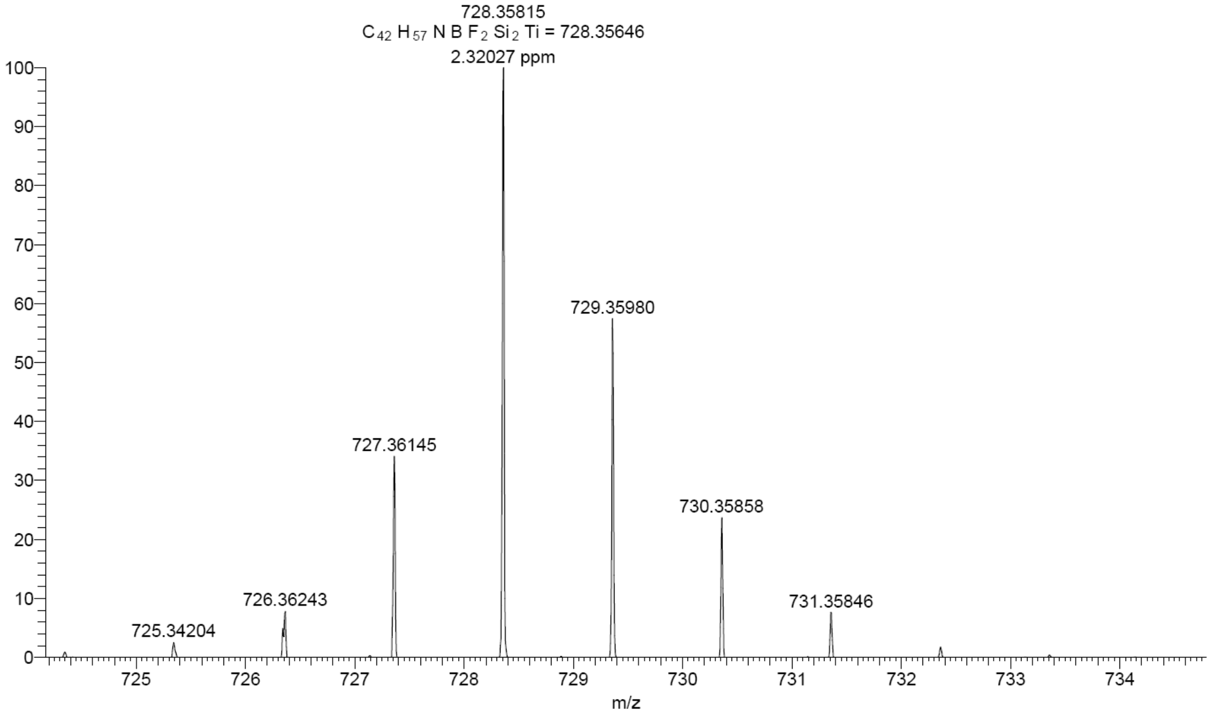

Figure S59: Zoomed in HRMS spectrum of compound **3d**.

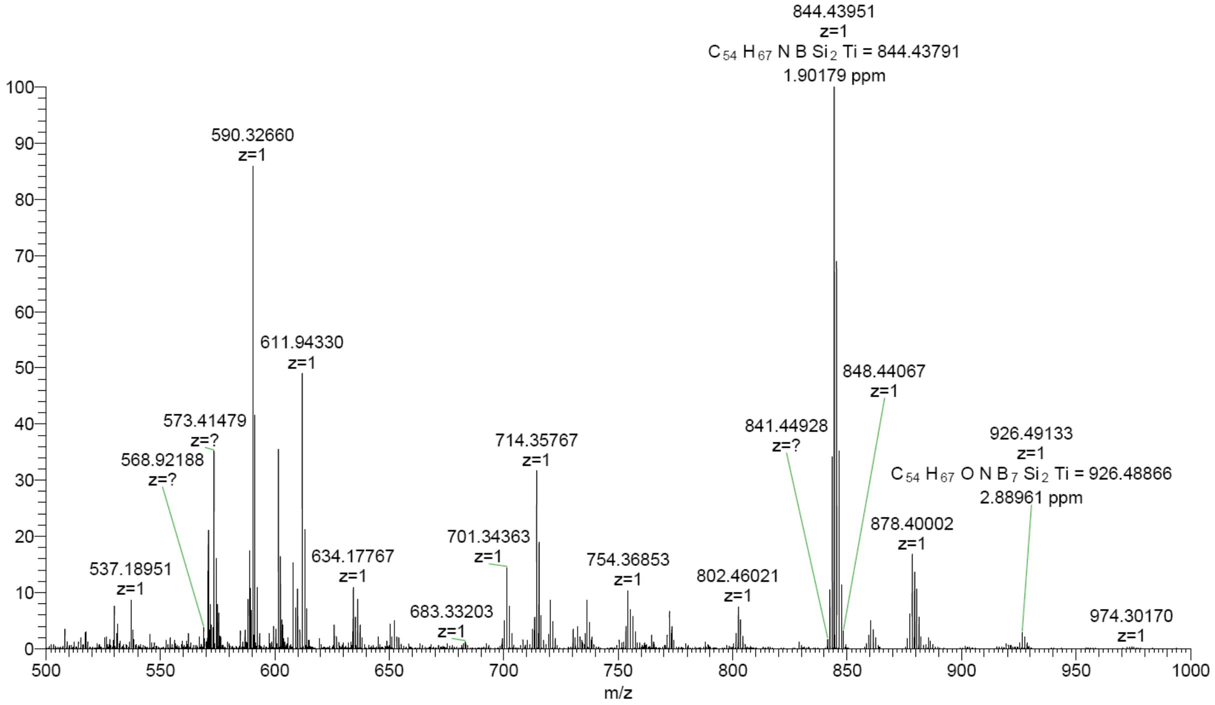

Figure S60: HRMS spectrum of compound **3e**.

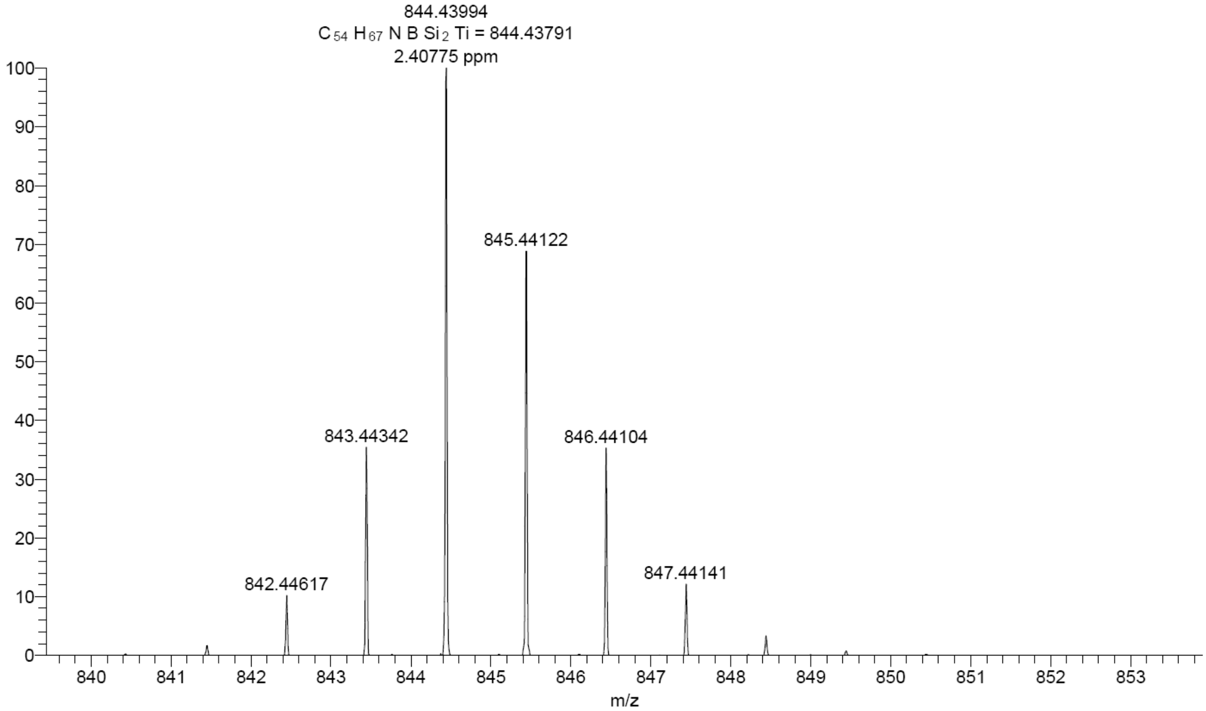

Figure S61: Zoomed in HRMS spectrum of compound **3e**.

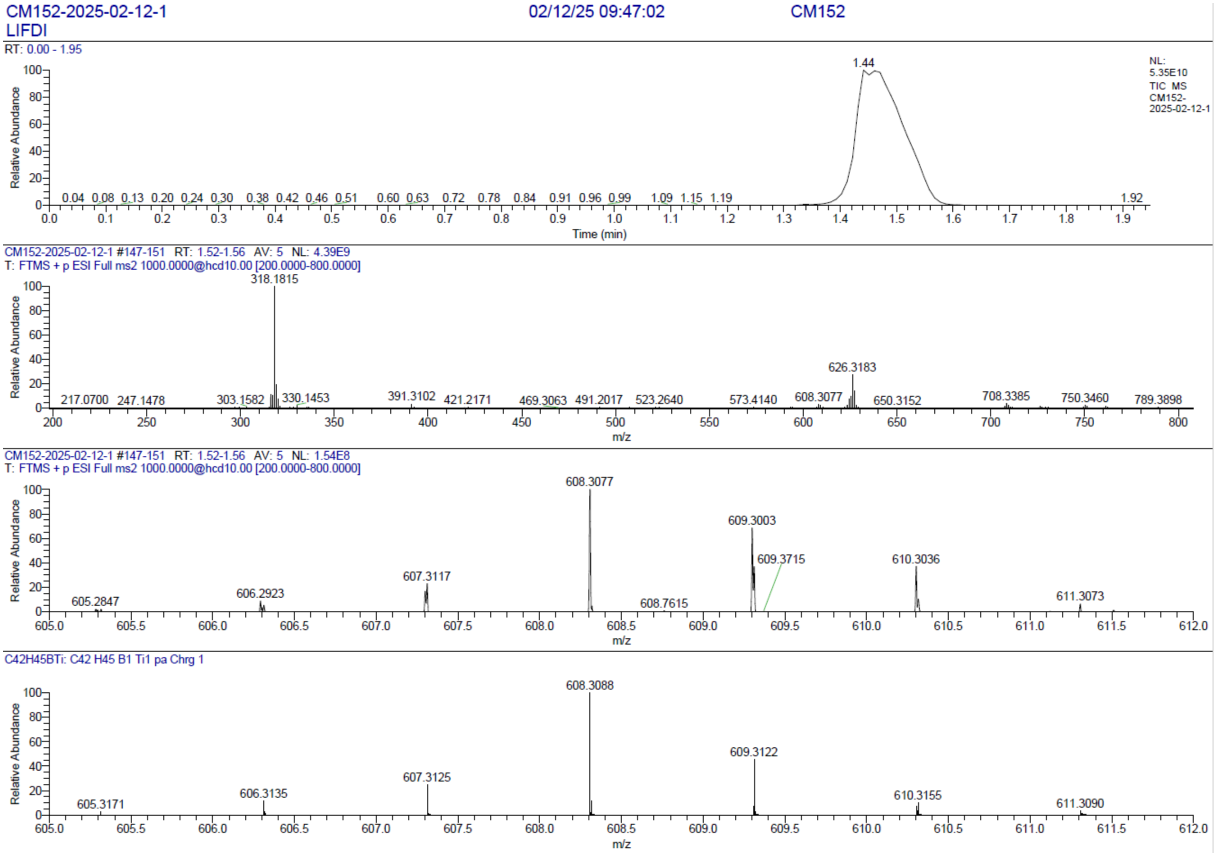

Figure S62: HRMS spectrum of compound **3f**.

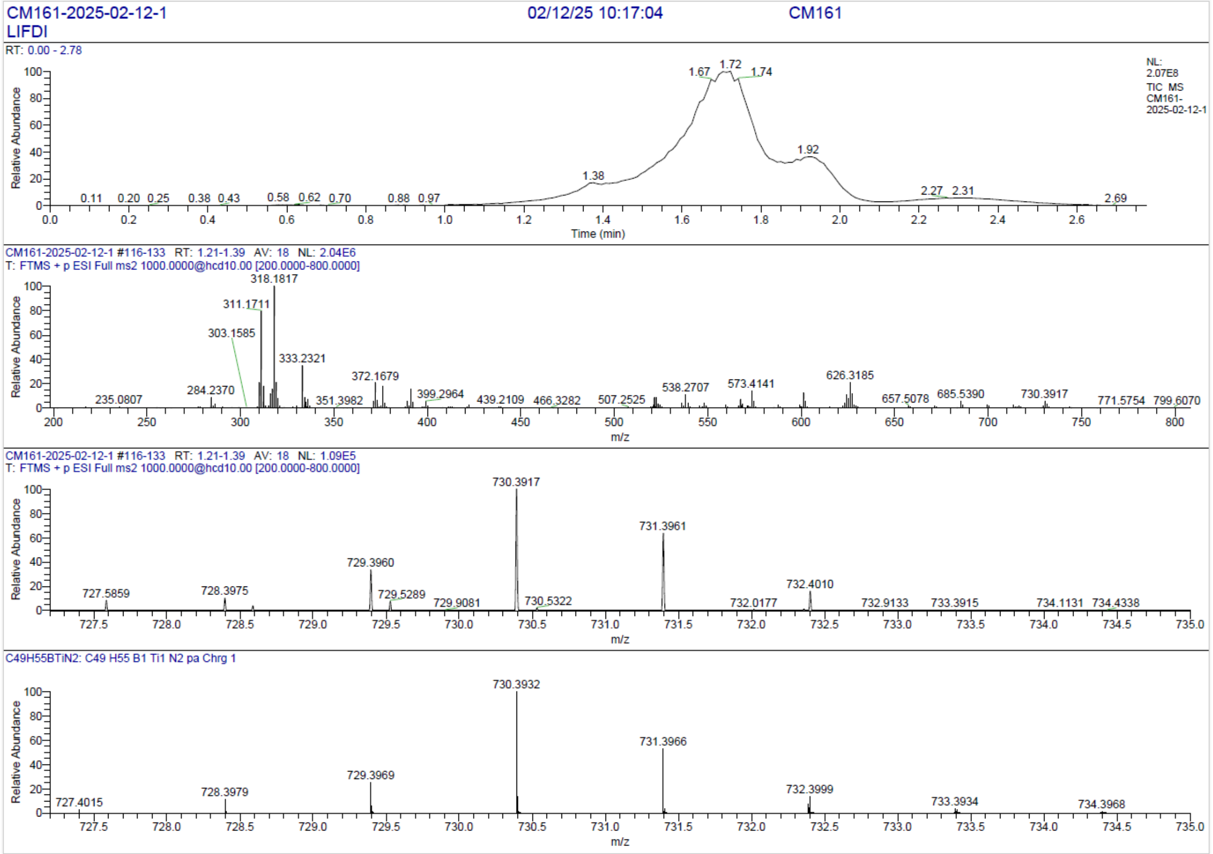

Figure S63: HRMS spectrum of compound **3f-DMAP**.

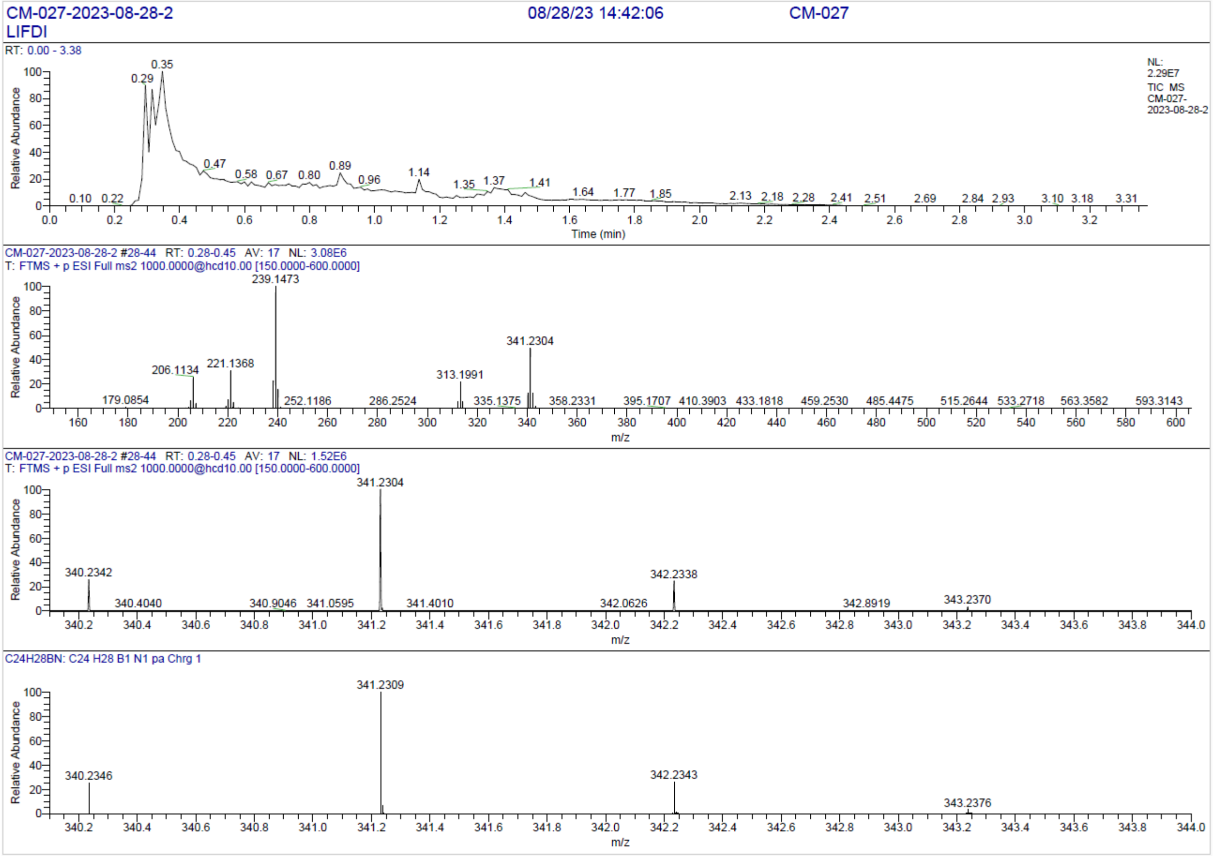

Figure S64: HRMS spectrum of compound **4b**.

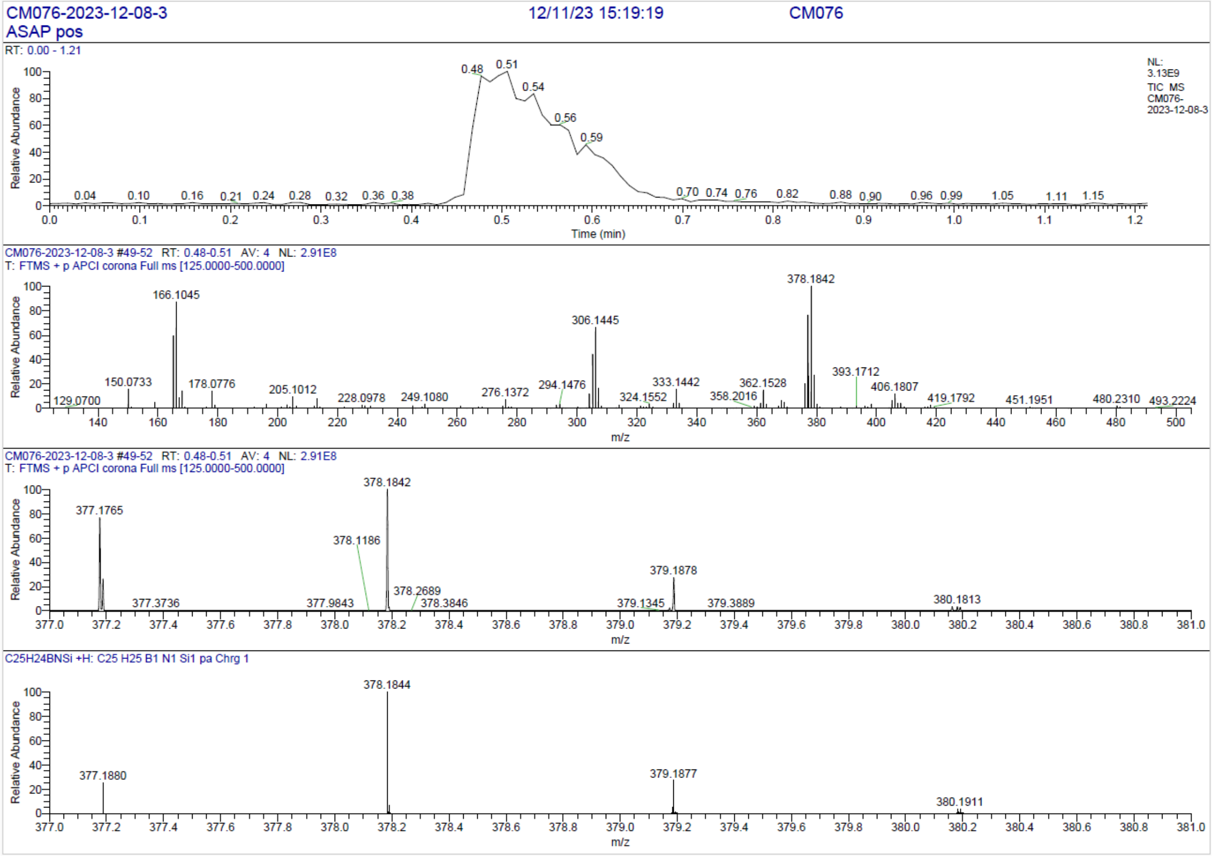

Figure S65: HRMS spectrum of compound **2g**.

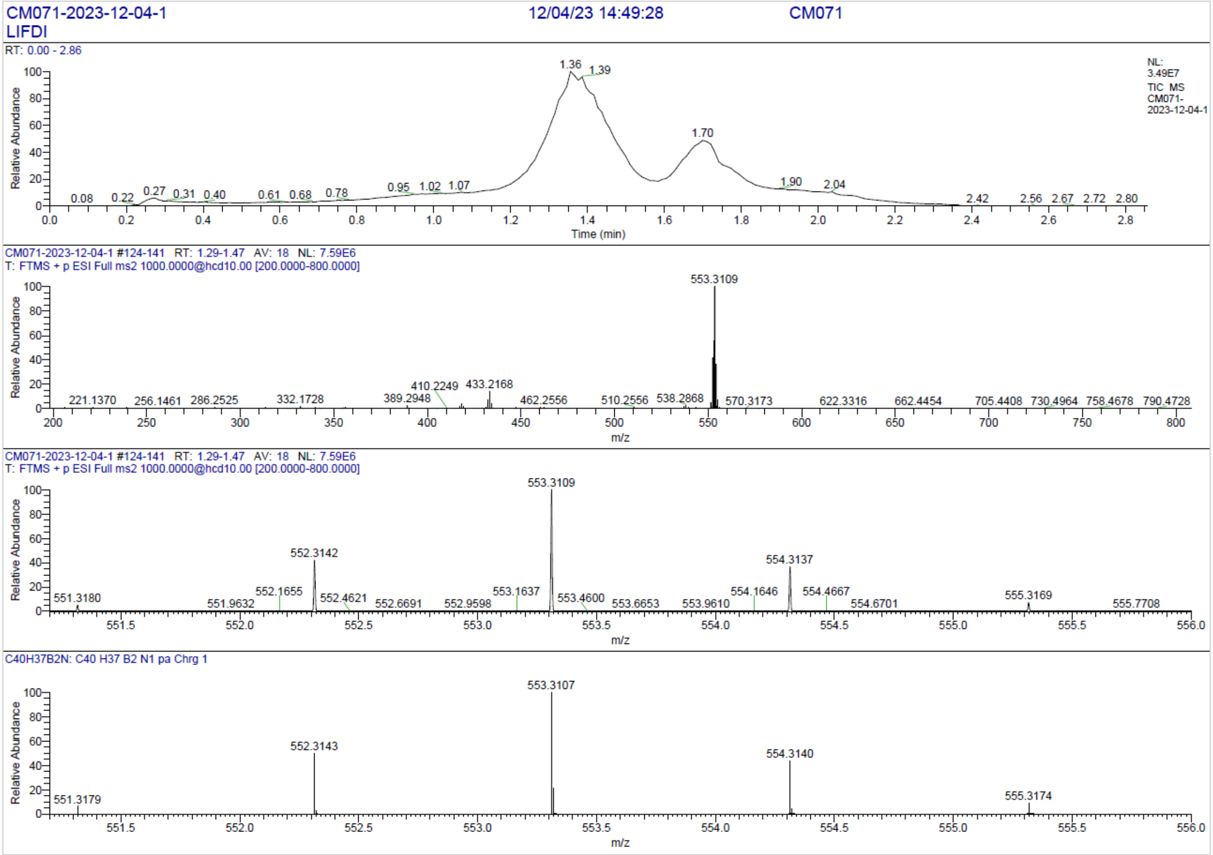

Figure S66: HRMS spectrum of compound **2h**.

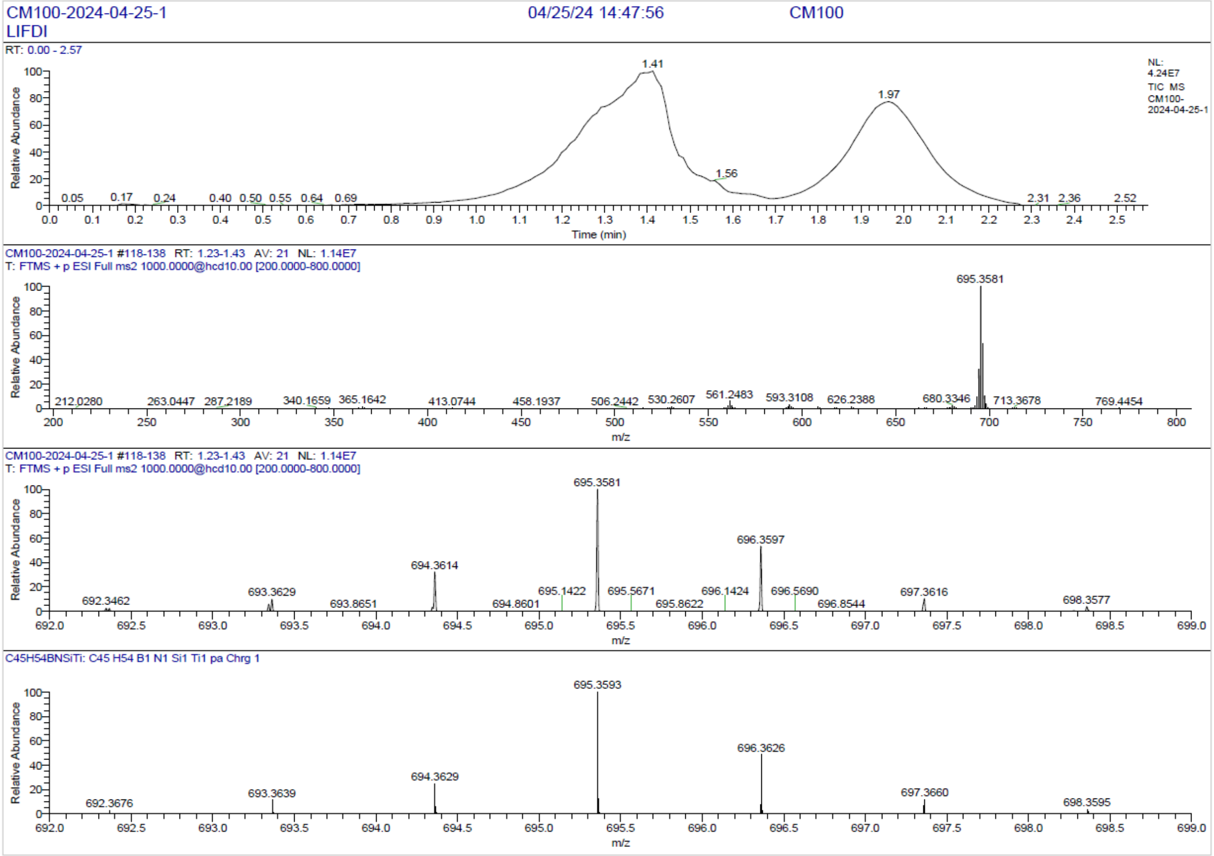

Figure S67: HRMS spectrum of compound **5a**.

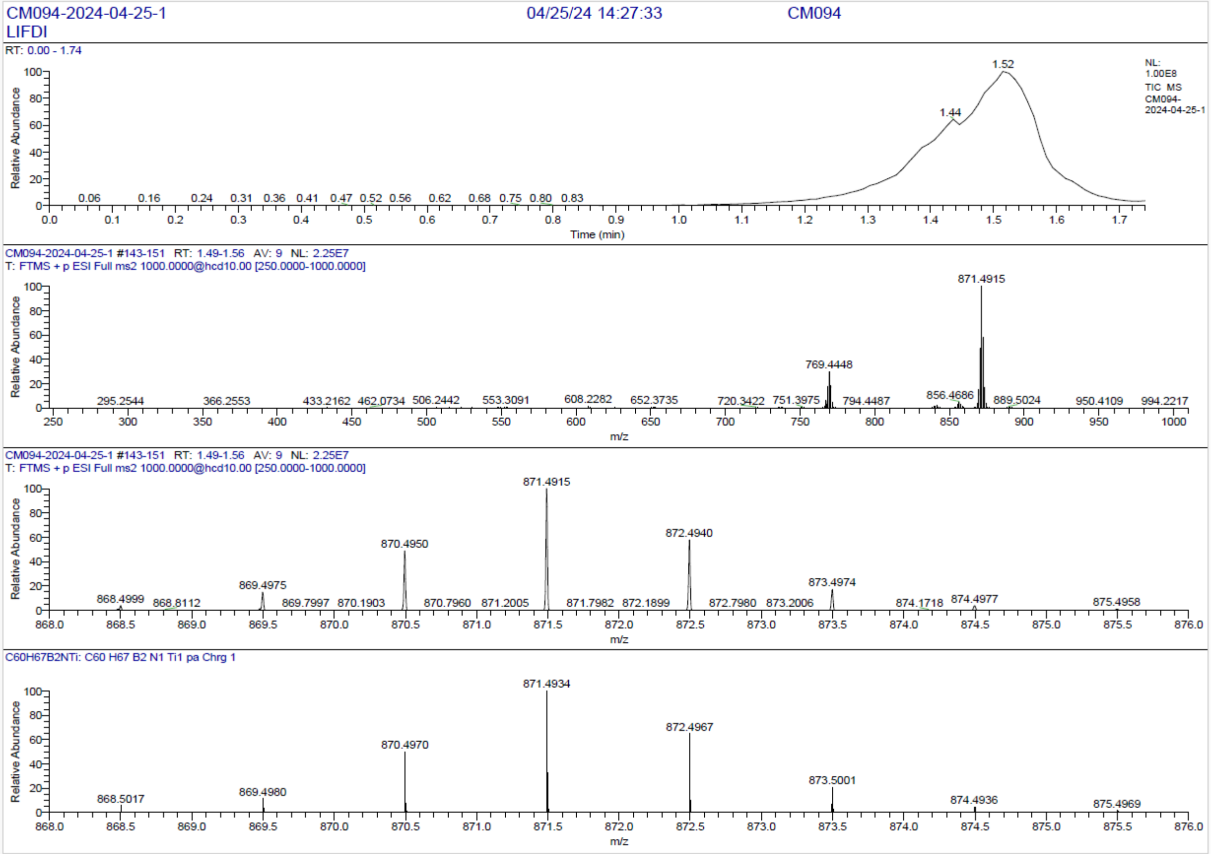

Figure S68: HRMS spectrum of compound **5b**.

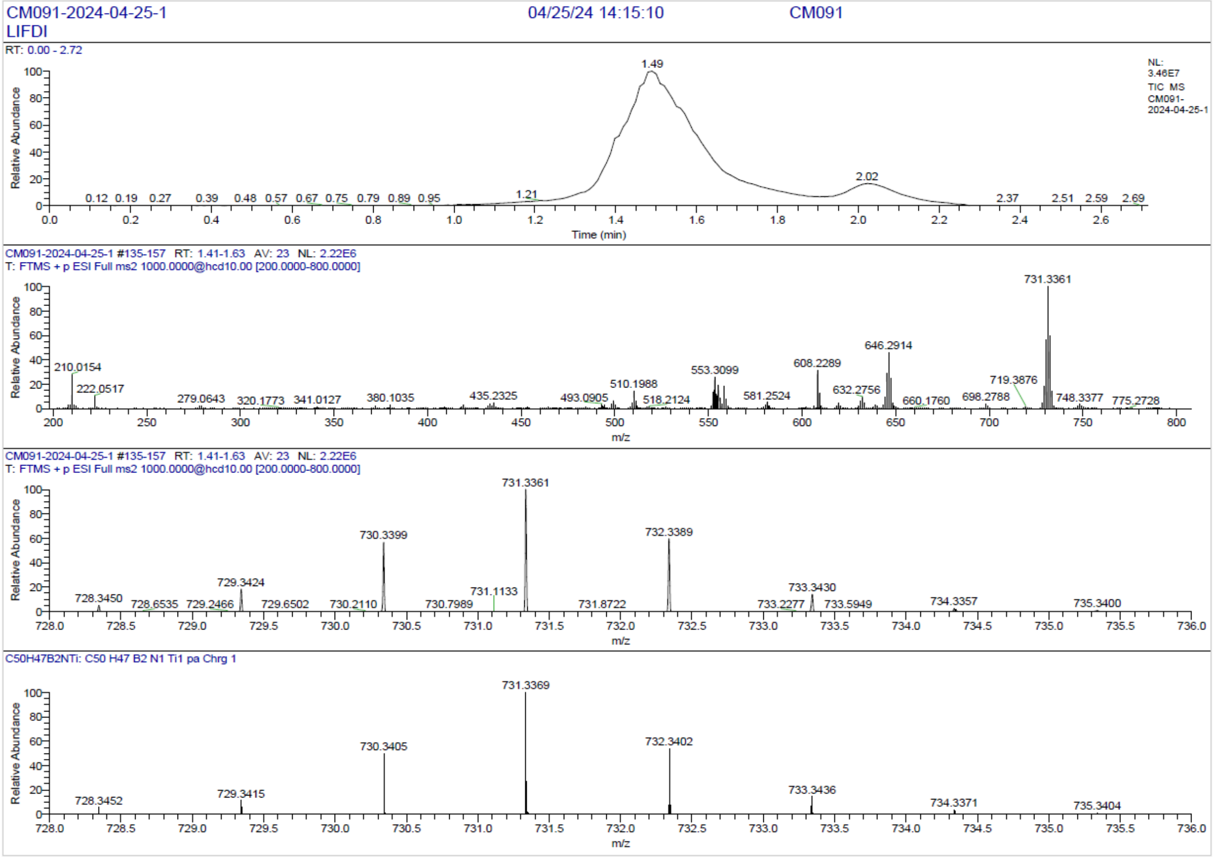

Figure S69: HRMS spectrum of compound **6h**.

Crystallographic Details

The crystal data of **3b-3e** was collected on a Bruker D8 VENTURE diffractometer with graphite monochromated $Mo_{K\alpha}$ radiation (λ = 0.71073 Å). Data reduction, scaling and absorption corrections were performed using SAINT (Bruker, V8.38A, 2013). The structure was solved with the XT structure solution program using the Intrinsic Phasing solution method[5] and by using Olex2[6] as the graphical interface. The model was refined with the ShelXL program[7] using Least Squares minimization. All non-hydrogen atoms were refined anisotropically. Hydrogen atoms were included in structure factor calculations. All hydrogen atoms were assigned to idealized geometric positions. The crystal data of **2h, 3a, 3f, 3f-DMAP, 4b, 5a, 5b and 6h** were collected on a Rigaku XtaLAB Synergy-R diffractometer with a HPA area detector and multi-layer mirror monochromated $Cu_{K\alpha}$ radiation (λ = 1.54184 Å). The structure was solved using intrinsic phasing method5, refined with the ShelXL program[7] and expanded using Fourier techniques. Crystallographic data have been deposited with the Cambridge Crystallographic Data as supplementary publication nos. CCDC-2423409 (**3a**), CCDC-2423410 (**3a-int**), CCDC-2423411 (**3b**), CCDC-2423412 (**3c**), CCDC-2423413 (**3d**), CCDC-2423414 (**3e**), CCDC-2423415 (**3f**), CCDC-2423416 (**3f-DMAP**), CCDC-2423417 (**4b**), CCDC-2423418 (**2h**), CCDC-2423419 (**5a**), CCDC-2423420 (**5b**), CCDC-2423421 (**6h**).

These data can be obtained free of charge from The Cambridge Crystallographic Data Centre via Data https://www.ccdc.cam.ac.uk

Details of the data collection and refinement for compounds **2h**, **3a–3f**, **3a-int**, **3-DMAP**, **4b, 5a-b** and **6h** are given in **Table 1–13**.

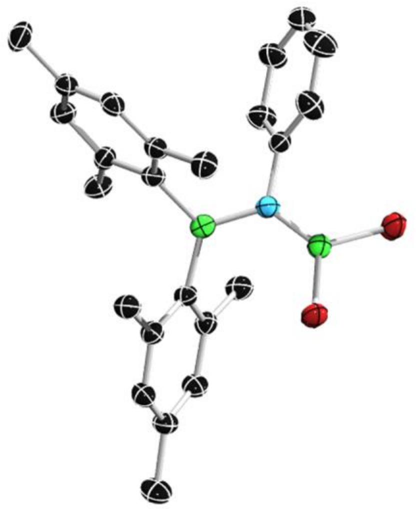

Figure S70. Single crystal structure of **4b**. (Some ellipsoids and hydrogen atoms have been removed for clarity.)

Table S1: Crystal data and structure refinement for **3a**.

Data	3a
Empirical formula	$C_{42}H_{58}BNSi_2Ti$
Formula weight (g·mol^{-1})	691.78
Temperature (K)	100(2)
Radiation, *l* (Å)	Cu$_{Ka}$, 1.54184
Crystal system	monoclinic
Space group	$P2_1/n$
Unit cell dimensions	
a (Å)	13.92040(10)
b (Å)	15.62270(10)
c (Å)	17.70590(10)
a (°)	90
b (°)	91.6330(10)
g (°)	90
Volume (Å^3)	3849.01(4)
Z	4
Calculated density (Mg·m^{-3})	1.194
Absorption coefficient (mm^{-1})	2.688
F(000)	1488
Theta range for collection	3.774 to 74.887°
Reflections collected	41858
Unique reflections	7702
Unique reflections with [I>2s(I)]	7309
Minimum/maximum transmission	0.402/1.000
Refinement method	Full-matrix least-squares on F^2
Data / parameters / restraints	7702 / 440 / 0
Goodness-of-fit on F^2	1.065
Final *R* indices [*I*>2*s*(*I*)]	R_1 = 0.0316, wR_2 = 0.0819
R indices (all data)	R_1 = 0.0332, wR_2 = 0.0830
Maximum/minimum residual electron density (e·Å^{-3})	0.397 / –0.429

Table S2: Crystal data and structure refinement for **3a-int**

Data	3a-int
Empirical formula	$C_{42}H_{58}BNSi_2Ti$
Formula weight (g·mol^{-1})	691.78
Temperature (K)	100(2)
Radiation, *l* (Å)	Cu_{Ka}, 1.54184
Crystal system	triclinic
Space group	$P\ \overline{1}$
Unit cell dimensions	
a (Å)	9.4394(2)
b (Å)	12.1805(3)
c (Å)	17.9860(4)
a (°)	86.645(2)
b (°)	85.408(2)
g (°)	75.892(2)
Volume (Å^3)	1997.53(8)
Z	2
Calculated density (Mg·m^{-3})	1.150
Absorption coefficient (mm^{-1})	2.590
F(000)	744
Theta range for collection	2.467 to 75.377°
Reflections collected	37652
Unique reflections	7906
Unique reflections with [I>2s(I)]	6678
Minimum/maximum transmission	0.803/1.000
Refinement method	Full-matrix least-squares on F^2
Data / parameters / restraints	7906 / 513 / 396
Goodness-of-fit on F^2	1.031
Final *R* indices [*I*>2*s*(*I*)]	R_1 = 0.0649, wR_2 = 0.1729
R indices (all data)	R_1 = 0.0738, wR_2 = 0.1787
Maximum/minimum residual electron density (e·Å^{-3})	0.668 / –0.702

Table S3: Crystal data and structure refinement for **3b**

Data	3b
Empirical formula	$C_{44}H_{62}BNSi_2Ti$
Formula weight (g·mol^{-1})	719.83
Temperature (K)	100(2)
Radiation, *l* (Å)	Mo_{Ka}, 0.71073
Crystal system	triclinic
Space group	$P\bar{1}$
Unit cell dimensions	
a (Å)	11.2086(13)
b (Å)	11.2647(14)
c (Å)	17.463(2)
a (°)	82.067(4)
b (°)	82.090(4)
g (°)	74.465(4)
Volume (Å^3)	2092.3(4)
Z	2
Calculated density (Mg·m^{-3})	1.143
Absorption coefficient (mm^{-1})	0.292
F(000)	776
Theta range for collection	2.311 to 24.827°
Reflections collected	38656
Unique reflections	7181
Unique reflections with [I>2s(I)]	4949
Minimum/maximum transmission	0.5402/0.7451
Refinement method	Full-matrix least-squares on F^2
Data / parameters / restraints	7181 / 616 / 175
Goodness-of-fit on F^2	1.038
Final *R* indices [*I*>2*s*(*I*)]	R_1 = 0.0656, wR_2 = 0.1700
R indices (all data)	R_1 = 0.0969, wR_2 = 0.1913
Maximum/minimum residual electron density (e·Å^{-3})	0.691 / –0.595

Table S4: Crystal data and structure refinement for **3c**

Data	3c
Empirical formula	$C_{44}H_{62}BNO_2Si_2Ti$
Formula weight (g·mol^{-1})	751.83
Temperature (K)	100(2)
Radiation, *l* (Å)	Mo_{Ka}, 0.71073
Crystal system	monoclinic
Space group	*Cc*
Unit cell dimensions	
a (Å)	17.627(2)
b (Å)	13.7569(14)
c (Å)	17.2138(18)
a (°)	90
b (°)	99.156(4)
g (°)	90
Volume (Å^3)	4121.0(8)
Z	4
Calculated density (Mg·m^{-3})	1.212
Absorption coefficient (mm^{-1})	0.302
F(000)	1616
Theta range for collection	2.333 to 24.746°
Reflections collected	44722
Unique reflections	6938
Unique reflections with [I>2s(I)]	5606
Minimum/maximum transmission	0.6408/0.7451
Refinement method	Full-matrix least-squares on F^2
Data / parameters / restraints	6938 / 479 / 815
Goodness-of-fit on F^2	1.050
Final *R* indices [*I*>2*s*(*I*)]	R_1 = 0.0769, wR_2 = 0.1567
R indices (all data)	R_1 = 0.1054, wR_2 = 0.1808
Maximum/minimum residual electron density (e·Å^{-3})	1.224 / –0.637
Flack parameter	0.40(10)

Table S5: Crystal data and structure refinement for **3d**

Data	3d
Empirical formula	$C_{42}H_{56}BF_2NSi_2Ti$
Formula weight (g·mol–1)	727.76
Temperature (K)	100(2)
Radiation, *l* (Å)	Mo_{Ka}, 0.71073
Crystal system	triclinic
Space group	$P\bar{1}$
Unit cell dimensions	
a (Å)	9.8690(9)
b (Å)	12.7157(15)
c (Å)	15.7213(17)
a (°)	79.850(4)
b (°)	86.970(3)
g (°)	83.932(4)
Volume ($Å^3$)	1929.9(4)
Z	2
Calculated density ($Mg·m^{-3}$)	1.252
Absorption coefficient (mm^{-1})	0.324
F(000)	776
Theta range for collection	2.269 to 24.746°
Reflections collected	14392
Unique reflections	6408
Unique reflections with [I>2s(I)]	3745
Minimum/maximum transmission	0.3860/0.7451
Refinement method	Full-matrix least-squares on F^2
Data / parameters / restraints	6408 / 574 / 859
Goodness-of-fit on F^2	1.030
Final *R* indices [*I*>2*s*(*I*)]	R_1 = 0.0771, wR_2 = 0.1596
R indices (all data)	R_1 = 0.1401, wR_2 = 0.1989
Maximum/minimum residual electron density ($e·Å^{-3}$)	0.340 / –0.761

Table S6: Crystal data and structure refinement for **3e.**

Data	3e
Empirical formula	$C_{54}H_{66}BNSi_2Ti$
Formula weight (g·mol^{-1})	843.96
Temperature (K)	100(2)
Radiation, *l* (Å)	Mo_{Ka}, 0.71073
Crystal system	triclinic
Space group	$P\bar{1}$
Unit cell dimensions	
a (Å)	11.357(6)
b (Å)	11.413(6)
c (Å)	19.719(10)
a (°)	81.323(17)
b (°)	81.350(18)
g (°)	74.726(18)
Volume (Å^3)	2421(2)
Z	2
Calculated density (Mg·m^{-3})	1.158
Absorption coefficient (mm^{-1})	0.262
F(000)	904
Theta range for collection	2.243 to 24.999°
Reflections collected	25131
Unique reflections	8514
Unique reflections with [I>2s(I)]	5701
Minimum/maximum transmission	0.5846/0.7451
Refinement method	Full-matrix least-squares on F^2
Data / parameters / restraints	8514 / 810 / 526
Goodness-of-fit on F^2	1.027
Final *R* indices [*I*>2*s*(*I*)]	R_1 = 0.0678, wR_2 = 0.1706
R indices (all data)	R_1 = 0.1064, wR_2 = 0.1912
Maximum/minimum residual electron density (e·Å^{-3})	0.380 / –0.318

Table S7: Crystal data and structure refinement for **3f**.

Identification code	3f
Empirical formula	$C_{42}H_{45}BTi$
Formula weight	608.49
Temperature/K	100(2)
Crystal system	triclinic
Space group	P-1
a/Å	9.9253(2)
b/Å	18.9950(3)
c/Å	20.3682(3)
α/°	84.7380(10)
β/°	88.8230(10)
γ/°	86.3770(10)
Volume/Å^3	3815.79(11)
Z	4
ρ_{calc}g/cm^3	1.059
μ/mm^{-1}	2.070
F(000)	1296.0
Crystal size/mm^3	0.22 × 0.07 × 0.06
Radiation	CuKα (λ = 1.54184)
2Θ range for data collection/°	6.1 to 149.986
Index ranges	-11 ≤ h ≤ 12, -23 ≤ k ≤ 23, -25 ≤ l ≤ 25
Reflections collected	87812
Independent reflections	15119 [R_{int} = 0.0318, R_{sigma} = 0.0246]
Data/restraints/parameters	15119/1229/1021
Goodness-of-fit on F^2	1.049
Final R indexes [I>=2σ (I)]	R_1 = 0.0429, wR_2 = 0.1092
Final R indexes [all data]	R_1 = 0.0500, wR_2 = 0.1129
Maximum/minimum residual electron density (e·Å^{-3})	0.45/-0.80

Table S8: Crystal data and structure refinement for **3f-DMAP**.

Identification code	3f-DMAP
Empirical formula	$C_{49}H_{55}BN_2Ti$
Formula weight	730.66
Temperature/K	100(2)
Crystal system	monoclinic
Space group	P21/n
a/Å	16.9732(2)
b/Å	12.01510(10)
c/Å	25.4409(2)
α/°	90
β/°	99.1780(10)
γ/°	90
Volume/Å^3	5121.86(9)
Z	4
ρ_{calc}g/cm^3	0.948
μ/mm^{-1}	1.619
F(000)	1560.0
Crystal size/mm^3	0.18 × 0.14 × 0.12
Radiation	CuKα (λ = 1.54184)
2Θ range for data collection/°	5.856 to 150.366
Index ranges	$-17 \leqslant h \leqslant 21$, $-14 \leqslant k \leqslant 14$, $-30 \leqslant l \leqslant 30$
Reflections collected	63381
Independent reflections	10108 [R_{int} = 0.0306, R_{sigma} = 0.0225]
Data/restraints/parameters	10108/340/490
Goodness-of-fit on F^2	1.026
Final R indexes [I>=2σ (I)]	R_1 = 0.0516, wR_2 = 0.1328
Final R indexes [all data]	R_1 = 0.0570, wR_2 = 0.1360
Maximum/minimum residual electron density (e·Å^{-3})	0.64/-0.54

Table S9: Crystal data and structure refinement for **4b**.

Identification code	4b
Empirical formula	$C_{24}H_{27}B_2NCl_2$
Formula weight	421.98
Temperature/K	100(2)
Crystal system	triclinic
Space group	P-1
a/Å	8.45820(10)
b/Å	8.52580(10)
c/Å	15.7164(2)
α/°	93.5130(10)
β/°	93.1010(10)
γ/°	93.2260(10)
Volume/Å^3	1127.55(2)
Z	2
ρ_{calc}g/cm^3	1.243
μ/mm^{-1}	2.645
F(000)	444.0
Crystal size/mm^3	0.13 × 0.12 × 0.08
Radiation	$Cu_{K\alpha}$ (λ = 1.54184)
2Θ range for data collection/°	5.644 to 149.836
Index ranges	-10 ≤ h ≤ 10, -9 ≤ k ≤ 10, -19 ≤ l ≤ 19
Reflections collected	21953
Independent reflections	4434 [R_{int} = 0.0325, R_{sigma} = 0.0202]
Data/restraints/parameters	4434/0/269
Goodness-of-fit on F^2	1.058
Final R indexes [I>=2σ (I)]	R_1 = 0.0395, wR_2 = 0.1028
Final R indexes [all data]	R_1 = 0.0419, wR_2 = 0.1042
Maximum/minimum residual electron density (e·Å^{-3})	0.32/-0.40

Table S10: Crystal data and structure refinement for **2h**.

Identification code	2h
Empirical formula	$C_{40}H_{37}B_2N$
Formula weight	553.32
Temperature/K	100(2)
Crystal system	monoclinic
Space group	I2/a
a/Å	19.4360(9)
b/Å	11.4650(7)
c/Å	29.2070(15)
α/°	90
β/°	94.779(5)
γ/°	90
Volume/Å^3	6485.7(6)
Z	8
ρ_{calc}g/cm^3	1.133
μ/mm^{-1}	0.478
F(000)	2352.0
Crystal size/mm^3	0.1 × 0.07 × 0.05
Radiation	$Cu_{K\alpha}$ (λ = 1.54184)
2Θ range for data collection/°	6.074 to 148.642
Index ranges	-24 ≤ h ≤ 24, -14 ≤ k ≤ 14, -29 ≤ l ≤ 36
Reflections collected	33422
Independent reflections	6319 [R_{int} = 0.0702, R_{sigma} = 0.0565]
Data/restraints/parameters	6319/348/450
Goodness-of-fit on F^2	1.099
Final R indexes [I>=2σ (I)]	R_1 = 0.1170, wR_2 = 0.2307
Final R indexes [all data]	R_1 = 0.1438, wR_2 = 0.2421
Maximum/minimum residual electron density (e·Å^{-3})	0.37/-0.34

Table S11: Crystal data and structure refinement for **5a**.

Identification code	5a
Empirical formula	$C_{45}H_{54}BNSiTi$
Formula weight	695.69
Temperature/K	100(2)
Crystal system	triclinic
Space group	$P\bar{1}$
a/Å	9.3091(2)
b/Å	12.6859(4)
c/Å	16.8282(6)
α/°	105.277(3)
β/°	97.899(2)
γ/°	100.904(2)
Volume/Å^3	1845.32(10)
Z	2
ρ_{calc}g/cm^3	1.252
μ/mm^{-1}	2.511
F(000)	744.0
Crystal size/mm^3	0.132 × 0.105 × 0.074
Radiation	CuK_{α} (λ = 1.54184)
2Θ range for data collection/°	5.554 to 149.708
Index ranges	-11 ≤ h ≤ 11, -15 ≤ k ≤ 15, -20 ≤ l ≤ 20
Reflections collected	12961
Independent reflections	12961 [R_{int} = 0.0817, R_{sigma} = 0.0202]
Data/restraints/parameters	12961/0/455
Goodness-of-fit on F^2	1.103
Final R indexes [I>=2σ (I)]	R_1 = 0.0846, wR_2 = 0.2380
Final R indexes [all data]	R_1 = 0.0904, wR_2 = 0.2419
Maximum/minimum residual electron density (e·Å^{-3})	0.84/-0.59

Table S12: Crystal data and structure refinement for **5b**.

Identification code	5b
Empirical formula	$C_{60}H_{67}B_2NTi$
Formula weight	871.66
Temperature/K	100(2)
Crystal system	monoclinic
Space group	P21/n
a/Å	11.92840(10)
b/Å	19.5291(2)
c/Å	23.2072(3)
α/°	90
β/°	95.7820(10)
γ/°	90
Volume/Å^3	5378.63(10)
Z	4
ρ_{calc}g/cm^3	1.076
μ/mm^{-1}	1.606
F(000)	1864.0
Crystal size/mm^3	0.21 × 0.09 × 0.06
Radiation	$Cu_{K\alpha}$ (λ = 1.54184)
2Θ range for data collection/°	5.928 to 150.348
Index ranges	-14 ≤ h ≤ 13, -24 ≤ k ≤ 23, -27 ≤ l ≤ 28
Reflections collected	55060
Independent reflections	10534 [R_{int} = 0.0601, R_{sigma} = 0.0403]
Data/restraints/parameters	10534/0/592
Goodness-of-fit on F^2	1.048
Final R indexes [I>=2σ (I)]	R_1 = 0.0557, wR_2 = 0.1494
Final R indexes [all data]	R_1 = 0.0636, wR_2 = 0.1551
Maximum/minimum residual electron density (e·Å^{-3})	0.63/-0.71

Table S13*:* Crystal data and structure refinement for **6h**

Identification code	6h
Empirical formula	$C_{50}H_{47}B_2NTi$
Formula weight	731.40
Temperature/K	100(2)
Crystal system	monoclinic
Space group	P21/n
a/Å	18.9629(2)
b/Å	10.46170(10)
c/Å	20.8712(3)
α/°	90
β/°	106.9170(10)
γ/°	90
Volume/Å^3	3961.34(8)
Z	4
ρ_{calc}g/cm^3	1.226
μ/mm^{-1}	2.088
F(000)	1544.0
Crystal size/mm^3	0.15 × 0.12 × 0.09
Radiation	$Cu_{K\alpha}$ (λ = 1.54184)
2Θ range for data collection/°	5.548 to 147.518
Index ranges	-23 ≤ h ≤ 21, -12 ≤ k ≤ 12, -25 ≤ l ≤ 25
Reflections collected	41049
Independent reflections	7864 [R_{int} = 0.0290, R_{sigma} = 0.0231]
Data/restraints/parameters	7864/651/539
Goodness-of-fit on F^2	1.060
Final R indexes [I>=2σ (I)]	R_1 = 0.0400, wR_2 = 0.1078
Final R indexes [all data]	R_1 = 0.0478, wR_2 = 0.1123
Maximum/minimum residual electron density (e·Å^{-3})	0.27/-0.46

Computational details

The optimization of reactants, transition states, intermediates and products were carried out by employing DFT hybrid functional (B3PW91)[5] along with small core pseudopotential Stuttgart basis set[6] for titanium, silicon atoms (polarization functions[7] were added for silicon atoms). Pople basis set[8] (6-31G**) were employed for the rest of the atoms. Frequency calculations were performed to confirm the minima for optimized structures (maxima for transition states) and for obtaining thermal corrections over the energies. All the calculations were performed using Gaussian 09 suite of programs.[9]

Table S14: Comparison of selected structural parameters between X-ray and DFT optimized structures for ^{1,2}**Int1**, ^{1,2}**TS1** (B-C bond cleavage) and ^{1,2}**TS1'** (H atom transfer)

Atom labels	DFT (Å)		
	^{1,2}**Int1**	^{1,2}**TS1**	^{1,2}**TS1'**
N4-B47	1.48	1.40	1.45
Ti1-C27	2.07	2.11	2.08
Ti1-C35	2.11	2.25	2.23
C33-C34	1.43	1.42	1.43
C34-C36	1.23	1.24	1.22
C35-B47	1.55	1.42	1.57
C36-B47	1.55	2.27	1.55

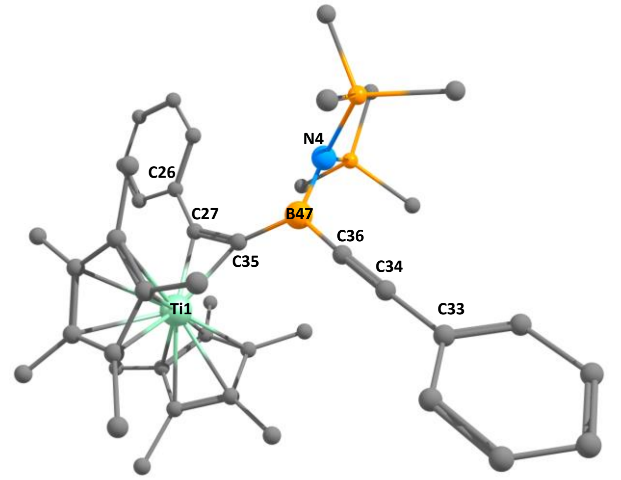

Table S15: Comparison of selected structural parameters between X-ray and DFT optimized structures for ^{1,6}**Int1**, ^{1,6}**TS1** (H atom transfer) and ^{1,6}**TS1'** (B-C bond cleavage)

Atom labels	DFT (Å/ °)		
	^{1,6}**Int1**	^{1,6}**TS1**	^{1,6}**TS1'**
N3-B42	1.44	1.43	1.38
Ti1-C22	2.06	2.09	2.11
Ti1-C30	2.13	2.23	2.33
C28-C29	1.43	1.43	1.43
C29-C31	1.22	1.22	1.24
C30-B42	1.54	1.56	1.39
C31-B42	1.55	1.55	2.76
C34-H80		1.45	
C30-H80		1.54	
C30-H80-C34		152.3	

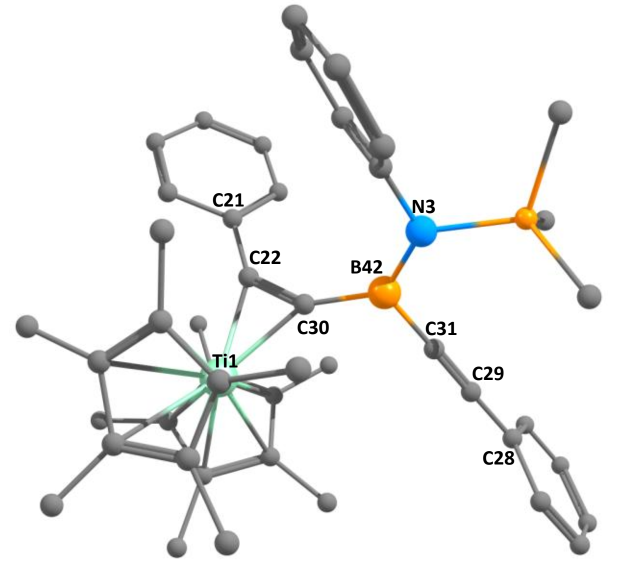

Table S16: DFT computed natural charges for selected atoms in ^{1,2}**Int1**

Atom labels	Natural charges
Ti1	1.89290
N4	-1.57553
C26	-0.09591
C27	-0.33265
C33	-0.12217
C34	-0.00258
C35	-0.71149
C36	-0.27399
B47	0.79622

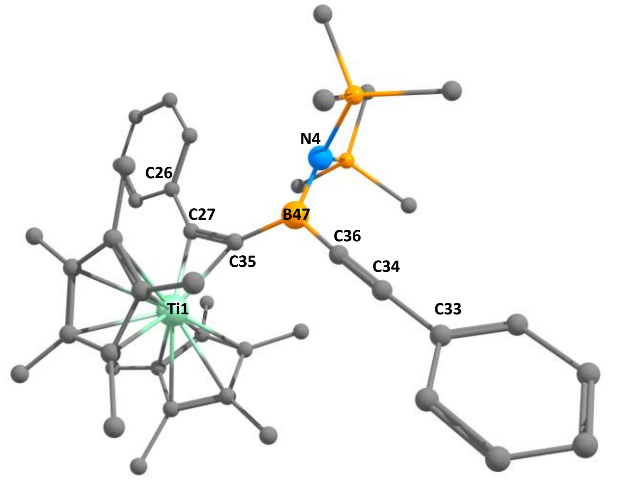

Table S17: DFT computed wiberg bond index between selected atoms in ^{1,2}**Int1**

Atom labels	Wiberg bond index	Atom labels	Wiberg bond index	Atom labels	Wiberg bond index
Ti1	0.0000	Ti1	0.0000	B47	0.0000
C27	0.5919	C35	0.6114	C35	0.9637
Atom labels	Wiberg bond index	Atom labels	Wiberg bond index	Atom labels	Wiberg bond index
B47	0.0000	B47	0.0000	C26	0.0000
C36	0.9388	N4	0.9069	C27	1.0678
Atom labels	Wiberg bond index	Atom labels	Wiberg bond index	Atom labels	Wiberg bond index
C27	0.0000	C33	0.0000	C34	0.0000
C35	1.7933	C34	1.1149	C36	2.6492

Table S18: Bonding orbitals from NBO analysis for ^{1,2}**Int1**

(1.82818) BD (1)Ti 1- C 27
(21.98%) 0.4688*Ti 1 s(18.16%)p 0.01(0.20%)d 4.49(81.61%)f 0.00(0.03%)
(78.02%) 0.8833* C 27 s(20.26%)p 3.94(79.73%)d 0.00(0.02%)
(1.80387) BD (1)Ti 1- C 35
(23.43%) 0.4840*Ti 1 s(15.40%)p 0.01(0.22%)d 5.48(84.35%)f 0.00(0.04%)
(76.57%) 0.8751* C 35 s(16.15%)p 5.19(83.81%)d 0.00(0.04%)
(1.96408) BD (1) N 4- B 47
(76.33%) 0.8737* N 4 s(42.93%)p 1.33(57.01%)d 0.00(0.07%)
(23.67%) 0.4865* B 47 s(32.22%)p 2.10(67.62%)d 0.01(0.16%)
(1.97470) BD (1) C 34- C 36
(50.50%) 0.7107* C 34 s(53.00%)p 0.89(46.94%)d 0.00(0.06%)
(49.50%) 0.7035* C 36 s(47.66%)p 1.10(52.28%)d 0.00(0.07%)
(1.95130) BD (2) C 34- C 36
(52.81%) 0.7267* C 34 s(0.05%)p99.99(99.89%)d 1.18(0.06%)
(47.19%) 0.6869* C 36 s(0.08%)p99.99(99.82%)d 1.13(0.09%)
(1.85296) BD (3) C 34- C 36
(48.70%) 0.6979* C 34 s(0.03%)p99.99(99.91%)d 1.53(0.05%)
(51.30%) 0.7162* C 36 s(0.00%)p 1.00(99.94%)d 0.00(0.06%)
(1.94192) BD (1) C 35- B 47
(66.49%) 0.8154* C 35 s(45.61%)p 1.19(54.35%)d 0.00(0.03%)
(33.51%) 0.5789* B 47 s(35.71%)p 1.80(64.21%)d 0.00(0.08%)
(1.96310) BD (1) C 36- B 47
(67.86%) 0.8238* C 36 s(52.14%)p 0.92(47.83%)d 0.00(0.04%)
(32.14%) 0.5669* B 47 s(31.94%)p 2.13(67.93%)d 0.00(0.12%)

Table S19: DFT computed MOs for ^{1,2}**Int1**. Left-HOMO Right-LUMO

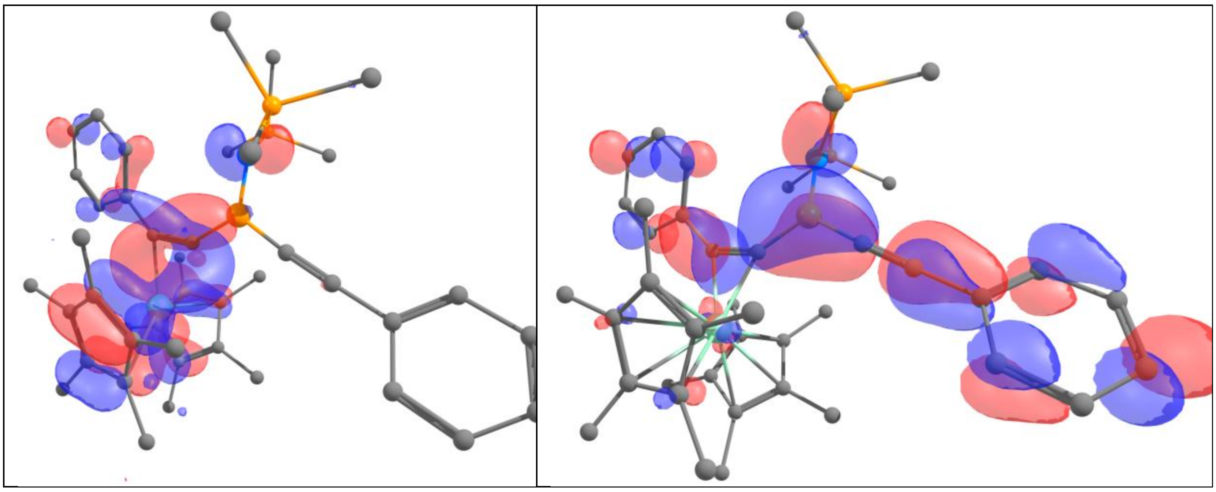

Table S20: DFT computed MOs for ^{1,2}**TS1**. Left-HOMO Right-LUMO

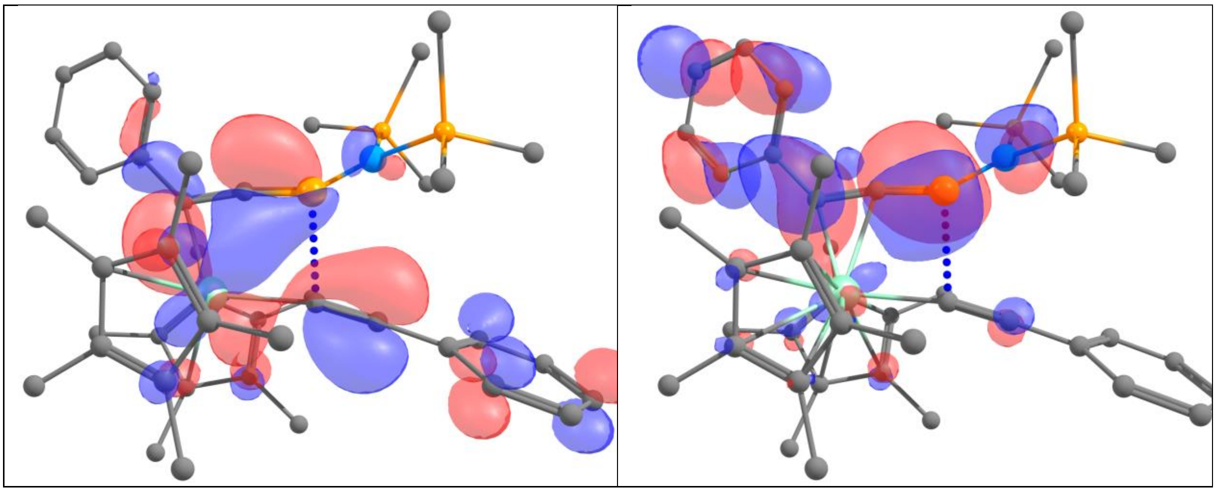

Table S21: DFT computed MOs for ^{1,2}**TS1'**. Left-HOMO Right-LUMO

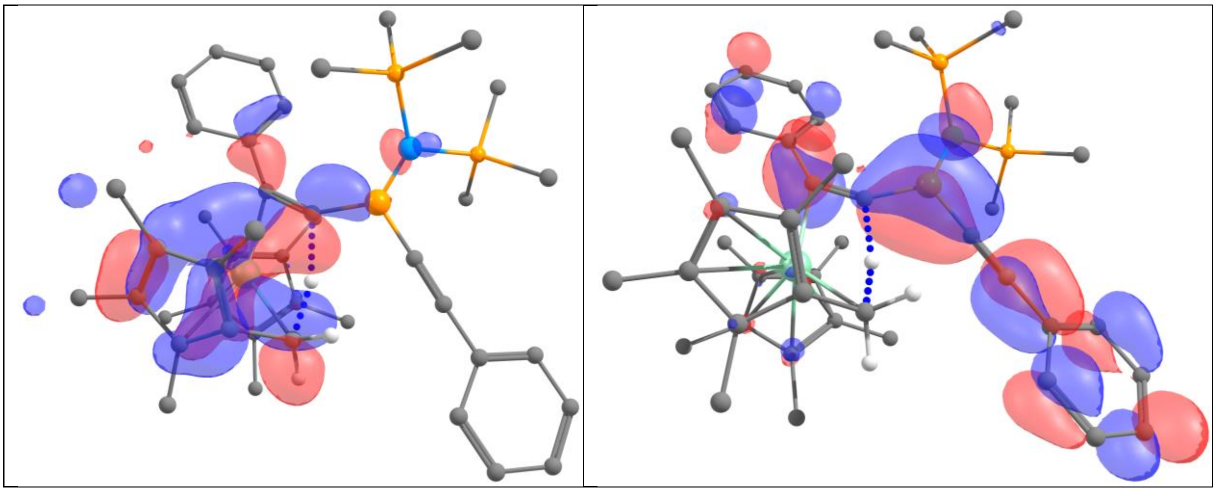

Table S22: Computed natural charges for selected atoms in **3a**.

Atom labels	Natural charges
Ti1	1.40336
C27	-0.30177
C34	-0.41725
C35	-0.41415
C36	-0.18839
B47	0.91262

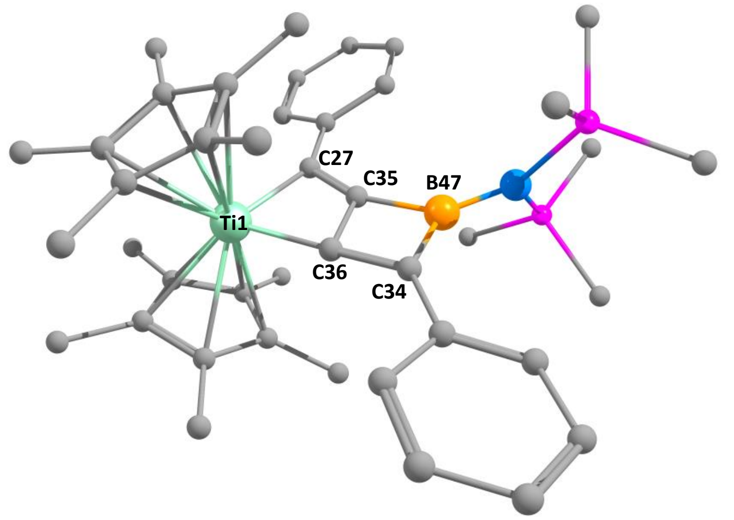

Table S23: Computed Wiberg bond index between selected atoms in **3a**.

Atom labels	Wiberg bond index	Atom labels	Wiberg bond index	Atom labels	Wiberg bond index
Ti1	0.0000	Ti1	0.0000	Ti1	0.0000
C27	0.7002	C35	0.1904	C36	0.7788
Atom labels	Wiberg bond index	Atom labels	Wiberg bond index	Atom labels	Wiberg bond index
C27	0.0000	C35	0.0000	C35	0.0000
C35	1.6614	C36	0.9191	B47	
Atom labels	Wiberg bond index	Atom labels	Wiberg bond index		
C34	0.0000	C34	0.0000		
C36	1.5023	B47	0.9275		

Table S24: Bonding orbitals between selected atoms from NBO analysis for **3a**.

(1.89353) BD (1)Ti 1- C 27
(26.30%)   0.5128*Ti 1 s(11.87%)p 0.00(0.06%)d 7.41(88.03%) f 0.00(0.04%)
(73.70%)   0.8585* C 27 s(24.99%)p 3.00(74.99%)d 0.00(0.02%)
(1.94159) BD (1)Ti 1- C 36
(23.82%)   0.4880*Ti 1 s(13.14%)p 0.00(0.05%)d 6.60(86.76%) f 0.00(0.05%)
(76.18%)   0.8728* C 36 s(32.70%)p 2.06(67.28%)d 0.00(0.02%)
(1.94247) BD (1) C 27- C 35
(48.18%)   0.6941* C 27 s(36.58%)p 1.73(63.34%)d 0.00(0.08%)
(51.82%)   0.7199* C 35 s(42.15%)p 1.37(57.78%)d 0.00(0.07%)
(1.78188) BD (2) C 27- C 35
(46.88%)   0.6847* C 27 s(0.26%)p99.99(99.64%)d 0.38(0.10%)
(53.12%)   0.7288* C 35 s(0.03%)p99.99(99.90%)d 1.93(0.06%)
(1.96232) BD (1) C 34- C 36
(51.69%)   0.7190* C 34 s(30.62%)p 2.26(69.30%)d 0.00(0.08%)
(48.31%)   0.6951* C 36 s(42.18%)p 1.37(57.74%)d 0.00(0.08%)
(1.93654) BD (1) C 34- B 47
(69.24%)   0.8321* C 34 s(34.01%)p 1.94(65.95%)d 0.00(0.04%)
(30.76%)   0.5546* B 47 s(34.71%)p 1.88(65.19%)d 0.00(0.10%)
(1.84130) BD (1) C 35- C 36
(52.24%)   0.7228* C 35 s(21.44%)p 3.66(78.45%)d 0.01(0.11%)
(47.76%)   0.6911* C 36 s(24.85%)p 3.02(75.02%)d 0.01(0.13%)
(1.91964) BD (1) C 35- B 47
(70.37%)   0.8389* C 35 s(36.22%)p 1.76(63.75%)d 0.00(0.03%)
(29.63%)   0.5444* B 47 s(30.81%)p 2.24(69.07%)d 0.00(0.12%)

Table S25: DFT computed NBO second order perturbation analysis for **3a**.

Donor NBO	Acceptor NBO	E(2) kcal/mol
(1.94247) BD (1) C 27- C 35<br>(48.18%) 0.6941* C 27 s(36.58%)p 1.73(63.34%)d 0.00(0.08%)<br>(51.82%) 0.7199* C 35 s(42.15%)p 1.37(57.78%)d 0.00(0.07%)	(0.33572) LV (3)Ti 1<br>s(3.34%)p 0.02(0.07%)d28.93(96.54%)f 0.02(0.05%)	15.23
(1.94247) BD (1) C 27- C 35<br>(48.18%) 0.6941* C 27 s(36.58%)p 1.73(63.34%)d 0.00(0.08%)<br>(51.82%) 0.7199* C 35 s(42.15%)p 1.37(57.78%)d 0.00(0.07%)	(0.15978) LV (4)Ti 1<br>s(69.52%)p 0.01(1.00%)d 0.42(29.42%)f 0.00(0.07%)	6.99
(1.94247) BD (1) C 27- C 35<br>(48.18%) 0.6941* C 27 s(36.58%)p 1.73(63.34%)d 0.00(0.08%)<br>(51.82%) 0.7199* C 35 s(42.15%)p 1.37(57.78%)d 0.00(0.07%)	(0.06216) BD*(1) C 35- B 47<br>(29.63%) 0.5444* C 35 s(36.22%)p 1.76(63.75%)d 0.00(0.03%)<br>(70.37%) -0.8389* B 47 s(30.81%)p 2.24(69.07%)d 0.00(0.12%)	10.53
(1.78188) BD (2) C 27- C 35<br>(46.88%) 0.6847* C 27<br>s(0.26%)p99.99(99.64%)d 0.38(0.10%)<br>(53.12%) 0.7288* C 35<br>s(0.03%)p99.99(99.90%)d 1.93(0.06%)	(0.39047) LV (2)Ti 1<br>s(0.04%)p 1.15(0.04%)d99.99(99.91%)f 0.19(0.01%)	27.41
(1.93654) BD (1) C 34- B 47<br>(69.24%) 0.8321* C 34 s(34.01%)p 1.94(65.95%)d 0.00(0.04%)<br>(30.76%) 0.5546* B 47 s(34.71%)p 1.88(65.19%)d 0.00(0.10%)	(0.15343) BD*(1)Ti 1- C 36<br>(76.18%) 0.8728*Ti 1 s(13.14%)p 0.00(0.05%)d 6.60(86.76%)f 0.00(0.05%)<br>(23.82%) -0.4880* C 36 s(32.70%)p 2.06(67.28%)d 0.00(0.02%)	15.76
(1.84130) BD (1) C 35- C 36<br>(52.24%) 0.7228* C 35 s(21.44%)p 3.66(78.45%)d 0.01(0.11%)<br>(47.76%) 0.6911* C 36 s(24.85%)p 3.02(75.02%)d 0.01(0.13%)	(0.33572) LV (3)Ti 1<br>s(3.34%)p 0.02(0.07%)d28.93(96.54%)f 0.02(0.05%)	22.38
(1.84130) BD (1) C 35- C 36<br>(52.24%) 0.7228* C 35 s(21.44%)p 3.66(78.45%)d 0.01(0.11%)<br>(47.76%) 0.6911* C 36 s(24.85%)p 3.02(75.02%)d 0.01(0.13%)	(0.15978) LV (4)Ti 1<br>s(69.52%)p 0.01(1.00%)d 0.42(29.42%)f 0.00(0.07%)	16.71
(1.91964) BD (1) C 35- B 47<br>(70.37%) 0.8389* C 35 s(36.22%)p 1.76(63.75%)d 0.00(0.03%)<br>(29.63%) 0.5444* B 47 s(30.81%)p 2.24(69.07%)d 0.00(0.12%)	(0.33572) LV (3)Ti 1<br>s(3.34%)p 0.02(0.07%)d28.93(96.54%)f 0.02(0.05%)	5.33

Table S26: DFT computed MOs for **3a**. (a)HOMO-2 (b)HOMO-1 (c)HOMO (d)LUMO

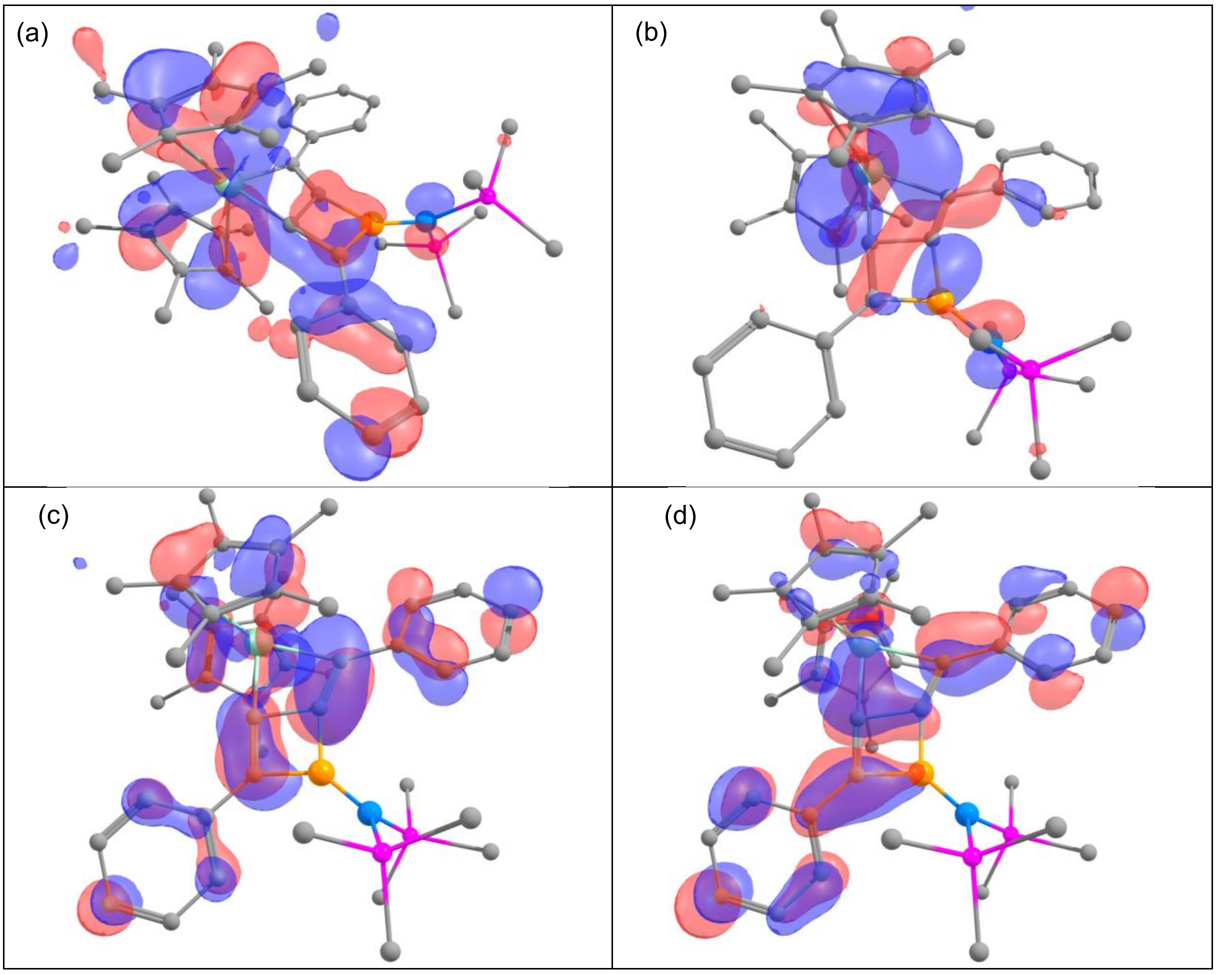

Table S27: DFT computed natural charges for selected atoms in **[1,6]Int1**

Atom labels	Natural charges
Ti1	1.87441
N3	-1.06670
C21	-0.06908
C22	-0.36864
C28	-0.12200
C29	-0.01332
C30	-0.64966
C31	-0.25167
B42	0.73126

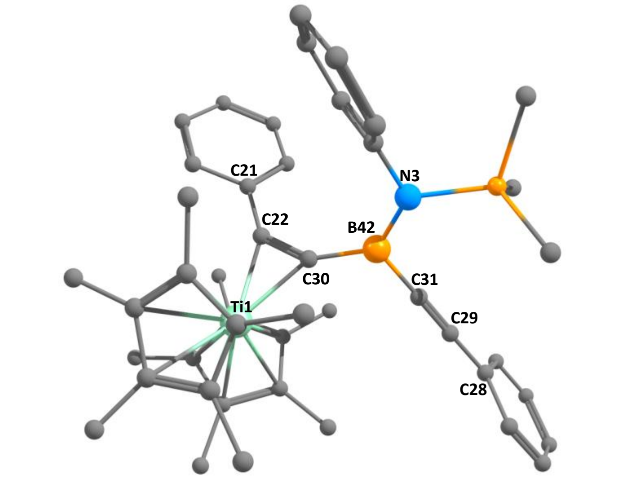

Table S28: DFT computed wiberg bond index between selected atoms in **[1,6]Int1**

Atom labels	Wiberg bond index	Atom labels	Wiberg bond index	Atom labels	Wiberg bond index
Ti1	0.0000	Ti1	0.0000	B42	0.0000
C22	0.6114	C30	0.5719	C30	0.9846
Atom labels	Wiberg bond index	Atom labels	Wiberg bond index	Atom labels	Wiberg bond index
B42	0.0000	B42	0.0000	C22	0.0000
C31	0.9315	N3	1.0352	C30	1.8513
Atom labels	Wiberg bond index	Atom labels	Wiberg bond index	Atom labels	Wiberg bond index
C21	0.0000	C28	0.0000	C29	0.0000
C22	1.0548	C29	1.1065	C31	2.6731

Table S29 Bonding orbitals from NBO analysis for **[1,6]Int1**

(1.82191) BD (1)Ti 1- C 22
(22.83%) 0.4778*Ti 1 s(17.31%)p 0.01(0.17%)d 4.76(82.48%) f 0.00(0.03%)
(77.17%) 0.8785* C 22 s(19.54%)p 4.12(80.44%)d 0.00(0.02%)
(1.75863) BD (1)Ti 1- C 30
(22.46%) 0.4740*Ti 1 s(16.09%)p 0.02(0.26%)d 5.20(83.61%) f 0.00(0.04%)
(77.54%) 0.8806* C 30 s(13.76%)p 6.26(86.21%)d 0.00(0.03%)
(1.96315) BD (1) N 3- B 42
(76.93%) 0.8771* N 3 s(41.88%)p 1.39(58.07%)d 0.00(0.05%)
(23.07%) 0.4804* B 42 s(31.46%)p 2.17(68.35%)d 0.01(0.19%)
(1.90408) BD (2) N 3- B 42
(86.28%) 0.9289* N 3 s(1.01%)p97.91(98.99%)d 0.00(0.00%)
(13.72%) 0.3704* B 42 s(0.53%)p99.99(99.17%)d 0.55(0.29%)
(1.96193) BD (1) C 21- C 22
(51.53%) 0.7179* C 21 s(32.51%)p 2.07(67.44%)d 0.00(0.05%)
(48.47%) 0.6962* C 22 s(39.60%)p 1.52(60.33%)d 0.00(0.06%)
(1.81802) BD (2) C 22- C 30
(49.55%) 0.7039* C 22 s(0.00%)p 1.00(99.91%)d 0.00(0.09%)
(50.45%) 0.7103* C 30 s(0.10%)p99.99(99.82%)d 0.85(0.08%)
(1.97301) BD (1) C 28- C 29
(50.77%) 0.7125* C 28 s(31.52%)p 2.17(68.41%)d 0.00(0.06%)
(49.23%) 0.7017* C 29 s(46.82%)p 1.13(53.12%)d 0.00(0.05%)
(1.97493) BD (1) C 29- C 31
(50.52%) 0.7107* C 29 s(53.02%)p 0.88(46.92%)d 0.00(0.06%)
(49.48%) 0.7035* C 31 s(48.06%)p 1.08(51.88%)d 0.00(0.07%)
(1.92112) BD (2) C 29- C 31
(49.63%) 0.7045* C 29 s(0.03%)p99.99(99.91%)d 1.84(0.06%)
(50.37%) 0.7097* C 31 s(0.01%)p99.99(99.92%)d 5.35(0.07%)
(1.90036) BD (3) C 29- C 31
(52.37%) 0.7237* C 29 s(0.01%)p 1.00(99.94%)d 0.00(0.05%)
(47.63%) 0.6901* C 31 s(0.00%)p 1.00(99.91%)d 0.00(0.08%)
(1.93979) BD (1) C 30- B 42
(65.80%) 0.8112* C 30 s(46.08%)p 1.17(53.89%)d 0.00(0.03%)
(34.20%) 0.5848* B 42 s(36.72%)p 1.72(63.21%)d 0.00(0.07%)
(1.95883) BD (1) C 31- B 42
(67.06%) 0.8189* C 31 s(51.86%)p 0.93(48.11%)d 0.00(0.03%)
(32.94%) 0.5739* B 42 s(31.20%)p 2.20(68.68%)d 0.00(0.11%)

Table S30. Computed MOs for ^{1,6}**Int1**. Left-HOMO Right-LUMO

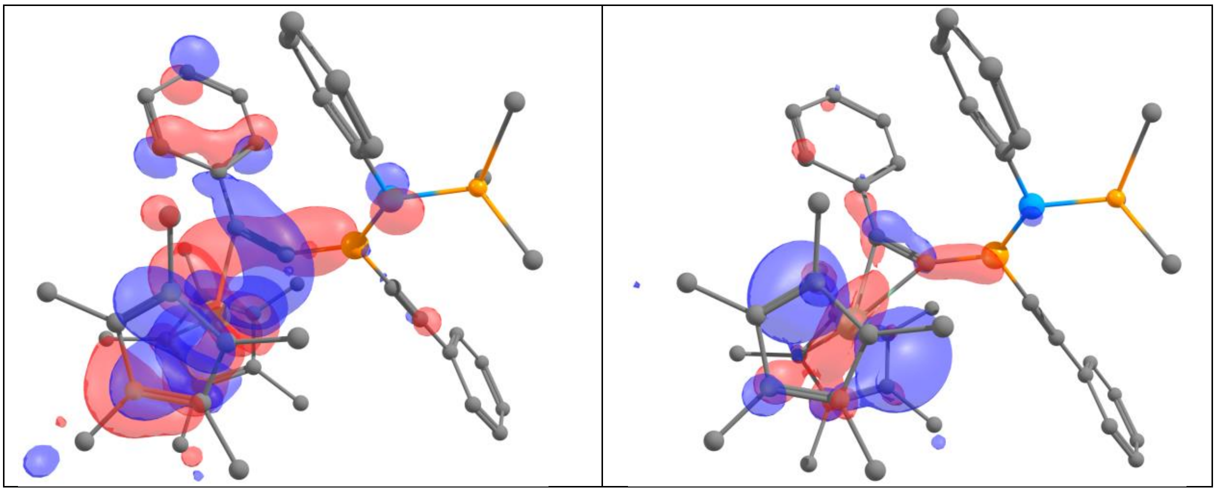

Table S31: DFT computed MOs for ^{1,6}**TS1**. Left-HOMO Right-LUMO

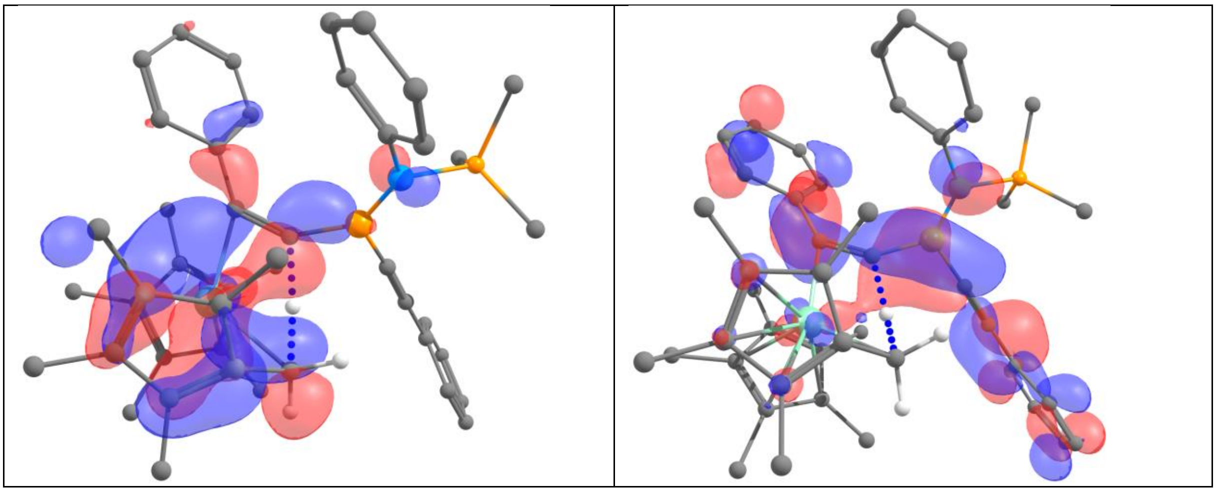

Table S32: DFT computed MOs for ^{1,6}**TS1’**. Left-HOMO Right-LUMO

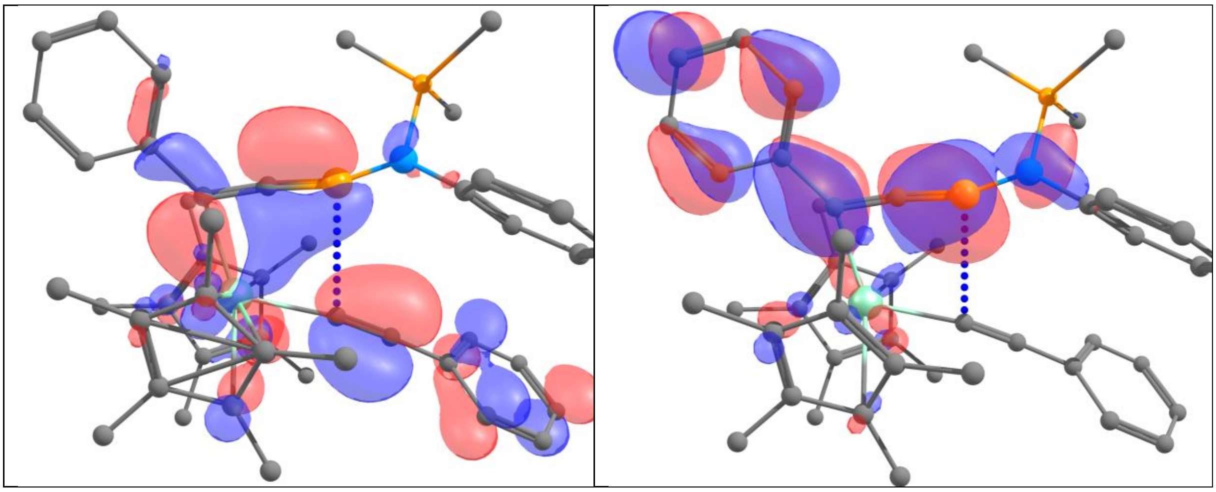

Table S33: Computed natural charges for selected atoms in **5a**.

Atom labels	Natural charges	
Ti1	1.87783	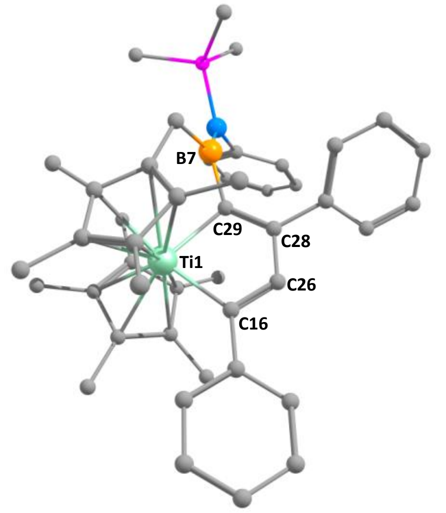
B7	0.87402	
C16	-0.44609	
C26	-0.26295	
C28	-0.07452	
C29	-0.69650	

Table S34: Computed Wiberg bond index between selected atoms in **5a**.

Atom labels	Wiberg bond index	Atom labels	Wiberg bond index	Atom labels	Wiberg bond index
Ti1	0.0000	Ti1	0.0000	Ti1	0.0000
C16	0.5828	C26	0.0502	C28	0.0432
Atom labels	Wiberg bond index	Atom labels	Wiberg bond index	Atom labels	Wiberg bond index
Ti1	0.0000	C16	0.0000	C26	0.0000
C29	0.5795	C26	1.6621	C28	1.1052
Atom labels	Wiberg bond index				
C28	0.0000				
C29	1.6458				

Table S35: Bonding orbitals between selected atoms from NBO analysis for **5a**.

(1.92398) BD (1)Ti 1- C 16
(19.15%) 0.4377*Ti 1 s(22.82%)p 0.01(0.19%)d 3.37(76.94%) f 0.00(0.04%)
(80.85%) 0.8991* C 16 s(27.80%)p 2.60(72.19%)d 0.00(0.01%)
(1.85293) BD (1)Ti 1- C 29
(20.36%) 0.4513*Ti 1 s(20.64%)p 0.01(0.23%)d 3.83(79.09%) f 0.00(0.04%)
(79.64%) 0.8924* C 29 s(19.87%)p 4.03(80.11%)d 0.00(0.02%)
(1.80222) BD (2) C 16- C 26
(47.20%) 0.6870* C 16 s(0.01%)p 1.00(99.91%)d 0.00(0.08%)
(52.80%) 0.7266* C 26 s(0.00%)p 1.00(99.95%)d 0.00(0.04%)
(1.96053) BD (1) C 26- C 28
(49.14%) 0.7010* C 26 s(33.45%)p 1.99(66.48%)d 0.00(0.07%)
(50.86%) 0.7131* C 28 s(31.41%)p 2.18(68.53%)d 0.00(0.06%)
(1.95095) BD (1) C 28- C 29
(51.84%) 0.7200* C 28 s(37.29%)p 1.68(62.66%)d 0.00(0.05%)
(48.16%) 0.6939* C 29 s(38.80%)p 1.58(61.11%)d 0.00(0.09%)
(1.79575) BD (2) C 28- C 29
(53.00%) 0.7280* C 28 s(0.00%)p 1.00(99.96%)d 0.00(0.03%)
(47.00%) 0.6856* C 29 s(0.02%)p99.99(99.90%)d 3.40(0.08%)

Table S36: DFT computed NBO second order perturbation analysis for **5a**.

Donor NBO	Acceptor NBO	E(2)<br>kcal/mol
(1.80222) BD (2) C 16- C 26<br>(47.20%) 0.6870* C 16 s(0.01%)p<br>1.00(99.91%)d 0.00(0.08%)<br>(52.80%) 0.7266* C 26 s(0.00%)p<br>1.00(99.95%)d 0.00(0.04%)	(0.29067) LV (2)Ti 1<br>s(0.14%)p 0.91(0.13%)d99.99(99.68%)f<br>0.34(0.05%)	8.43
(1.96053) BD (1) C 26- C 28<br>(49.14%) 0.7010* C 26 s(33.45%)p<br>1.99(66.48%)d 0.00(0.07%)<br>(50.86%) 0.7131* C 28 s(31.41%)p<br>2.18(68.53%)d 0.00(0.06%)	(0.10949) LV (4)Ti 1<br>s(5.64%)p 0.04(0.24%)d16.67(94.08%)f<br>0.01(0.03%)	3.44
(1.79575) BD (2) C 28- C 29<br>(53.00%) 0.7280* C 28 s(0.00%)p<br>1.00(99.96%)d 0.00(0.03%)<br>(47.00%) 0.6856* C 29<br>s(0.02%)p99.99(99.90%)d 3.40(0.08%)	(0.32572) LV (1)Ti 1<br>s(0.24%)p 0.02(0.00%)d99.99(99.74%)f<br>0.06(0.02%)	3.95
(1.79575) BD (2) C 28- C 29<br>(53.00%) 0.7280* C 28 s(0.00%)p<br>1.00(99.96%)d 0.00(0.03%)<br>(47.00%) 0.6856* C 29<br>s(0.02%)p99.99(99.90%)d 3.40(0.08%)	(0.29067) LV (2)Ti 1<br>s(0.14%)p 0.91(0.13%)d99.99(99.68%)f<br>0.34(0.05%)	4.46

Table S37: DFT computed MOs for **5a**. (a)HOMO-2 (b)HOMO-1 (c)HOMO (d)LUMO.

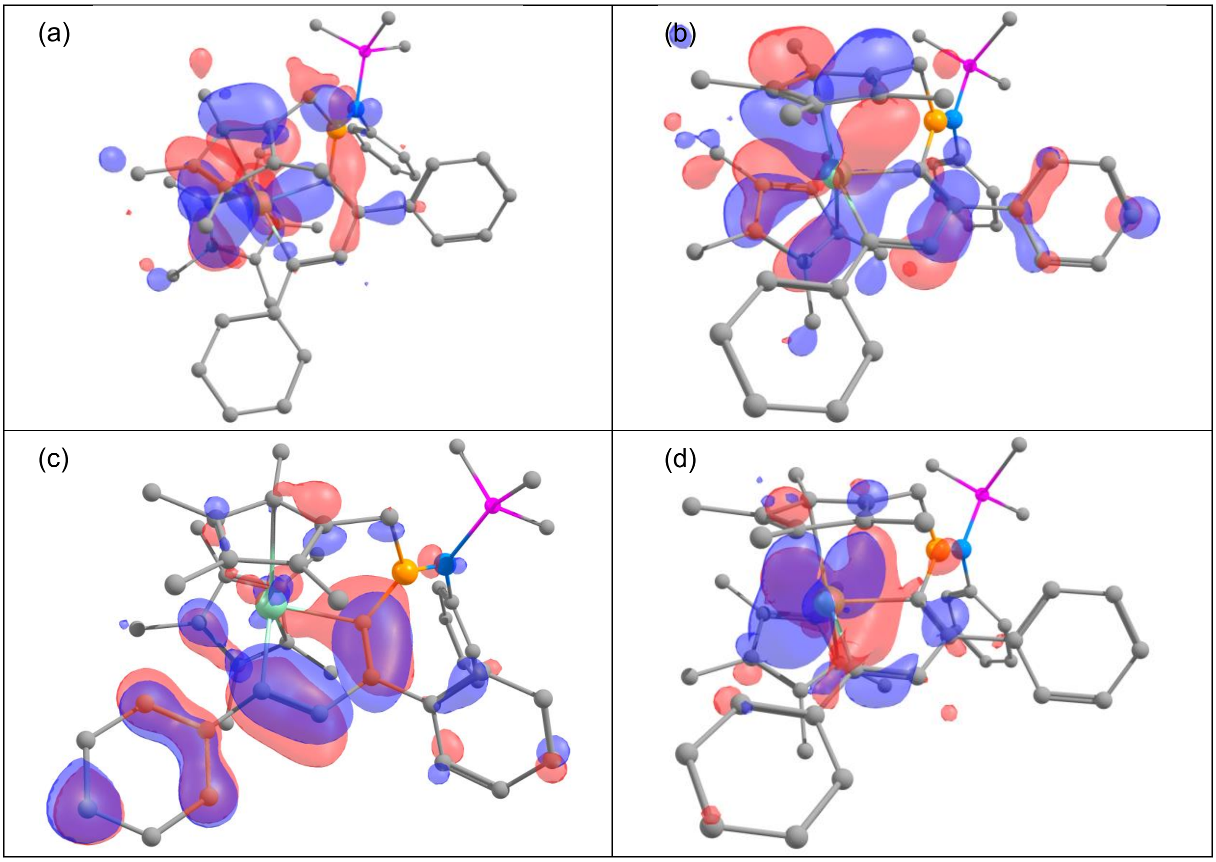

Optimized geometries

[1,2]Int1

Ti	-1.978488000	0.016650000	0.174250000
Si	3.507402000	0.506811000	1.751177000
Si	3.891111000	-1.483043000	-0.576659000
N	2.786459000	-0.490646000	0.443450000
C	-4.874487000	2.157432000	-0.690887000
C	-1.965094000	2.915734000	-1.765591000
C	-1.627269000	1.802744000	3.222278000
C	-4.577779000	1.151679000	2.318513000
C	-0.038667000	2.896579000	0.703422000
C	-3.584351000	1.923476000	0.032634000
C	-2.301139000	2.335699000	-0.423593000
C	-1.400621000	2.283476000	0.678852000
C	-2.113474000	1.783690000	1.805192000
C	-3.456927000	1.532273000	1.394295000
C	2.351612000	0.805996000	3.213771000
C	5.061243000	-0.291457000	2.488338000
C	3.999111000	2.196069000	1.039456000
C	2.982553000	-2.264597000	-2.042134000
C	4.533996000	-2.957773000	0.431014000
C	5.351766000	-0.501854000	-1.287507000
C	-1.297928000	-1.624676000	3.695433000
C	-1.084600000	-2.409678000	4.825152000
C	0.104065000	-3.126049000	4.963157000
C	1.078728000	-3.041859000	3.966122000
C	0.874923000	-2.236954000	2.850257000
C	-0.320935000	-1.508003000	2.691227000
C	-0.563900000	-0.711889000	1.494466000
C	0.999306000	2.078476000	-4.780454000
C	1.225523000	2.803580000	-5.945190000
C	2.420458000	3.502660000	-6.117621000
C	3.393010000	3.470460000	-5.117520000
C	3.177590000	2.741514000	-3.953637000
C	1.974505000	2.033652000	-3.766406000
C	1.759973000	1.288279000	-2.570597000
C	0.101969000	-0.203819000	0.469966000
C	1.609701000	0.647878000	-1.535947000
C	-5.302931000	-1.276935000	-0.286325000
C	-3.736280000	-0.217575000	-2.911110000
C	-0.870752000	-1.438652000	-2.856392000
C	-3.358785000	-3.062917000	1.340384000
C	-0.616196000	-3.289754000	-0.304863000
C	-3.143707000	-0.913264000	-1.721733000
C	-1.848657000	-1.518508000	-1.725495000
C	-1.755200000	-2.370459000	-0.598318000
C	-2.954559000	-2.244014000	0.151325000
C	-3.830996000	-1.369379000	-0.560138000
B	1.512310000	-0.048118000	-0.155554000
H	-0.992130000	2.566085000	-2.125516000
H	-1.911970000	4.011322000	-1.713224000
H	-2.714851000	2.666840000	-2.520538000
H	-5.532912000	-1.004398000	0.747449000
H	-5.774017000	-2.252223000	-0.467705000
H	-5.798808000	-0.560222000	-0.942694000
H	-4.755127000	2.141277000	-1.775504000
H	-5.257655000	3.153457000	-0.430063000

H	-5.652021000	1.440194000	-0.423330000
H	0.465416000	2.825338000	-6.721248000
H	-5.499641000	0.941623000	1.771948000
H	-3.866749000	-0.930796000	-3.736134000
H	-3.101213000	0.589321000	-3.289165000
H	-4.718742000	0.204833000	-2.694580000
H	-0.842654000	-0.442237000	-3.305039000
H	-1.142127000	-2.147915000	-3.651114000
H	0.587009000	2.455716000	1.481362000
H	0.481042000	2.777322000	-0.250348000
H	0.070048000	1.534547000	-4.643650000
H	-2.501639000	-3.379759000	1.938208000
H	-3.893926000	-3.967570000	1.020151000
H	-4.034058000	-2.512577000	2.002782000
H	-2.310177000	1.273450000	3.891226000
H	-1.555801000	2.837276000	3.584232000
H	-0.641518000	1.347119000	3.337845000
H	0.339400000	-2.756167000	-0.308096000
H	-0.559800000	-4.083171000	-1.060691000
H	-0.719577000	-3.766403000	0.671997000
H	3.931368000	2.713647000	-3.172982000
H	-4.797190000	1.968855000	3.017895000
H	-4.345666000	0.269241000	2.925319000
H	4.743352000	2.090127000	0.244601000
H	4.425217000	2.834797000	1.821463000
H	3.133047000	2.713460000	0.615198000
H	4.982296000	0.303704000	-1.929552000
H	5.975520000	-1.161300000	-1.901747000
H	5.992996000	-0.059523000	-0.519916000
H	-2.232561000	-1.086386000	3.570487000
H	2.593086000	4.070554000	-7.027532000
H	2.121580000	-2.858277000	-1.721380000
H	3.680925000	-2.947864000	-2.539910000
H	2.646687000	-1.528712000	-2.774638000
H	2.005041000	-3.602521000	4.064644000
H	0.268673000	-3.749202000	5.838119000
H	-1.851439000	-2.473783000	5.592921000
H	0.144771000	-1.674081000	-2.535582000
H	1.355968000	1.115313000	2.893762000
H	2.775947000	1.604701000	3.833002000
H	2.239230000	-0.087325000	3.832178000
H	4.324777000	4.014125000	-5.246703000
H	1.637698000	-2.147748000	2.082162000
H	5.211329000	-2.683419000	1.241278000
H	5.075261000	-3.632508000	-0.242669000
H	3.698856000	-3.520985000	0.860309000
H	4.829211000	-1.234133000	2.992170000
H	5.445254000	0.400011000	3.248010000
H	5.866354000	-0.466666000	1.770743000
H	-0.110067000	3.972902000	0.914179000

^{1,2}TS1’

Ti	-2.247335000	-0.047145000	-0.092328000
Si	3.859566000	-0.595703000	1.438200000
N	2.299527000	-1.505626000	1.210186000
C	-5.296499000	1.725761000	0.626502000
C	-3.167692000	3.274435000	-1.254920000

C	-0.671124000	1.457696000	2.768272000
C	-3.760485000	0.617379000	3.079497000
C	-3.801978000	1.667915000	0.713643000
C	-2.867935000	2.332579000	-0.127678000
C	-1.585812000	2.242983000	0.490493000
C	-1.739390000	1.576981000	1.730772000
C	-3.100505000	1.178613000	1.855800000
C	4.801427000	-1.231880000	2.961241000
C	5.038387000	-0.748602000	-0.036623000
C	3.520384000	1.220089000	1.862098000
C	-2.717052000	-2.560191000	2.854722000
C	-2.944104000	-3.197231000	4.070963000
C	-1.960154000	-3.188015000	5.059434000
C	-0.744405000	-2.548709000	4.811770000
C	-0.504481000	-1.946518000	3.580033000
C	-1.488180000	-1.938544000	2.573338000
C	-1.286495000	-1.254998000	1.300536000
C	1.918925000	2.027367000	-3.733816000
C	2.408104000	2.873886000	-4.722557000
C	3.663225000	3.467271000	-4.584455000
C	4.432140000	3.205479000	-3.450220000
C	3.954479000	2.354398000	-2.460108000
C	2.687057000	1.753839000	-2.586385000
C	2.197080000	0.888779000	-1.564930000
C	-0.276630000	-0.894089000	0.524700000
C	1.802272000	0.157561000	-0.665056000
C	-5.813522000	-1.387275000	-0.838611000
C	-4.410579000	0.725060000	-2.773332000
C	-1.227688000	0.201204000	-2.122256000
C	-3.744391000	-3.668741000	-0.278697000
C	-1.019952000	-2.915415000	-1.687245000
C	-3.717219000	-0.318020000	-1.950881000
C	-2.354218000	-0.732819000	-2.157771000
C	-2.196926000	-2.011981000	-1.531719000
C	-3.411183000	-2.340126000	-0.884757000
C	-4.349269000	-1.300715000	-1.146407000
B	1.285353000	-0.832430000	0.415046000
H	-2.526888000	3.118290000	-2.129834000
H	-2.993447000	4.306962000	-0.923931000
H	-4.205634000	3.213310000	-1.584818000
H	-6.001993000	-1.685304000	0.198286000
H	-6.299270000	-2.137095000	-1.477859000
H	-6.323920000	-0.437429000	-1.009226000
H	-5.654589000	1.892316000	-0.390849000
H	-5.674740000	2.555877000	1.238884000
H	-5.766336000	0.813833000	1.002752000
H	1.805898000	3.073223000	-5.604653000
H	-4.563357000	-0.082801000	2.830699000
H	-4.875324000	0.242723000	-3.644008000
H	-3.709912000	1.465410000	-3.162798000
H	-5.205814000	1.252807000	-2.242319000
H	-1.406586000	1.208644000	-2.497755000
H	-0.281251000	-0.187202000	-2.501344000
H	0.942331000	1.565985000	-3.839595000
H	-2.903818000	-4.095324000	0.275309000
H	-4.013941000	-4.389382000	-1.063337000
H	-4.598220000	-3.608149000	0.402301000

H	-0.968524000	0.798637000	3.585652000
H	-0.450356000	2.443056000	3.198288000
H	0.260964000	1.066151000	2.347899000
H	-0.080855000	-2.363582000	-1.785127000
H	-1.135946000	-3.528864000	-2.591275000
H	-0.919671000	-3.590757000	-0.837907000
H	4.552046000	2.146585000	-1.578305000
H	-4.209364000	1.423810000	3.675941000
H	-3.056878000	0.084499000	3.721045000
H	3.061233000	1.779470000	1.046139000
H	4.471753000	1.701930000	2.116937000
H	2.870980000	1.291672000	2.740838000
H	-3.493629000	-2.519233000	2.098969000
H	4.040553000	4.130494000	-5.357735000
H	0.024823000	-2.529066000	5.579369000
H	-2.140968000	-3.667560000	6.017638000
H	-3.898568000	-3.682936000	4.257445000
H	-0.638972000	-0.020478000	-0.786605000
H	4.250346000	-1.050951000	3.888286000
H	5.730545000	-0.651390000	3.016569000
H	5.080787000	-2.286909000	2.929805000
H	5.410365000	3.664711000	-3.337780000
H	0.445098000	-1.459374000	3.382625000
H	5.436507000	-1.763655000	-0.120687000
H	5.888205000	-0.070428000	0.106206000
H	4.547536000	-0.494091000	-0.977599000
C	-0.355720000	2.956979000	0.022300000
H	-0.379389000	4.009865000	0.336730000
H	0.552496000	2.509709000	0.429937000
H	-0.260921000	2.945481000	-1.066876000
Si	2.339705000	-3.245558000	1.688214000
C	2.507456000	-3.512768000	3.568167000
H	1.693761000	-4.151956000	3.922988000
H	3.454649000	-4.009550000	3.800898000
H	2.470866000	-2.582904000	4.139528000
C	3.833911000	-4.061579000	0.840642000
H	4.803069000	-3.691608000	1.184723000
H	3.801753000	-5.138001000	1.047804000
H	3.784553000	-3.930505000	-0.245024000
C	0.863558000	-4.236308000	1.067262000
H	0.883310000	-4.320215000	-0.021606000
H	0.958778000	-5.247414000	1.480950000
H	-0.098181000	-3.829554000	1.381116000

^{1,2}Int2’

Ti	-2.038392000	0.416994000	-0.575549000
Si	4.315242000	-0.513206000	1.347328000
N	2.701306000	-1.283089000	1.006175000
C	-5.123880000	1.941897000	0.561609000
C	-3.481957000	3.569404000	-1.687019000
C	-0.206195000	2.171437000	1.917805000
C	-3.131838000	1.170832000	2.797918000
C	-3.634741000	2.024759000	0.416181000
C	-2.909757000	2.709649000	-0.600730000
C	-1.537325000	2.742709000	-0.214899000
C	-1.429857000	2.128879000	1.059873000
C	-2.723337000	1.663596000	1.443440000

C	5.165123000	-1.325678000	2.840070000
C	5.503782000	-0.655078000	-0.119102000
C	4.084433000	1.289415000	1.881850000
C	-2.252894000	-1.964148000	2.489975000
C	-2.457220000	-2.505409000	3.755377000
C	-1.479372000	-2.369892000	4.740346000
C	-0.291323000	-1.704800000	4.435855000
C	-0.071748000	-1.200678000	3.156399000
C	-1.051938000	-1.314643000	2.151172000
C	-0.900679000	-0.714965000	0.823707000
C	2.470305000	2.707873000	-3.647429000
C	2.971030000	3.593460000	-4.595149000
C	4.287586000	4.047092000	-4.508676000
C	5.106240000	3.607330000	-3.467890000
C	4.615876000	2.717329000	-2.519094000
C	3.287969000	2.255577000	-2.594878000
C	2.784897000	1.342661000	-1.622931000
C	0.219633000	-0.474854000	0.106547000
C	2.390740000	0.553293000	-0.773164000
C	-5.362328000	-1.350697000	-0.738655000
C	-4.608275000	0.844925000	-2.913165000
C	-1.354562000	0.833764000	-2.708907000
C	-2.922857000	-3.342436000	-0.565147000
C	-0.575321000	-2.207592000	-2.328172000
C	-3.642260000	-0.048780000	-2.197028000
C	-2.276400000	-0.263209000	-2.613929000
C	-1.851902000	-1.498535000	-2.014951000
C	-2.896069000	-1.985823000	-1.196343000
C	-3.991516000	-1.082099000	-1.282635000
B	1.794672000	-0.503485000	0.196551000
H	-2.966385000	3.453305000	-2.645705000
H	-3.379637000	4.625250000	-1.403051000
H	-4.543936000	3.383240000	-1.852090000
H	-5.335383000	-1.698999000	0.299029000
H	-5.869731000	-2.128125000	-1.326100000
H	-5.992005000	-0.459566000	-0.771041000
H	-5.646757000	1.999688000	-0.395423000
H	-5.489017000	2.776086000	1.176425000
H	-5.434904000	1.019923000	1.059690000
H	2.330591000	3.932804000	-5.404538000
H	-3.869868000	0.365959000	2.743313000
H	-5.138157000	0.258121000	-3.676045000
H	-4.096722000	1.653089000	-3.437385000
H	-5.370706000	1.284291000	-2.265847000
H	-1.715688000	1.774468000	-3.118217000
H	-0.323651000	0.614459000	-2.979875000
H	1.446038000	2.354853000	-3.709163000
H	-1.984006000	-3.584409000	-0.060281000
H	-3.078778000	-4.106011000	-1.339461000
H	-3.735241000	-3.450217000	0.157625000
H	-0.245669000	1.436969000	2.723873000
H	-0.106546000	3.164010000	2.377203000
H	0.702150000	1.987144000	1.337337000
H	0.141218000	-1.547538000	-2.823778000
H	-0.773467000	-3.043604000	-3.012625000
H	-0.097430000	-2.611400000	-1.435421000
H	5.250493000	2.369983000	-1.709919000

H	-3.585997000	1.990561000	3.371922000
H	-2.285920000	0.794683000	3.374814000
H	3.712482000	1.931663000	1.082529000
H	5.050173000	1.683219000	2.219888000
H	3.389812000	1.351365000	2.726271000
H	-3.033474000	-2.018601000	1.739369000
H	4.674473000	4.740548000	-5.250137000
H	0.474118000	-1.586764000	5.198443000
H	-1.643847000	-2.770137000	5.737077000
H	-3.391719000	-3.012549000	3.981986000
H	-0.003871000	0.107695000	-0.839620000
H	4.635699000	-1.119364000	3.774574000
H	6.158189000	-0.866865000	2.921039000
H	5.312005000	-2.404803000	2.762764000
H	6.132247000	3.957615000	-3.396979000
H	0.864475000	-0.703002000	2.923691000
H	5.820593000	-1.690656000	-0.272813000
H	6.401760000	-0.057270000	0.076664000
H	5.043545000	-0.295974000	-1.041619000
C	-0.451309000	3.509532000	-0.905279000
H	-0.383933000	4.531925000	-0.507752000
H	0.525813000	3.038544000	-0.771310000
H	-0.633790000	3.589520000	-1.979621000
Si	2.554751000	-3.035170000	1.408440000
C	2.609565000	-3.368312000	3.282132000
H	1.694933000	-3.878859000	3.596822000
H	3.461842000	-4.005913000	3.538355000
H	2.689894000	-2.450863000	3.867613000
C	3.995791000	-3.951939000	0.573398000
H	4.983795000	-3.680231000	0.953513000
H	3.865828000	-5.028392000	0.737021000
H	3.987675000	-3.776592000	-0.507219000
C	1.011395000	-3.858652000	0.708089000
H	1.045627000	-3.916727000	-0.382398000
H	0.997408000	-4.885797000	1.092448000
H	0.087111000	-3.369405000	1.017053000

^{1,2}TS1

Ti	-1.647559000	0.057722000	-0.046445000
Si	3.563073000	0.642037000	1.268421000
Si	3.587695000	-1.023536000	-1.362427000
N	2.622848000	-0.210511000	-0.051386000
C	-4.649909000	2.140185000	-0.826782000
C	-1.876797000	3.187900000	-1.822261000
C	-1.403661000	1.959885000	3.096418000
C	-4.372615000	1.204742000	2.170686000
C	0.125637000	3.082733000	0.618540000
C	-3.352446000	1.930142000	-0.106720000
C	-2.109636000	2.448777000	-0.542912000
C	-1.214648000	2.424148000	0.571825000
C	-1.910388000	1.902301000	1.687333000
C	-3.226454000	1.555721000	1.264377000
C	2.619159000	0.690489000	2.898856000
C	5.212384000	-0.204555000	1.657731000
C	3.923067000	2.410358000	0.706881000
C	2.554894000	-1.714050000	-2.771293000
C	4.443028000	-2.550025000	-0.626621000

C	4.859804000	0.179862000	-2.070084000
C	-2.251898000	-1.227248000	3.844840000
C	-2.370704000	-1.815610000	5.100500000
C	-1.325321000	-2.578702000	5.620021000
C	-0.156237000	-2.743127000	4.875132000
C	-0.032589000	-2.136921000	3.630216000
C	-1.077202000	-1.362096000	3.088214000
C	-0.918945000	-0.765600000	1.757461000
C	1.391353000	1.309977000	-4.659644000
C	2.128821000	1.960120000	-5.642858000
C	2.895306000	3.082780000	-5.328715000
C	2.913743000	3.549339000	-4.013658000
C	2.181423000	2.902233000	-3.024135000
C	1.400609000	1.762755000	-3.321539000
C	0.632539000	1.113206000	-2.312722000
C	0.196277000	-0.552872000	1.098610000
C	-0.080296000	0.593273000	-1.436940000
C	-5.130131000	-1.167807000	-0.339362000
C	-3.675099000	-0.175729000	-3.059080000
C	-1.021617000	-1.666022000	-3.247620000
C	-3.166171000	-3.000985000	1.175511000
C	-0.473005000	-3.228451000	-0.508047000
C	-3.056717000	-0.893456000	-1.898491000
C	-1.800177000	-1.549611000	-1.979782000
C	-1.606004000	-2.293863000	-0.788656000
C	-2.768297000	-2.132227000	0.021957000
C	-3.671093000	-1.277524000	-0.673986000
B	1.249125000	-0.272861000	0.192999000
H	-0.821200000	3.191185000	-2.097139000
H	-2.202751000	4.232804000	-1.721750000
H	-2.430802000	2.754822000	-2.659618000
H	-5.343130000	-0.620377000	0.581314000
H	-5.550763000	-2.173687000	-0.214059000
H	-5.689586000	-0.690282000	-1.146855000
H	-4.515327000	2.197360000	-1.907896000
H	-5.081842000	3.100750000	-0.511051000
H	-5.394555000	1.372505000	-0.615823000
H	2.100902000	1.589577000	-6.664393000
H	-5.301547000	1.664593000	1.816941000
H	-3.774232000	-0.853946000	-3.917149000
H	-3.064058000	0.669696000	-3.393364000
H	-4.672728000	0.201511000	-2.828969000
H	-0.876670000	-0.695409000	-3.726786000
H	-1.567836000	-2.312534000	-3.949185000
H	0.677749000	2.801153000	1.518849000
H	0.726209000	2.809903000	-0.253185000
H	0.789612000	0.443982000	-4.918067000
H	-2.307884000	-3.374549000	1.736749000
H	-3.721660000	-3.874792000	0.805321000
H	-3.817228000	-2.482011000	1.883637000
H	-2.107587000	1.524023000	3.807483000
H	-1.240586000	3.004611000	3.390335000
H	-0.454267000	1.432416000	3.226401000
H	0.486616000	-2.804368000	-0.819959000
H	-0.600225000	-4.182066000	-1.037733000
H	-0.392434000	-3.451617000	0.558813000
H	2.202955000	3.272649000	-2.004276000

H	-4.197291000	1.581545000	3.183358000
H	-4.565328000	0.130122000	2.256866000
H	4.492390000	2.439137000	-0.225441000
H	4.506916000	2.926663000	1.477556000
H	2.995896000	2.968871000	0.556786000
H	4.361639000	1.033425000	-2.538770000
H	5.438395000	-0.337363000	-2.843978000
H	5.568600000	0.555204000	-1.327181000
H	-3.071947000	-0.647937000	3.439115000
H	3.468444000	3.590026000	-6.099593000
H	1.973211000	-2.580456000	-2.445750000
H	3.256251000	-2.061445000	-3.539330000
H	1.887491000	-0.979305000	-3.216726000
H	0.660323000	-3.343017000	5.268727000
H	-1.422821000	-3.047669000	6.595420000
H	-3.285969000	-1.686774000	5.672312000
H	-0.041622000	-2.111219000	-3.086341000
H	1.707392000	1.286552000	2.842723000
H	3.277197000	1.146410000	3.648502000
H	2.345577000	-0.308358000	3.245806000
H	3.503130000	4.425621000	-3.755771000
H	0.871891000	-2.262098000	3.041315000
H	5.269489000	-2.328127000	0.049244000
H	4.840258000	-3.150339000	-1.453747000
H	3.718722000	-3.170973000	-0.088947000
H	5.069099000	-1.210753000	2.061094000
H	5.694544000	0.397679000	2.437247000
H	5.904816000	-0.260048000	0.815264000
H	0.024554000	4.176602000	0.629469000

[1,2]Int2

Ti	-1.650986000	0.019248000	-0.022051000
Si	3.987221000	0.667264000	1.778004000
Si	3.952745000	-1.087086000	-0.906044000
N	3.106454000	-0.307313000	0.514028000
C	-4.590788000	2.245183000	-0.623258000
C	-1.862216000	3.289854000	-1.555819000
C	-1.290648000	1.654181000	3.225649000
C	-4.281259000	0.985675000	2.313225000
C	0.255573000	2.825196000	0.783251000
C	-3.307138000	1.928167000	0.083326000
C	-2.057737000	2.448795000	-0.334057000
C	-1.132934000	2.277917000	0.735360000
C	-1.821603000	1.678980000	1.824020000
C	-3.163201000	1.425588000	1.411272000
C	2.976290000	0.656484000	3.368666000
C	5.677543000	-0.099352000	2.129355000
C	4.209690000	2.433229000	1.155490000
C	2.634321000	-1.744300000	-2.061815000
C	4.997259000	-2.536247000	-0.284063000
C	5.036799000	0.193051000	-1.765596000
C	-1.864251000	-1.561308000	3.721905000
C	-1.878731000	-2.235054000	4.940249000
C	-0.767803000	-2.974952000	5.343392000
C	0.358988000	-3.031983000	4.520137000
C	0.375004000	-2.341752000	3.314005000
C	-0.733751000	-1.584712000	2.889628000

C	-0.706347000	-0.877503000	1.601703000
C	1.037030000	1.195509000	-4.880089000
C	1.828188000	1.736212000	-5.887870000
C	2.751535000	2.742972000	-5.603312000
C	2.871993000	3.203578000	-4.291887000
C	2.085309000	2.664234000	-3.278739000
C	1.146142000	1.643775000	-3.546109000
C	0.336942000	1.097097000	-2.506692000
C	0.368753000	-0.566803000	0.873216000
C	-0.374505000	0.656379000	-1.594873000
C	-5.174657000	-0.895969000	-0.598744000
C	-3.309847000	-0.201662000	-3.156109000
C	-0.844074000	-2.065782000	-2.963756000
C	-3.599104000	-2.806383000	1.249059000
C	-0.814721000	-3.490375000	-0.147876000
C	-2.927274000	-0.950018000	-1.917090000
C	-1.758852000	-1.764915000	-1.821900000
C	-1.788604000	-2.440961000	-0.583052000
C	-2.995069000	-2.075239000	0.090449000
C	-3.710875000	-1.181202000	-0.752914000
B	1.758423000	-0.464240000	0.690100000
H	-0.807020000	3.406701000	-1.802883000
H	-2.289142000	4.289416000	-1.389616000
H	-2.355214000	2.861654000	-2.432732000
H	-5.444509000	-0.507218000	0.385946000
H	-5.736629000	-1.830097000	-0.735241000
H	-5.537928000	-0.194115000	-1.350007000
H	-4.565959000	1.989064000	-1.685290000
H	-4.773152000	3.327118000	-0.566851000
H	-5.452335000	1.749526000	-0.175793000
H	1.720110000	1.370779000	-6.906217000
H	-5.232522000	1.430731000	2.004841000
H	-3.472118000	-0.894383000	-3.993028000
H	-2.517213000	0.492193000	-3.456218000
H	-4.233883000	0.367007000	-3.028753000
H	-0.472221000	-1.154090000	-3.435492000
H	-1.384066000	-2.650649000	-3.721425000
H	0.852639000	2.307460000	1.538686000
H	0.752242000	2.702113000	-0.183136000
H	0.315563000	0.418243000	-5.114215000
H	-2.845737000	-3.208502000	1.927973000
H	-4.199368000	-3.652408000	0.884668000
H	-4.271926000	-2.175999000	1.837628000
H	-1.945892000	1.117743000	3.913133000
H	-1.192107000	2.682829000	3.596868000
H	-0.303639000	1.188075000	3.294171000
H	0.219706000	-3.149712000	-0.262994000
H	-0.933781000	-4.404764000	-0.743194000
H	-0.951016000	-3.760539000	0.901868000
H	2.182144000	3.031376000	-2.261002000
H	-4.099628000	1.309381000	3.343341000
H	-4.430917000	-0.099280000	2.338888000
H	4.814197000	2.470348000	0.245352000
H	4.713530000	3.034743000	1.920580000
H	3.243370000	2.897715000	0.941280000
H	4.431317000	1.008867000	-2.171502000
H	5.550695000	-0.288565000	-2.605349000

H	5.805120000	0.614895000	-1.110891000
H	-2.732897000	-1.002468000	3.394779000
H	3.365801000	3.165554000	-6.393532000
H	2.155777000	-2.634240000	-1.641593000
H	3.091450000	-2.029242000	-3.015776000
H	1.863186000	-0.992892000	-2.256786000
H	1.223576000	-3.617826000	4.821977000
H	-0.781814000	-3.511166000	6.288495000
H	-2.762077000	-2.191786000	5.571967000
H	0.016727000	-2.656395000	-2.646617000
H	2.026249000	1.184471000	3.254145000
H	3.546483000	1.164144000	4.155026000
H	2.758569000	-0.359203000	3.710413000
H	3.582375000	3.992348000	-4.055937000
H	1.246142000	-2.386280000	2.665705000
H	5.878960000	-2.228340000	0.282031000
H	5.341380000	-3.113129000	-1.150339000
H	4.401702000	-3.206610000	0.343757000
H	5.592828000	-1.130340000	2.483965000
H	6.156820000	0.488107000	2.921004000
H	6.343598000	-0.082144000	1.262548000
H	0.255576000	3.893602000	1.040100000

^{1,2}TS2

Ti	6.891745000	11.600340000	11.519645000
Si	12.312038000	12.425963000	13.107342000
Si	12.537507000	10.289123000	10.778104000
N	11.520972000	11.396086000	11.817949000
C	3.952178000	13.706855000	10.659074000
C	6.764705000	14.622824000	9.655411000
C	7.158811000	13.470407000	14.621083000
C	4.223209000	12.751205000	13.712962000
C	8.745008000	14.535481000	12.143161000
C	5.229803000	13.491554000	11.412991000
C	6.492567000	13.963147000	10.972643000
C	7.380957000	13.932129000	12.090460000
C	6.670954000	13.421896000	13.205999000
C	5.346355000	13.116972000	12.784850000
C	11.192073000	12.582594000	14.613830000
C	13.927460000	11.654071000	13.718616000
C	12.704006000	14.126265000	12.389522000
C	11.494584000	9.407525000	9.486450000
C	13.269518000	8.915954000	11.859533000
C	13.895894000	11.285841000	9.930426000
C	6.894207000	10.128167000	15.411851000
C	6.980286000	9.466104000	16.633140000
C	8.113831000	8.715247000	16.943904000
C	9.165455000	8.638908000	16.028009000
C	9.087116000	9.322580000	14.819990000
C	7.948970000	10.082798000	14.485601000
C	7.879281000	10.797097000	13.210789000
C	10.563662000	12.212663000	7.336544000
C	11.442707000	12.720103000	6.385389000
C	12.231698000	13.834991000	6.670850000
C	12.127692000	14.438238000	7.924992000
C	11.259128000	13.928441000	8.884994000
C	10.454172000	12.801338000	8.615597000

C	9.538910000	12.298753000	9.593112000
C	8.825131000	11.168855000	12.366479000
C	8.475181000	12.019870000	10.195970000
C	3.420200000	10.447960000	11.152731000
C	5.027546000	11.213414000	8.443194000
C	7.699684000	9.664408000	8.517331000
C	5.247236000	8.694017000	12.897862000
C	8.004552000	8.223395000	11.312917000
C	5.570569000	10.551271000	9.674623000
C	6.810633000	9.856019000	9.703880000
C	6.910806000	9.171601000	10.939586000
C	5.721131000	9.434869000	11.685709000
C	4.884664000	10.264889000	10.889144000
B	10.139469000	11.361011000	11.793187000
H	7.777905000	14.416835000	9.300868000
H	6.648994000	15.712915000	9.732804000
H	6.070905000	14.280675000	8.882773000
H	3.205702000	10.815665000	12.158782000
H	2.909692000	9.479908000	11.054891000
H	2.953098000	11.126613000	10.437806000
H	4.026810000	13.426607000	9.606494000
H	3.692070000	14.773881000	10.684833000
H	3.111487000	13.164541000	11.093035000
H	11.504445000	12.246182000	5.408960000
H	3.257834000	12.768984000	13.202102000
H	4.989779000	10.499904000	7.609699000
H	5.646558000	12.054962000	8.113870000
H	4.010331000	11.582237000	8.590346000
H	7.733664000	10.557012000	7.888361000
H	7.323321000	8.834695000	7.901567000
H	9.499559000	13.804771000	12.455683000
H	9.046152000	14.919002000	11.166260000
H	9.938059000	11.358353000	7.096167000
H	6.070328000	8.314061000	13.505090000
H	4.631699000	7.833219000	12.599652000
H	4.626116000	9.320740000	13.544353000
H	6.459268000	12.994312000	15.311261000
H	7.274151000	14.515088000	14.941499000
H	8.127699000	12.981411000	14.752215000
H	8.986381000	8.712167000	11.295209000
H	8.044264000	7.372156000	10.621575000
H	7.865757000	7.824803000	12.320966000
H	11.184869000	14.402693000	9.857276000
H	4.156153000	13.468852000	14.540262000
H	4.334112000	11.757326000	14.161495000
H	13.299293000	14.047488000	11.475653000
H	13.275793000	14.709437000	13.120189000
H	11.792255000	14.682629000	12.156663000
H	13.462917000	12.027509000	9.253203000
H	14.518543000	10.606377000	9.337382000
H	14.552947000	11.802576000	10.635506000
H	6.006974000	10.697319000	15.156312000
H	12.913318000	14.232224000	5.924108000
H	10.745220000	8.754408000	9.941449000
H	12.169773000	8.774562000	8.898202000
H	10.999470000	10.100547000	8.806997000
H	10.047968000	8.048613000	16.261488000

H	8.176376000	8.187818000	17.891949000
H	6.157031000	9.525337000	17.340225000
H	8.722807000	9.424016000	8.808624000
H	10.288489000	13.159858000	14.412213000
H	11.751506000	13.100221000	15.402094000
H	10.890232000	11.604760000	14.999047000
H	12.728146000	15.314037000	8.158440000
H	9.902184000	9.271892000	14.102746000
H	14.094370000	9.239298000	12.495741000
H	13.647440000	8.127583000	11.198196000
H	12.496689000	8.469255000	12.493252000
H	13.759540000	10.702199000	14.228952000
H	14.345965000	12.351716000	14.454075000
H	14.683029000	11.509895000	12.942849000
H	8.779122000	15.367437000	12.859229000

[1,2]Int3

Ti	-1.911967000	0.122901000	-0.144391000
Si	3.777097000	0.672803000	1.771293000
Si	4.082289000	-1.417837000	-0.516932000
N	3.081059000	-0.235167000	0.390268000
C	-5.006393000	1.982750000	-0.922748000
C	-2.318729000	3.038128000	-2.137301000
C	-1.556126000	2.108628000	2.833542000
C	-4.491736000	1.155750000	2.117850000
C	-0.216381000	3.227414000	0.253574000
C	-3.670410000	1.886772000	-0.251244000
C	-2.473844000	2.423488000	-0.780730000
C	-1.518605000	2.493138000	0.280364000
C	-2.126949000	1.978185000	1.457126000
C	-3.445092000	1.566002000	1.125344000
C	2.448534000	1.377645000	2.911328000
C	4.913845000	-0.416654000	2.825182000
C	4.785090000	2.138420000	1.111852000
C	3.270813000	-1.899382000	-2.152987000
C	4.246054000	-3.015515000	0.491835000
C	5.817651000	-0.754964000	-0.901106000
C	-1.396211000	-1.240240000	3.648247000
C	-1.184721000	-1.905722000	4.852715000
C	0.027449000	-2.550146000	5.095744000
C	1.023928000	-2.518964000	4.118218000
C	0.815338000	-1.843120000	2.920423000
C	-0.400802000	-1.176169000	2.656504000
C	-0.634709000	-0.499938000	1.383775000
C	0.743660000	1.541172000	-3.736446000
C	1.193897000	2.167676000	-4.893550000
C	2.523939000	2.578438000	-5.001077000
C	3.396878000	2.355856000	-3.936306000
C	2.946323000	1.725992000	-2.779526000
C	1.609684000	1.303187000	-2.653483000
C	1.164966000	0.675072000	-1.415080000
C	0.276283000	-0.111162000	0.473590000
C	-0.115911000	0.448585000	-0.940438000
C	-5.261338000	-1.308767000	-0.567207000
C	-3.664481000	-0.408831000	-3.227668000
C	-0.831793000	-1.706631000	-3.058306000
C	-3.298393000	-2.821695000	1.291277000

C	-0.517018000	-3.136142000	-0.287729000
C	-3.086404000	-1.007336000	-1.980265000
C	-1.782540000	-1.579436000	-1.909486000
C	-1.673610000	-2.273842000	-0.674008000
C	-2.892346000	-2.111453000	0.036824000
C	-3.780022000	-1.359642000	-0.789241000
B	1.761284000	0.091713000	-0.108693000
H	-1.277387000	3.041394000	-2.465823000
H	-2.665422000	4.080821000	-2.128313000
H	-2.906224000	2.513133000	-2.895788000
H	-5.531794000	-1.026010000	0.453429000
H	-5.683654000	-2.308336000	-0.737327000
H	-5.764677000	-0.629257000	-1.255469000
H	-4.967787000	1.725207000	-1.983391000
H	-5.364500000	3.019554000	-0.862200000
H	-5.763105000	1.358585000	-0.446274000
H	0.505331000	2.337687000	-5.717600000
H	-5.360473000	0.704496000	1.634509000
H	-3.741836000	-1.169450000	-4.015555000
H	-3.046030000	0.400955000	-3.627229000
H	-4.669727000	-0.014254000	-3.068220000
H	-0.989514000	-0.927875000	-3.807217000
H	-0.978320000	-2.674154000	-3.557964000
H	0.563825000	2.678626000	0.788440000
H	0.140070000	3.397574000	-0.764081000
H	-0.291134000	1.225154000	-3.653065000
H	-2.440894000	-3.068341000	1.919093000
H	-3.816656000	-3.760297000	1.049918000
H	-3.984985000	-2.221938000	1.895934000
H	-2.127969000	1.543424000	3.571540000
H	-1.572911000	3.162681000	3.142889000
H	-0.519620000	1.767406000	2.885133000
H	0.437557000	-2.621737000	-0.441830000
H	-0.502229000	-4.048661000	-0.898021000
H	-0.561653000	-3.434607000	0.761334000
H	3.624271000	1.564891000	-1.946364000
H	-4.854425000	2.027639000	2.678497000
H	-4.112944000	0.439293000	2.852197000
H	5.622082000	1.818688000	0.484835000
H	5.193887000	2.722826000	1.944227000
H	4.153538000	2.805516000	0.515765000
H	5.774742000	0.125955000	-1.549085000
H	6.382839000	-1.529334000	-1.432576000
H	6.387257000	-0.491800000	-0.005405000
H	-2.350555000	-0.762097000	3.454568000
H	2.874880000	3.068569000	-5.905032000
H	2.309590000	-2.397986000	-1.999138000
H	3.931142000	-2.611704000	-2.661483000
H	3.115377000	-1.046873000	-2.817669000
H	1.968710000	-3.029011000	4.290087000
H	0.191481000	-3.078359000	6.030989000
H	-1.973397000	-1.929945000	5.600624000
H	0.211746000	-1.650430000	-2.742097000
H	1.863416000	2.164812000	2.429076000
H	2.950280000	1.827451000	3.776243000
H	1.764908000	0.608395000	3.277889000
H	4.432869000	2.677562000	-4.006170000

H	1.593396000	-1.823620000	2.162487000
H	4.784460000	-2.876428000	1.432457000
H	4.786375000	-3.767114000	-0.095493000
H	3.257491000	-3.424270000	0.725461000
H	4.362523000	-1.243121000	3.283105000
H	5.325439000	0.196767000	3.635037000
H	5.759189000	-0.831784000	2.269528000
H	-0.324188000	4.205060000	0.742219000

[1,6]Int1

Ti	-1.963314000	0.260606000	0.010636000
Si	4.203019000	-1.095501000	-0.689945000
N	2.456926000	-1.330755000	-0.203234000
C	-4.165870000	2.891930000	-1.270869000
C	-1.083191000	3.354539000	-1.438776000
C	-2.224031000	1.679811000	3.284815000
C	-4.832739000	1.572833000	1.603943000
C	-3.180487000	2.389094000	-0.260140000
C	-1.786797000	2.685086000	-0.295990000
C	-1.244739000	2.423153000	0.991593000
C	-2.279734000	1.881292000	1.801055000
C	-3.478544000	1.854910000	1.024272000
C	5.144394000	-2.696889000	-0.327558000
C	4.359141000	-0.724364000	-2.536777000
C	5.007417000	0.239037000	0.382717000
C	-1.486934000	-1.783369000	3.444889000
C	-1.325754000	-2.174945000	4.771103000
C	-0.206993000	-1.764342000	5.495764000
C	0.751728000	-0.963216000	4.873753000
C	0.598973000	-0.590430000	3.541278000
C	-0.522692000	-0.997276000	2.791999000
C	-0.700847000	-0.571815000	1.406667000
C	2.713746000	3.534547000	-3.307943000
C	3.176925000	4.706222000	-3.896723000
C	3.794192000	5.688362000	-3.121860000
C	3.945570000	5.492015000	-1.749125000
C	3.484627000	4.324180000	-1.150858000
C	2.861767000	3.326583000	-1.923903000
C	2.392814000	2.124722000	-1.315224000
C	0.070882000	-0.264899000	0.383506000
C	2.013977000	1.088600000	-0.786684000
C	-5.261241000	-0.128454000	-1.667753000
C	-2.595790000	0.323855000	-3.438224000
C	-0.467240000	-1.693418000	-2.429690000
C	-4.573876000	-2.102960000	0.644359000
C	-1.650924000	-3.266829000	0.078038000
C	-2.722171000	-0.454715000	-2.161733000
C	-1.732633000	-1.376921000	-1.697029000
C	-2.275946000	-2.084799000	-0.589265000
C	-3.578809000	-1.568354000	-0.342597000
C	-3.877813000	-0.607794000	-1.351344000
B	1.517558000	-0.239019000	-0.156517000
H	-0.033404000	3.061182000	-1.496463000
H	-1.116575000	4.447127000	-1.330463000
H	-1.551695000	3.115085000	-2.397925000
H	-5.808156000	0.224831000	-0.789926000
H	-5.843393000	-0.961448000	-2.084994000

H	-5.266394000	0.668305000	-2.412408000
H	-3.897741000	2.639104000	-2.299948000
H	-4.209809000	3.988067000	-1.217676000
H	-5.175183000	2.523339000	-1.084261000
H	3.056509000	4.852762000	-4.966595000
H	-5.598027000	1.445960000	0.835676000
H	-2.598747000	-0.354333000	-4.301854000
H	-1.660716000	0.891627000	-3.486505000
H	-3.419990000	1.026456000	-3.579349000
H	-0.679728000	-2.291727000	-3.327151000
H	0.214111000	-2.270019000	-1.804638000
H	2.234759000	2.768133000	-3.909549000
H	-4.090603000	-2.528627000	1.527545000
H	-5.176919000	-2.902037000	0.192184000
H	-5.269074000	-1.330659000	0.984559000
H	-2.791341000	0.804755000	3.612867000
H	-2.645700000	2.555323000	3.798349000
H	-1.200625000	1.548457000	3.639133000
H	-0.599561000	-3.095213000	0.317735000
H	-1.701506000	-4.143797000	-0.580025000
H	-2.164002000	-3.527896000	1.006362000
H	3.601425000	4.169897000	-0.082571000
H	-5.155070000	2.404264000	2.245161000
H	-4.832307000	0.674610000	2.229401000
H	4.542673000	1.217652000	0.256884000
H	6.068201000	0.323431000	0.120017000
H	4.947586000	-0.042396000	1.439313000
H	-2.371230000	-2.086040000	2.892827000
H	4.155577000	6.601867000	-3.585518000
H	1.623748000	-0.627321000	5.429683000
H	-0.087662000	-2.056887000	6.535312000
H	-2.083419000	-2.793791000	5.245517000
H	0.059417000	-0.790926000	-2.754711000
H	5.025211000	-3.011542000	0.713299000
H	6.210327000	-2.509136000	-0.502649000
H	4.838567000	-3.529449000	-0.965628000
H	4.425632000	6.253060000	-1.140115000
H	1.349298000	0.033222000	3.062292000
H	3.961236000	-1.553054000	-3.131569000
H	5.415904000	-0.601695000	-2.800036000
H	3.827772000	0.187810000	-2.813872000
C	2.135875000	-2.662510000	0.210470000
C	1.978287000	-2.991309000	1.559695000
C	2.044965000	-3.688004000	-0.742325000
C	1.705288000	-4.303073000	1.945014000
C	1.780761000	-5.000377000	-0.356256000
C	1.603938000	-5.313759000	0.990766000
H	2.067976000	-2.213111000	2.308043000
H	2.188004000	-3.445862000	-1.791866000
H	1.574652000	-4.530107000	2.999481000
H	1.712749000	-5.778459000	-1.112071000
H	1.394595000	-6.335955000	1.293297000
C	0.127805000	2.769285000	1.468560000
H	0.071224000	3.374526000	2.381132000
H	0.721395000	1.876949000	1.695551000
H	0.676680000	3.340867000	0.718300000

[1,6]TS1’

Ti	-1.649957000	0.108190000	0.006628000
Si	4.002160000	0.168922000	1.283489000
N	2.689434000	-0.261551000	0.082498000
C	-4.603129000	2.167213000	-0.978904000
C	-1.865146000	3.329020000	-1.691414000
C	-1.666468000	1.953798000	3.188902000
C	-4.550152000	1.110240000	2.017219000
C	0.067082000	3.029164000	0.855440000
C	-3.377698000	1.949190000	-0.143661000
C	-2.122193000	2.517236000	-0.462615000
C	-1.297967000	2.439066000	0.698457000
C	-2.069139000	1.872909000	1.747652000
C	-3.347679000	1.525835000	1.219281000
C	3.333484000	-0.030648000	3.031609000
C	5.452916000	-1.000589000	0.997543000
C	4.526712000	1.958446000	0.993383000
C	-2.454841000	-1.206243000	3.769793000
C	-2.666284000	-1.750077000	5.033555000
C	-1.604104000	-2.313778000	5.739758000
C	-0.328546000	-2.327668000	5.171931000
C	-0.120163000	-1.773997000	3.914401000
C	-1.177758000	-1.194719000	3.187033000
C	-0.937062000	-0.653167000	1.844192000
C	1.017154000	1.426677000	-4.777517000
C	1.649091000	2.081484000	-5.828881000
C	2.339134000	3.275169000	-5.610733000
C	2.385750000	3.807569000	-4.321857000
C	1.754326000	3.156940000	-3.266617000
C	1.053813000	1.947078000	-3.466472000
C	0.394629000	1.291392000	-2.386387000
C	0.231353000	-0.439479000	1.272542000
C	-0.245390000	0.780113000	-1.458954000
C	-5.027572000	-0.985929000	-0.997602000
C	-2.860170000	-0.274586000	-3.290684000
C	-0.321077000	-1.992615000	-2.648198000
C	-3.643025000	-2.737133000	1.136642000
C	-0.672519000	-3.336837000	0.174560000
C	-2.615468000	-0.969038000	-1.986925000
C	-1.432974000	-1.715642000	-1.689747000
C	-1.614494000	-2.353472000	-0.443190000
C	-2.916626000	-2.026096000	0.037404000
C	-3.544176000	-1.196943000	-0.934857000
B	1.374162000	-0.293706000	0.499128000
H	-0.814562000	3.600781000	-1.788526000
H	-2.451702000	4.257857000	-1.643523000
H	-2.152100000	2.803379000	-2.605551000
H	-5.456241000	-0.598448000	-0.069877000
H	-5.514834000	-1.951409000	-1.190654000
H	-5.316763000	-0.315962000	-1.807564000
H	-4.467959000	1.868112000	-2.021374000
H	-4.842777000	3.239656000	-0.990219000
H	-5.476787000	1.647021000	-0.585963000
H	1.601501000	1.657091000	-6.828776000
H	-5.412129000	1.744367000	1.778649000
H	-2.877392000	-0.995390000	-4.118703000
H	-2.068495000	0.452195000	-3.502914000

H	-3.817999000	0.250740000	-3.303513000
H	-0.159077000	-1.157602000	-3.329346000
H	-0.560158000	-2.886123000	-3.242255000
H	0.636471000	2.505148000	1.630040000
H	0.627924000	2.955165000	-0.079644000
H	0.483336000	0.498815000	-4.958703000
H	-2.974405000	-3.068722000	1.932858000
H	-4.145406000	-3.629153000	0.735681000
H	-4.420174000	-2.116739000	1.591385000
H	-2.330907000	1.395279000	3.848844000
H	-1.683327000	3.003865000	3.509208000
H	-0.652176000	1.580046000	3.358141000
H	0.370778000	-3.047366000	0.015027000
H	-0.808492000	-4.333729000	-0.265460000
H	-0.824049000	-3.426518000	1.253210000
H	1.793398000	3.580809000	-2.266810000
H	-4.366384000	1.216548000	3.089605000
H	-4.860154000	0.075145000	1.839326000
H	4.982229000	2.094707000	0.008837000
H	5.265440000	2.252656000	1.747449000
H	3.671790000	2.636429000	1.074595000
H	-3.280419000	-0.778836000	3.215011000
H	2.829785000	3.785631000	-6.434825000
H	0.503012000	-2.771782000	5.713074000
H	-1.769523000	-2.745492000	6.723227000
H	-3.663348000	-1.741181000	5.466022000
H	0.626133000	-2.178736000	-2.138594000
H	2.413896000	0.536561000	3.193730000
H	4.088834000	0.328798000	3.739762000
H	3.131961000	-1.080980000	3.259257000
H	2.914767000	4.739458000	-4.137236000
H	0.866137000	-1.785058000	3.461138000
H	5.158762000	-2.044020000	1.145386000
H	6.250210000	-0.769336000	1.712734000
H	5.862036000	-0.901299000	-0.011154000
H	0.015588000	4.087237000	1.146990000
C	3.166690000	-0.607658000	-1.230693000
C	3.624278000	0.368201000	-2.115820000
C	3.265360000	-1.958148000	-1.582411000
C	4.163486000	-0.002719000	-3.346608000
C	3.804727000	-2.324775000	-2.813521000
C	4.257885000	-1.347597000	-3.698620000
H	3.526056000	1.415691000	-1.856256000
H	2.929230000	-2.714297000	-0.878548000
H	4.491364000	0.769042000	-4.036424000
H	3.874612000	-3.376689000	-3.076795000
H	4.678229000	-1.632839000	-4.658790000

^{1,6}Int2’

Ti	-1.738489000	0.159874000	-0.042540000
Si	4.150601000	0.159661000	1.258954000
N	2.804751000	-0.262150000	0.102388000
C	-4.838756000	2.028576000	-0.908166000
C	-2.193482000	3.411136000	-1.618763000
C	-1.829976000	1.885966000	3.204971000
C	-4.653330000	0.861397000	2.043776000
C	-0.210092000	3.153403000	0.905043000

C	-3.585576000	1.874398000	-0.100330000
C	-2.379355000	2.545909000	-0.412823000
C	-1.532505000	2.479318000	0.731860000
C	-2.242159000	1.812251000	1.766032000
C	-3.500776000	1.394342000	1.241415000
C	3.391422000	1.141566000	2.674986000
C	4.940395000	-1.438264000	1.879561000
C	5.415718000	1.196240000	0.323811000
C	-2.280035000	-1.347887000	3.690254000
C	-2.389740000	-1.902742000	4.962695000
C	-1.244511000	-2.280207000	5.662601000
C	0.011150000	-2.101775000	5.077559000
C	0.114622000	-1.545764000	3.808504000
C	-1.027662000	-1.148307000	3.087968000
C	-0.897600000	-0.595609000	1.730750000
C	0.966882000	1.899698000	-4.683512000
C	1.694535000	2.592752000	-5.644898000
C	2.511270000	3.665097000	-5.281883000
C	2.587420000	4.037379000	-3.939595000
C	1.860963000	3.348480000	-2.973145000
C	1.031704000	2.258497000	-3.319587000
C	0.286747000	1.555000000	-2.329838000
C	0.238606000	-0.317648000	1.108746000
C	-0.394672000	0.983260000	-1.470908000
C	-5.036266000	-1.142673000	-1.053725000
C	-2.953771000	-0.183823000	-3.333742000
C	-0.287860000	-1.728927000	-2.787394000
C	-3.489910000	-2.858275000	1.010599000
C	-0.512544000	-3.215623000	-0.016909000
C	-2.644232000	-0.911177000	-2.062309000
C	-1.405290000	-1.577127000	-1.806006000
C	-1.524950000	-2.272685000	-0.582731000
C	-2.840376000	-2.057848000	-0.075225000
C	-3.541093000	-1.246212000	-1.010903000
B	1.502172000	-0.254172000	0.546232000
H	-1.153414000	3.707464000	-1.752162000
H	-2.797142000	4.323724000	-1.510750000
H	-2.510724000	2.912609000	-2.538423000
H	-5.481570000	-0.818634000	-0.109782000
H	-5.454554000	-2.133852000	-1.276206000
H	-5.381650000	-0.467765000	-1.837335000
H	-4.692060000	1.803047000	-1.967672000
H	-5.174694000	3.073497000	-0.853803000
H	-5.654846000	1.408206000	-0.537228000
H	1.621707000	2.294898000	-6.688127000
H	-5.557337000	1.456078000	1.866152000
H	-2.941962000	-0.871389000	-4.189679000
H	-2.210603000	0.597196000	-3.526733000
H	-3.941120000	0.283150000	-3.310716000
H	-0.197182000	-0.853871000	-3.430820000
H	-0.472695000	-2.608195000	-3.420746000
H	0.457378000	2.551846000	1.531156000
H	0.281078000	3.296076000	-0.059190000
H	0.332983000	1.067962000	-4.976114000
H	-2.782282000	-3.153223000	1.787655000
H	-3.920152000	-3.778995000	0.591020000
H	-4.310715000	-2.320332000	1.494095000

H	-2.420238000	1.237383000	3.852647000
H	-1.957115000	2.917504000	3.559636000
H	-0.778347000	1.622293000	3.349544000
H	0.507003000	-2.876051000	-0.222258000
H	-0.621788000	-4.213031000	-0.463463000
H	-0.612517000	-3.323256000	1.066285000
H	1.924176000	3.646693000	-1.930260000
H	-4.443532000	0.916090000	3.115215000
H	-4.909220000	-0.178336000	1.815325000
H	5.822728000	0.659015000	-0.537018000
H	6.247599000	1.444262000	0.992395000
H	4.977589000	2.132977000	-0.032833000
H	-3.168697000	-1.059040000	3.143142000
H	3.077181000	4.205426000	-6.035612000
H	0.908275000	-2.399977000	5.614187000
H	-1.328605000	-2.716096000	6.654503000
H	-3.371000000	-2.044046000	5.408182000
H	0.677517000	-1.872925000	-2.298006000
H	2.966453000	2.083312000	2.315436000
H	4.168764000	1.379896000	3.409590000
H	2.600276000	0.585817000	3.185143000
H	3.215585000	4.873679000	-3.642385000
H	1.086506000	-1.414474000	3.340635000
H	4.209887000	-2.065722000	2.399565000
H	5.751187000	-1.211424000	2.580600000
H	5.363892000	-2.017420000	1.053640000
H	-0.321679000	4.134355000	1.387134000
C	3.187315000	-0.816888000	-1.173509000
C	3.428551000	0.010630000	-2.270060000
C	3.359504000	-2.200058000	-1.295310000
C	3.827081000	-0.545414000	-3.484345000
C	3.762304000	-2.750145000	-2.511257000
C	3.996871000	-1.923619000	-3.608876000
H	3.280040000	1.080005000	-2.177675000
H	3.173037000	-2.836270000	-0.434935000
H	3.992091000	0.107321000	-4.336290000
H	3.889549000	-3.825726000	-2.597944000
H	4.306974000	-2.351978000	-4.557796000

^{1,6}TS1

Ti	-2.335154000	-0.245147000	-0.086380000
Si	3.946115000	-1.940548000	0.094300000
N	2.138702000	-2.150789000	0.289277000
C	-4.784226000	2.391123000	-0.949696000
C	-1.697529000	2.913666000	-1.508213000
C	-2.322140000	1.205784000	3.277087000
C	-5.072434000	0.956093000	1.850846000
C	-3.642758000	1.843770000	-0.148845000
C	-2.269909000	2.171566000	-0.337948000
C	-1.583412000	1.885115000	0.879010000
C	-2.524746000	1.354970000	1.802370000
C	-3.785606000	1.285261000	1.155348000
C	4.789235000	-3.472614000	0.813303000
C	4.424025000	-1.781990000	-1.727099000
C	4.537708000	-0.460660000	1.111442000
C	-2.091489000	-2.327488000	3.395631000
C	-1.996185000	-2.735931000	4.723996000

C	-0.884938000	-2.386029000	5.490519000
C	0.134750000	-1.629441000	4.909792000
C	0.050928000	-1.246217000	3.574491000
C	-1.062962000	-1.592203000	2.784486000
C	-1.176864000	-1.161062000	1.393653000
C	2.468175000	2.299712000	-3.442419000
C	2.900124000	3.399620000	-4.175503000
C	3.472683000	4.496147000	-3.530527000
C	3.611168000	4.487115000	-2.142546000
C	3.181118000	3.392565000	-1.400036000
C	2.602838000	2.281423000	-2.041535000
C	2.161391000	1.155157000	-1.286190000
C	-0.289019000	-0.970721000	0.429504000
C	1.803064000	0.186982000	-0.629782000
C	-5.972274000	-1.445083000	-0.554140000
C	-4.457865000	0.068909000	-2.964211000
C	-1.374467000	-0.397680000	-2.206508000
C	-4.011200000	-3.601342000	0.560596000
C	-1.290898000	-3.400539000	-1.020615000
C	-3.844892000	-0.787726000	-1.898549000
C	-2.503233000	-1.302155000	-1.991361000
C	-2.402163000	-2.408912000	-1.086894000
C	-3.617209000	-2.491287000	-0.364193000
C	-4.512914000	-1.507335000	-0.882891000
B	1.231348000	-1.057780000	0.096333000
H	-0.658455000	2.633097000	-1.699561000
H	-1.719241000	3.997563000	-1.332851000
H	-2.264213000	2.727219000	-2.425449000
H	-6.156071000	-1.571462000	0.516692000
H	-6.516155000	-2.248638000	-1.069472000
H	-6.427104000	-0.500561000	-0.863490000
H	-4.525295000	2.578520000	-1.992705000
H	-5.086571000	3.355453000	-0.518080000
H	-5.666755000	1.747277000	-0.927756000
H	2.790075000	3.400200000	-5.256434000
H	-5.880706000	0.749152000	1.147169000
H	-4.635911000	-0.550406000	-3.853035000
H	-3.802547000	0.886512000	-3.278861000
H	-5.417838000	0.495187000	-2.672391000
H	-1.578845000	0.497147000	-2.791098000
H	-0.433224000	-0.857207000	-2.520657000
H	2.024098000	1.444483000	-3.942456000
H	-3.140912000	-4.046899000	1.048370000
H	-4.521248000	-4.403879000	0.009033000
H	-4.702461000	-3.263385000	1.339061000
H	-2.856802000	0.348844000	3.692913000
H	-2.695945000	2.106106000	3.785100000
H	-1.269700000	1.093803000	3.540256000
H	-0.390808000	-3.026156000	-1.512567000
H	-1.584157000	-4.323591000	-1.538647000
H	-1.016364000	-3.663461000	0.003314000
H	3.287486000	3.384270000	-0.319666000
H	-5.397876000	1.795355000	2.481218000
H	-4.968551000	0.086104000	2.507073000
H	4.109889000	0.481896000	0.767130000
H	5.629343000	-0.389544000	1.043413000
H	4.277348000	-0.597325000	2.166100000

H	-2.970448000	-2.571644000	2.808904000
H	3.809425000	5.353225000	-4.106769000
H	0.999662000	-1.337910000	5.500765000
H	-0.818114000	-2.688896000	6.531859000
H	-2.800787000	-3.317251000	5.167600000
H	-0.751444000	-0.332801000	-0.893401000
H	4.497745000	-3.642420000	1.853908000
H	5.873528000	-3.313091000	0.788131000
H	4.566336000	-4.382289000	0.250542000
H	4.056437000	5.337969000	-1.634345000
H	0.848068000	-0.658012000	3.127008000
H	4.124404000	-2.673634000	-2.286990000
H	5.511668000	-1.681780000	-1.816637000
H	3.958833000	-0.909009000	-2.189106000
C	1.739680000	-3.488182000	0.616568000
C	1.393187000	-3.863792000	1.915879000
C	1.768067000	-4.471540000	-0.382571000
C	1.053184000	-5.185681000	2.203831000
C	1.438759000	-5.792877000	-0.091926000
C	1.074420000	-6.156010000	1.204483000
H	1.382054000	-3.116901000	2.700217000
H	2.040085000	-4.185124000	-1.395026000
H	0.776361000	-5.452896000	3.220022000
H	1.462983000	-6.538544000	-0.882258000
H	0.813894000	-7.185701000	1.432925000
C	-0.173416000	2.247016000	1.219478000
H	-0.155986000	3.007745000	2.010559000
H	0.395427000	1.382675000	1.577761000
H	0.359140000	2.649520000	0.356725000

^{1,6}Int2

Ti	-2.398132000	0.359437000	-0.669504000
Si	4.616319000	-1.724473000	0.266949000
N	2.803323000	-1.687479000	0.052447000
C	-5.290566000	2.427941000	0.008766000
C	-3.685455000	3.020260000	-2.727267000
C	-0.327257000	2.727931000	1.064796000
C	-3.208842000	2.088041000	2.296075000
C	-3.813000000	2.315859000	-0.214239000
C	-3.104231000	2.580662000	-1.418202000
C	-1.713451000	2.607984000	-1.111594000
C	-1.568060000	2.416954000	0.290733000
C	-2.864979000	2.182264000	0.839064000
C	5.120322000	-3.494541000	0.693511000
C	5.463904000	-1.215297000	-1.342764000
C	5.119971000	-0.609067000	1.706193000
C	-1.459575000	-0.860379000	2.619377000
C	-1.334635000	-1.063578000	3.992620000
C	-0.129703000	-0.789411000	4.636309000
C	0.938123000	-0.293280000	3.886593000
C	0.808207000	-0.104616000	2.513287000
C	-0.387316000	-0.411849000	1.829659000
C	-0.545461000	-0.232885000	0.364522000
C	4.440639000	3.623870000	-1.998741000
C	5.027723000	4.873583000	-2.164521000
C	5.169195000	5.736536000	-1.077643000
C	4.717478000	5.341848000	0.181825000

C	4.127752000	4.094985000	0.358008000
C	3.982400000	3.217087000	-0.732125000
C	3.379419000	1.938002000	-0.552883000
C	0.542025000	-0.304432000	-0.450518000
C	2.883315000	0.831142000	-0.387233000
C	-5.809760000	-1.120205000	0.295658000
C	-5.247751000	0.045906000	-2.630226000
C	-2.052601000	-0.140053000	-2.797778000
C	-3.437382000	-3.013990000	1.016365000
C	-1.267921000	-2.855592000	-1.271713000
C	-4.250153000	-0.617146000	-1.730124000
C	-2.948372000	-1.072978000	-2.160101000
C	-2.463550000	-1.971363000	-1.143901000
C	-3.424714000	-2.024263000	-0.107088000
C	-4.512108000	-1.175331000	-0.452940000
B	2.089563000	-0.485812000	-0.200678000
H	-3.196797000	2.548044000	-3.585290000
H	-3.550794000	4.103444000	-2.845083000
H	-4.755632000	2.818790000	-2.797268000
H	-5.664377000	-0.938990000	1.365980000
H	-6.347444000	-2.073554000	0.204047000
H	-6.469458000	-0.340414000	-0.090260000
H	-5.859352000	2.392156000	-0.922114000
H	-5.520623000	3.390261000	0.485260000
H	-5.673400000	1.647276000	0.670510000
H	5.376990000	5.175375000	-3.148137000
H	-4.112379000	1.493382000	2.462138000
H	-5.868261000	-0.723438000	-3.109346000
H	-4.761027000	0.601451000	-3.434003000
H	-5.926014000	0.722230000	-2.106258000
H	-2.462460000	0.539059000	-3.541916000
H	-1.049269000	-0.486070000	-3.041595000
H	4.329079000	2.951116000	-2.843471000
H	-2.429058000	-3.281762000	1.340048000
H	-3.932172000	-3.938244000	0.686394000
H	-3.991630000	-2.651080000	1.887052000
H	-0.346587000	2.296031000	2.066270000
H	-0.238169000	3.817401000	1.174286000
H	0.573591000	2.370242000	0.560191000
H	-0.527397000	-2.437411000	-1.955867000
H	-1.574752000	-3.829071000	-1.677393000
H	-0.766498000	-3.028886000	-0.318601000
H	3.772657000	3.786643000	1.336436000
H	-3.398682000	3.086126000	2.714734000
H	-2.404866000	1.632246000	2.877357000
H	4.868349000	0.436557000	1.518261000
H	6.202032000	-0.679630000	1.865026000
H	4.623860000	-0.927365000	2.628654000
H	-2.411141000	-1.040072000	2.132739000
H	5.628634000	6.711764000	-1.211589000
H	1.874376000	-0.037269000	4.376888000
H	-0.029244000	-0.937141000	5.708180000
H	-2.186817000	-1.425643000	4.562844000
H	0.365845000	-0.056294000	-1.505665000
H	4.631501000	-3.847801000	1.605951000
H	6.203599000	-3.517406000	0.858856000
H	4.884586000	-4.200363000	-0.107291000

H	4.823701000	6.009599000	1.032342000
H	1.640963000	0.327135000	1.965537000
H	5.207435000	-1.907720000	-2.151052000
H	6.552145000	-1.234902000	-1.215826000
H	5.171464000	-0.207022000	-1.644085000
C	2.142986000	-2.955924000	0.141540000
C	1.606570000	-3.405315000	1.352364000
C	2.092820000	-3.795493000	-0.977266000
C	1.032861000	-4.673542000	1.439624000
C	1.523740000	-5.064217000	-0.885557000
C	0.993502000	-5.509769000	0.324661000
H	1.638966000	-2.753022000	2.219299000
H	2.505247000	-3.442032000	-1.918521000
H	0.620927000	-5.009009000	2.387765000
H	1.494524000	-5.704853000	-1.763009000
H	0.552368000	-6.499932000	0.397519000
C	-0.610730000	2.964793000	-2.060348000
H	-0.433088000	4.048917000	-2.068864000
H	0.329681000	2.483693000	-1.777992000
H	-0.848417000	2.662285000	-3.084003000

[1,6]Int3

Ti	-2.967530000	0.676089000	-0.120138000
Si	3.251927000	-1.533147000	-1.210190000
N	1.776541000	-1.644201000	-0.168634000
C	-5.952321000	2.359632000	0.733358000
C	-4.373325000	3.631911000	-1.771725000
C	-0.951452000	2.699962000	1.885895000
C	-3.879645000	2.055501000	3.035690000
C	-4.465259000	2.444026000	0.550000000
C	-3.769916000	2.959266000	-0.578150000
C	-2.385691000	3.012286000	-0.256438000
C	-2.217000000	2.585008000	1.094074000
C	-3.510594000	2.246524000	1.595283000
C	4.231019000	-3.142517000	-1.019464000
C	2.794389000	-1.375934000	-3.039686000
C	4.369333000	-0.110999000	-0.653990000
C	-3.539038000	-1.085977000	2.718231000
C	-4.070493000	-1.394194000	3.969237000
C	-3.288261000	-1.261079000	5.114430000
C	-1.969153000	-0.822662000	4.986993000
C	-1.441058000	-0.528222000	3.733233000
C	-2.210612000	-0.655397000	2.557316000
C	-1.642986000	-0.343227000	1.215431000
C	2.442423000	3.775728000	-1.740299000
C	2.918433000	4.845120000	-2.491645000
C	2.743593000	4.867760000	-3.875421000
C	2.089094000	3.809218000	-4.505595000
C	1.610203000	2.735952000	-3.761623000
C	1.778485000	2.702313000	-2.364288000
C	1.303581000	1.596091000	-1.597334000
C	-0.364110000	-0.793558000	1.013666000
C	0.944498000	0.618037000	-0.953785000
C	-5.505264000	0.706285000	-2.635884000
C	-2.397394000	0.891718000	-3.476382000
C	-1.016973000	-1.460549000	-1.985024000
C	-5.793584000	-1.386435000	-0.361614000

C	-2.971713000	-2.890354000	-0.073678000
C	-2.974939000	0.131185000	-2.320504000
C	-2.296014000	-1.028039000	-1.736920000
C	-3.216955000	-1.578940000	-0.751489000
C	-4.484485000	-0.948379000	-0.950628000
C	-4.351591000	0.057814000	-1.937835000
B	0.702252000	-0.662996000	-0.112572000
H	-3.834257000	3.417473000	-2.698082000
H	-4.327243000	4.719788000	-1.627104000
H	-5.423014000	3.372570000	-1.915598000
H	-6.285935000	1.044293000	-1.949729000
H	-5.969255000	-0.027508000	-3.308930000
H	-5.197439000	1.551985000	-3.250708000
H	-6.484761000	2.354230000	-0.220666000
H	-6.321988000	3.221849000	1.304126000
H	-6.250120000	1.462834000	1.285938000
H	3.430191000	5.664486000	-1.994159000
H	-4.741908000	1.397994000	3.164342000
H	-2.331132000	0.248067000	-4.364872000
H	-1.383420000	1.239958000	-3.256390000
H	-2.999586000	1.761988000	-3.748205000
H	-0.408158000	-1.008191000	-2.757263000
H	-0.660662000	-2.381470000	-1.545127000
H	2.581008000	3.755432000	-0.663515000
H	-5.668072000	-1.926229000	0.580273000
H	-6.302449000	-2.072919000	-1.051309000
H	-6.472150000	-0.547402000	-0.182750000
H	-1.001787000	2.110881000	2.803475000
H	-0.771619000	3.745834000	2.169285000
H	-0.087903000	2.347639000	1.315998000
H	-1.997234000	-2.903511000	0.422511000
H	-2.980184000	-3.698775000	-0.818570000
H	-3.732126000	-3.126080000	0.674573000
H	1.106077000	1.909561000	-4.253382000
H	-4.145957000	3.032166000	3.463538000
H	-3.063786000	1.642509000	3.629241000
H	3.863172000	0.853818000	-0.725812000
H	5.270099000	-0.074424000	-1.277251000
H	4.686029000	-0.258796000	0.383736000
H	-4.161770000	-1.200048000	1.836038000
H	3.117223000	5.704088000	-4.459456000
H	-1.347033000	-0.709809000	5.871707000
H	-3.698862000	-1.495320000	6.092712000
H	-5.098320000	-1.740488000	4.048681000
H	0.014786000	-1.416916000	1.834991000
H	4.561870000	-3.309484000	0.009317000
H	5.120934000	-3.084922000	-1.656966000
H	3.650758000	-4.016516000	-1.329007000
H	1.952451000	3.818268000	-5.583586000
H	-0.413663000	-0.185614000	3.648210000
H	2.162249000	-2.214572000	-3.348794000
H	3.706127000	-1.400604000	-3.647433000
H	2.265741000	-0.445350000	-3.253208000
C	1.729793000	-2.788231000	0.689399000
C	2.335015000	-2.753199000	1.952967000
C	1.105948000	-3.973545000	0.281867000
C	2.313049000	-3.869858000	2.786029000

C	1.082522000	-5.090427000	1.116055000
C	1.685306000	-5.044017000	2.371837000
H	2.815645000	-1.833198000	2.274659000
H	0.648869000	-4.016203000	-0.702371000
H	2.786665000	-3.820833000	3.763201000
H	0.593523000	-6.001136000	0.779868000
H	1.667046000	-5.914636000	3.021383000
C	-1.340724000	3.661992000	-1.108403000
H	-1.482440000	4.751297000	-1.106007000
H	-0.338129000	3.457361000	-0.735917000
H	-1.376587000	3.336264000	-2.151372000

^{1,6}TS2

Ti	-2.960200000	0.670691000	-0.097400000
Si	3.206573000	-1.542047000	-1.263808000
N	1.729627000	-1.672530000	-0.237710000
C	-5.978490000	2.327916000	0.697928000
C	-4.376155000	3.602381000	-1.793752000
C	-0.994391000	2.726601000	1.912327000
C	-3.934556000	2.069380000	3.034232000
C	-4.490196000	2.431060000	0.536759000
C	-3.785399000	2.942629000	-0.586929000
C	-2.405793000	3.006369000	-0.249180000
C	-2.251109000	2.594104000	1.109336000
C	-3.547943000	2.252440000	1.597448000
C	4.193590000	-3.149790000	-1.092303000
C	2.769340000	-1.355524000	-3.096859000
C	4.322723000	-0.126716000	-0.684101000
C	-3.509887000	-1.058023000	2.777975000
C	-4.019648000	-1.345957000	4.042747000
C	-3.222542000	-1.179687000	5.173157000
C	-1.910077000	-0.729786000	5.017679000
C	-1.403530000	-0.454110000	3.750783000
C	-2.189531000	-0.612485000	2.589879000
C	-1.647220000	-0.331482000	1.232543000
C	2.419282000	3.769040000	-1.697500000
C	2.910975000	4.851814000	-2.418992000
C	2.753190000	4.906251000	-3.803942000
C	2.100540000	3.865654000	-4.465122000
C	1.606143000	2.779335000	-3.750747000
C	1.756088000	2.713496000	-2.352383000
C	1.264458000	1.594200000	-1.614915000
C	-0.377126000	-0.788987000	1.000071000
C	0.895647000	0.606426000	-0.991548000
C	-5.385090000	0.683759000	-2.712783000
C	-2.247928000	0.828185000	-3.430225000
C	-0.944679000	-1.530670000	-1.842779000
C	-5.779206000	-1.355889000	-0.391460000
C	-2.996117000	-2.898971000	0.000995000
C	-2.877526000	0.079736000	-2.293788000
C	-2.237427000	-1.072749000	-1.663086000
C	-3.199166000	-1.599430000	-0.712320000
C	-4.449200000	-0.948311000	-0.955068000
C	-4.267401000	0.036295000	-1.957501000
B	0.630256000	-0.700155000	-0.190344000
H	-3.814211000	3.394991000	-2.708267000
H	-4.349237000	4.691034000	-1.650298000

H	-5.418401000	3.326868000	-1.959240000
H	-6.189008000	1.045531000	-2.066397000
H	-5.829990000	-0.059237000	-3.388430000
H	-5.043066000	1.512691000	-3.332189000
H	-6.495184000	2.296521000	-0.264273000
H	-6.370188000	3.194711000	1.246482000
H	-6.271007000	1.436831000	1.262198000
H	3.421906000	5.656569000	-1.897332000
H	-4.788315000	1.399847000	3.157289000
H	-2.209928000	0.200913000	-4.331891000
H	-1.219637000	1.112515000	-3.188394000
H	-2.797632000	1.737210000	-3.687012000
H	-0.333775000	-1.148401000	-2.651019000
H	-0.671476000	-2.494589000	-1.436402000
H	2.545316000	3.723648000	-0.619914000
H	-5.688683000	-1.833063000	0.588068000
H	-6.260176000	-2.087766000	-1.053867000
H	-6.464570000	-0.509657000	-0.291940000
H	-1.053401000	2.154995000	2.840263000
H	-0.819446000	3.777992000	2.177334000
H	-0.124621000	2.365260000	1.357512000
H	-2.045006000	-2.911522000	0.540149000
H	-2.978068000	-3.719869000	-0.729659000
H	-3.793660000	-3.115369000	0.715538000
H	1.104422000	1.966663000	-4.267251000
H	-4.222116000	3.045952000	3.448215000
H	-3.120700000	1.674982000	3.643040000
H	3.815783000	0.838660000	-0.742652000
H	5.225724000	-0.079381000	-1.303514000
H	4.636023000	-0.289612000	0.352474000
H	-4.142495000	-1.201235000	1.906473000
H	3.139049000	5.752829000	-4.364840000
H	-1.276187000	-0.593949000	5.890704000
H	-3.615895000	-1.399013000	6.161901000
H	-5.041584000	-1.703582000	4.144487000
H	0.030623000	-1.387647000	1.825645000
H	4.512739000	-3.333563000	-0.062578000
H	5.091216000	-3.075228000	-1.717139000
H	3.623117000	-4.021779000	-1.424904000
H	1.977852000	3.898849000	-5.544354000
H	-0.380708000	-0.104428000	3.642544000
H	2.153206000	-2.196711000	-3.430617000
H	3.689010000	-1.356126000	-3.693178000
H	2.231662000	-0.427552000	-3.300283000
C	1.696039000	-2.809350000	0.627845000
C	2.266434000	-2.747496000	1.907456000
C	1.121724000	-4.019146000	0.216492000
C	2.256787000	-3.856782000	2.750359000
C	1.110110000	-5.129192000	1.060236000
C	1.676501000	-5.053533000	2.331297000
H	2.711639000	-1.810923000	2.232292000
H	0.697351000	-4.087474000	-0.781002000
H	2.703618000	-3.784864000	3.738746000
H	0.660550000	-6.058198000	0.718790000
H	1.668003000	-5.918795000	2.988236000
C	-1.354499000	3.658628000	-1.091269000
H	-1.499418000	4.747499000	-1.089634000

H	-0.354924000	3.456373000	-0.709660000
H	-1.379253000	3.332026000	-2.134061000

[1,6]Int4

Ti	-2.564176000	0.629381000	-0.344145000
Si	3.021505000	-1.438169000	-0.968674000
N	1.453539000	-1.531861000	-0.134598000
C	-5.226935000	1.826648000	1.887637000
C	-5.165369000	3.115109000	-0.911782000
C	-0.356751000	3.014956000	0.851138000
C	-2.206674000	1.883513000	3.041089000
C	-3.966376000	2.088575000	1.119027000
C	-3.938935000	2.629281000	-0.201663000
C	-2.592496000	2.984312000	-0.511778000
C	-1.793097000	2.660340000	0.623162000
C	-2.647073000	2.123806000	1.633233000
C	4.037510000	-2.980884000	-0.527858000
C	2.798226000	-1.458375000	-2.852862000
C	4.061324000	0.077610000	-0.487648000
C	-3.949023000	-1.188482000	2.732124000
C	-4.437785000	-1.730177000	3.917984000
C	-3.554510000	-2.279851000	4.846206000
C	-2.186671000	-2.299913000	4.565645000
C	-1.702235000	-1.771806000	3.374550000
C	-2.574954000	-1.180180000	2.440448000
C	-2.097192000	-0.648854000	1.161509000
C	2.334736000	3.621108000	-1.773924000
C	2.897159000	4.735050000	-2.388715000
C	2.684253000	4.973841000	-3.746281000
C	1.907309000	4.083451000	-4.487954000
C	1.344129000	2.966050000	-3.880944000
C	1.543338000	2.715384000	-2.508783000
C	0.977236000	1.565825000	-1.877154000
C	-0.815528000	-0.546567000	0.728988000
C	0.546965000	0.557582000	-1.326018000
C	-4.681023000	1.097622000	-3.333604000
C	-1.681430000	0.537925000	-3.796585000
C	-0.745087000	-1.728204000	-1.700609000
C	-5.730672000	-0.750614000	-0.943270000
C	-3.407362000	-2.807475000	-0.327202000
C	-2.474308000	-0.031849000	-2.659988000
C	-2.097484000	-1.133707000	-1.829577000
C	-3.242066000	-1.510256000	-1.060353000
C	-4.306554000	-0.603957000	-1.389679000
C	-3.839593000	0.260880000	-2.421636000
B	0.149188000	-0.829300000	-0.633584000
H	-4.967811000	3.399190000	-1.945394000
H	-5.540521000	4.011242000	-0.398724000
H	-5.980916000	2.387148000	-0.905705000
H	-5.659375000	1.334453000	-2.914490000
H	-4.856126000	0.535366000	-4.261094000
H	-4.192688000	2.031893000	-3.623960000
H	-5.934341000	1.198128000	1.337490000
H	-5.740073000	2.775729000	2.092995000
H	-5.027542000	1.348029000	2.847639000
H	3.507663000	5.418599000	-1.804837000
H	-2.935125000	1.311045000	3.616559000

H	-2.068568000	0.145900000	-4.747338000
H	-0.629056000	0.268032000	-3.717858000
H	-1.743528000	1.629248000	-3.845858000
H	-0.231270000	-1.766217000	-2.667117000
H	-0.822702000	-2.759357000	-1.346458000
H	2.509210000	3.431551000	-0.719296000
H	-5.803810000	-1.118031000	0.084125000
H	-6.261145000	-1.473379000	-1.577501000
H	-6.280454000	0.192258000	-1.001510000
H	0.239515000	2.152847000	1.164081000
H	-0.287067000	3.774189000	1.641342000
H	0.102518000	3.424456000	-0.048205000
H	-2.486580000	-3.117110000	0.169950000
H	-3.684564000	-3.593171000	-1.042695000
H	-4.191568000	-2.759246000	0.431289000
H	0.749524000	2.269865000	-4.463570000
H	-2.056872000	2.846429000	3.547115000
H	-1.255593000	1.345185000	3.079561000
H	3.531619000	1.006654000	-0.710956000
H	5.008106000	0.083595000	-1.040380000
H	4.300885000	0.067468000	0.581007000
H	-4.632494000	-0.769590000	2.001502000
H	3.124752000	5.844250000	-4.224359000
H	-1.491920000	-2.744221000	5.273126000
H	-3.929125000	-2.702389000	5.774639000
H	-5.506510000	-1.726612000	4.116606000
H	-0.094926000	-0.516777000	1.557324000
H	4.309610000	-3.017175000	0.530421000
H	4.963112000	-2.983144000	-1.115155000
H	3.487048000	-3.897665000	-0.761854000
H	1.743470000	4.257457000	-5.548143000
H	-0.639373000	-1.824886000	3.159139000
H	2.290632000	-2.374255000	-3.172692000
H	3.782686000	-1.434535000	-3.334840000
H	2.225205000	-0.600122000	-3.210092000
C	1.414251000	-2.362740000	1.005862000
C	2.127846000	-2.021189000	2.172117000
C	0.675713000	-3.561797000	1.037530000
C	2.123681000	-2.842131000	3.299842000
C	0.656680000	-4.373390000	2.169157000
C	1.383910000	-4.023768000	3.308025000
H	2.691136000	-1.091467000	2.176707000
H	0.137891000	-3.866098000	0.145709000
H	2.696832000	-2.551174000	4.177176000
H	0.082039000	-5.296521000	2.155485000
H	1.377867000	-4.665766000	4.184588000
C	-2.127989000	3.735269000	-1.723454000
H	-2.082372000	4.812000000	-1.513947000
H	-1.130085000	3.422890000	-2.040096000
H	-2.805530000	3.600811000	-2.570481000

^{1,6}TS3

Ti	-2.593452000	0.650418000	-0.309795000
Si	3.089314000	-1.376062000	-0.830823000
N	1.443308000	-1.537341000	-0.147526000
C	-5.188724000	1.890757000	1.953344000
C	-5.196683000	3.124327000	-0.875812000

C	-0.339505000	3.049400000	0.762431000
C	-2.144359000	1.959451000	3.031132000
C	-3.948238000	2.150406000	1.152038000
C	-3.953574000	2.658943000	-0.180684000
C	-2.613772000	3.002632000	-0.532034000
C	-1.786299000	2.705676000	0.590620000
C	-2.617041000	2.196017000	1.633094000
C	4.118074000	-2.872699000	-0.279379000
C	3.043014000	-1.442440000	-2.725821000
C	4.012020000	0.183109000	-0.265044000
C	-3.911838000	-1.181385000	2.814908000
C	-4.363749000	-1.756103000	3.999072000
C	-3.453846000	-2.348636000	4.874753000
C	-2.096633000	-2.374158000	4.547201000
C	-1.647705000	-1.806726000	3.360071000
C	-2.547091000	-1.178053000	2.478092000
C	-2.098076000	-0.599360000	1.215882000
C	2.337457000	3.644617000	-1.903456000
C	2.855040000	4.774306000	-2.528761000
C	2.643734000	4.985011000	-3.891206000
C	1.913068000	4.051658000	-4.627057000
C	1.393575000	2.919023000	-4.009260000
C	1.592935000	2.696169000	-2.632591000
C	1.067339000	1.532679000	-1.992249000
C	-0.866696000	-0.353606000	0.723840000
C	0.653907000	0.517769000	-1.444508000
C	-4.726207000	1.092448000	-3.303304000
C	-1.722361000	0.554703000	-3.772891000
C	-0.772947000	-1.730421000	-1.693236000
C	-5.752846000	-0.750055000	-0.895267000
C	-3.408817000	-2.783574000	-0.271891000
C	-2.507489000	-0.018930000	-2.631723000
C	-2.122627000	-1.114867000	-1.799755000
C	-3.260991000	-1.497080000	-1.027756000
C	-4.334113000	-0.603190000	-1.359215000
C	-3.876440000	0.262951000	-2.392221000
B	0.222227000	-0.838215000	-0.747191000
H	-5.016783000	3.398940000	-1.915311000
H	-5.575943000	4.021774000	-0.368000000
H	-6.003382000	2.386613000	-0.851363000
H	-5.703332000	1.326735000	-2.879290000
H	-4.904803000	0.527477000	-4.228681000
H	-4.244581000	2.028464000	-3.599419000
H	-5.907694000	1.258032000	1.422990000
H	-5.699861000	2.839031000	2.167353000
H	-4.965378000	1.417185000	2.910944000
H	3.428875000	5.492465000	-1.949251000
H	-2.857472000	1.380618000	3.620011000
H	-2.159871000	0.222340000	-4.724362000
H	-0.683701000	0.227764000	-3.741290000
H	-1.723525000	1.648875000	-3.780237000
H	-0.307901000	-1.836712000	-2.682217000
H	-0.871499000	-2.741512000	-1.293004000
H	2.508916000	3.478260000	-0.844473000
H	-5.812177000	-1.098547000	0.139892000
H	-6.288017000	-1.486430000	-1.509589000
H	-6.307669000	0.189391000	-0.963572000

H	0.265453000	2.176730000	1.026005000
H	-0.225887000	3.791095000	1.563618000
H	0.080251000	3.477272000	-0.148505000
H	-2.488801000	-3.069079000	0.241834000
H	-3.666568000	-3.589047000	-0.972642000
H	-4.200889000	-2.733802000	0.478484000
H	0.833213000	2.189914000	-4.585758000
H	-1.990051000	2.921168000	3.537998000
H	-1.189664000	1.425933000	3.048665000
H	3.466909000	1.088699000	-0.539604000
H	5.001878000	0.224349000	-0.734427000
H	4.159128000	0.183239000	0.820270000
H	-4.614421000	-0.730488000	2.121936000
H	3.049401000	5.867760000	-4.377421000
H	-1.384142000	-2.851474000	5.214347000
H	-3.801432000	-2.800114000	5.800165000
H	-5.424236000	-1.747518000	4.237598000
H	-0.057167000	-0.252160000	1.454752000
H	4.318756000	-2.879124000	0.795042000
H	5.080834000	-2.849641000	-0.803213000
H	3.619858000	-3.813962000	-0.531718000
H	1.750262000	4.204740000	-5.690590000
H	-0.594988000	-1.855766000	3.099950000
H	2.598671000	-2.385019000	-3.062560000
H	4.066804000	-1.397630000	-3.115405000
H	2.476992000	-0.616926000	-3.161251000
C	1.320872000	-2.454547000	0.924030000
C	1.920002000	-2.162209000	2.164696000
C	0.651676000	-3.686586000	0.809799000
C	1.870148000	-3.058873000	3.231165000
C	0.581115000	-4.573143000	1.882581000
C	1.192695000	-4.270272000	3.098992000
H	2.436491000	-1.211968000	2.274056000
H	0.217109000	-3.963372000	-0.144586000
H	2.358072000	-2.805045000	4.169289000
H	0.061734000	-5.520259000	1.758138000
H	1.149095000	-4.972128000	3.927134000
C	-2.181206000	3.724917000	-1.772922000
H	-2.198404000	4.811185000	-1.613641000
H	-1.164705000	3.455360000	-2.069526000
H	-2.841111000	3.512482000	-2.618007000

^{1,6}Int5

Ti	-2.772052000	0.831789000	-0.112850000
Si	3.223567000	-1.277481000	-0.029926000
N	1.539094000	-1.831082000	-0.460090000
C	-4.982809000	2.240112000	2.260067000
C	-5.605450000	2.973893000	-0.705370000
C	-0.539880000	3.511450000	0.000665000
C	-1.869484000	2.765038000	2.763850000
C	-3.945101000	2.531984000	1.217503000
C	-4.220872000	2.782239000	-0.162896000
C	-2.992767000	3.122693000	-0.797011000
C	-1.966900000	3.101315000	0.201492000
C	-2.570304000	2.787779000	1.445519000
C	4.001287000	-2.586208000	1.088426000
C	4.273139000	-1.121735000	-1.592818000

C	3.138759000	0.347658000	0.930294000
C	-3.710986000	-1.072756000	3.335736000
C	-3.948178000	-1.656266000	4.578170000
C	-2.891706000	-1.888529000	5.457688000
C	-1.595669000	-1.528111000	5.081586000
C	-1.361999000	-0.948609000	3.839409000
C	-2.413950000	-0.711968000	2.931113000
C	-2.164624000	-0.106587000	1.626302000
C	2.802209000	3.387434000	-2.355287000
C	3.257639000	4.531958000	-3.000468000
C	2.910272000	4.776706000	-4.329247000
C	2.100741000	3.868850000	-5.012430000
C	1.636100000	2.724350000	-4.373856000
C	1.980697000	2.467591000	-3.033427000
C	1.509203000	1.290753000	-2.379434000
C	-1.116386000	0.251466000	0.919078000
C	1.113004000	0.273940000	-1.826685000
C	-5.053569000	0.771787000	-3.018298000
C	-1.964735000	0.607633000	-3.606607000
C	-0.762216000	-1.687136000	-1.748508000
C	-5.695304000	-1.039433000	-0.483223000
C	-3.045412000	-2.665718000	0.171806000
C	-2.653045000	0.010347000	-2.416478000
C	-2.094638000	-0.995071000	-1.566343000
C	-3.142896000	-1.479421000	-0.732936000
C	-4.333448000	-0.762318000	-1.049977000
C	-4.040699000	0.117376000	-2.129878000
B	0.628516000	-1.074846000	-1.254899000
H	-5.608877000	3.132772000	-1.784274000
H	-6.061948000	3.861492000	-0.247064000
H	-6.270082000	2.131528000	-0.488057000
H	-6.015469000	0.910758000	-2.521861000
H	-5.235275000	0.131348000	-3.892467000
H	-4.729312000	1.741709000	-3.403070000
H	-5.788543000	1.613228000	1.865304000
H	-5.446930000	3.167798000	2.622008000
H	-4.556958000	1.725053000	3.124635000
H	3.888594000	5.235328000	-2.464281000
H	-2.396637000	2.149314000	3.496081000
H	-1.915314000	-0.129402000	-4.421240000
H	-0.939091000	0.904115000	-3.377321000
H	-2.497748000	1.478832000	-3.993324000
H	-0.622939000	-1.835189000	-2.833513000
H	-0.858738000	-2.696494000	-1.336898000
H	3.074052000	3.193319000	-1.322567000
H	-5.645071000	-1.344828000	0.565971000
H	-6.191556000	-1.852527000	-1.029811000
H	-6.351796000	-0.166390000	-0.541539000
H	0.124280000	3.000964000	0.703376000
H	-0.416746000	4.592071000	0.155388000
H	-0.185668000	3.284368000	-1.007674000
H	-2.112918000	-2.669321000	0.739808000
H	-3.078202000	-3.594600000	-0.414044000
H	-3.868184000	-2.701729000	0.889096000
H	1.008027000	2.015083000	-4.903797000
H	-1.791755000	3.780579000	3.173670000
H	-0.855661000	2.364947000	2.671120000

H	2.576258000	1.107827000	0.384329000
H	4.150685000	0.727194000	1.111571000
H	2.659803000	0.192895000	1.902507000
H	-4.535495000	-0.888486000	2.652882000
H	3.269742000	5.670844000	-4.830649000
H	-0.765498000	-1.696790000	5.763348000
H	-3.074989000	-2.340595000	6.428660000
H	-4.961738000	-1.928445000	4.862283000
H	-0.036570000	0.216425000	1.041202000
H	3.425034000	-2.735054000	2.005841000
H	5.007069000	-2.253683000	1.369881000
H	4.091950000	-3.554705000	0.589153000
H	1.828953000	4.053776000	-6.047954000
H	-0.353491000	-0.657506000	3.556033000
H	4.346561000	-2.087926000	-2.102505000
H	5.288702000	-0.804520000	-1.330208000
H	3.854600000	-0.394460000	-2.291476000
C	1.187796000	-3.143865000	-0.001655000
C	0.569610000	-3.324762000	1.239807000
C	1.484761000	-4.266973000	-0.782413000
C	0.255163000	-4.605824000	1.691290000
C	1.169607000	-5.546676000	-0.328741000
C	0.555829000	-5.721207000	0.911030000
H	0.322956000	-2.454044000	1.839636000
H	1.958365000	-4.124161000	-1.749957000
H	-0.231352000	-4.726810000	2.655271000
H	1.403370000	-6.408666000	-0.948031000
H	0.310252000	-6.718700000	1.264308000
C	-2.814137000	3.697297000	-2.170679000
H	-2.799494000	4.794788000	-2.124759000
H	-1.872642000	3.384095000	-2.629425000
H	-3.623503000	3.416752000	-2.848070000

^{1,6}Int6

Ti	0.180227000	-1.861211000	-0.222900000
Si	-2.934383000	2.570634000	3.533036000
N	-1.849290000	2.706850000	2.075354000
C	2.971531000	-2.943829000	-1.900858000
C	2.927409000	-4.346755000	-1.823563000
H	2.113086000	-4.809554000	-1.273034000
B	-0.811268000	1.800479000	1.690311000
C	3.899647000	-5.137986000	-2.430947000
H	3.840398000	-6.221012000	-2.354809000
C	4.947256000	-4.544505000	-3.133566000
H	5.707169000	-5.159139000	-3.608564000
C	5.011655000	-3.151874000	-3.221187000
H	5.824756000	-2.679790000	-3.767507000
C	4.039438000	-2.364686000	-2.614851000
H	4.090598000	-1.281308000	-2.687963000
C	1.943975000	-2.116294000	-1.275819000
C	-0.568753000	-2.074854000	2.148643000
C	0.064099000	-0.802469000	2.027157000
C	1.433023000	-1.030861000	1.699227000
C	1.637491000	-2.434073000	1.592071000
C	0.391159000	-3.081329000	1.840588000
C	-1.900907000	-2.311333000	2.791925000
H	-2.702824000	-1.702275000	2.367664000

H	-1.843875000	-2.067554000	3.862189000
H	-2.208101000	-3.356178000	2.723032000
C	1.648750000	-0.842108000	-1.180724000
H	2.047634000	0.110356000	-1.519532000
C	0.816349000	2.664552000	-0.351363000
C	0.083242000	2.221840000	0.521277000
C	-3.900680000	4.183998000	3.699071000
H	-3.240904000	5.045496000	3.833716000
H	-4.548515000	4.111327000	4.580197000
H	-4.531931000	4.379739000	2.828232000
C	-1.228856000	-1.030378000	-2.005456000
C	-2.070714000	-1.396863000	-0.905300000
C	-2.010088000	-2.811199000	-0.764600000
C	-1.101837000	-3.308793000	-1.747575000
C	-0.672177000	-2.217656000	-2.545166000
C	-1.110770000	0.340811000	-2.597866000
H	-0.142528000	0.483563000	-3.085308000
H	-1.209948000	1.121849000	-1.840195000
H	-1.888606000	0.508597000	-3.355763000
C	-0.528776000	0.486727000	2.553813000
H	0.161666000	0.844660000	3.338111000
H	-1.443125000	0.228311000	3.098051000
C	2.530582000	-0.015149000	1.678205000
H	3.222380000	-0.200612000	0.849256000
H	3.115108000	-0.057863000	2.607574000
H	2.141266000	0.998050000	1.565323000
C	2.975665000	-3.096680000	1.486932000
H	2.902408000	-4.120902000	1.114457000
H	3.457188000	-3.138448000	2.473914000
H	3.646572000	-2.554935000	0.815677000
C	0.216013000	-4.566518000	1.968323000
H	0.757023000	-5.110393000	1.187071000
H	-0.832639000	-4.868949000	1.914423000
H	0.605030000	-4.923709000	2.931168000
C	1.651072000	3.206039000	-1.369772000
C	3.012694000	2.858113000	-1.447840000
H	3.423821000	2.166418000	-0.719389000
C	3.818815000	3.399091000	-2.443595000
H	4.868987000	3.125139000	-2.493144000
C	3.282677000	4.286814000	-3.376859000
H	3.914277000	4.704234000	-4.156095000
C	1.934396000	4.639797000	-3.306010000
H	1.515259000	5.333730000	-4.029373000
C	1.122086000	4.111198000	-2.309153000
H	0.075053000	4.388655000	-2.240409000
C	-2.030227000	3.906839000	1.304945000
C	-2.990769000	3.955858000	0.290235000
H	-3.583862000	3.069862000	0.081704000
C	-3.176759000	5.122352000	-0.450693000
H	-3.926497000	5.145024000	-1.237161000
C	-2.405374000	6.253066000	-0.186107000
H	-2.551204000	7.162403000	-0.762314000
C	-1.444098000	6.207803000	0.823536000
H	-0.833385000	7.081667000	1.034043000
C	-1.258459000	5.043641000	1.565478000
H	-0.503824000	4.999717000	2.345562000
C	-1.913035000	2.340846000	5.109244000

H	-1.173445000	3.141684000	5.209133000
H	-1.387181000	1.384271000	5.149282000
H	-2.579962000	2.394174000	5.977293000
C	-4.176170000	1.161822000	3.314118000
H	-4.751238000	1.296909000	2.392873000
H	-4.881399000	1.164188000	4.152946000
H	-3.704198000	0.177183000	3.279118000
C	-3.048657000	-0.466979000	-0.250281000
H	-3.865570000	-0.225330000	-0.944076000
H	-2.594144000	0.482963000	0.046833000
H	-3.505368000	-0.906260000	0.638866000
C	-2.937501000	-3.690158000	0.017895000
H	-3.652516000	-4.170934000	-0.663530000
H	-3.521002000	-3.134030000	0.752597000
H	-2.415021000	-4.495208000	0.542944000
C	-0.833927000	-4.762106000	-2.002508000
H	-1.657154000	-5.217447000	-2.570012000
H	-0.737674000	-5.331106000	-1.071932000
H	0.081623000	-4.909714000	-2.579786000
C	0.167345000	-2.290513000	-3.778721000
H	0.764220000	-3.204383000	-3.817999000
H	0.860149000	-1.447006000	-3.842074000
H	-0.468921000	-2.267281000	-4.673246000

^{1,6}TS4

Ti	1.664153000	4.471019000	3.770822000
Si	-1.301566000	8.443732000	7.532391000
N	-0.525388000	8.105467000	5.917512000
C	4.101117000	2.659429000	2.048222000
C	3.967707000	1.304145000	2.390004000
H	3.246563000	1.026577000	3.149753000
B	0.444013000	7.082338000	5.717083000
C	4.740283000	0.321499000	1.776090000
H	4.617800000	-0.718910000	2.067089000
C	5.660466000	0.664911000	0.786848000
H	6.257822000	-0.102378000	0.301817000
C	5.802439000	2.004777000	0.422862000
H	6.514880000	2.287008000	-0.348494000
C	5.037402000	2.984677000	1.046056000
H	5.154593000	4.027180000	0.762025000
C	3.324858000	3.731621000	2.676740000
C	0.956242000	3.586005000	6.025917000
C	1.562129000	4.851983000	6.206126000
C	2.939898000	4.739814000	5.842393000
C	3.186661000	3.391152000	5.488534000
C	1.956971000	2.680914000	5.552201000
C	-0.374454000	3.207976000	6.604181000
H	-1.168920000	3.910518000	6.341883000
H	-0.307920000	3.194338000	7.701379000
H	-0.694634000	2.213005000	6.293334000
C	3.451778000	5.017230000	2.760347000
H	4.185623000	5.763975000	2.479171000
C	2.172213000	7.117172000	3.619110000
C	1.245839000	6.889585000	4.415017000
C	-2.113866000	10.145865000	7.453678000
H	-1.395360000	10.916810000	7.159311000
H	-2.489604000	10.398139000	8.451791000

H	-2.951747000	10.185997000	6.754235000
C	-0.108396000	5.369711000	2.134460000
C	-0.767568000	4.655898000	3.179851000
C	-0.399625000	3.284381000	3.060293000
C	0.527306000	3.168192000	1.987636000
C	0.683470000	4.459901000	1.403265000
C	-0.410551000	6.782763000	1.744926000
H	0.454221000	7.303148000	1.326931000
H	-0.763529000	7.375203000	2.589753000
H	-1.200511000	6.795399000	0.981040000
C	0.913560000	6.052387000	6.833275000
H	1.636769000	6.550019000	7.494215000
H	0.091829000	5.735250000	7.486203000
C	4.007460000	5.775993000	6.014270000
H	4.607801000	5.891584000	5.105081000
H	4.692758000	5.503788000	6.828353000
H	3.584547000	6.754342000	6.254564000
C	4.563684000	2.836416000	5.311540000
H	4.563574000	1.797322000	4.980410000
H	5.103047000	2.884284000	6.267149000
H	5.141090000	3.411749000	4.581657000
C	1.806968000	1.190970000	5.430443000
H	1.482743000	0.857095000	4.438989000
H	1.076041000	0.808416000	6.150546000
H	2.755870000	0.689519000	5.643667000
C	3.120828000	7.949799000	2.926237000
C	3.868233000	8.879588000	3.671961000
H	3.754032000	8.905938000	4.751148000
C	4.744048000	9.755487000	3.037624000
H	5.312420000	10.467373000	3.630018000
C	4.894751000	9.718884000	1.651825000
H	5.580024000	10.402217000	1.158284000
C	4.164892000	8.794935000	0.904647000
H	4.279415000	8.755592000	-0.175125000
C	3.290121000	7.913763000	1.532699000
H	2.737192000	7.182959000	0.953700000
C	-0.879234000	8.997634000	4.852735000
C	-2.177561000	8.996414000	4.328076000
H	-2.905499000	8.288865000	4.715199000
C	-2.531043000	9.878206000	3.308322000
H	-3.542330000	9.860968000	2.911062000
C	-1.591002000	10.769204000	2.793509000
H	-1.864628000	11.453674000	1.995737000
C	-0.295980000	10.773257000	3.309970000
H	0.446610000	11.461323000	2.915520000
C	0.056659000	9.900206000	4.336623000
H	1.063794000	9.906746000	4.741121000
C	0.002756000	8.511271000	8.901210000
H	0.839226000	9.156048000	8.613761000
H	0.401003000	7.531750000	9.173559000
H	-0.456987000	8.944973000	9.796696000
C	-2.605629000	7.131077000	7.920623000
H	-3.399757000	7.131003000	7.167665000
H	-3.065944000	7.339933000	8.892787000
H	-2.179898000	6.124669000	7.961282000
C	-1.904928000	5.207792000	3.986468000
H	-2.723175000	5.527356000	3.327340000

H	-1.623981000	6.080083000	4.584326000
H	-2.320237000	4.461328000	4.667420000
C	-1.144540000	2.112940000	3.628032000
H	-1.737534000	1.644081000	2.830793000
H	-1.843158000	2.406463000	4.412755000
H	-0.494590000	1.334102000	4.033609000
C	0.910742000	1.875927000	1.334360000
H	0.097233000	1.533551000	0.677727000
H	1.091422000	1.077474000	2.058664000
H	1.809986000	1.971801000	0.724000000
C	1.442810000	4.748049000	0.147747000
H	2.522188000	4.613801000	0.282197000
H	1.265474000	5.770693000	-0.197690000
H	1.128212000	4.076995000	-0.660514000

^{1,6}Int7

Ti	0.027228000	-1.527404000	-0.070708000
Si	-2.067755000	3.006095000	3.103729000
N	-1.510556000	2.321218000	1.513533000
C	2.606295000	-2.179508000	-2.084602000
C	2.446093000	-3.568814000	-1.935543000
H	1.698424000	-3.922485000	-1.233414000
B	-0.547712000	1.259318000	1.407012000
C	3.210539000	-4.488761000	-2.649600000
H	3.052622000	-5.554008000	-2.498389000
C	4.174755000	-4.045769000	-3.551625000
H	4.775089000	-4.756114000	-4.113345000
C	4.354577000	-2.671856000	-3.725640000
H	5.097638000	-2.307403000	-4.430925000
C	3.586355000	-1.760383000	-3.011278000
H	3.744443000	-0.700963000	-3.188836000
C	1.761355000	-1.250336000	-1.307120000
C	-0.491778000	-2.116974000	2.199498000
C	0.320020000	-0.953645000	2.265952000
C	1.620016000	-1.295794000	1.794694000
C	1.616182000	-2.674213000	1.451321000
C	0.312414000	-3.187552000	1.683638000
C	-1.832599000	-2.241200000	2.862390000
H	-2.542537000	-1.473590000	2.543819000
H	-1.717148000	-2.141819000	3.949679000
H	-2.289137000	-3.215421000	2.681812000
C	2.001395000	0.092672000	-1.341674000
H	2.839141000	0.506452000	-1.910213000
C	1.242309000	1.047134000	-0.532536000
C	0.149234000	0.625036000	0.173234000
C	-2.753035000	4.741235000	2.799852000
H	-2.017836000	5.372718000	2.291823000
H	-2.981881000	5.200717000	3.768259000
H	-3.664352000	4.740649000	2.197770000
C	-1.488153000	-0.792060000	-1.776480000
C	-2.293095000	-1.342573000	-0.734300000
C	-2.060255000	-2.742517000	-0.696925000
C	-1.094196000	-3.061525000	-1.693266000
C	-0.760281000	-1.860346000	-2.377927000
C	-1.593499000	0.577095000	-2.367882000
H	-0.609614000	0.995803000	-2.595103000
H	-2.099904000	1.274678000	-1.701074000

H	-2.162458000	0.531785000	-3.306996000
C	-0.122492000	0.406701000	2.715648000
H	0.703605000	0.904683000	3.238662000
H	-0.928850000	0.311822000	3.452134000
C	2.847428000	-0.445951000	1.836764000
H	3.266992000	-0.289160000	0.837684000
H	3.618591000	-0.927358000	2.450961000
H	2.645426000	0.535738000	2.268071000
C	2.855702000	-3.476440000	1.203839000
H	2.638790000	-4.468372000	0.802260000
H	3.386876000	-3.620412000	2.155220000
H	3.541515000	-2.979137000	0.514517000
C	-0.016285000	-4.651494000	1.706439000
H	0.221109000	-5.156654000	0.764643000
H	-1.070264000	-4.832103000	1.923385000
H	0.561886000	-5.155416000	2.491808000
C	1.826133000	2.420789000	-0.450436000
C	2.020005000	3.080234000	0.772301000
H	1.750783000	2.573054000	1.694053000
C	2.556314000	4.365711000	0.826030000
H	2.701262000	4.848973000	1.789016000
C	2.915279000	5.027114000	-0.347485000
H	3.332806000	6.029381000	-0.307865000
C	2.737291000	4.384657000	-1.572729000
H	3.010297000	4.889111000	-2.496269000
C	2.208219000	3.097260000	-1.621342000
H	2.063768000	2.613602000	-2.583998000
C	-2.054730000	2.997731000	0.370935000
C	-3.424402000	2.894132000	0.086395000
H	-4.051148000	2.273639000	0.720244000
C	-3.983771000	3.567271000	-0.998096000
H	-5.047304000	3.469211000	-1.199979000
C	-3.182947000	4.356735000	-1.821798000
H	-3.615284000	4.878687000	-2.670856000
C	-1.823529000	4.474348000	-1.538269000
H	-1.186810000	5.093809000	-2.164117000
C	-1.264564000	3.811316000	-0.446912000
H	-0.211620000	3.923990000	-0.220628000
C	-0.630819000	3.204661000	4.322197000
H	0.233000000	3.670167000	3.837727000
H	-0.308650000	2.267088000	4.779187000
H	-0.957597000	3.873104000	5.127016000
C	-3.424709000	1.933639000	3.878625000
H	-4.308514000	1.889039000	3.234515000
H	-3.734092000	2.366646000	4.836724000
H	-3.092144000	0.908861000	4.067827000
C	-3.335185000	-0.598039000	0.043021000
H	-4.009216000	-0.065256000	-0.636938000
H	-2.903554000	0.151995000	0.712819000
H	-3.945401000	-1.277310000	0.643247000
C	-2.918566000	-3.764872000	-0.016484000
H	-3.726837000	-4.062321000	-0.698992000
H	-3.392452000	-3.390750000	0.891820000
H	-2.369086000	-4.674694000	0.232496000
C	-0.771067000	-4.457749000	-2.138998000
H	-1.641395000	-4.903647000	-2.639405000
H	-0.513114000	-5.121425000	-1.307227000

H	0.059141000	-4.476155000	-2.846737000
C	-0.064002000	-1.749690000	-3.698129000
H	0.620929000	-2.578542000	-3.882431000
H	0.509329000	-0.822699000	-3.774110000
H	-0.812178000	-1.746390000	-4.502578000

References

[1] V. Varga, K. Mach, M. Polášek, P. Sedmera, J. Hiller, U. Thewalt, S. I. Troyanov, *J. Organomet. Chem.* **1996**, *506*, 241-251.

[2] W. Ma, I. Douair, L. Maron, Q. Ye, *Chem. Eur. J.* **2020**, *26*, 13573-13577.

[3] F. Gründler, H. Scholz, M. Herbig, S. Schwarzer, J. Wagler, E. Kroke, *Eur. J. Inorg. Chem.* **2021**, *2021*, 2211-2224.

[4] R. A. Bartlett, X. Feng, M. M. Olmstead, P. P. Power, K. J. Weese, *J. Am. Chem. Soc.* **1987**, *109*, 4851-4854.

[5] Sheldrick, G.M. SHELXT – Integrated space-group and crystal-structure determination. *Acta Crystallographica Section A Foundations and Advances*. **2015**, *71*, 3-8.

[6] Dolomanov, O.V., Bourhis, L.J., Gildea, R.J., Howard, J.A.K. & Puschmann, H. OLEX2: a complete structure solution, refinement and analysis program. *J. Appl. Crystallogr*. **2009**, *42*, 339-341.

[7] Sheldrick, G.M. A short history of SHELX. *Acta Crystallographica Section A Foundations of Crystallography*. **2008**, *64*, 112-122.

[8] A. D. Becke, *J. Chem. Phys*., 1993, **98**, 5648

[9] (a) A. Bergner, M. Dolg, W. Kuechle, H. Stoll, H. Preuss, *Mol. Phys*. 1993, **80**, 1431 (b) W. Kuechle, M. Dolg, H. Stoll, H. Preuss, Mol. Phys. 1991, **74**, 1245.

[10] A. Hollwarth, M. Bohme, S. Dapprich, A.W. Ehlers, A. Gobbi, V. Jonas, K.F. Kohler, R. Stegmann, A. Veldkamp, G. Frenking *J. Chem. Phys*. 1993, **208**, 237.

[11] (a) R. Ditchfield, W. J. Hehre and J. A. Pople, *J. Chem. Phys*., 1971, **54**, 724; (b) W. J. Hehre, R. Ditchfield and J. A. Pople, *J. Chem. Phys*., 1972, **56**, 2257; (c) P. C. Hariharan and J. A. Pople, *Theor. Chem. Acc*., 1973, **28**, 213.

[12] Gaussian 09, Revision D.01: M. J. Frisch, G. W. Trucks, H. B. Schlegel, G. E. Scuseria, M. A. Robb, J. R. Cheesman, G. Scalmani, V. Barone, B. Mennucci, G. A. Petersson, H. Nakatsuji, M. Caricato, X. Li, H. P. Hratchian, A. F. Izmaylov, J. Bloino, G. Zheng, J. L. Sonnenberg, M. Hada, M. Ehara, K. Toyota, R. Fukuda, J. Hasegawa, M. Ishida, T. Nakajima, Y. Honda, O. Kitao, H. Nakai, T. Vreven, J. A., Jr. Montgomery, J. E. Peralta, F. Ogliaro, M. Bearpark, J. J. Heyd, E. Brothers, K. N. Kudin, V. N. Staroverov, R. Kobayashi, J. Normand, K. Raghavachari, J. C. Burant, S. S. Iyengar, J. Tomasi, M. Cossi, N. Rega, M. J.

Millam, M. Klene, J. E. Knox, J. B. Cross, V. Bakken, C. Adamo, J. Jaramillo, R. Gomperts, R. E. Stratmann, O. Yazyev, A. J. Austin, R. Cammi, C. Pomelli, J. W. Ochterski, R. L. Martin, K. Morokuma, V. G. Zakrzewski, G. A. Voth, P. Salvador, J. J. Dannenberg, S. Dapprich, A. D. Daniels, O. Farkas, J. B. Foresman, J. V. Ortiz, J. Cioslowski and D. J. Fox, Gaussian Inc., 2009, Wallingford CT.
